# Identification of Neurensin-2 as a novel modulator of emotional behavior

**DOI:** 10.1038/s41380-021-01058-5

**Published:** 2021-03-19

**Authors:** Gali Umschweif, Lucian Medrihan, Andrés Guillén-Samander, Wei Wang, Yotam Sagi, Paul Greengard

**Affiliations:** 1 Laboratory for Molecular and Cellular Neuroscience, The Rockefeller University, New York, NY USA; 2 Department of Cell Biology, Yale School of Medicine, New Haven, CT USA

**Keywords:** Neuroscience, Depression

## Abstract

Among the hallmarks of major depressive disorders (MDD) are molecular, functional, and morphological impairments in the hippocampus. Recent studies suggested a key role for hippocampal GABAergic interneurons both in depression and in the response to its treatments. These interneurons highly express the chromatin-remodeler SMARCA3 which mediates the response to chronic antidepressants in an unknown mechanism. Using cell-type-specific molecular and physiological approaches, we report that SMARCA3 mediates the glutamatergic signaling in interneurons by repressing the expression of the neuronal protein, Neurensin-2. This vesicular protein associates with endosomes and postsynaptic proteins and is highly and selectively expressed in subpopulations of GABAergic interneurons. Upregulation of Neurensin-2 in the hippocampus either by stress, viral overexpression, or by SMARCA3 deletion, results in depressive-like behaviors. In contrast, the deletion of Neurensin-2 confers resilience to stress and induces AMPA receptor localization to synapses. This pathway which bidirectionally affects emotional behavior could be involved in neuropsychiatric disorders, and suggests novel therapeutic approaches.

## Introduction

The hippocampus is a key brain region in the neuronal circuit that regulates cognitive and emotional processing. Therefore, it is implicated in many neurological and neuropsychiatric disorders, including depression [1]. In depression, the hippocampus is highly subjected to plastic changes. Human and preclinical studies indicate that in the depressed brain, the granule cells in the hippocampal dentate gyrus (DG) undergo functional and morphological impairments. These include dendritic atrophy accompanied by volume reduction [2], spine loss [3], attenuated activity [4], and downregulation of α-amino-3-hydroxy-5-methyl-4-isoxazole propionic acid receptor (AMPAR) subunits [5]. In contrast, therapeutic electroconvulsive treatment induced DG volume in depressed individuals who exhibit clinical improvement [6]. In rodents, this treatment enhanced the excitability of granule cells, an effect that was blocked by augmentation of synaptic inhibition. These densely packed principal granule neurons are massively inhibited by local GABAergic interneurons, which regulate the excitability and activity of the principal cells [7]. Accumulating evidence implicate these local inhibitory interneurons in the treatment of depression. Chronic treatment with selective serotonin reuptake inhibitors (SSRIs) induce dynamic expression of serotonergic receptors on cholecystokinin (CCK)-and parvalbumin (PV)-expressing interneurons which modulate their activity [8, 9]. CCK interneurons are essential for feedback inhibition in the DG [10]. These interneurons play a central role in the initiation of the behavioral response to SSRIs [9], an effect that is mediated by the antidepressive-like protein, p11 [9, 11, 12]. To mediate antidepressant-like behavior, p11 forms a protein complex with the chromatin-remodeler factor SMARCA3, which is highly expressed in DG interneurons [13]. SMARCA3 is a member of the SWI/SNF chromatin-remodelers family that regulates gene transcription [14]. SWI/SNF chromatin remodelers use the energy of ATP hydrolysis to slide the DNA around the nucleosome and therefore, alter the accessibility of genomic regions for the transcription regulatory machinery [15]. However, the genes that are regulated by SMARCA3 in the adult brain and their roles in emotional behavior, remain unknown. In this study we aimed to elucidate the molecular mechanism by which SMARCA3 is implicated in neuronal function and emotional behavior.

## Materials and methods

### Animals

All procedures were approved by The Rockefeller University Institutional Animal Care and Use Committee and were in accordance with the National Institutes of Health Guide for the Care and Use of Laboratory Animals guidelines. Mice were maintained on a C57BL/6 background and were kept on a 12 h light/dark cycle with food and water ad libitum. C57BL/6 mice were purchased from Jackson Laboratories. For social defeat studies, CD-1 mice were purchased from Charles River Laboratories. SMARCA3 conditional KO mice were generated by crossing mice that harbor the Hltf gene flanked by lox cassettes [13] with either CCK^tm1.1(cre)Zjh/J^, GAD2^tm2(cre)Zjh/J^, and Pvalb^tm1(cre)Arbr/J^ mice. For TRAP experiments, CCK^tm1.1(cre)Zjh/J^, GAD2^tm2(cre)Zjh/J^, and Pvalb^tm1(cre)Arbr/J^ mice were crossed with mice expressing loxP-stop-loxP-EGFP-RPL10a sequence in the Eef1α1 promoter (EEF1A1–LSL.EGFPL10) [16]. In order to analyze the expression levels of Neurensin-2 in somatostatin-expressing neurons, Tg(Cort-cre)42Gsat mice were crossed with mice expressing loxP-stop-loxP-EGFP-RPL10a [8]. For behavioral tests, TRAP-sequencing, ATAC-sequencing, and biochemistry, only males were used. For immunohistochemistry, qPCR, and electrophysiology, both males and females were used, with no differences observed between genders.

### Generation of Nrsn2 KO mice

The Nrsn2 knockout mouse model was generated at The Rockefeller University CRISPR and Genome Editing Resource Center. Briefly, six CRISPR gRNAs targeting Nrsn2 exon 2 were designed using Benchling (https://www.benchling.com) and CRISPOR (http://crispor.tefor.net) softwares. All were subject to in vivo validation, in mESCs and in zygotes for cleavage efficiency and indels pattern. A guide targeting Nrsn2 exon 2 site (TGGAGGAAAGTACATGGTATGGG) which mediated a 5 bp deletion by SpCas9 was chosen. This 5 bp deletion creates a frame-shift mutation, introducing a premature stop codon (UGA) to translate a truncated Nrsn2 protein (21 amino acids) instead of its WT protein (202 amino acids). The selected guide was delivered with Cas9 in ctRNP complex (CrRNA, tracrRNA, HiFi Cas9; IDT cat# 1072532, 1081060) into mouse zygotes via pronuclear injection as well as iGONAD (improved-Genome editing via Oviductal Nucleic Acid Delivery) [17]. Pups carrying the desired 5 bp deletion in exon 2, confirmed by PCR and Sanger sequencing were chosen as founders.

### TRAP-ATAC-seq

#### Nuclei preparation and sorting

Adult GAD2^TRAP^ mice were used for ATAC-seq experiments. Six hippocampi from three mice were quickly dissected on ice and washed with ice-cold PBS. Nuclei preparation and sorting was conducted as previously described [18], with mild modifications. Samples were homogenized in ice-cold homogenization buffer containing (all in mM) 320 sucrose, 5 CaCl2, 3 MgCl2, 10 Tris, pH = 8.0, 0.1 EDTA, and 0.1% IPEGAL supplemented with protease inhibitor cocktail, complete-EDTAfree (Roche, Mannheim, Germany). For gradient centrifugation, 50% OptiPrep density gradient medium (Sigma Aldrich) containing (all in mM) 5 CaCl2, 3 MgCl2, 10 Tris pH = 8, was added and mixed. The lysate was gently loaded on the top of 10 ml 29% OptiPrep solution. Samples were centrifuged for 30 min at 4 °C using a swinging bucket rotor in a WX Ultra 80 centrifuge (Thermo Fisher Scientific). The nuclei pellet was gently resuspended in isotonic buffer, supplemented with protease inhibitor, 10 μM Dyecycle Ruby (Invitrogen, Carlsbad, CA) and 1% BSA. Samples were then incubated for 30 min and subjected to sorting using a fluorescence activated sorter (FACS). Nuclear preparation was sorted with a FACSAria (BD, San Jose, CA, USA) cell sorter equipped with 640 and 488 nm excitation lasers and a 70 μm nozzle. Nuclei were gated by two criteria: the presence of a GFP signal above the background fluorescence level (as assessed by comparison with nuclei obtained from WT, a non-GFP expressing littermate mouse) and the signal from DyeCycle ruby corresponding to a single nucleus. 50,000 sorted nuclei were collected in 100 μL of isotonic buffer supplemented with 5 mM CaCl2. All GFP− samples were validated for GFP+ nuclei by post sorting. Random samples from GFP− and GFP+ were analyzed for nuclei purity and integrity by ImageStream imaging flow cytometer (Amnis). Images were acquired (×5000 cells/sample; objective ×40) and data were analyzed with IDEAS 4.0 software.

CRISPR off-targets analysis, RNA-seq, TRAP samples preparation and data analysis, behavioral assays, immunohistochemistry, western blotting, ATAC-seq libraries preparations and analysis, live-cell imaging, immuno-electron microscopy, AAV preparation and stereotaxic delivery, electrophysiology, co-immunoprecipitation, mass spectrometry, synaptosomes preparation, and statistical analysis were performed as described in the Extended Data Supplementary Methods section.

## Results

### Deletion of SMARCA3 in CCK cells results in impaired emotional behavior

**Fig. 1:**
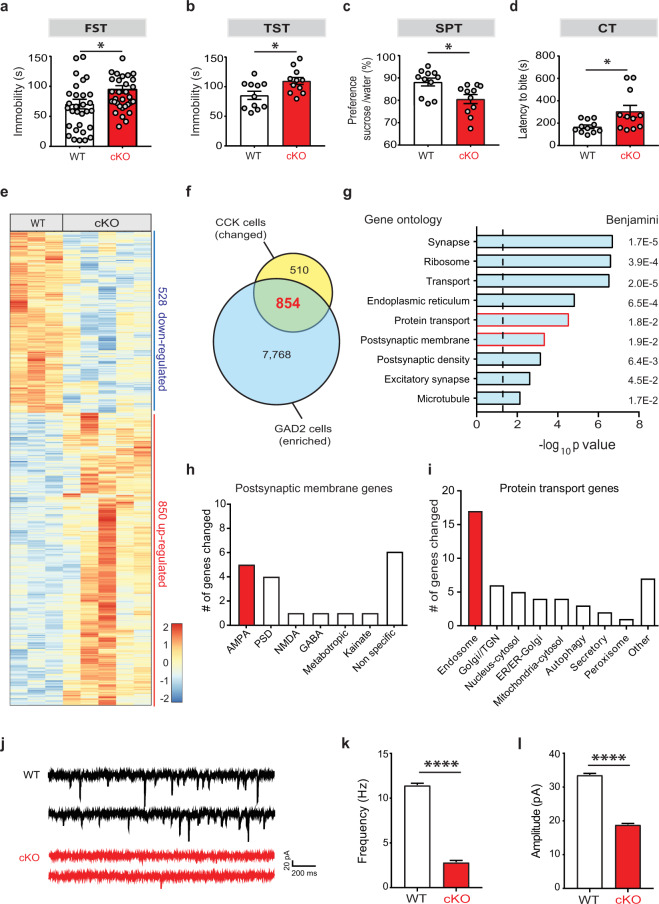
Deletion of SMARCA3 in CCK cells results in impaired AMPA receptor signaling and depressive-like behavior. **a**–**d** WT mice or those with SMARCA3 deletion in CCK cells (cKO) were tested behaviorally: (**a**) FST, forced swim test, unpaired *t* test, *p = 0.016, *n* = 30, 32. **b** TST, tail suspension test, unpaired *t* test, *p = 0.013, *n* = 11, 11. **c** SPT sucrose preference test, unpaired *t* test, *p = 0.010, *n* = 11, 11. **d** CT, cookie test, unpaired *t* test, *p = 0.022, *n* = 11, 11. **e** Heat map representation of differentially expressed genes between the translatomes of hippocampal CCK cells from WT (*n* = 3 replicates from 6 mice) and from cKO mice (*n* = 5 replicates from 10 mice). **f** Venn diagram summary of genes which are both differentially expressed in cKO-CCK cells (yellow) and enriched in GABAergic interneurons of the hippocampus (blue). 854 overlapping genes (in red) were used for the functional annotation analysis. **g** Ontology analysis for the genes regulated by SMARCA3 in CCK cells. Red bar lines highlight the protein transport and postsynaptic membrane ontologies. Dashed line represents significance threshold. **h** Bar graph summary of the postsynaptic membrane-related genes changed in cKO. **i** Bar graph summary of the protein transport-related genes changed in cKO. TGN trans-Golgi network. **j** Representative traces of AMPA miniature postsynaptic currents (mPSCs), as measured in CCK neurons in the DG subgranular zone (SGZ) of WT and cKO mice. **k**–**l** Bar graphs of AMPA mPSCs frequency (**k**) and amplitude (**l**) in SGZ CCK neurons from WT (*n* = 4824 events, ten cells, five mice) and cKO mice (*n* = 849, 10, 4). Kolmogorov–Smirnov test, ****p < 0.0001.

To understand the role of SMARCA3 in hippocampal function, we first analyzed its expression level in different hippocampal cell types. Using the Translating Ribosome Affinity Purification (TRAP) method, we found that both CCK and PV interneurons are highly enriched in SMARCA3 (Fig. S1a). To elucidate if SMARCA3 in CCK- or PV-expressing interneurons is involved in emotional behavior, we generated mice with conditional deletion of its encoding gene, *Hltf* [13] in CCK cells (cKO-CCK) or in PV cells (cKO-PV), and subjected them and their WT littermates to behavioral tests. We observed a robust despair-like behavior in the forced swim test and in the tail suspension test (TST, Fig. 1b) in cKO-CCK mice (Fig. 1a), but not in cKO-PV mice (Fig. S1b, c), suggesting that SMARCA3 in CCK cells promotes antidepressive-like behavior. We further validated this observation using a battery of emotional-behavior tests. Deletion of SMARCA3 from CCK cells resulted in anhedonia-like behaviors in the sucrose preference test (SPT, Fig. 1c) and in the cookie test (CT, Fig. 1d). Additionally, using the elevated plus maze (EPM, Fig. S1d) and the open field tests (Fig. S1e,) we identified anxiety-like behavior in these mice, with no change in locomotion (Fig. S1f).

### SMARCA3 mediates AMPAR signaling in CCK cells

In order to identify the genes regulated by the chromatin remodeling factor in hippocampal CCK cells, we used the TRAP technology (Fig. S1g). CCK^*TRAP*^ mice were used to profile the translatome of hippocampal CCK cells, by isolating the mRNA that is bound to GFP-tagged ribosomes from these cells. First, we validated the CCK^*TRAP*^ mice. As previously shown, GFP expressing cells were found in the hilus and subgranular zone (SGZ) of the DG, (Fig. S1h) [9]. To identify genes that are regulated by SMARCA3 in CCK cells, we generated both WT-CCK^*TRAP*^ and CCK^*TRAP*^ SMARCA3-cKO (Fig. S1g). As expected, the expression level of the *Cck* transcript was enriched in the translatome isolated from hippocampal CCK cells (Fig. S1i). We confirmed a specific deletion of SMARCA3 in CCK cells as the SMARCA3 transcript was down-regulated in the TRAP preparation from the hippocampus of CCK^*TRAP*^ SMARCA3-cKO mice (Fig. S1j), but not in the bulk preparation from that tissue (Fig. S1k).

We then utilized RNA-seq analysis and identified 1,378 differentially expressed genes (DEGs) in these cells following the deletion of SMARCA3 (Fig. 1e, Table S1). To identify the molecular pathways that are mediated by SMARCA3 in inhibitory interneurons, we analyzed the ontologies of the 854 DEGs that were also enriched in hippocampal glutamate decarboxylase (GAD2) expressing cells (Fig. 1f, Table S2). Pathway-analysis identified changes in genes associated with synapse-related genes, specifically genes associated with excitatory synapses and postsynaptic density (Fig. 1g). Inspection of the postsynaptic membrane-related DEGs revealed that 26% of them were associated with AMPAR signaling (Fig. 1h, Table S3). Protein transport-related genes were also altered in CCK cells (Fig. 1g, Table S4). Among these, ~40% were related to endosomal transport (Fig. 1i, Table S4), suggesting that SMARCA3 regulates an endocytic program that, in-turn, regulates AMPAR signaling in these cells.

To identify a possible physiological change in AMPAR signaling in CCK expressing interneurons from cKO-CCK mice, we recorded AMPAR miniature postsynaptic currents (mPSC) from GFP+ cells located at the SGZ. Analysis revealed that SMARCA3 deletion resulted in a dramatic 75% decrease in AMPA mPSC frequency and a 50% decrease in the corresponding amplitudes in CCK cells (Figs. 1j–l, S1l, m), without changes in decay time (Fig. S1n, o). These results suggest that in DG CCK expressing interneurons, SMARCA3 regulates AMPAR signaling.

### Neurensin-2 is repressed by SMARCA3 in interneurons

**Fig. 2:**
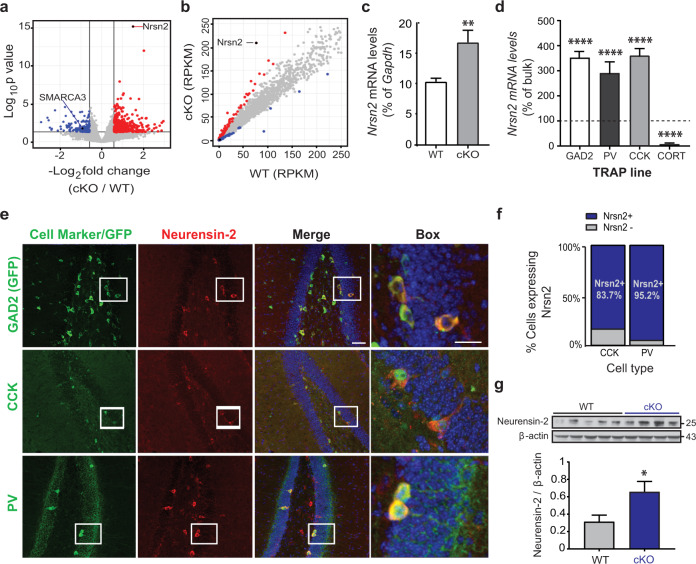
Neurensin-2 expression is repressed by SMARCA3 in CCK cells. **a** Volcano plot analysis of the translatome of CCK hippocampal cells from cKO mice. Red dots and blue dots indicate significantly increased and decreased genes in cKO mice, respectively, compared to WT. Lines indicate significance thresholds. *Nrsn2* is the most significantly changed gene (FDR = 1 × 10^−11^), SMARCA3 is significantly decreased in cKO mice. **b** Scatter plot of translated gene levels (in RPKM) in CCK cells from WT (*X* axis) and cKO (*Y* axis) mice. Color coded dots represent DEG. **c** qPCR analysis of *Nrsn2* translated mRNA levels in CCK cells. Translated mRNA from CCK hippocampal cells was isolated from WT and cKO mice (Unpaired *t* test, **p = 0.008, *n* = 9, 10 mice). **d** Bar graph showing *Nrsn2* translated mRNA levels in four types of hippocampal inhibitory cells that express GAD65 (GAD2), parvalbumin (PV), CCK, or cortistatin (CORT). Data are presented as percentages of *Nrsn2* levels in bulk hippocampal mRNA (100%, dashed line). One-way ANOVA. ****p < 0.0001 vs. bulk, *n* = 8, 6, 10, and 2 replicates from 8, 24, 20, and 8 mice, respectively. **e** Representative immunohistochemical images showing localization of Neurensin-2 in DG interneurons. Blue, DAPI. Scale bars: Merge, 50 μm; box, 20 μm. **f** Quantification of Neurensin-2 expression in CCK and parvalbumin (PV) cells in the SGZ. (CCK, *n* = 37 cells, 18 slices, 4 mice; PV, *n* = 41, 10, 4). **g** Immunoblot scan (top) and quantification (bottom) of Neurensin-2 in hippocampi from WT and from mice with SMARCA3 deletion in CCK cells (cKO). Unpaired *t* test, *p = 0.036, *n* = 4–5/group. Molecular weights are presented in KDa.

We then aimed to elucidate a molecular mechanism by which SMARCA3 regulates AMPAR signaling and behavior, by identifying a downstream gene that mediates these effects. Among all DEGs, the most notable change was in the level of *Nrsn2* (*q* value = 1.01E−11; Fig. 2a, b, Table S1). This gene encodes Neurensin-2, a neuronal-specific vesicular protein, with a suggested role in maintenance and transport of vesicles [19]. qPCR analysis confirmed the upregulation of *Nrsn2* levels in CCK cells from cKO, suggesting that SMARCA3 may mediate the transcriptional repression of *Nrsn2* (Fig. 2c). Using additional TRAP mouse lines, we found that *Nrsn2* expression in the hippocampus is highly and selectively enriched in subpopulations of GABAergic neurons including CCK- and PV positive cells, but not in those that express cortistatin, which also co-express somatostatin [8] (Fig. 2d). Immunohistochemical analysis of the hippocampal DG confirmed the selective expression of Neurensin-2 in these two interneuron populations (Figs. 2e,  S2a), and quantification of the immunopositive cells confirmed that Neurensin-2 is highly expressed in the vast majority of CCK cells and in nearly all PV neurons in the SGZ (Fig. 2f). Neurensin-2 was also highly expressed in a small subset of GABAergic interneurons proximate to the pyramidal cells throughout the hippocampus, as well as in all cerebellar GABAergic Purkinje cells (Fig. S2b). We then measured Neurensin-2 protein levels in hippocampal lysates. Western blot analysis confirmed the induction of the Neurensin-2 protein level in cKO mice (Fig. 2g). This repression of Neurensin-2 by SMARCA3 seems to be specific, since a similar effect was not observed in the levels of Neurensin-1 (Fig. S2c).

### The SMARCA3-Neurensin-2-AMPAR pathway dynamically responds to chronic stress

**Fig. 3:**
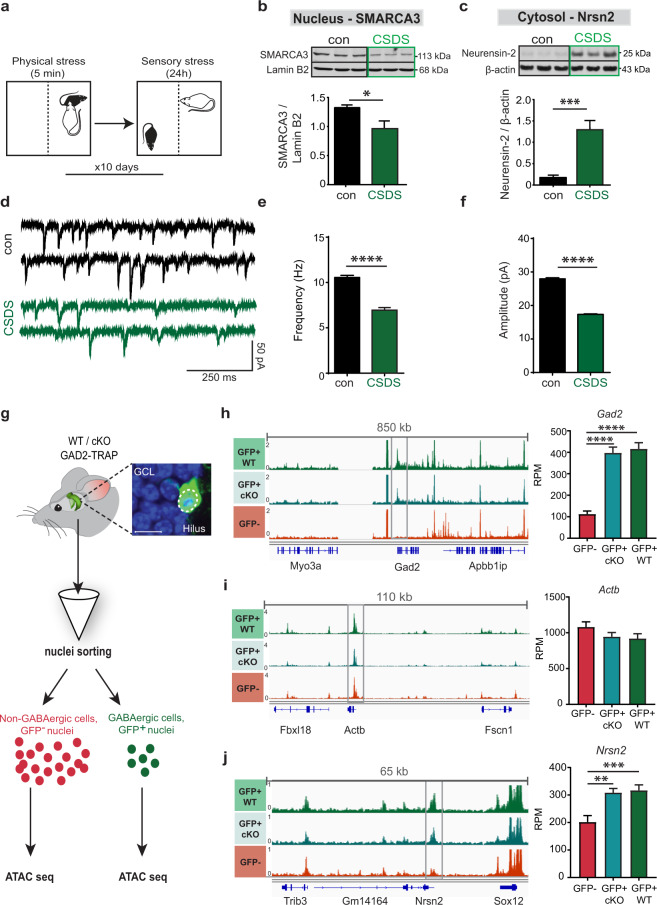
Neurensin-2 expression is dynamically regulated by stress. **a** Schematic representation of the chronic social defeat stress (CSDS) paradigm. **b**, **c** Western blot scans (top) and quantification (bottom) of hippocampal nuclear SMARCA3 (**b**) and cytosolic Neurensin-2 (**c**) following CSDS. Unpaired *t* test, *p = 0.017, ***p = 0.0055. *n* = 5–6/group. **d** Representative traces of AMPA-mediated currents in DG SGZ CCK neurons from control and CSDS susceptible mice. **e**, **f** Bar graphs of AMPA mPSCs frequency (**e**) and amplitude (**f**) in SGZ CCK neurons from control (*n* = 4943 events, 15 cells, 5 mice) and CSDS susceptible mice (*n* = 2522, 16, 5). Kolmogorov–Smirnov test, ****p < 0.0001. **g** Schematic of ATAC-seq analysis in GABAergic cells. Hippocampal cell nuclei from GAD2^TRAP^ mice (WT/cKO-GAD2) were sorted to GFP positive (GABAergic) or GFP negative (non-GABAergic) pools and subjected to ATAC-seq. The image shows a SGZ GABAergic cell with GFP expression in the cell nucleus (dashed circle). GCL, granule cell layer; Scale bar, 10 μm. **h**–**j** Visualization of the normalized overlay of all ATAC-seq tracks at three gene loci: *Gad2* (**h**), *Actb* (**i**) and *Nrsn2* (**j**). Bar graphs (right) show quantification of promoter accessibility (gray frame) in GABAergic cells nuclei (GFP+) from WT, cKO and in non-GABAergic (GFP−) cells. One-way ANOVA; **p = 0.003; ***p = 0.0009; ****p < 0.0001. *n* = 7, 8, 9 replicates pooled from 21, 24, and 27 mice, respectively.

It was previously proposed that the activity of SMARCA3 in the cell nucleus is dynamic and is enhanced following SSRIs [13]. Since SMARCA3 deletion in CCK cells resulted in depressive-like behavior, it is likely that SMARCA3 activity is dynamically suppressed in the depressive state. In order to understand if SMARCA3-mediated repression of Neurensin-2 in the adult hippocampus is attenuated by chronic stress, we measured the changes in their subcellular levels in hippocampal lysates. For that, we utilized the chronic social defeat stress paradigm, a well-established mouse model of depression (CSDS, Fig. 3a) [20]. We observed that chronic stress resulted in reduced SMARCA3 levels in the hippocampal cell nuclei (Fig. 3b). Examination of Neurensin-2 levels in the corresponding cytoplasm revealed that it was significantly induced in stressed mice (Fig. 3c), supporting the idea that the nuclear activity of SMARCA3 as a repressor of *Nrsn2* is attenuated by stress-induced depression. Notably, stress-resilient mice did not show differences in cytosolic Neurensin-2 or nuclear SMARCA3 levels relative to controls (Fig. S3a–d), suggesting that activation of the SMARCA3-Neurensin-2 pathway confers resilience to stress. Moreover, in stress-sensitive mice, Neurensin-2 protein levels were induced in other stress-related brain regions including the prefrontal cortex, amygdala and the nucleus accumbens (Fig. S3e–g). To explore the possibility that chronic stress results in similar physiological deficit as that seen in cKO− mice, we recorded AMPAR-mediated currents in SGZ CCK neurons from stress-sensitive mice. Indeed, robust reduction was found in both frequency and amplitude of AMPAR mPSCs in cells from stress-susceptible animals (Fig. 3d–f). Together, these results demonstrate that both the genetic and behavioral models of depression are associated with impairment in AMPAR signaling in CCK cells. These impairments coincided with a dynamic upregulation of Neurensin-2 levels in the depressive state.

### SMARCA3 indirectly represses Neurensin-2 in interneurons

An adaptive, stress responsive, and chromatin remodeling activity was previously reported in the nucleus accumbens. The ACF complex was dynamically upregulated after chronic stress, leading to increased repression of gene transcription [21]. To examine the possibility that SMARCA3 represses the Nrsn2 gene by direct chromatin remodeling, we devised an ATAC-seq assay in GABAergic hippocampal cells. We took advantage of the fact that GFP-tagged ribosomes in TRAP mice were also found inside the cell nucleus (Figs. 3g, S4a, b). Cell nuclei from fresh hippocampi of GAD2^*TRAP*^ or SMARCA3 cKO-GAD2^*TRAP*^ mice were sorted and their chromatin was subjected to transposase reaction followed by sequencing. The nuclear purity and integrity of GFP+ nuclei, as well as the transposase activity, were validated (Fig. S4b–e). Increased accessibility of the *Gad2* gene promoter in GABAergic cells confirmed the fidelity and specificity of this approach (Fig. 3h, i). The accessibility of the Nrsn2 gene in GABAergic cells was not altered in the absence of SMARCA3, suggesting that SMARCA3 activity represses Nrsn2 expression levels without affecting the accessibility of its gene (Fig. 3j). Nonetheless, the accessibility of Nrsn2 promoter in GABAergic neurons was increased relative to that from non-GABAergic cells (Fig. 3j), suggesting that chromatin modulation plays a major role in its cell-type-specific expression and regulation.

### Neurensin-2 level regulates depressive behaviors

**Fig. 4:**
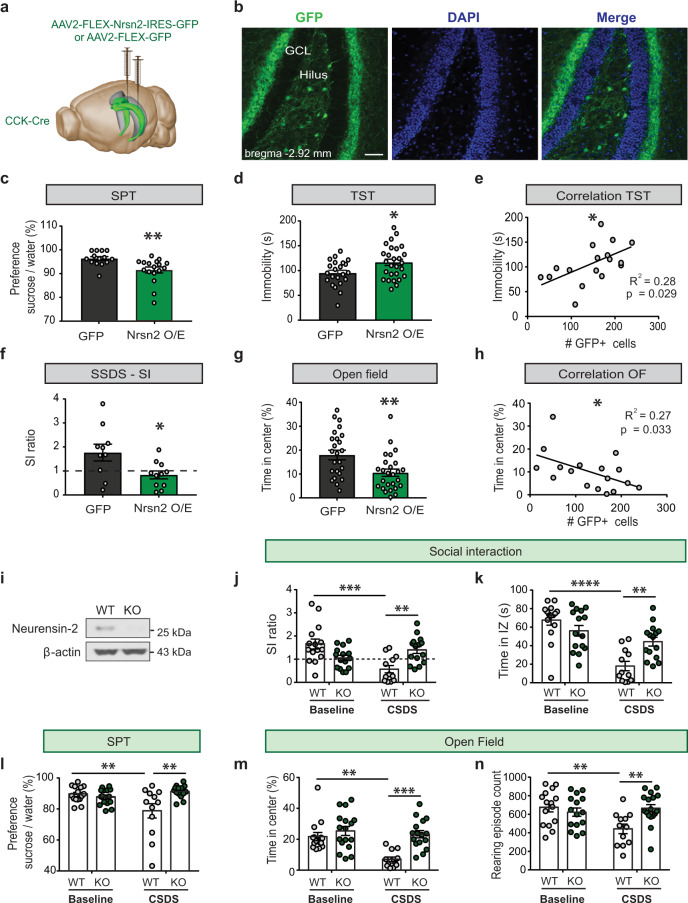
Neurensin-2 dynamics regulate emotional behaviors. **a** Schematic of the strategy to up regulate Neurensin-2 in DG CCK cells. AAVs were injected bilaterally to the DG (in green) of CCK-Cre mice. **b** Representative images showing GFP expressing cells in the DG, 21 days after AAV2-FLEX-GFP injection. Scale bar, 50 μm. **c** SPT. Unpaired *t* test, **p = 0.005, *n* = 14, 17. **d** TST. Unpaired *t* test, *p = 0.016, *n* = 22, 27. **e** Correlation between the number of cells expressing GFP in Nrsn2 O/E mice and the corresponding immobility in the TST (Pearson, *n* = 17). **f** Social interaction (SI) test following subthreshold social defeat stress (SSDS). Unpaired *t* test, *p = 0.023, *n* = 10, 11. Dashed line represents stress resilience threshold. **g** OF. Unpaired *t* test, **p = 0.005, *n* = 24, 27. **h** Correlation between the number of transfected cells and the time spent in the center of the OF arena. (Pearson, *n* = 17). **i** Neurensin-2 protein analysis from hippocampi of a WT mouse and a Neurensin-2 KO mouse (KO). **j**–**n** WT or Neurensin-2 KO mice were stress-naïve (baseline) or subjected to CSDS, followed by behavioral tests. **j** Ratio of time in interaction zone in the social interaction (SI) test. Two-way ANOVA, interaction, *F* (1, 53) = 18.63, *P* < 0.0001; stress, *P* = 0.030; genotype, *P* = 0.495; **p = 0.006, ***p = 0.0002. *n* = 12–15/group. **k** Time in interaction zone (IZ) in the SI test. Two-way ANOVA, interaction, *F* (1, 53) = 12.63, *P* = 0.0008; stress, *P* < 0.0001; genotype, *P* = 0.172. **p = 0.007, ****p < 0.0001. *n* = 12–15/group. **l** SPT. Two-way ANOVA, interaction, *F* (1, 54) = 10.51, *P* = 0.002; stress, *P* = 0.125; genotype, *P* = 0.017. **p = 0.0097 for stress effect, **p = 0.0013 for genotype effect. *n* = 12–16/group. **m** Time in the center of an open field arena. Two-way ANOVA, interaction, *F* (1, 54) = 6.187, *P* = 0.016; stress, *P* = 0.0022; genotype, *P* = 0.0004. **p = 0.0016, ***p = 0.0003. *n* = 12–16/group. **n** Number of rearing episodes during the open field test. Two-way ANOVA, interaction, *F* (1, 54) = 0.0013; *P* = 11.54; stress, *P* = 0.071; genotype, *P* = 0.030. **p = 0.0041 for stress effect, **p = 0.0015 for genotype effect. *n* = 12–16/group.

To identify causality between the dynamic upregulation of Neurensin-2 in CCK cells and the depressive-like behavior seen in stressed mice or in cKO mice, we used viral-mediated gene delivery to overexpress Neurensin-2 in DG CCK cells (Figs. 4a, b,  S5a, b). Neurensin-2 overexpression in the adult hippocampus resulted in reduced preference to sucrose as compared to that in mice transfected with the control-GFP vector (Fig. 4c). Similarly, induction of Neurensin-2, but not of GFP, resulted in increased immobility in the TST (Fig. 4d, e). We then evaluated the vulnerability of these mice to depression using a subthreshold social defeat stress paradigm (SSDS, Fig. S5c) [20]. As expected, 80% of the control mice showed social interaction (SI) ratio greater than 1. In contrast, 73% of the Neurensin-2 overexpressing mice showed SI ratio below 1, an indication of vulnerability to depression (Fig. 4f). In addition to anhedonic and despair-like behaviors, Neurensin-2 overexpression resulted in anxiety-like behavior in the open field test, without a deficit in locomotion (Figs. 4g, h,  S5d). We also observed an impairment in nesting behavior of Neurensin-2 overexpressing mice (Fig. S5e, f), an indication of compromised well-being, which is known to be induced by stress models of depression [22, 23]. These data imply that elevated Neurensin-2 levels in DG CCK cells is sufficient to cause depressive- and anxiety-like behaviors in the adult mouse.

Then we studied whether downregulation of Neurensin-2 might render mice resilient in response to chronic stress. We generated Neurensin-2 constitutive KO mice using the CRISPR-Cas9 technology (Figs. 4i,  S6a, Table S5), and tested their sociability following CSDS. Strikingly, defeated Neurensin-2 KO mice showed less social avoidance (Fig. 4j, k), anhedonia (Fig. 4l), or anxiety-like behavior (Figs. 4m, n,  S6b, c) following the stress, relative to WT controls. Interestingly, the KO mice did not show basal behavioral alterations in these tests. Taken together, these data indicate that deletion of Neurensin-2 confers resilience to chronic stress, whereas its induction in CCK cells results in depressive-like behaviors.

### Neurensin-2 binds postsynaptic proteins and regulates AMPAR signaling

**Fig. 5:**
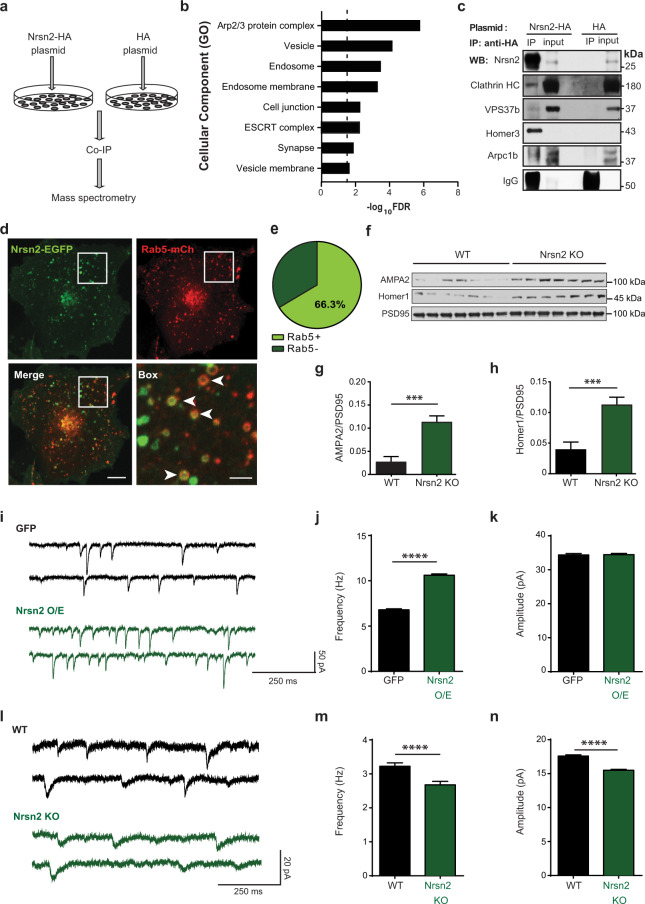
Neurensin-2 associates with endosomes and postsynaptic proteins. **a** Schematic representation of co-immunoprecipitation (co-IP) experiment. N2a cells were transfected with HA-tagged Neurensin-2 (Nrsn2-HA) or HA control (HA). **b** Cellular component annotations for 166 suggested Neurensin-2 binding partners. Dashed line represents significance threshold. **c** Immunoblot scans of the Neurensin-2 binding partners after co-IP of HA-Nrsn2 or HA control and the corresponding 1% inputs in N2a cells transfected with HA-Nrsn2 or HA control. HC heavy chain, VPS37b vacuolar protein sorting-associated protein 37B, Arpcb1 actin related protein 2/3 complex subunit 1b. **d**, **e** Representative images of live-cell imaging (**d**) and Pie chart summary (**e**) showing co-localization of EGFP-tagged Nrsn2 protein with mCherry-tagged Rab5 (Rab5-mCh) in Cos7 cells. Arrowheads indicate co-labeled vesicle-like structures. Scale bars: Merge, 5 μm; box, 1 μm. *n* = 104 vesicles, 12 cells. **f**–**h** Western blot scans (**f**) and quantification (**g**, **h**) of synaptosomal protein isolated from WT or Neurensin-2 KO mice (Nrsn2 KO). **g** AMPA2 unpaired *t* test, ***p = 0.0003, *n* = 7/group. **h** Homer1 unpaired *t* test, ***p = 0.0006. **i** Representative traces of AMPA-mediated currents in DG SGZ CCK neurons from GFP and Nrsn2 O/E mice. **j**, **k** Bar graphs of AMPA mPSCs frequency (**j**) and amplitude (**k**) in SGZ CCK neurons from GFP (*n* = 2942 events, 14 cells, 3 mice) and Nrsn2 O/E mice (*n* = 3477, 14, 3). Kolmogorov–Smirnov test, ****p < 0.0001. **l** Representative traces of AMPA-mediated currents in cerebellar Purkinje neurons from WT and Nrsn2 KO mice. **m**, **n** Bar graphs of AMPA mPSCs frequency (**m**) and amplitude (**n**) in cerebellar Purkinje neurons from WT (*n* = 1270 events, 14 cells, 3 mice) and Nrsn2 KO mice (*n* = 1105, 14, 4). Kolmogorov–Smirnov test, ****p < 0.0001.

To identify a possible role for Neurensin-2 in regulating neuronal function, we first characterized its binding partners and subcellular localization. We used an in vitro approach by transfecting N2a cells with HA-tagged Neurensin-2 and co-immunoprecipitating the tagged protein (Fig. 5a). Mass spectrometry analysis identified 166 proteins as putative binding partners for Neurensin-2. The strongest observed association of Neurensin-2 was with two members of the Homer scaffold proteins, namely Homer1 and Homer3 (Table S6). Functional annotation of all putative binding partners indicated that Neurensin-2 is incorporated into endocytic vesicles and interacts with postsynaptic-related proteins (Fig. 5b). We then used co-immunoprecipitation to validate the protein–protein interaction between Neurensin-2 and two endocytosis-related proteins, clathrin heavy chain and the Endosomal Sorting Complexes Required for Transport-related protein, VPS37b (Fig. 5c). Furthermore, we also confirmed a direct binding of Neurensin-2 to the postsynaptic scaffold protein, Homer3, and to the actin related protein 2/3 complex subunit 1b (Arpc1b, Fig. 5c), another postsynaptic-related protein [24]. Live-cell imaging in non-neuronal Cos7 cells as well as in neuronal N2a cells showed co-localization of EGFP-tagged Neurensin-2 with Rab5 positive vesicles, supporting a role for Neurensin-2 in the endocytosis of postsynaptic proteins (Figs. 5d, e, S7a, b). Immuno-electron microscopy demonstrated the proximity of EGFP-Neurensin-2 to clathrin (Fig. S7c), supporting the idea that Neurensin-2 plays a role in endocytosis.

We then studied if Neurensin-2 is implicated in subcellular localization of postsynaptic AMPAR in the hippocampus. We measured the level of the AMPA2 subunit and the scaffold protein that regulates its endocytic cycle, Homer1 [25], in hippocampal synaptosomes. We observed induction in the levels of both proteins in synaptosomal preparations from Neurensin-2 KO mice, compared to those from WT mice (Fig. 5f–h), further supporting a link between the SMARCA3/Neurensin-2 pathway and AMPAR shuttling.

Finally, to confirm a direct role for Neurensin-2 in the regulation of AMPAR signaling in GABAergic cells, we recorded AMPA mPSCs from SGZ CCK interneurons following Neurensin-2 overexpression in these neurons. We found that the frequency of AMPAR-mediated currents was increased when Neurensin-2 was overexpressed in CCK cells (Fig. 5i–k). In a different set of experiments, AMPAR-mediated currents were attenuated in Purkinje cells from Neurensin-2 KO mice. Deletion of Neurensin-2 led to a reduction in both frequency and amplitude of AMPAR-mediated currents when compared to WT mice (Fig. 5l–n). Together, these data strongly implicate Neurensin-2 in the regulation of AMPAR signaling in GABAergic neurons.

## Discussion

In this study we identified a new emotion-regulating protein, Neurensin-2. In the hippocampus, Neurensin-2 is selectively expressed in subpopulations of GABAergic interneurons where its expression level is dynamically repressed by the activity of SMARCA3. Neurensin-2 is a major modulator of the excitatory synapse and is incorporated into early endosomes, where it interacts with synaptic proteins.

### SMARCA3 in interneurons mediates emotional behavior and AMPAR signaling

Deletion of SMARCA3 in CCK cells resulted in basal depression-like and anxiety-like behaviors. Such phenotype imply that both SMARCA3 and CCK interneurons mediate the basal emotional state [13]. A role for CCK cells in depression and anxiety was suggested by recent studies which applied chemogenetic manipulation of hippocampal CCK cells. Two independent studies showed that CCK cell inhibition leads to antidepressive like behavior [9], and their activation to anxiolytic-like behavior [26]. Our current data indicates that in CCK cells, SMARCA3 mediates cell-type specific genes which modulate emotional behavior. Indeed, deletion of SMARCA3 resulted in gene-expression changes in the adult brain. Very little is known about the mechanisms by which SMARCA3 regulates gene-expression in vivo. Its best established cellular role is mediating DNA repair and genome-stability maintenance, which is extensively studied in dividing cells in vitro in the context of cancer [14, 27, 28]. In the neonate brain, SMARCA3 was suggested to regulate the expression of cell-cycle genes [29]. Here we show that in the adult brain, SMARCA3 regulates genes that are implicated mostly in cellular functions. Cell-type specific RNA-seq revealed that SMARCA3 is involved in the expression of ~1400 genes in CCK cells. Many of these genes were clustered to transport via the endosome, indicating that SMARCA3 mediates endocytosis program in these cells. Additionally, many genes regulated by SMARCA3 were annotated to the postsynaptic membrane cellular component, specifically to the function of AMPAR signaling. This finding has a profound physiological significance in these cells since excitatory postsynaptic terminals at hippocampal interneurons predominantly contain AMPARs [10]. Indeed, SMARCA3 cKO mice had a dramatic decrease in AMPAR signaling in CCK interneurons, a similar effect to the one we observed in social defeat stress model. In line with our data, a recent study reported that the deletion of AMPAR subunits from caudal ganglionic eminence-derived interneurons resulted in anxiety-like behavior [30]. The glutamatergic signaling in interneurons becomes increasingly associated with depressive disorders and their treatments [31, 32]. In the prefrontal cortex, glutamatergic signaling in PV and somatostatin interneurons mediates the rapid antidepressant action of ketamine, an effect which is mediated by NMDA receptor inhibition [31]. Our current data strongly indicate that in the hippocampus, depression-like behavior is associated with altered AMPAR signaling in CCK interneurons. We suggest that this alteration is molecularly regulated by SMARCA3.

### *Nrsn2* expression is regulated by SMARCA3 in hippocampal interneurons

Examination of the specific genes altered by SMARCA3 deletion in CCK cells revealed a remarkable upregulation in the expression levels of the *Nrsn2* gene. This gene encodes Neurensin-2, a neuronal-specific vesicular protein with a possible role in protein transport [19]. The precise mechanism by which SMARCA3 deletion leads to *Nrsn2* induction is not completely understood. Our ATAC-seq results indicate that in GABAergic hippocampal cells, the global accessibility of the Nrsn2 gene was not targeted by SMARCA3. This suggests that in the adult brain, SMARCA3 mediates gene repression in a different mechanism than the ACF chromatin remodeling complex. In the adult nucleus accumbens, ACF induces gene repression by altering nucleosome positioning to mediate depressive-like phenotype [21]. Nevertheless, we find that the promoter of *Nrsn2* is specifically accessible in GABAergic neurons, indicating that its cell-type specific expression involves cell-type specific chromatin remodeling. It is possible that the repression of Nrsn2 gene expression by SMARCA3 is mediated by other mechanisms, for example, by long-distance chromatin looping, which depends on both ATP and additional binding proteins. Such SMARCA3-mediated looping promotes the suppression of the *Prl* gene-expression [33]. Additionally, SMARCA3 was also shown to act as a transcription factor by directly binding specific promoters to induce gene transcription. Two motifs were suggested for this specific function of SMARCA3, (A/G)G(T/C)(G/T)G, and (C/A)C(T/A)TN(T/G) [14]. Promoter analysis of both murine and human Nrsn2 genes predicts SMARCA3 binding sites, which opens the possibility that SMARCA3 directly binds to the Nrsn2 gene to promote repression. While such direct repression was not documented for SMARCA3, it was observed with other SWI/SNF family member, Snf2. This chromatin remodeler directly binds the SER3 gene*-*promoter to induce repression in yeast [34]. Hence, we cannot exclude a direct repression mechanism for SMARCA3 at the *Nrsn2* promoter. Finally, it is possible that SMARCA3 directly modulates the chromatin accessibility of another regulatory factor that in turn, regulates *Nrsn2* gene expression.

### Neurensin-2 directly modulates emotional behavior

The subcellular localization of both SMARCA3 and Neurensin-2 were dynamically regulated by the emotional state. After chronic stress, the levels of SMARCA3 in the cell nucleus were reduced, suggesting that the intracellular localization of SMARCA3 is dynamically regulated by the emotional state. Additionally, chronic stress induced the expression levels of Neurensin-2, further supporting the idea that attenuated SMARCA3 activity leads to Neurensin-2 upregulation. Manipulation of Neurensin-2 levels resulted in robust changes in depressive-like behaviors. Overexpression of Neurensin-2 in DG CCK cells led to depression-like and anxiety-like behaviors, a similar phenotype to that observed in the SMARCA3 cKO mice. In both models, Neurensin-2 is upregulated in hippocampal CCK cells, suggesting that emotional deficits are caused by such Neurensin-2 upregulation. Moreover, Neurensin-2 KO mice are highly resilient to social defeat stress, with no basal behavioral differences. Together, these observations further indicate that the dynamic upregulation of Neurensin-2 plays a key role in facilitating emotional behavior deficits.

### Neurensin-2 as a potential mediator of AMPAR shuttling in interneurons

We showed that SMARCA3 is crucial for AMPAR function in CCK cells as well as for the expression of endocytosis-related genes, and we propose that Neurensin-2 is a part of a cellular endocytic program which modulates AMPAR signaling in these cells and is regulated by SMARCA3. The endocytic machinery is highly plastic and may be regulated according to cellular demands. Such regulation includes different transcriptional programs controlling endocytosis [35]. In a recent study, we elucidated the involvement of p11, a SMARCA3-activity regulator protein, in modulating the endocytosis of potassium channel Kv3.1 in DG interneurons [36], suggesting that p11-SMARCA3 signaling mediates an endocytic program to regulate the activity of DG interneurons. Collectively, these findings support the idea that the p11-SMARCA3-Neurensin-2 signaling dynamically regulates a cellular endocytosis program in DG interneurons to mediate their function, AMPAR signaling, and consequently, to modulate emotional behavior. In order to understand which cellular function is regulated by Neurensin-2, we studied its cellular expression and localization as well as the proteins it interacts with. Using TRAP and immunohistochemistry, we found that Neurensin-2 is expressed predominantly in GABAergic cells, at least in the hippocampus and in the cerebellum. Specifically, we found that the vast majority of CCK and PV interneurons in the DG express Neurensin-2. In the cerebellum, Neurensin-2 is highly expressed in the Purkinje cells, another population of GABAergic cells which are also immunopositive to PV. The highly specific pattern of Neurensin-2 expression indicates that this protein is involved in a cell-type specific regulation of cellular function.

We found that Neurensin-2 is localized in vesicular structure corresponding to the endosome and binds endocytic and postsynaptic density-related proteins. The most profound binding of Neurensin-2 was with two members of the Homer scaffold proteins. Association with both endocytosis-related proteins and with Homers, strongly suggests that Neurensin-2 is involved in the endocytosis-cycle of glutamatergic receptors including AMPARs. In hippocampal neurons, Homer physically couples the postsynaptic density with the endocytic machinery to maintain and regulate local AMPAR recycling within the dendrite [25]. Neurensin-2 also associates with the Arp2/3-complex, an AMPAR endocytosis regulator protein [37], further supporting a role for Neurensin-2 in the regulation of AMPAR endocytosis. Intriguingly, when Neurensin-2 is deleted, both Homer1 and AMPA2 are induced at the synapses, suggesting that the endocytosis of this subunit is impaired. However, it is important to note that these preparations are extracted from whole hippocampal lysates, where interneurons are significantly underrepresented. Therefore, it is likely that this reflects a secondary effect in the principle cells and an adaptive response to circuit-level changes following deletion on Neurenisn-2 in local interneurons.

Our physiological data from Neurensin-2 KO and overexpressing mice indicate that Neurensin-2 is involved in AMPAR signaling. Neurensin-2 KO mice showed reduced AMPAR signaling whereas Neurensin-2 overexpression in CCK cells led to increased AMPAR-mediated currents. Notably, socially defeated mice showed a profound impairment in AMPAR-mediated currents. While impairment in glutamatergic signaling in the DG is highly associated with depression [1], to our knowledge, this is the first report showing that AMPAR signaling is attenuated in DG interneurons in a model of depression. In line with this, SMARCA3 cKO mice showed depressive-like behavior and attenuation of AMPAR-mediated currents. Interestingly, overexpression of Neurensin-2 resulted in depression-like behavior accompanied by enhanced AMPAR signaling. In contrast, deletion of Neurensin-2 resulted in attenuated AMPAR signaling and resilience to stress. These results strongly support the idea that Neurensin-2 regulates AMPAR signaling in a complicated mechanism that is dynamically linked to the emotional state. However, they also associate changes in AMPA signaling in DG neurons with changes in emotional states, and suggest that these changes are both mediated by SMARCA-3 and Neurensin-2. It is also likely that changes in Neurensin-2 levels in inhibitory CCK neurons lead to alterations of the excitatory-inhibitory balance in the entire DG circuitry. Indeed, we found that not only the amplitude of AMPAR-mediated currents was dramatically altered by genetic manipulations of Neurensin-2 in CCK neurons, but also the frequency. Such frequency changes in hippocampal caudal ganglionic eminence- derived neurons were also observed when AMPAR subunits were deleted. Theses frequency changes were also accompanied by anxiety-like behaviors [30]. Therefore, SMARCA3/Neurensin-2 modulation of AMPAR frequency in CCK neurons may mediate impairments in emotional behavior. Moreover, depressed mice also showed dramatic attenuation in both amplitude and frequency of AMPAR-mediated currents, strongly suggesting that the depressive state is associated with massive changes in DG circuitry. Further studies are required to fully understand the mechanisms by which dynamic changes in Neurensin-2 expression modulate AMPAR cellular localization and function in the DG.

In conclusion, here we identified a novel, cell-type specific pathway which dynamically modulates emotional behavior. The identification of Neurensin-2 as an emotion-regulating protein may lead to the development of new antidepressants that will target specifically the interneurons, and hence will be highly specific and tolerable. Since dysfunction of these interneurons is implicated in the pathophysiology of neurological and neuropsychiatric diseases other than depression, including epilepsy [38], schizophrenia, autism [39], and memory dysfunction [40, 41], the SMARCA3/Neurensin-2 pathway is most likely implicated in these pathologies and in their future treatments.

## Supplementary information

Supplemental Materials and Methods

Supplemental Figures

Table S1

Table S2

Table S3

Table S4

Table S5

Table S6
