# Supplementary material for: Identification of Neurensin-2 as a novel modulator of emotional behavior
Source: Mol Psychiatry. 2021 Mar 19;26(7):2872–85. doi: 10.1038/s41380-021-01058-5 (PMC8505262; doi:10.1038/s41380-021-01058-5)
Supplement: Supplementary file 1 — Supplemental Materials and Methods [file 41380_2021_1058_MOESM1_ESM.pdf]

Supplemental Materials and Methods

Generation of Nrsn2 KO mice: off-target examination

A total of 361 off-target sites predicted by CRISPOR software for the Nrsn2-guide used for generating the KO model: 7 sites are on the exons, 12 on the introns, and the rest are on the intergenic region. For actual off-target verification via Sanger sequencing, all 7 exon sites were included, 8 additional candidates (3 intronic; 5 intergenic) were examined for potential offtarget, based on their mis-match (mm) to the used guide. All 15 examined off targets were confirmed negative by PCR and Sanger sequencing (Table S5).

RNA-seq of TRAP samples

4 freshly harvested hippocampi from 2 CCK^{TRAP} mice were pooled for translating ribosome affinity purification (TRAP) sample, and 2 hippocampi from 1 GAD2^{TRAP} mouse were used in each sample for the TRAP experiment. The TRAP procedure was conducted as described [1]. RNA was purified using RNeasy Micro Kit (Qiagen, Hilden, Germany). All RNA samples were validated for high quality and were quantified using Bioanalyzer RNA 6000 Pico Kit (Agilent, San Diego, CA). 1 ng of translating mRNA from each I.P. sample or bulk mRNA was reversed transcribed using Ovation RNA-seq V2 kit (Nugen, San Carlos, CA). 75 bp long, single end labeled cDNA libraries were constructed using Truseq RNA sample preparation kit V2 (Illumina, San Diego, CA) and were sequenced by Illumina NextSeq 500 Sequencer at the Genomics Center at The Rockefeller University. TRAP data for granule cells, CA1, CA2 and CA3 neurons were analyzed from Roussarie et al., 2020 [2].

RNA-seq Biostatistics

For data analysis, fastp [3] with default parameters for the quality control of raw reads was used. Raw reads were mapped to the mouse mm10 genome using STAR [4] with default parameters. The systemPipeR [5] was invoked to build the workflow for calculating reads per kilobase of transcript per million mapped (RPKM) and detecting the differentially expressed genes (DEGs). R package DESeq2 [6] was used for detection of DEGs. For heat map generation, genes with p value < 0.05 and fold-change > 1.2 were used.

Gene Ontology (GO) term enrichment analysis and cluster enrichment were conducted using the DAVID Bioinformatics Resources 6.8 (https://david.ncifcrf.gov/). All filtered clusters had enrichment factor > 1.4. Venn diagrams were generated using the VIB / UGent Bioinformatics & Evolutionary Genomics (http://bioinformatics.psb.ugent.be/webtools/Venn/). Gene expression changes $p < 0.05$ were considered significant. Sequencing data are available at GEO, GSE141481 and GSE141483.

TRAP quantitative PCR (qPCR)

For qPCR analysis of TRAP samples, 1 ng of purified RNA was reverse transcribed using Ovation PicoSL WTA System V2 kit (Nugen), and 20 ng of cDNA was used as a template for analysis. TaqMan Gene Expression Assays and Taqman Universal PCR Master Mix, no AmpErase UNG (Life Technologies, Carlsbad, CA) was used for all analyses. Fluorescence was detected using ABI 7900HT (Applied Biosystems, Foster City, CA). Changes in gene expression were calculated using the ΔΔ Ct formula.

Behavioral assays

Behavioral tests were performed during the light cycle in a designated sound-proof behavioral room by experimenters blinded to the genotype of animals and drug treatments. Adult (9-12 weeks) male mice were used for the experiments. Mice undergoing more than one test were tested in the following order: open field test (OF), elevated plus maze (EPM), novelty suppressed feeding (NSF), cookie test (CT), forced tail suspension test (TST), swimming test (FST), sucrose preference test (SPT) and nesting. One mouse was subjected to no more than 4 behavioral tests. At least 48 hours of interval were allowed between tests. All tested mice were habituated in the test room for at least 1 h prior to the test.

Tail suspension test (TST)

Immobility time was examined as previously described [7]. Mice were suspended by the tail for 6 min and the immobility time was analyzed during the last 4 min by the automated TST analysis software (CleverSys Inc, Reston, VA, USA).

Forced swim test (FST)

Immobility time was examined as previously described [8] . Mice were placed in glass cylinder (16 cm diameter, 50 cm height) filled with water (23−24°C) to a height of 30 cm for 6 min. Immobility time was analyzed during the last 4 min by the automated FST analysis software (CleverSys Inc, Reston, VA, USA).

Sucrose preference test (SPT)

Singly housed mice were habituated to two water bottles for 24 h. The next day, 1 h before the dark period, one random water bottle was replaced with 1.5% sucrose solution. The consumption of water and sucrose solution was measured 12 h later by weighing the bottles. The sucrose preference was calculated as follows: (sucrose weight)/ (sucrose weight +water weight) x 100%.

Cookie test (CT)

Hedonic behavior was examined using the cookie test as previously described [9] .Time to bite the cookie was measured after mice were introduced to cookies for 5 days following 5 days of cookie deprivation.

Open field test (OF)

Time spent at the center of the arena and the locomotor activity were examined as previously described [7]. Mice were allowed to freely explore the open field arena (50 cm x 50 cm x 22.5 cm). Total distance traveled and time spent in the center/periphery of the arena during a 60 min test session was automatically recorded and calculated using the automated Superflex software (Accuscan Instruments, Columbus, OH, USA).

Elevated plus maze (EPM)

Innate anxiety behavior was measured in the EPM. A plus-shaped maze with two open arms and two closed arms was placed approximately 40 cm elevated from the floor. Animals were placed in the center

of the maze facing an open arm, and the time spent in each arm during a 5 min session was recorded and analyzed by the video-tracking Ethnovision 7.0 (Noldus, Wageningen, Netherlands)

Nesting

Nesting behavior was measured after a cotton ball was introduced into the home cage of a singly housed mouse 1 h before the dark period. Scores (range 1-5) were given by an experimenter blinded to groups and treatments, following a previously published scoring protocol [10].

Chronic social defeat stress (CSDS)

The CSDS was carried out as described previously [11]. For 10 consecutive days, the experimental mice were placed for 5-min in the home cage of a previously screened, unfamiliar, aggressive CD-1 mouse. Following this physical stress, the two mice were separated by a perforated divider for the remaining 24 h until the next defeat session. Each day, the experimental mouse was exposed to a different aggressor. At the same time, control mice were placed in pairs within an identical divided cages and were rotated daily between cages with no physical contact with one another.

Sub-threshold social defeat stress (SSDS)

The sub-threshold social defeat was carried out as described previously [11], with minor modifications. Briefly, experimental mice were placed into the home cage of a CD-1 aggressor for a 2-min physical contact. Mice were then allowed to rest without physical contact for 15 min. Defeat episodes were repeated three times. After SSDS, experimental mice were returned to a group housing in their home cages. Social interaction test was performed 24 h after the SSDS.

Social interaction (SI)

24 h after the SSDS or 10 days of CSDS, SI test was performed [11]. SI test was composed of two phases, 2.5 min each, in which the experimental mice were allowed to explore an open field arena (42 cm x 42 cm x42 cm) with a wire mesh (10 cm wide x 6.5 cm deep x 42 cm high) located in a designated place inside

the arena. In the first phase, the wire mesh was empty. In the second phase, a novel CD-1 aggressor mouse was placed inside the wire mesh. The amount of time spent by the experimental mice in the interaction zone (IZ) surrounding the wire mesh was recorded and analyzed by the video-tracking apparatus and software Ethnovision 7.0 (Noldus, Wageningen, the Netherlands). SI ratio was calculated by dividing the amount of time spent by the experimental mice in the IZ in the second phase over the time in the first phase. Susceptible mice were defined by a SI ratio < 1 whereas resilient mice were defined by a SI ratio >1.

Immunohistochemistry

Animals were deeply anesthetized and were slowly perfused transcardially with PBS, followed by 4% paraformaldehyde (PFA) in PBS. For detection and determination of Neurensin-2 in CCK cells, mice were intracerebrovascularly infused with 15 μg of colchicine 24 h prior to the intracardial perfusion to inhibit the transport and secretion of CCK. Brains were post-fixed in 4% PFA for 1 h at 4ºC, and then incubated overnight with 30% sucrose. Then, brains were frozen in the OCT medium over dry ice block and stored at -80°C. Coronal sections of 20 μm were obtained using Leica CM3050 S cryostat and were frozen for later use. Sections were then thawed to room temperature and fixed with 4% PFA for 15 min, followed by 3 PBS washes and 1hour block in 5% normal goat serum. Commercial primary antibodies against Neurensin-2 (mouse monoclonal, Sigma Aldrich, St. Louis, MO, 1:500), PV (Rabbit polyclonal, Abcam, Cambridge, MA, 1:1000) GFP (Rabbit polyclonal, Thermo Fisher Scientific, Waltham, MA, 1:1000) or CCK (rabbit polyclonal, Acris, Rockville, MD, 1:200) were used for overnight incubation. For detection, secondary Alexa goat anti- mouse or goat anti-rabbit were used (Thermo Fisher Scientific, 1:2,000). Finally, slices were incubated with DAPI (Roche, Mannheim, Germany, 1 μg/mL) for 10 min. Images were taken by a LSM710 confocal microscope (Zeiss, Germany). For PV and CCK colocalization with Neurensin-2, 3–5 sections were stained per antibody per mouse. To quantify the number of cells co-expressing Neurensin-2, only cells in the subgranular zone (SGZ) of the DG were selected. The pixel mean value of Neurensin-2 inside a PV or CCK cell was divided by its level outside the cell, using a

custom written Matlab code. A total of 37 CCK+ SGZ cells and 41 PV+ SGZ cells from 4 mice in each group were used for analysis. Cells with Neurensin-2 mean pixel values above 140% of the background were considered immunopositive.

Western blotting

Mice hippocampi were flash frozen and kept in -80°C until use. For whole cell lysates, the hippocampi were added in 10 times excess volume of RIPA lysis buffer (Sigma Aldrich), supplemented with a protease inhibitor cocktail (Complete-EDTAfree; Roche), homogenized and sonicated with probe-type sonicator (Branson, Danbury, CT) for 10 sec twice, followed by centrifugation (5,000 rpm, 10 min). For cytosolic/nuclear fractionation, hippocampi were processed using NE-PER Nuclear and Cytoplasmic Extraction Reagents (Thermo Fisher Scientific). Protein level was determined by BCA protein assay (Thermo Fisher Scientific) and 20-30 μg protein were loaded onto SDS-PAGE followed by a transfer to a nitrocellulose membrane. Proteins were detected using antibodies for Neurensin-2 (mouse monoclonal, Sigma Aldrich, 1:750), β actin (Rabbit polyclonal, Cell Signaling Technology, Danvers, MA), SMARCA3 (Rabbit polyclonal, Bethyl Laboratories, Montgomery, TX, 1: 2,000), Lamin B2 (mouse monoclonal, Santa Cruz, Dallas, TX, 1:1,000), Neurensin-1 (Elabscience Biotechnology, Houston, Texas, 1:1,000), and p11 (goat polyclonal, 1:200, R&D systems, Minneapolis, MN).

TRAP-ATAC-seq

Nuclei preparation and sorting

Adult GAD2^{TRAP} mice were used for ATAC-seq experiments. Six hippocampi from 3 mice were quickly dissected on ice and washed with ice-cold PBS. Nuclei preparation and sorting was conducted as previously described [12], with mild modifications. Samples were homogenized in ice-cold homogenization buffer containing (all in mM) 320 sucrose, 5 CaCl2, 3 MgCl2, 10 Tris, pH = 8.0, 0.1 EDTA, 0.1% IPEGAL supplemented with protease inhibitor cocktail, complete-EDTAfree (Roche, Mannheim, Germany). For gradient centrifugation, 50% OptiPrep density gradient medium (Sigma Aldrich)

containing (all in mM) 5 $CaCl_2$, 3 $MgCl_2$, 10 Tris pH = 8, was added and mixed. The lysate was gently loaded on the top of 10 ml 29% OptiPrep solution. Samples were centrifuged for 30 min at 4°C using a swinging bucket rotor in a WX Ultra 80 centrifuge (Thermo Fisher Scientific). The nuclei pellet was gently re-suspended in isotonic buffer, supplemented with protease inhibitor, 10 μM Dyecycle Ruby (Invitrogen, Carlsbad, CA) and 1% BSA. Samples were then incubated for 30 min and subjected to sorting using a fluorescence activated sorter (FACS). Nuclear preparation was sorted with a FACSAria (BD, San Jose, CA, USA) cell sorter equipped with 640-nm and 488-nm excitation lasers and a 70 μm nozzle. Nuclei were gated by two criteria: the presence of a GFP signal above the background fluorescence level (as assessed by comparison with nuclei obtained from WT, a non-GFP expressing littermate mouse) and the signal from DyeCycle ruby corresponding to a single nucleus. 50,000 sorted nuclei were collected in 100 μL of isotonic buffer supplemented with 5 mM $CaCl_2$. All GFP- samples were validated for GFP+ nuclei by post sorting. Random samples from GFP- and GFP+ were analyzed for nuclei purity and integrity by ImageStream imaging flow cytometer (Amnis). Images were acquired (× 5,000 cells/sample; objective 40×) and data were analyzed with IDEAS 4.0 software.

ATAC-seq libraries preparations

Sorted nuclei were centrifuged for 20 min at 4°C. The Nuclei pellet was re-suspended in 50 μL of Transposase reaction (Illumina) and incubated for 30 min at 37 °C. Then, DNA was purified with Qiagen MinElute kit (Qiagen) and frozen at -20°C until the next step. Library preparation was then generated following the well-established protocol [13]. Libraries were then quantified and verified by 4200 TapeStation System (Agilent) and sequenced by Illumina NextSeq 500 Sequencer.

ATAC-seq data analysis

ATAC-seq reads were aligned to the mm10 genome adapted from the Bsgenome.Mmusculus. UCSC.mm10 Bioconductor package (version 1.4.0) using Rsubread's align method in paired end mode with fragments between 1 to 5,000 base-pairs considered properly paired [14]. BigWigs were created from

properly paired reads as defined by Rsubread and normalized to reads per million using the Rtracklayer package. Fragment length distribution plots were produced using the soGGi package [15]. TransData was visualized in IGV software.

Live cell imaging

$7.5x10^4$ Cos7 cells (ATCC, Manassas, VA, USA) were seeded on glass-bottomed dishes (MatTek Corp, Ashland, MA, USA) and after 24 h were transiently transfected using Lipofectamine 2000 (Invitrogen). N2a cells (ATCC, Manassas, VA, USA) were transfected using the 4D-Nucleofector (Lonza) and seeded on glass-bottomed dishes immediately after transfection at a density of $1x10^5$ cells/dish. Rab5 tagged with mcherry and Nrsn2 tagged with EGFP (Genecopoeia, Rockville, MD, USA) plasmids were used for transfections. Prior to imaging, growth media was washed and cells were imaged in Live Cell Imaging solution (Life Technologies). All live cell imaging was carried out at 37^oC and 5% CO_2. Spinning-disk confocal microscopy was performed using an Andor Dragonfly system equipped with a PlanApo objective (63X, 1.4NA, Oil) and a Zyla sCMOS camera. Quantification of co-localization was measured using a custom written Matlab code [16]. Only clear, whole, hollow vesicular structures were quantified. The pixel mean value of Rab5 inside one Neurensin-2 (GFP) positive vesicle was divided by its level outside the cell. Vesicles with Rab5 mean pixel values above 140% of the background were considered immunopositive for co-localization.

Immuno-electron microscopy

N2A cells were stably transfected with Nrsn-2 tagged with EGFP in its N terminus (GeneCopoeia). For Imaging, cells were detached from the culture dish using Accutase (Innovative Cell Technologies, Inc., San Diego, CA). Cells were then subjected to high pressure freezing (Leica EMPAC2, Vienna, Austria) followed by immediate freeze substitution in 0.2% uranyl acetate in 95% acetone and 5% water. Subsequently they were embedded in Lowicryl HM20 resin at -50C° and cut into 80nm ultrathin sections. These sections were subjected for immunology gold labeling. Briefly, the sections were incubated with a

blocking solution, containing 3% BSA and 0.10% saponin, 0.10% cold fish skin gelatin and 1.5% sodium chloride in 20mM TBS (pH 7.4) for 1 hour at room temperature and followed by primary antibody incubation with anti-GFP raised in chicken (Abcam,1:150 dilution) and anti-clathrin raised in rabbit (Abcam, 1:60 dilution) in the blocking solution for overnight at 4°C. Those antigen-antibody complexes were recognized by the anti-chicken and anti-rabbit antibody conjugated with 18nm and 12nm colloidal gold (Jackson immunoResearch Lab Inc., West Grover, PA) for 1 hour at room temperature. Negative control was conducted by following the same procedure, except for omitting the primary antibody incubation. The sections were examined under a transmission electron microscope (120 KV, ThermoFisher Scientific, TECNAI BioTwin, Hillsboro, OR) equipped with the digital imaging system (BioSprint 6576 x 4384, Advanced Microscopy Techniques, Wobum, MA) in the Electron Microscopy Resource Center in The Rockefeller University.

AAV preparation and stereotaxic delivery

A sequence of the mouse Nrsn2 gene flanked by an internal ribosome entry site (IRES) and GFP was inserted into pAAV.Flex plasmid and was validated by sequencing. rAAV2 Flex.Nrsn2-IRES GFP or rAAV2.Flex.GFP were packaged at the Virus Vector Core Facility, UNC (Chapel Hill, NC, USA). 8 week-old mice were injected with 1 μl of AAV to the DG. The needle was left in the injection site for five minutes for complete diffusion. Injection coordinates were: ±2.00, −2.92 and −2.20 mm lateral, posterior and ventral relative to Bregma, according to the Franklin and Paxinos Mouse Brain atlas, 3rd edition. Three weeks post injection, behavioral tests or physiological recordings were conducted. Successful injections were verified by visualization of GFP under florescence microscope. Injection-site illustration in fig. 4 was generated using Brain Explorer 2 software, Allen Brain Atlas. For quantification of transfections, GFP positive cells at the SGZ were counted.

Slice preparation and electrophysiology

12-16 week old mice were euthanized by CO_2. After decapitation and removal of the brains, hippocampal transversal slices (400 µm thickness) or cerebellar coronal slices (300 µm thickness) were cut using a Vibratome 1000 Plus (Leica Microsystems, USA) at 2 °C in a cutting solution containing (all in mM): 93 NMDG, 1.2 $NaHPO_4$, 2.5 KCl, 0.5 $CaCl_2$, 10 $MgCl_2$, 30 $NaHCO_3$, 20 HEPES, 25 glucose, 5 sodium ascorbate, 2 thiourea, and 3 sodium pyruvate, and saturated with 95% O_2 and 5% CO_2. After cutting, slices were recovered for 15 min at 36°C and then for 2-4 h at room temperature (RT) in the recording solution (see below). The extracellular solution used for recordings contained (all in mM): 125 NaCl, 25 $NaHCO_3$, 2.5 KCl, 1.25 NaH_2PO_4, 2 $CaCl_2$, 1 $MgCl_2$ and 10 glucose (bubbled with 95% O_2 and 5% CO_2). For recordings, the slice was placed in a recording chamber (RC-27L, Warner Instruments, USA) and constantly perfused with oxygenated aCSF at 24°C (TC-324B, Warner Instruments, USA) at a rate of 1.5–2.0 ml/min. GFP-positive CCK cells in the SGZ were selected for recording based on fluorescent expression from genetic background, using an upright Olympus BX51WI microscope equipped with the appropriate filters (Olympus, Japan) and a SPECTRA X LED light engine (Lumencor, OR, USA). Cerebellar Purkinje neurons were visually selected for recordings based on their large cell body and their position adjacent to the cerebellar granular layer. Whole-cell patch-clamp recordings were performed with a Multiclamp 700B/Digidata1550A system (Molecular Devices, Sunnyvale CA, USA) and with glass pipettes (King Precision Glass, Inc, Glass type 8250) pulled in a horizontal pipette puller (Narishige) to a resistance of 3–4 MΩ. The intracellular solution contained (all in mM): 126 K-gluconate, 4 NaCl, 1 $MgSO_4$, 0.02 $CaCl_2$, 0.1 BAPTA, 15 glucose, 5 HEPES, 3 ATP, 0.1 GTP (pH 7.3). Recordings of miniature excitatory postsynaptic AMPA currents (mAMPA PSCs) were performed in the presence of tetradotoxin (0.5 µM) to block Na^+ currents, bicuculline (30 µM) to block GABA activity, and of APV (50 µM) to block NMDA-mediated excitatory currents. Data were acquired at a sampling frequency of 50 kHz, filtered at 1 kHz and analyzed offline using pClamp10 software (Molecular Devices, Sunnyvale, CA, USA). Data are shown as pooled events from each cell per treatment, and expressed as means ±

SEM. Statistical analysis was performed using Kolmogorov–Smirnov test or unpaired t test, with the help of GraphPad Prism 5. In all experiments, $p < 0.05$ was considered significant.

Co-immunoprecipitation (Co-IP)

$8x10^5$ N2a cells were seeded in 100 mm dish with cell media supplemented with 10% serum, 100 μg/mL Penicillin and 100 μg/mL Streptomycin. As cells reached 80% confluence, plasmids were transfected with Neurensin-2 tagged with 3 x HA on its N terminus (GeneCopoeia,) or empty plasmid with HA tag (Addgene, Watertown, MA,USA) using Lipofectamine 2000 reagent (Invitrogen). 36 h later, cells were lysed and Co-IP was done using MS-Compatible Magnetic IP Kit (ThermoFisher Scientific) following manufacturer's instructions and using rabbit anti-HA antibody (Rabbit monoclonal, Cell Signaling Technology) 5 μg/mg protein in lysate). Eluted proteins were subjected to SDS-PAGE followed by protein transfer onto a nitrocellulose membrane. Immunoblotting was performed with a standard protocol using the following antibodies: anti Neurensin-2 (mouse monoclonal, Sigma Aldrich,1:750), anti Arpc1b (Bethyl Laboratories) anti clathrin heavy chain (rabbit polyclonal, Abcam 1:1000), anti VPS37B (rabbit polyclonal, Abcam 1:500) and anti Homer3 (mouse monoclonal, Santa Cruz Biotechnology, 1:500).

Mass spectrometry

Following Co-IP, proteins were subjected to mass spectrometry as previously described [17]. In brief, proteins were eluted and reduced in 8M urea/10mM DTT and alkylated (30 mM iodoacetamide) and diluted 2-fold prior to overnight digestion with Endopeptidase LysC (Wako Chemicals, Richmond, VA, USA). Digest was further diluted 2-fold and then digested with trypsin. Digestion was halted by acidification. Peptides were desalted and analyzed using nano-LC-MS/MS (EasyLC1200 and Fusion Lumos operated in High-High mode, ThermoFisher, Scientific). Data were queried against UniProts mouse database concatenated with common contaminants and quantitated using Mascot and ProteomeDiscoverer. Matches were filtered using a Peptide False discovery rates of 1%. Data was generated by The Proteomics Resource Center at The Rockefeller University. Putative Neurensin-2

interacting-proteins were considered positive when no signal was detected in the control samples while all 3 experimental replicates had notable binding signals. For Co-IP data, STRING (version 11.0, https://string-db.org) was used for calculation of the GO term enrichment for cell component.

Synaptosomes Preparation

Fresh tissue was homogenized in 10 ml of ice–cold 0.32 M sucrose with a motor–driven (300-900 rpm) Teflon–glass homogenizer, and the homogenate was centrifuged for 5 min at 3,020g. The supernatant was then centrifuged for 12 min at 14,600g. The pellet was re-suspended in 2 ml of sucrose, manually grinded using dounce homogenizer and layered over 6 ml of Percoll (Sigma Aldrich) gradient discontinuous (23, 10 and 3%, 2 ml of each), and was centrifuged for 7 min at 35,100g. The ring-shaped synaptosome pellet formed between the 10 and 23% gradients was gently collected and centrifuged for 17 min at 30,000g. The pellet was dissolved in 1% SDS with protease inhibitor and was briefly sonicated. Protein concentration was determined by the BCA method. 10 μg protein was then loaded on SDS gel and proteins were detected using primary antibodies to AMPA2 (mouse monoclonal, 1:1000, MilliporeSigma, Burlington,MA, USA), Homer1 (Rabbit polyclonal, Cell Signaling Technology, 1:1000) and PSD95 (Rabbit polyclonal, Cell Signaling Technology, 1:1000)

Statistical analysis

In all experiments, $p < 0.05$ was considered significant and the variance was similar between the groups that were statistically compared. For one-way ANOVA. Significance was determined by Bonferroni's post hoc test. For two-way ANOVA significance was determined by Tukey's post hoc test. Sample size was chosen based on previous reports to ensure adequate power and mice were randomly allocated to experimental groups. Statistical analysis was performed using GraphPad Prism 7.02

References for Supplementary Methods section:

1. Heiman M, Kulicke R, Fenster RJ, Greengard P, Heintz N. Cell type-specific mRNA purification by translating ribosome affinity purification (TRAP). *Nat Protoc* 2014; **9**(6)**:** 1282-1291.

2. Roussarie JP, Yao V, Rodriguez-Rodriguez P, Oughtred R, Rust J, Plautz Z *et al.* Selective Neuronal Vulnerability in Alzheimer's Disease: A Network-Based Analysis. *Neuron* 2020; **107**(5)**:** 821-835.e812.

3. Chen S, Zhou Y, Chen Y, Gu J. fastp: an ultra-fast all-in-one FASTQ preprocessor. *Bioinformatics* 2018; **34**(17)**:** i884-i890.

4. Dobin A, Davis CA, Schlesinger F, Drenkow J, Zaleski C, Jha S *et al.* STAR: ultrafast universal RNA-seq aligner. *Bioinformatics* 2013; **29**(1)**:** 15-21.

5. H Backman TW, Girke T. systemPipeR: NGS workflow and report generation environment. *BMC Bioinformatics* 2016; **17:** 388.

6. Love MI, Huber W, Anders S. Moderated estimation of fold change and dispersion for RNA-seq data with DESeq2. *Genome Biol* 2014; **15**(12)**:** 550.

7. Svenningsson P, Chergui K, Rachleff I, Flajolet M, Zhang X, El Yacoubi M *et al.* Alterations in 5-HT1B receptor function by p11 in depression-like states. *Science* 2006; **311**(5757)**:** 77-80.

8. Warner-Schmidt JL, Schmidt EF, Marshall JJ, Rubin AJ, Arango-Lievano M, Kaplitt MG *et al.* Cholinergic interneurons in the nucleus accumbens regulate depression-like behavior. *Proc Natl Acad Sci U S A* 2012; **109**(28)**:** 11360-11365.

9. Virk MS, Sagi Y, Medrihan L, Leung J, Kaplitt MG, Greengard P. Opposing roles for serotonin in cholinergic neurons of the ventral and dorsal striatum. *Proc Natl Acad Sci U S A* 2016; **113**(3)**:** 734-739.

10. Deacon RM. Assessing nest building in mice. *Nat Protoc* 2006; **1**(3)**:** 1117-1119.

11. Golden SA, Covington HE, Berton O, Russo SJ. A standardized protocol for repeated social defeat stress in mice. *Nat Protoc* 2011; **6**(8)**:** 1183-1191.

12. Jordi E, Heiman M, Marion-Poll L, Guermonprez P, Cheng SK, Nairn AC *et al.* Differential effects of cocaine on histone posttranslational modifications in identified populations of striatal neurons. *Proc Natl Acad Sci U S A* 2013; **110**(23)**:** 9511-9516.

13. Buenrostro JD, Wu B, Chang HY, Greenleaf WJ. ATAC-seq: A Method for Assaying Chromatin Accessibility Genome-Wide. *Curr Protoc Mol Biol* 2015; **109:** 21.29.21-29.

14. Liao Y, Smyth GK, Shi W. The R package Rsubread is easier, faster, cheaper and better for alignment and quantification of RNA sequencing reads. *Nucleic Acids Res* 2019; **47**(8)**:** e47.

15. de Santiago I, Carroll T. Analysis of ChIP-seq Data in R/Bioconductor. *Methods Mol Biol* 2018; **1689:** 195-226.

16. Medrihan L, Sagi Y, Inde Z, Krupa O, Daniels C, Peyrache A *et al*. Initiation of Behavioral Response to Antidepressants by Cholecystokinin Neurons of the Dentate Gyrus. *Neuron* 2017; **95**(3)**:** 564-576.e564.

17. Rappsilber J, Mann M, Ishihama Y. Protocol for micro-purification, enrichment, pre-fractionation and storage of peptides for proteomics using StageTips. *Nat Protoc* 2007; **2**(8)**:** 1896-1906.
