# Supplementary material for: Identification of Neurensin-2 as a novel modulator of emotional behavior
Source: Mol Psychiatry. 2021 Mar 19;26(7):2872–85. doi: 10.1038/s41380-021-01058-5 (PMC8505262; doi:10.1038/s41380-021-01058-5)
Supplement: Supplementary file 2 — Supplemental Figures [file 41380_2021_1058_MOESM2_ESM.pdf]

Figure S1

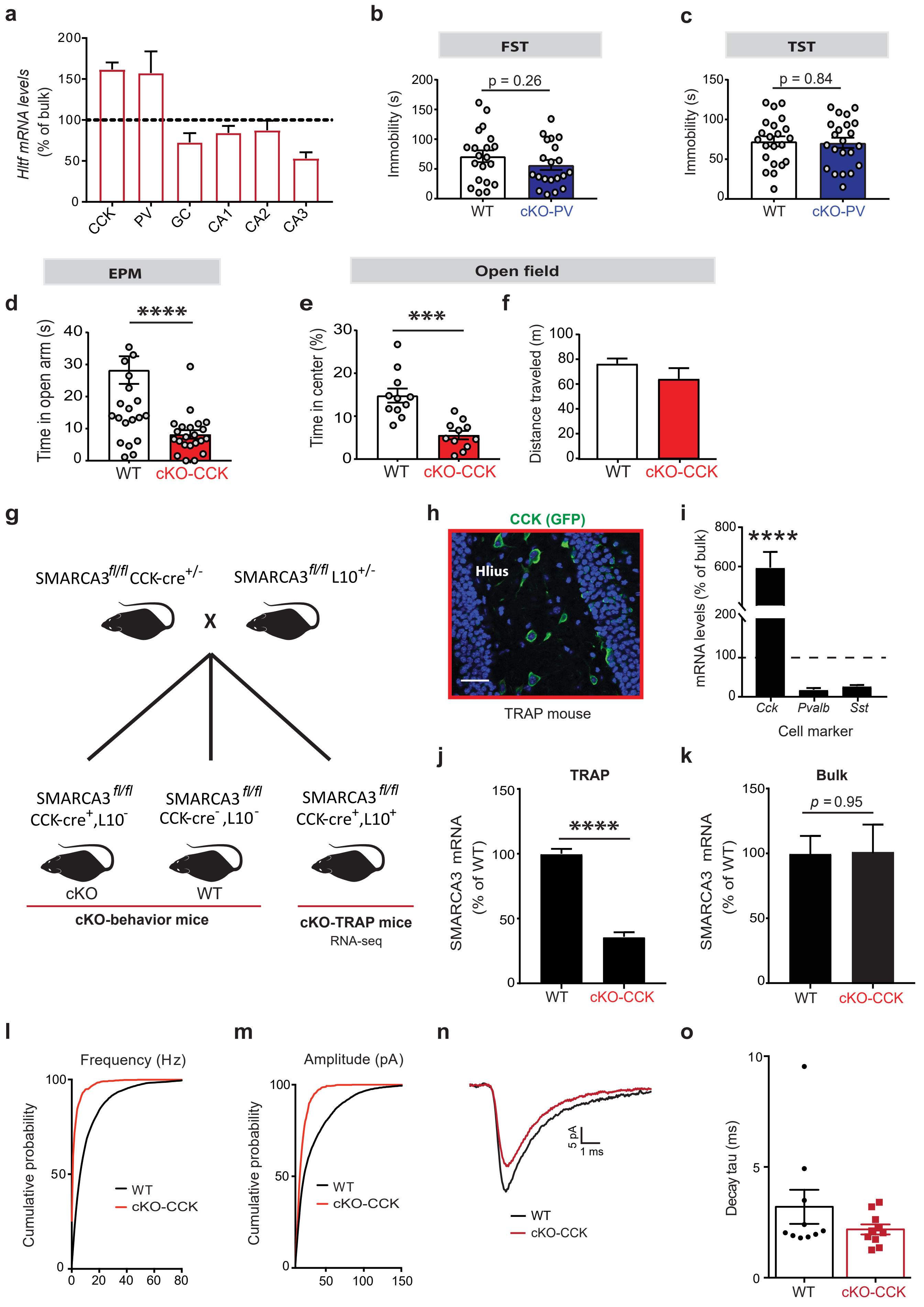
a
Hltf mRNA levels (% of bulk)
200
150
100
50
0
CCK
PV
GC
CA1
CA2
CA3
b
FST
p = 0.26
Immobility (s)
WT
cKO-PV
c
TST
p = 0.84
Immobility (s)
WT
cKO-PV
EPM
Open field
d
****
Time in open arm (s)
WT
cKO-CCK
e
***
Time in center (%)
WT
cKO-CCK
f
Distance traveled (m)
WT
cKO-CCK
g
SMARCA3^fl/fl CCK-cre^+/-
SMARCA3^fl/fl L10^+/-
X
SMARCA3^fl/fl CCK-cre^+,L10^-
SMARCA3^fl/fl CCK-cre^-,L10^-
SMARCA3^fl/fl CCK-cre^+,L10^+
cKO
WT
cKO-behavior mice
cKO-TRAP mice
RNA-seq
h
CCK (GFP)
Hlius
TRAP mouse
i
****
mRNA levels (% of bulk)
Cck
Pvalb
Sst
Cell marker
j
TRAP
****
SMARCA3 mRNA (% of WT)
WT
cKO-CCK
k
Bulk
p = 0.95
SMARCA3 mRNA (% of WT)
WT
cKO-CCK
l
Frequency (Hz)
Cumulative probability
WT
cKO-CCK
m
Amplitude (pA)
Cumulative probability
WT
cKO-CCK
n
5 pA
1 ms
WT
cKO-CCK
o
Decay tau (ms)
WT
cKO-CCK

Figure S2

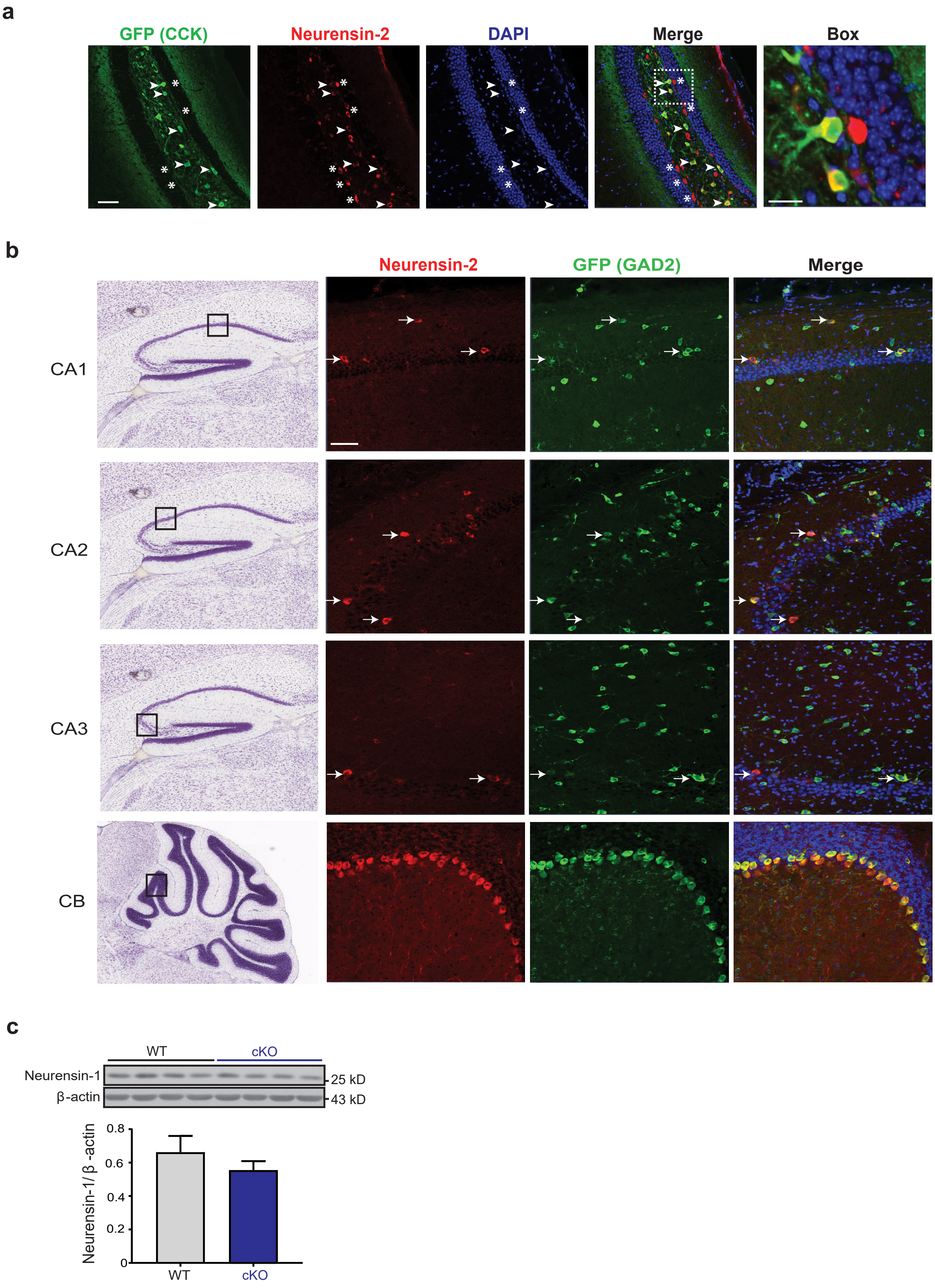

a
GFP (CCK)
Neurensin-2
DAPI
Merge
Box
b
Neurensin-2
GFP (GAD2)
Merge
CA1
CA2
CA3
CB
c
WT
cKO
Neurensin-1
β-actin
25 kD
43 kD
Neurensin-1/β -actin
0.8
0.6
0.4
0.2
0
WT
cKO

Figure S3

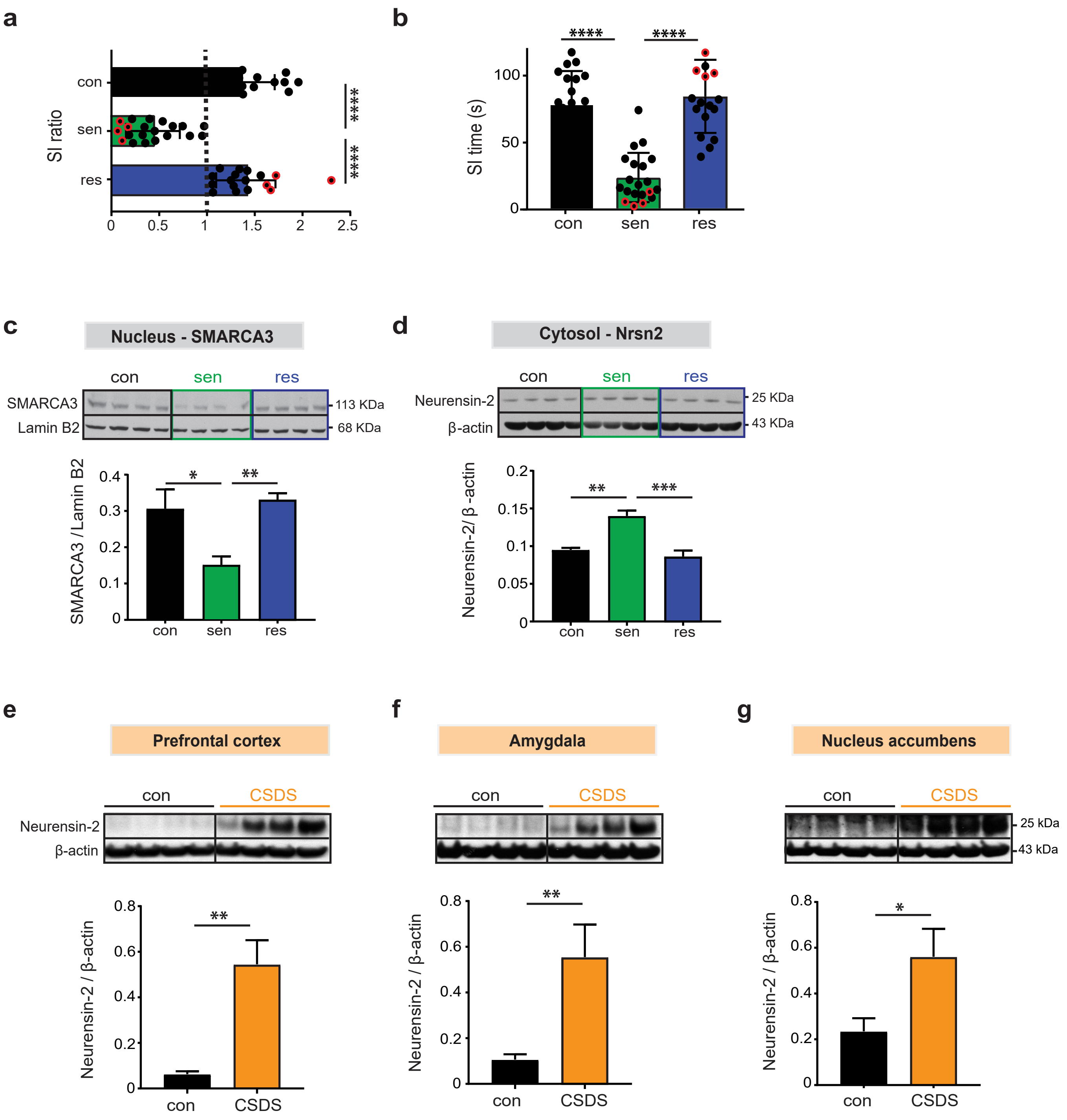
a
con
sen
res
SI ratio
0
0.5
1
1.5
2
2.5
****
****
b
****
****
100
50
0
SI time (s)
con
sen
res
c
Nucleus - SMARCA3
con
sen
res
SMARCA3
Lamin B2
113 KDa
68 KDa
0.4
0.3
0.2
0.1
0
SMARCA3 /Lamin B2
*
**
con
sen
res
d
Cytosol - Nrsn2
con
sen
res
Neurensin-2
β-actin
25 KDa
43 KDa
0.2
0.15
0.1
0.05
0
Neurensin-2/ β -actin
**
***
con
sen
res
e
Prefrontal cortex
con
CSDS
Neurensin-2
β-actin
0.8
0.6
0.4
0.2
0
Neurensin-2 / β-actin
**
con
CSDS
f
Amygdala
con
CSDS
0.8
0.6
0.4
0.2
0
Neurensin-2 / β-actin
**
con
CSDS
g
Nucleus accumbens
con
CSDS
25 kDa
43 kDa
0.8
0.6
0.4
0.2
0
Neurensin-2 / β-actin
*
con
CSDS

Figure S4

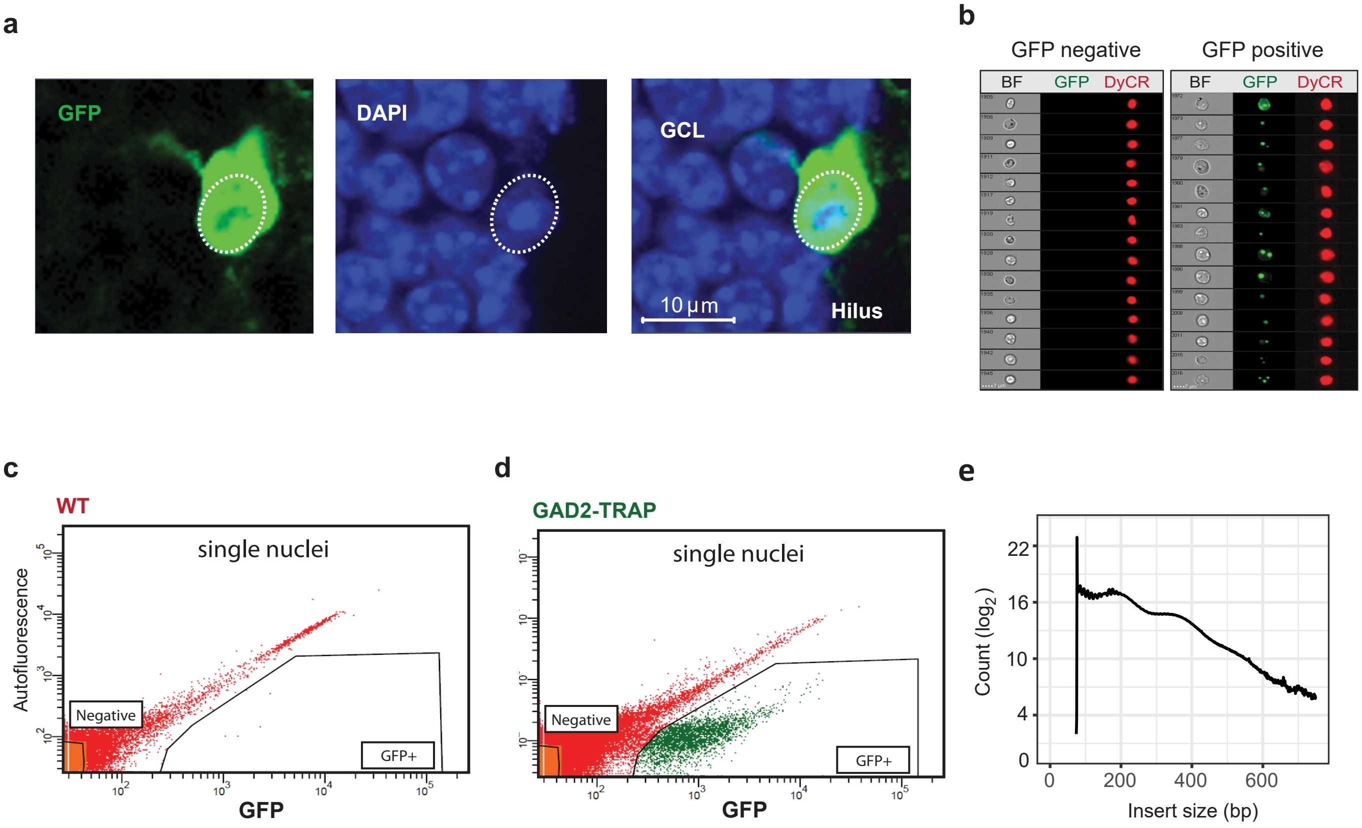
a
GFP
DAPI
GCL
10 μm
Hilus
b
GFP negative
GFP positive
BF
GFP
DyCR
c
WT
single nuclei
Autofluorescence
Negative
GFP+
GFP
d
GAD2-TRAP
single nuclei
Negative
GFP+
GFP
e
Count (log2)
Insert size (bp)
22
16
10
4
0
0
200
400
600

Figure S5

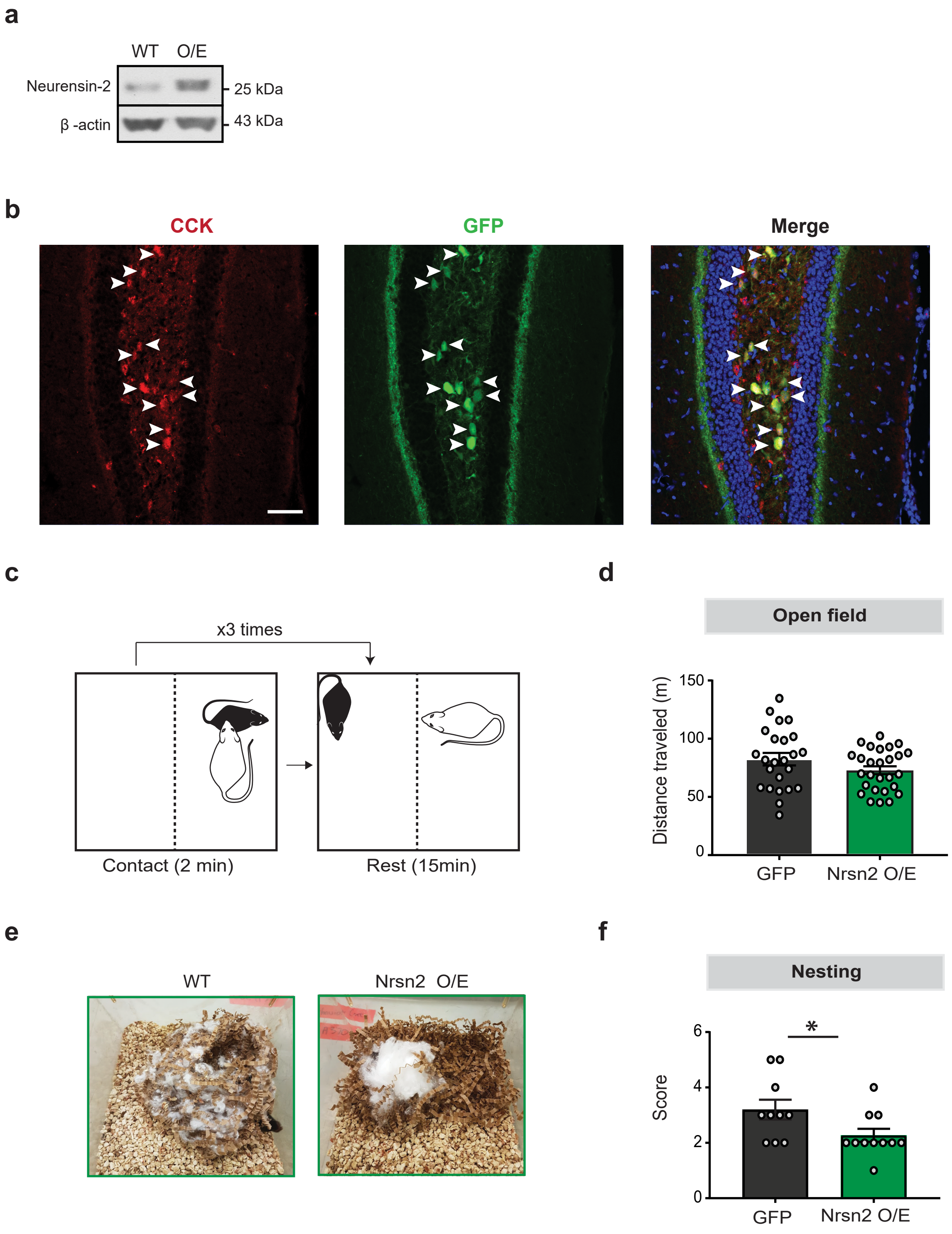
a
WT
O/E
Neurensin-2
β -actin
25 kDa
43 kDa
b
CCK
GFP
Merge
c
x3 times
Contact (2 min)
Rest (15min)
d
Open field
Distance traveled (m)
150
100
50
0
GFP
Nrsn2 O/E
e
WT
Nrsn2 O/E
f
Nesting
Score
6
4
2
0
*
GFP
Nrsn2 O/E

Figure S6

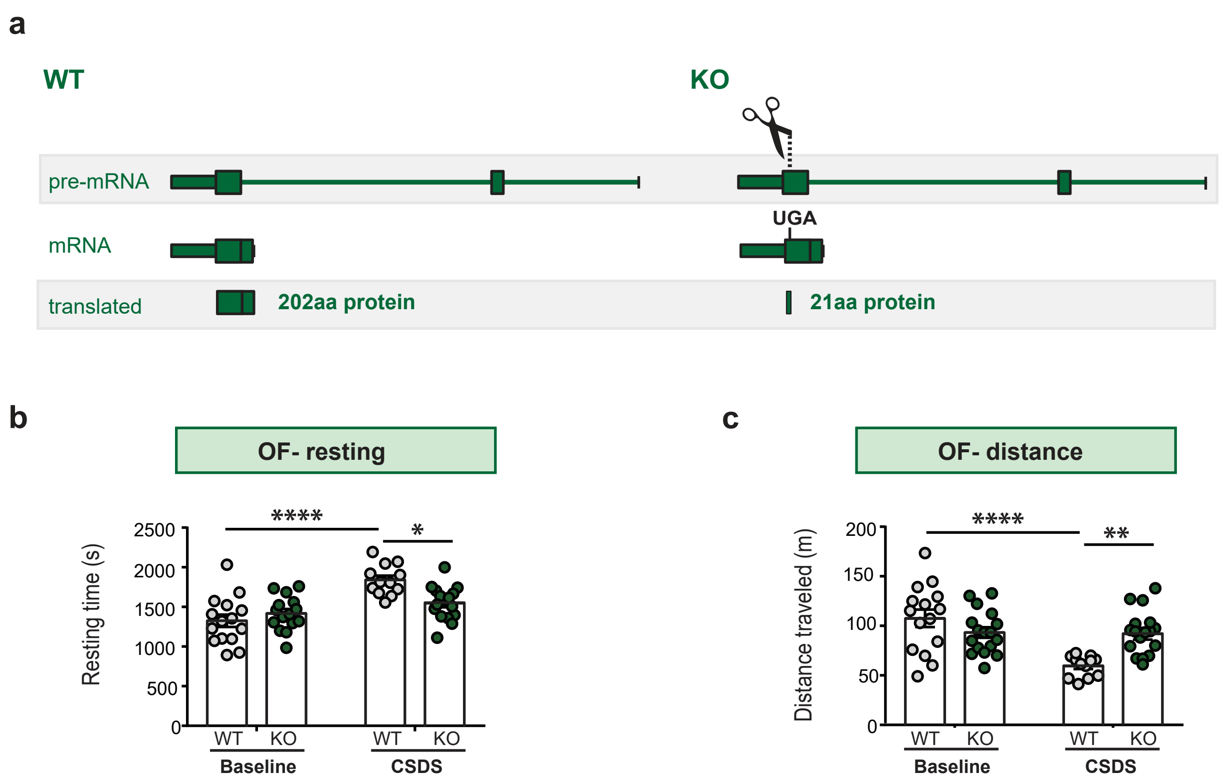
a
WT
KO
pre-mRNA
UGA
mRNA
translated
202aa protein
21aa protein
b
OF- resting
Resting time (s)
2500
2000
1500
1000
500
0
****
*
WT
KO
WT
KO
Baseline
CSDS
c
OF- distance
Distance traveled (m)
200
150
100
50
0
****
**
WT
KO
WT
KO
Baseline
CSDS

Figure S7

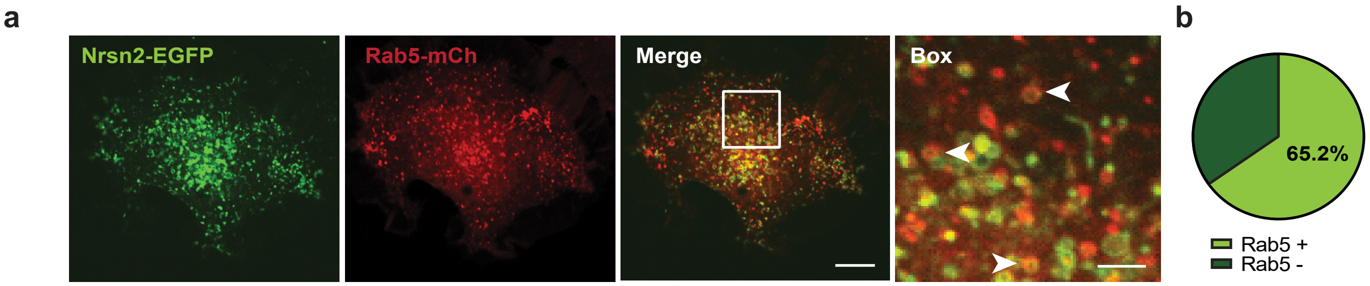

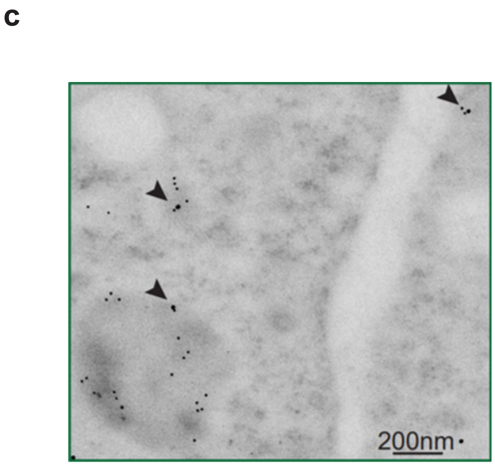

Supplemental figures legends

Figure S1. Generation, validation and characterization of SMARCA3 cKO-CCK mice. Related to Figure 1.

(a) Bar graph showing the enrichment of SMARCA3 gene (*Hltf*) in different hippocampal cell-types, compared to bulk mRNA preparation. TRAP data was obtained from cholecystokinin (CCK, n = 10) parvalbumin (PV) granule cells (CG), CA1, CA2 and CA3 neurons (n = 3/ group).

(b-c) WT or mice with SMARCA3 conditional KO in PV cells (cKO-PV) were tested behaviorally: (b) FST, forced swim test, unpaired t-test, n = 20, 20. (c) TST, tail suspension test, unpaired t-test, n =22, 22.

(d-e) WT or mice with SMARCA3 conditional KO in CCK cells (cKO-CCK) were tested behaviorally: (d) EPM, elevated plus maze, unpaired t-test, ****$p<0.0001$, n = 21, 24. (e) open field, ***$p = 0.0001$, n =11, 11. (f) Bar graph of distance travelled in the open field test. unpaired t-test; $p=0.17$, n = 11, 11.

(g) Schematic of breeding cKO-CCK mice and experimental design. Mice with exons 11-13 of *hltf* flanked by LoxP (SMARCA3$^{fl/fl}$) were used to target SMARCA3 deletion. Breeding mice carrying cre-recombinase (cre) under the CCK promoter (SMARCA3$^{fl/fl}$ CCK-cre$^{+/-}$) with mice carrying flox-stop-flox-EGFP-L10a (SMARCA3$^{fl/fl}$ -L10$^{+/-}$) generated 3 groups of experimental mice; mice carrying only cre (SMARCA3$^{fl/fl}$ CCK-cre^{+}, L10^{-}) were used as cKO-CCK for behavioral tests. Mice carrying neither cre nor EGFP-L10a (SMARCA3$^{fl/fl}$ CCK-cre^{-}, L10^{-}) were used as control in behavioral tests. Mice carrying both cre and EGFP-L10a (SMARCA3$^{fl/fl}$ CCK-cre^{+}, L10^{+}) were used as cKO for TRAP RNAseq and ATAC-seq. WT mice carrying CCK-cre^{+} and L10^{+} were used as TRAP controls (not shown).

(h) Representative image of GFP expression in the dentate gyrus (DG) of CCK-TRAP mouse. Scale bar, 20 μM.

(i) Bar graph of translated gene expression levels in CCK cells for 3 genes: *Cck*, parvalbumin (*Pvalb*), and somatostatin (*Sst*). Data is represented as percentage (enrichment) of the gene levels in the bulk mRNA (100%, dashed line). One way ANOVA; **** $p < 0.0001$, n = 9, 10.

(j) qPCR analysis of SMARCA3 translated mRNA levels in CCK cells. Translated mRNA from CCK hippocampal cells was isolated from WT (n = 15 from 30 mice) and cKO-CCK mice (n = 17 from 34 mice). Unpaired t-test; **** $p < 0.0001$.

(k) qPCR analysis of SMARCA3 transcript levels in bulk hippocampal mRNA extraction from WT mice and cKO-CCK. Unpaired t-test, n = 5/ group.

(l-m) Cumulative percentage graphs of AMPA mPSCs frequency (l) and amplitude (m) in SGZ CCK neurons. WT mice, n=4,824 events, 10 cells, 5 mice. cKO-CCK mice n=849, 10, 4.

(n) Averaged AMPA mPSCs traces in DG SGZ CCK neurons, as measured from WT and cKO-CCK mice.

(o) Decay time (tau) of AMPA mPSCs in DG SGZ CCK neurons, as measured from WT (n=10 cells, 5 mice) and cKO-CCK mice (n=10 cells, 4 mice). Unpaired t-test; p = 0.22.

Figure S2. Cellular localization of Neurensin-2 in inhibitory neurons. Related to Figure 2.

(a) Representative immunohistochemical images showing localization of Neurensin-2 in CCK interneurons in the DG. Mice expressing Cre in CCK cells were injected with AAV2-FLEX-GFP and after 21 days were subjected to transcardial perfusion followed by immunolabeling of Neurensin-2 and GFP. Arrowheads point to GFP positive CCK cells that are co-express Neurensin-2. Asterisks mark Neurensin-2 expressing cells that are GFP negative (non-CCK cells). Scale bars: 50 μm, box: 20 μm.

(b) Representative immunohistochemical images showing localization of Neurensin-2 in interneurons in different structures of the hippocampal formation (CA1-CA3) and in cerebral Purkinje cells. Blue, DAPI. Scale bars: 50 μm. Nissl staining was adapted from Allan Brain Atlas.

(c) Immunoblot scan (top) and quantification (bottom) of Neurensin-1 in hippocampi from WT and from mice with SMARCA3 deletion in CCK cells (cKO). Unpaired t-test, $p = 0.37$, $n = 4$ mice /group.

Figure S3. Neurensin-2 is up-regulated is stress-sensitive mice. Related to Figure 3.

(a-b) Bar graphs showing the social interaction test (SI) following chronic social defeat stress (CSDS). (a) SI ratio of time spent exploring a novel aggressor compared to time spent exploring an empty mesh. One way ANOVA; F (2, 53) = 57.5, ****$p < 0.0001$, n = 19, 20, 17. (b) Time spent interacting with a novel aggressor. One way ANOVA; F (2, 53) = 46.0, ****$p < 0.0001$. n = 19, 20, 17. Mice selected for biochemical analysis are represented in red circles. Con, control; sen, sensitive; res, resilient

(c-d) Western blot scans (top) and quantification (bottom) of hippocampal cytosolic Neurensin-2 (c), and nuclear SMARCA3 (d). One way ANOVA; F (2, 9) = 7.93 *$p = 0.020$; **$p = 0.001$ (Neurensin-2), **$p=0.009$ (SMARCA3), ***$p=0.0004$, n = 4, 4, 4 mice. con, control; sen, sensitive; res, resilient.

(e-g) Western blot scan (top) and quantification (bottom) of Neurensin-2 protein expression in the prefrontal cortex (e), amygdala (f) and nucleus accumbens (g) of control mice and stress-sensitive mice after chronic social defeat stress (CSDS). t-test, *$p = 0.036$, **$p = 0.0014$ for prefrontal cortex, **$p = 0.0096$ for amygdala. n = 4-5/group.

Data are presented as means ± SEM.

Figure S4. Validations of ATAC-seq. Related to Figure 3.

(a) Magnified images of the GFP and DAPI channels from Figure 3g. GCL, granule cell layer.

(b) After sorting, cell nuclei are purified and intact as verified by Amnis ImageStream analysis. Representative results from post-sort fractions of GAD2-TRAP hippocampi. Bright-field (BF), GFP and dyecycle ruby (DyCR) are shown. Original magnification 40x.

(c-d) Fluorescence intensity scatter plots from WT (c) and $GAD2^{TRAP}$ mice (d). GFP positive cell nuclei are shown in the gating box.

(e) A representative ATAC sequencing fragment length distribution.

Figure S5. Neurensin-2 overexpression in DG CCK neurons impairs nesting behavior. Related to Figure 4.

(a) Neurensin-2 protein analysis from hippocampi of a WT mouse and a mouse with viral-mediated Nrsn2 O/E in CCK cells.

(b) Representative immunohistochemical images showing localization of Neurensin-2 in DG CCK interneurons. Mice were injected with AAV2-Flex-GFP virus to the DG and after 21 days were injected i.c.v with colchicine. Blue, DAPI. Arrowheads point to CCK positive cells that are transfected with the virus (GFP). Scale bar: 50 µm.

(c) Schematic of the Subthreshold Social Defeat Stress (SSDS) paradigm.

(d) Bar graph of distance travelled by mice during the open field test. Unpaired t-test*;* $p = 0.13$, $n = 24, 27$.

(e) Representative images of nesting behavior of GFP and Nrsn2 overexpressing mice (Nrsn2 O/E).

(f) Bar graph summarizing nesting behavior in WT and Nrsn2 O/E. Unpaired t-test, $^{*}p = 0.040$, $n = 10, 11$.

Data in b and d are represented as means ± SEM.

Figure S6. Generation and behavioral characterization of Nrsn2 KO mice. Related to Figure 4.

(a) Schematic of Nrsn2 KO mice generation. 5bp deletion in exon 2 of *Nrsn2* gene by CRISPR-Cas9 results in reading-frame shift and the creation of an early stop codon (UGA). The translated protein is a 22 amino acids protein instead of the WT 202 amino acids full length protein.

(b-c) WT or Nrsn2 KO mice were subjected to the open field test following chronic social defeat. (b) Resting time. Two-way ANOVA; interaction, F (1, 54) = 9.417, P= 0.0034; stress, P < 0.0001; genotype, P = 0.113. *p = 0.010, ****p < 0.0001, n = 12-16. (c) Distance traveled for 60 min. Two-way ANOVA; interaction, F (1, 54) = 15.66, P = 0.0002; stress, P = 0.0008; genotype, P = 0.112, ** p = 0.0017, ****p < 0.0001, n = 12-16.

Data in b and c are represented as means ± SEM.

Figure S7. Neurensin-2 is expressed in endosomes in neuronal N2a cells. Related to Figure 5.

(a-b) Representative images of live-cell imaging (a) and pie chart summary (b) showing colocalization of EGFP-tagged Neurensin-2 protein with mCherry-tagged Rab5 (Rab5-mCh) in N2a cells. Arrowheads indicate co-labeled vesicle-like structures. Scale bars: Merge, 5 µm; box, 1 µm. n = 115 vesicles, 8 cells.

(c) Immuno electron-microscopy image showing by arrowheads the proximate labeling of clathrin heavy-chain (18nm) and EGFP-tagged Neurensin-2 (12nm) on vesicle structures in N2a cells transfected with EGFP tagged Neurensin-2.
