# Supplementary material for: Identification of Neurensin-2 as a novel modulator of emotional behavior
Source: Mol Psychiatry. 2021 Mar 19;26(7):2872–85. doi: 10.1038/s41380-021-01058-5 (PMC8505262; doi:10.1038/s41380-021-01058-5)
Supplement: Supplementary file 3 — Table S1 [file 41380_2021_1058_MOESM3_ESM.pdf]

Table S1- RNA seq : CCK TRAP

All CCK changed genes

GeneSymbol	cckKO_meanRPKM	wt_meanRPKM	wt-cckKO_logFC	wt-cckKO_PValue	wt-cckKO_FDR
Nrsn2	209.13	76.13	-1.50	6.57E-16	1.01E-11
Gm14164	11.39	2.89	-2.02	9.71E-13	7.46E-09
Syp	99.30	56.30	-0.85	1.14E-08	5.82E-05
Gm10359	43.41	21.58	-1.04	4.14E-07	1.59E-03
Eif6	11.77	5.99	-1.01	6.40E-07	1.97E-03
Phyhip	74.54	44.91	-0.77	2.27E-06	3.87E-03
mt-Rnr1	931.24	364.56	-1.37	1.92E-06	3.87E-03
Gm7336	39.44	21.42	-0.92	1.93E-06	3.87E-03
Gm26917	58.61	17.58	-1.78	2.25E-06	3.87E-03
Ctsd	32.40	20.00	-0.73	1.04E-05	1.59E-02
Caskin1	14.60	9.74	-0.62	1.64E-05	2.11E-02
Gm14703	46.88	28.50	-0.75	1.62E-05	2.11E-02
Gna11	16.27	10.62	-0.65	2.08E-05	2.46E-02
Casp1	1.20	4.19	1.76	2.49E-05	2.46E-02
Gse1	24.02	12.13	-1.03	3.20E-05	2.46E-02
Atp1a3	163.97	110.62	-0.60	2.38E-05	2.46E-02
Selenow	126.96	65.41	-1.00	2.94E-05	2.46E-02
Chtf8	10.79	6.31	-0.81	2.62E-05	2.46E-02
Chchd2	200.96	296.74	0.53	3.05E-05	2.46E-02
Gm14760	39.28	22.21	-0.86	2.90E-05	2.46E-02
Gm12070	26.06	15.40	-0.80	3.36E-05	2.46E-02
Unc5a	21.16	13.59	-0.68	4.09E-05	2.78E-02
Rpl12	34.99	52.21	0.54	4.16E-05	2.78E-02
Gins2	33.41	18.10	-0.93	4.67E-05	2.93E-02
Adgrb1	16.08	10.20	-0.69	4.77E-05	2.93E-02
Slc17a7	162.97	118.74	-0.49	4.96E-05	2.93E-02
Edf1	83.38	57.16	-0.58	6.35E-05	3.52E-02
Gm21984	6.67	2.22	-1.63	6.40E-05	3.52E-02
Rps27a	64.86	98.38	0.57	7.53E-05	3.86E-02
Tra2a	19.01	91.53	2.24	7.32E-05	3.86E-02
Prrt2	141.08	92.09	-0.65	7.84E-05	3.89E-02
Pagr1a	106.82	69.93	-0.65	8.64E-05	4.15E-02
Cyb5r3	12.81	6.74	-0.97	9.49E-05	4.35E-02
Slc6a11	4.03	2.07	-0.99	9.88E-05	4.35E-02
Gm8430	142.03	221.51	0.61	9.90E-05	4.35E-02
Syt2	0.38	1.05	1.45	1.06E-04	4.48E-02
Jarid2	3.00	5.92	0.95	1.08E-04	4.48E-02
Sap30	10.15	21.15	1.03	1.20E-04	4.87E-02
mt-Co1	142.99	82.68	-0.82	1.46E-04	5.76E-02
Gm12671	37.71	22.02	-0.81	1.50E-04	5.76E-02
Hmgcs1	21.54	31.70	0.52	1.59E-04	5.82E-02
Gm12537	37.49	21.91	-0.81	1.57E-04	5.82E-02
Rpl27a	71.12	102.23	0.49	1.74E-04	6.23E-02
Lpcat4	19.62	12.00	-0.75	1.85E-04	6.24E-02
Ttll3	10.60	6.61	-0.72	1.82E-04	6.24E-02
Arhgap23	4.51	2.61	-0.82	1.87E-04	6.24E-02
Nkiras1	31.82	45.46	0.48	2.02E-04	6.61E-02
Tnrc18	3.01	1.13	-1.46	2.36E-04	7.39E-02
Rps27a-ps2	104.53	163.98	0.62	2.32E-04	7.39E-02

mt-Rnr2	926.82	406.87	-1.21	2.42E-04	7.44E-02
Sorcs2	1.94	1.11	-0.84	2.55E-04	7.55E-02
mt-Cytb	69.93	40.97	-0.80	2.54E-04	7.55E-02
Rptor	3.31	2.16	-0.65	2.85E-04	8.10E-02
mt-Nd1	97.19	59.03	-0.75	2.80E-04	8.10E-02
Prrc2a	26.93	19.05	-0.54	3.04E-04	8.50E-02
Reep2	20.77	13.12	-0.70	3.16E-04	8.66E-02
Nlrx1	1.18	0.37	-1.70	3.29E-04	8.87E-02
Lrrn2	46.69	32.64	-0.55	3.36E-04	8.91E-02
Trp53i11	10.12	6.43	-0.69	3.42E-04	8.91E-02
Nts	3.27	8.45	1.35	3.55E-04	8.94E-02
Gm26767	0.58	1.48	1.32	3.50E-04	8.94E-02
Tpd52l1	26.97	43.15	0.65	3.71E-04	8.94E-02
Fgf5	6.95	12.59	0.83	3.84E-04	8.94E-02
Basp1	637.41	829.45	0.35	3.61E-04	8.94E-02
Zfp710	2.63	1.24	-1.11	3.83E-04	8.94E-02
Haus3	1.11	3.75	1.73	3.84E-04	8.94E-02
Ptprs	35.08	25.54	-0.49	4.11E-04	9.24E-02
Ncor2	6.81	4.48	-0.64	4.06E-04	9.24E-02
Col4a2	2.17	1.14	-0.97	4.15E-04	9.24E-02
Ggct	6.82	10.65	0.61	4.79E-04	9.71E-02
Grina	159.36	121.49	-0.42	4.58E-04	9.71E-02
Scg5	120.79	73.49	-0.75	4.72E-04	9.71E-02
Rgs2	34.45	49.65	0.50	4.44E-04	9.71E-02
Hyou1	5.29	3.26	-0.73	4.80E-04	9.71E-02
Galnt17	8.52	6.19	-0.49	4.64E-04	9.71E-02
Gm23935	52440.63	34563.67	-0.63	4.79E-04	9.71E-02
Cnnm1	32.40	23.98	-0.47	5.05E-04	1.01E-01
Bcl9l	4.89	2.59	-0.96	5.23E-04	1.03E-01
Has3	3.40	2.09	-0.74	5.44E-04	1.05E-01
Eid2	56.55	80.94	0.49	5.37E-04	1.05E-01
Hsbp1l1	0.32	2.02	2.60	6.18E-04	1.17E-01
Aldh1l2	0.24	0.03	-3.03	6.31E-04	1.18E-01
Jag2	0.49	0.11	-2.14	7.07E-04	1.19E-01
Grik5	16.77	10.99	-0.65	6.47E-04	1.19E-01
Ddx46	6.98	10.34	0.54	7.23E-04	1.19E-01
Nudt16l1	24.04	35.65	0.54	7.43E-04	1.19E-01
Grin1	23.56	15.17	-0.67	6.62E-04	1.19E-01
Dennd1a	9.87	6.59	-0.62	6.96E-04	1.19E-01
Dusp26	11.13	18.25	0.68	6.64E-04	1.19E-01
Ttn	0.04	0.01	-1.92	6.84E-04	1.19E-01
Gm13691	5.48	1.30	-2.12	7.76E-04	1.19E-01
NA	0.62	0.00	-8.14	6.70E-04	1.19E-01
Gm6222	94.49	137.73	0.51	6.89E-04	1.19E-01
Gm13698	5.48	1.30	-2.12	7.75E-04	1.19E-01
Gm13693	5.48	1.30	-2.12	7.76E-04	1.19E-01
Gm13697	5.48	1.30	-2.12	7.76E-04	1.19E-01
Gm13694	5.48	1.30	-2.12	7.73E-04	1.19E-01
Gm13696	5.48	1.30	-2.12	7.72E-04	1.19E-01
Gm13695	5.50	1.30	-2.13	7.56E-04	1.19E-01
Gm26602	30.71	12.87	-1.29	7.09E-04	1.19E-01
Ube2e1	19.10	27.19	0.48	7.94E-04	1.21E-01

Syt17	33.84	23.89	-0.54	8.11E-04	1.22E-01
Vti1b	49.48	70.46	0.48	8.39E-04	1.24E-01
Pcnx2	6.57	4.54	-0.57	8.31E-04	1.24E-01
Prrt1	150.15	103.56	-0.57	8.82E-04	1.29E-01
Mir6236	1098.47	480.95	-1.23	9.39E-04	1.36E-01
Tmem132a	1.66	0.82	-1.05	9.67E-04	1.38E-01
Nfic	6.55	4.31	-0.64	9.62E-04	1.38E-01
Rogdi	98.04	71.02	-0.50	1.01E-03	1.39E-01
Hmgn1	21.17	29.49	0.45	1.02E-03	1.39E-01
Elfn2	14.97	9.53	-0.69	1.02E-03	1.39E-01
Spock2	67.91	48.12	-0.54	1.02E-03	1.39E-01
NA	1.93	0.57	-1.79	9.93E-04	1.39E-01
Slc22a15	1.86	0.20	-3.28	1.05E-03	1.39E-01
Rai1	6.40	4.53	-0.54	1.04E-03	1.39E-01
Eif5a	84.29	56.57	-0.61	1.04E-03	1.39E-01
Ubald1	11.08	8.04	-0.50	1.06E-03	1.39E-01
Neurl1a	8.70	6.01	-0.57	1.07E-03	1.39E-01
Fam45a	11.11	7.31	-0.63	1.12E-03	1.42E-01
Slit1	7.70	5.31	-0.57	1.12E-03	1.42E-01
Kcnc3	15.08	10.34	-0.58	1.10E-03	1.42E-01
Tmem121b	8.49	5.51	-0.66	1.12E-03	1.42E-01
Setd1a	7.77	5.03	-0.67	1.14E-03	1.42E-01
Pom121	22.93	16.74	-0.49	1.15E-03	1.43E-01
Slc35a4	13.13	8.45	-0.67	1.19E-03	1.45E-01
Arid4b	3.47	5.06	0.51	1.19E-03	1.45E-01
mt-Nd5	65.11	45.06	-0.56	1.18E-03	1.45E-01
Man1c1	4.15	2.54	-0.74	1.23E-03	1.46E-01
Grin1os	22.37	13.24	-0.80	1.24E-03	1.46E-01
Cox16	16.96	23.96	0.47	1.22E-03	1.46E-01
Tmem63c	7.14	4.57	-0.68	1.27E-03	1.47E-01
Mcrip1	8.24	4.32	-0.96	1.26E-03	1.47E-01
Sema4f	10.39	6.77	-0.66	1.29E-03	1.49E-01
Scap	6.31	4.24	-0.61	1.31E-03	1.50E-01
Sh3bp1	9.65	6.61	-0.58	1.36E-03	1.54E-01
Gm12350	231.58	322.87	0.45	1.35E-03	1.54E-01
Ywhah	1358.06	1688.52	0.28	1.44E-03	1.59E-01
Zrsr1	11.15	16.19	0.50	1.44E-03	1.59E-01
Rad21l	0.20	1.17	2.55	1.43E-03	1.59E-01
Tmem254b	13.44	25.66	0.89	1.49E-03	1.61E-01
Tmem254a	13.44	25.66	0.89	1.49E-03	1.61E-01
Gm26821	40.45	27.47	-0.60	1.48E-03	1.61E-01
Rheb	24.46	34.06	0.45	1.57E-03	1.69E-01
Prpf39	56.99	77.17	0.41	1.59E-03	1.69E-01
2810455O05Rik	7.04	11.94	0.73	1.59E-03	1.69E-01
Slc25a22	14.07	9.42	-0.62	1.64E-03	1.71E-01
Rtraf	66.49	91.59	0.43	1.64E-03	1.71E-01
Mbd1	2.16	4.83	1.14	1.65E-03	1.71E-01
Ly6e	101.41	78.30	-0.41	1.71E-03	1.71E-01
Gnl1	13.59	19.59	0.50	1.67E-03	1.71E-01
Cln3	1.10	0.41	-1.46	1.68E-03	1.71E-01
Atp6v0e2	72.30	54.58	-0.44	1.72E-03	1.71E-01
Commd7	4.25	6.52	0.58	1.71E-03	1.71E-01

Gm2606	24.54	15.30	-0.71	1.71E-03	1.71E-01
Lynx1	78.90	60.55	-0.42	1.87E-03	1.73E-01
Tsc22d3	7.04	11.66	0.70	1.87E-03	1.73E-01
Zbed5	52.35	73.58	0.46	1.84E-03	1.73E-01
Prrc2b	29.69	22.11	-0.46	1.87E-03	1.73E-01
Plppr2	29.94	19.64	-0.65	1.78E-03	1.73E-01
Hebp1	68.17	105.24	0.60	1.83E-03	1.73E-01
Tmem254c	15.53	29.42	0.88	1.86E-03	1.73E-01
Pin4	30.73	46.24	0.56	1.77E-03	1.73E-01
Pabpc1l2b-ps	18.82	9.92	-0.96	1.84E-03	1.73E-01
Lrch4	3.35	1.87	-0.88	1.80E-03	1.73E-01
Muc3a	0.12	0.99	3.04	1.78E-03	1.73E-01
NA	5.31	0.70	-2.95	1.82E-03	1.73E-01
Psd	56.17	34.98	-0.72	1.91E-03	1.73E-01
Prr12	11.09	5.38	-1.09	1.92E-03	1.73E-01
Naca	29.02	38.27	0.37	1.91E-03	1.73E-01
Zfp26	3.80	2.56	-0.60	1.91E-03	1.73E-01
Gm24187	31260.76	21893.46	-0.55	1.94E-03	1.75E-01
Prkaca	115.86	87.13	-0.45	2.00E-03	1.77E-01
Cntnap1	15.88	11.52	-0.50	2.01E-03	1.77E-01
Gm10222	92.45	52.66	-0.84	1.99E-03	1.77E-01
Shank3	3.17	1.85	-0.81	2.08E-03	1.81E-01
mt-Nd4l	92.67	52.84	-0.84	2.07E-03	1.81E-01
Rsrp1	5.63	9.77	0.75	2.14E-03	1.84E-01
Meis3	4.22	2.34	-0.89	2.12E-03	1.84E-01
Ube2s	17.71	9.26	-0.97	2.14E-03	1.84E-01
Dck	7.20	11.28	0.62	2.19E-03	1.85E-01
Gm13304	22.63	11.92	-0.97	2.16E-03	1.85E-01
Gm21541	21.84	11.50	-0.97	2.18E-03	1.85E-01
BC052040	4.73	8.35	0.79	2.22E-03	1.86E-01
Fyb2	2.02	4.04	0.97	2.22E-03	1.86E-01
Tmem126a	39.54	28.67	-0.50	2.27E-03	1.88E-01
Krt20	0.39	1.42	1.84	2.28E-03	1.88E-01
Cox6c	88.81	119.05	0.39	2.30E-03	1.89E-01
Rpl4	196.12	257.13	0.36	2.32E-03	1.89E-01
Kdm6b	2.97	1.57	-0.96	2.36E-03	1.89E-01
Bcor	3.45	1.69	-1.07	2.37E-03	1.89E-01
Map10	1.36	0.54	-1.36	2.36E-03	1.89E-01
Gm13292	231.03	134.97	-0.81	2.38E-03	1.89E-01
A230005M16Rik	19.30	11.20	-0.83	2.34E-03	1.89E-01
Dctd	0.73	1.79	1.27	2.42E-03	1.91E-01
Arpc4	44.75	32.32	-0.50	2.41E-03	1.91E-01
Fgd6	0.67	0.27	-1.36	2.44E-03	1.91E-01
Acp5	0.36	0.02	-4.21	2.47E-03	1.92E-01
Tnfrsf13c	0.09	0.74	2.91	2.47E-03	1.92E-01
Tmcc2	6.17	4.20	-0.59	2.50E-03	1.93E-01
Nfasc	22.71	17.03	-0.45	2.52E-03	1.94E-01
Ndufa6	165.52	222.69	0.40	2.55E-03	1.95E-01
Shank1	100.40	61.87	-0.74	2.56E-03	1.95E-01
Slc9a1	9.40	6.57	-0.55	2.57E-03	1.95E-01
Fancg	1.25	0.46	-1.46	2.61E-03	1.96E-01
Syt9	2.22	1.14	-1.00	2.62E-03	1.96E-01

Atn1	54.02	37.06	-0.58	2.65E-03	1.96E-01
L1cam	20.11	15.43	-0.42	2.65E-03	1.96E-01
Tlcd1	26.22	35.00	0.38	2.66E-03	1.97E-01
Palm	9.85	6.55	-0.62	2.72E-03	2.00E-01
Nwd1	0.43	0.17	-1.39	2.77E-03	2.02E-01
Mon1a	3.48	1.79	-0.99	2.86E-03	2.05E-01
Hic2	2.28	0.31	-2.88	2.87E-03	2.05E-01
Snrpa	37.35	28.34	-0.43	2.86E-03	2.05E-01
Celsr2	20.79	14.98	-0.51	2.87E-03	2.05E-01
Gm16409	109.92	152.69	0.45	2.84E-03	2.05E-01
Fkbp3	375.31	490.43	0.36	2.89E-03	2.06E-01
Selenom	31.54	22.53	-0.52	2.91E-03	2.06E-01
Fam216a	66.96	88.91	0.38	2.96E-03	2.06E-01
Tecr	388.86	286.08	-0.48	2.95E-03	2.06E-01
Rnf144b	0.58	1.42	1.26	2.97E-03	2.06E-01
4731419I09Rik	0.66	0.19	-1.86	2.96E-03	2.06E-01
Usp54	3.16	1.99	-0.70	3.01E-03	2.09E-01
Aim2	0.21	0.02	-3.15	3.08E-03	2.12E-01
Nsmce4a	10.43	15.40	0.53	3.11E-03	2.13E-01
Gm11263	129.84	179.34	0.44	3.12E-03	2.13E-01
Nfs1	10.07	7.31	-0.49	3.17E-03	2.15E-01
Gal3st3	87.76	62.86	-0.52	3.21E-03	2.15E-01
Sec61b	38.10	55.83	0.52	3.19E-03	2.15E-01
Bbip1	44.18	58.71	0.38	3.21E-03	2.15E-01
Tet3	2.67	1.64	-0.75	3.27E-03	2.19E-01
AF529169	0.29	0.03	-3.16	3.31E-03	2.19E-01
Bahcc1	1.92	0.85	-1.23	3.31E-03	2.19E-01
Csf1	1.35	0.64	-1.10	3.41E-03	2.20E-01
Grk2	10.95	8.26	-0.44	3.39E-03	2.20E-01
Gm20605	2.99	1.74	-0.82	3.41E-03	2.20E-01
Urb1	0.67	0.27	-1.36	3.36E-03	2.20E-01
Fam204a	22.87	16.50	-0.50	3.35E-03	2.20E-01
Gm21986	27.00	18.86	-0.56	3.41E-03	2.20E-01
Cep41	1.31	0.59	-1.18	3.51E-03	2.25E-01
Syt3	13.26	8.89	-0.62	3.56E-03	2.28E-01
Tomm7	57.23	78.70	0.43	3.57E-03	2.28E-01
Apc2	2.42	1.53	-0.70	3.62E-03	2.29E-01
Cobll1	0.21	0.60	1.46	3.61E-03	2.29E-01
Chchd2-ps	84.49	119.37	0.47	3.67E-03	2.31E-01
9130019P16Rik	1.60	0.72	-1.19	3.71E-03	2.33E-01
Pcdh1	39.78	30.15	-0.44	3.73E-03	2.33E-01
Mpp2	31.11	22.95	-0.48	3.76E-03	2.34E-01
Rps27l	25.95	40.23	0.60	3.77E-03	2.34E-01
Nsmf	39.96	31.83	-0.36	3.81E-03	2.34E-01
Psmb1	89.65	120.94	0.40	3.80E-03	2.34E-01
Cox6c2	372.58	503.26	0.40	3.84E-03	2.35E-01
Porcn	16.84	12.61	-0.45	3.90E-03	2.37E-01
Gm5577	154.17	122.40	-0.37	3.91E-03	2.37E-01
Dcp1b	2.10	3.25	0.60	3.93E-03	2.38E-01
Cavin2	0.30	0.90	1.58	3.96E-03	2.39E-01
Cpne9	2.11	1.23	-0.81	4.05E-03	2.41E-01
Slc24a3	47.25	34.84	-0.48	4.02E-03	2.41E-01

Umad1	12.34	16.40	0.38	4.04E-03	2.41E-01
Brms1l	26.54	35.56	0.39	4.08E-03	2.42E-01
Atp9a	9.88	7.15	-0.50	4.17E-03	2.46E-01
Islr2	2.52	1.39	-0.90	4.16E-03	2.46E-01
NA	7.67	2.73	-1.53	4.25E-03	2.49E-01
Atf7	6.43	4.49	-0.55	4.24E-03	2.49E-01
Zcrb1	28.89	38.30	0.37	4.30E-03	2.49E-01
Tada3	8.37	5.91	-0.54	4.29E-03	2.49E-01
NA	111.04	151.63	0.42	4.28E-03	2.49E-01
Eno2	86.84	64.12	-0.48	4.34E-03	2.50E-01
Tbl2	3.27	2.06	-0.70	4.45E-03	2.52E-01
Arl8a	64.03	45.27	-0.54	4.42E-03	2.52E-01
Ulk1	19.36	14.38	-0.47	4.47E-03	2.52E-01
Gapdh	533.28	412.93	-0.40	4.46E-03	2.52E-01
Sarnp	30.48	43.41	0.48	4.40E-03	2.52E-01
Gm13340	19.11	11.76	-0.73	4.43E-03	2.52E-01
Gcn1l1	0.60	0.35	-0.82	4.54E-03	2.55E-01
Capn1	4.01	2.18	-0.92	4.61E-03	2.55E-01
Ndufaf5	32.81	44.84	0.42	4.64E-03	2.55E-01
Slitrk5	26.21	19.75	-0.44	4.63E-03	2.55E-01
Gm10123	1770.47	2233.10	0.30	4.63E-03	2.55E-01
Gm5859	9.26	5.87	-0.70	4.66E-03	2.55E-01
Rps6-ps3	133.72	182.03	0.42	4.67E-03	2.55E-01
Gm20417	55.13	30.33	-0.89	4.59E-03	2.55E-01
Mobp	1.32	0.65	-1.06	4.69E-03	2.56E-01
Clcn7	1.81	0.84	-1.15	4.75E-03	2.58E-01
Gabra3	10.31	7.52	-0.49	4.77E-03	2.58E-01
Sox11	0.76	0.13	-2.53	4.80E-03	2.59E-01
Plp1	2.17	1.30	-0.78	4.85E-03	2.59E-01
Gpr171	0.23	0.00	-5.74	4.84E-03	2.59E-01
Gm6563	29.24	42.06	0.49	4.85E-03	2.59E-01
Chchd7	2.83	4.80	0.74	4.94E-03	2.62E-01
Mdga1	4.20	2.31	-0.91	4.95E-03	2.62E-01
Cend1	164.68	125.34	-0.43	4.96E-03	2.62E-01
Mef2c	14.37	20.86	0.51	5.06E-03	2.63E-01
Cpne6	68.84	54.81	-0.36	5.07E-03	2.63E-01
Msrb2	24.12	34.83	0.50	5.01E-03	2.63E-01
Fam234b	14.26	10.46	-0.48	5.12E-03	2.63E-01
Cdk16	28.84	22.46	-0.39	5.13E-03	2.63E-01
Dph6	7.45	10.70	0.49	5.12E-03	2.63E-01
mt-Nd4	143.59	98.35	-0.57	5.02E-03	2.63E-01
Gm10284	3.14	0.84	-1.93	5.11E-03	2.63E-01
NA	9.06	14.20	0.62	5.14E-03	2.63E-01
Pabpc1l2a-ps	19.14	10.32	-0.93	5.16E-03	2.64E-01
Uqcrb	137.76	182.89	0.38	5.19E-03	2.64E-01
Gm6180	62.06	44.12	-0.53	5.22E-03	2.65E-01
Pld3	82.95	63.29	-0.43	5.24E-03	2.65E-01
Necab1	22.77	31.59	0.44	5.33E-03	2.69E-01
Map3k15	14.24	19.28	0.41	5.42E-03	2.72E-01
Ubc	154.72	117.70	-0.42	5.46E-03	2.73E-01
Ap4s1	60.83	84.98	0.45	5.48E-03	2.73E-01
Ccl21a	13.08	6.82	-0.98	5.48E-03	2.73E-01

Lxn	29.93	42.24	0.47	5.53E-03	2.74E-01
Grm1	7.93	5.50	-0.56	5.58E-03	2.75E-01
Pde4a	20.07	15.13	-0.44	5.57E-03	2.75E-01
Vps72	2.21	1.20	-0.92	5.75E-03	2.77E-01
Rbfox2	23.65	18.19	-0.42	5.68E-03	2.77E-01
Fchsd1	7.05	5.20	-0.47	5.73E-03	2.77E-01
Ldlrad3	0.94	0.42	-1.20	5.77E-03	2.77E-01
Gm7536	188.25	249.47	0.37	5.79E-03	2.77E-01
Rnf169	5.75	8.33	0.50	5.79E-03	2.77E-01
Gm15564	552.14	220.12	-1.37	5.76E-03	2.77E-01
Gm25911	13793.09	9864.07	-0.51	5.66E-03	2.77E-01
5830462I19Rik	2.37	1.36	-0.83	5.77E-03	2.77E-01
Il17rd	0.46	0.15	-1.61	5.81E-03	2.78E-01
Gm10275	41.34	58.60	0.47	5.87E-03	2.79E-01
Mef2d	46.04	33.69	-0.49	5.89E-03	2.79E-01
Cfap36	54.24	71.44	0.37	5.92E-03	2.79E-01
Ddn	121.03	86.96	-0.52	5.93E-03	2.79E-01
Znrf3	3.88	2.74	-0.53	6.00E-03	2.81E-01
Gm13301	9.54	6.22	-0.66	5.98E-03	2.81E-01
Dynll1	427.84	535.85	0.29	6.11E-03	2.84E-01
Urah	0.63	1.88	1.53	6.10E-03	2.84E-01
Itga6	0.27	0.09	-1.59	6.15E-03	2.85E-01
Amy1	1.10	0.30	-1.91	6.13E-03	2.85E-01
Gm26560	2.28	0.94	-1.32	6.22E-03	2.87E-01
Eif3f	25.23	33.87	0.39	6.29E-03	2.90E-01
Arl4d	0.79	2.63	1.70	6.40E-03	2.90E-01
Abhd14b	2.98	1.38	-1.14	6.36E-03	2.90E-01
Gm6863	78.11	103.57	0.37	6.40E-03	2.90E-01
Gm6851	27.15	41.74	0.59	6.39E-03	2.90E-01
Wdr83os	84.74	60.00	-0.54	6.35E-03	2.90E-01
Gm960	0.38	0.08	-2.23	6.41E-03	2.90E-01
Btbd17	1.60	0.83	-0.98	6.46E-03	2.91E-01
Gm20499	46.00	26.26	-0.84	6.56E-03	2.95E-01
Ccdc97	9.05	5.88	-0.66	6.65E-03	2.97E-01
Ddx47	6.39	9.62	0.56	6.90E-03	2.97E-01
Uqcrfs1	67.46	93.86	0.45	6.72E-03	2.97E-01
Btaf1	1.54	2.53	0.68	6.85E-03	2.97E-01
Dennd6a	9.05	6.66	-0.48	6.92E-03	2.97E-01
Hsd3b7	3.98	2.54	-0.69	6.84E-03	2.97E-01
Shld1	2.66	0.86	-1.68	6.91E-03	2.97E-01
Gprin3	3.99	2.20	-0.90	6.82E-03	2.97E-01
Atxn7l2	1.07	2.24	1.04	6.86E-03	2.97E-01
Rpp38	13.98	7.51	-0.93	6.88E-03	2.97E-01
Vsnl1	5704.63	6837.99	0.23	6.75E-03	2.97E-01
Gm17494	492.65	621.06	0.30	6.82E-03	2.97E-01
Mrpl18	24.03	34.17	0.48	6.70E-03	2.97E-01
Zfp993	5.58	2.69	-1.09	6.74E-03	2.97E-01
Rps6-ps4	219.95	288.64	0.36	6.83E-03	2.97E-01
Gm17167	6.69	4.33	-0.67	6.81E-03	2.97E-01
Gm38393	438.05	356.33	-0.33	6.96E-03	2.97E-01
Pnpla1	0.15	0.01	-3.89	6.95E-03	2.97E-01
Tab1	2.64	1.34	-1.02	7.06E-03	2.99E-01

Ly6h	3.79	2.29	-0.77	7.09E-03	2.99E-01
Prr5	0.52	0.08	-2.78	7.13E-03	2.99E-01
Kcnb1	14.02	8.81	-0.71	7.04E-03	2.99E-01
Gm12715	238.42	311.92	0.36	7.08E-03	2.99E-01
Actg1	221.49	286.94	0.35	7.12E-03	2.99E-01
Tiam2	2.70	4.13	0.58	7.20E-03	3.02E-01
Med13l	8.99	6.57	-0.49	7.31E-03	3.05E-01
Rps20	19.72	26.38	0.39	7.34E-03	3.06E-01
Capn15	1.48	0.56	-1.47	7.36E-03	3.06E-01
Tpmt	1.41	0.56	-1.37	7.39E-03	3.06E-01
Tk2	4.66	7.14	0.58	7.41E-03	3.06E-01
Gm20498	22.90	30.36	0.37	7.47E-03	3.07E-01
Ccl28	1.72	0.73	-1.27	7.48E-03	3.07E-01
Gm8172	98.95	133.24	0.40	7.47E-03	3.07E-01
Ufc1	28.30	37.78	0.39	7.50E-03	3.07E-01
Mark2	8.29	6.16	-0.46	7.52E-03	3.07E-01
Xlr3b	0.26	0.80	1.62	7.55E-03	3.07E-01
Plxna4	20.15	15.66	-0.40	7.59E-03	3.08E-01
Gm1821	417.23	333.98	-0.36	7.65E-03	3.09E-01
Gm4995	41.97	61.35	0.52	7.65E-03	3.09E-01
Gm14494	64.71	92.76	0.49	7.71E-03	3.10E-01
Rpl23a	188.67	243.44	0.34	7.76E-03	3.11E-01
Gm13302	5.90	3.62	-0.74	7.78E-03	3.11E-01
Afap1	2.21	1.17	-0.97	7.84E-03	3.13E-01
NA	15.67	9.62	-0.74	7.85E-03	3.13E-01
Prkn	2.34	0.53	-2.18	7.95E-03	3.15E-01
Sbf1	7.03	5.16	-0.49	7.95E-03	3.15E-01
Angel1	0.94	0.29	-1.77	8.10E-03	3.18E-01
Rassf3	1.52	2.78	0.84	8.08E-03	3.18E-01
Slc5a2	1.71	0.75	-1.23	8.08E-03	3.18E-01
Ubb	331.69	269.93	-0.33	8.17E-03	3.19E-01
Rangrf	6.49	10.79	0.70	8.15E-03	3.19E-01
Adam11	5.08	3.66	-0.51	8.21E-03	3.20E-01
Pxmp4	5.91	4.04	-0.58	8.35E-03	3.21E-01
Dyrk1b	3.06	1.78	-0.81	8.35E-03	3.21E-01
Lsm2	6.38	4.38	-0.57	8.34E-03	3.21E-01
Gm12918	164.52	213.47	0.34	8.33E-03	3.21E-01
Htra2	4.40	2.77	-0.70	8.28E-03	3.21E-01
A330102I10Rik	1.56	0.60	-1.43	8.28E-03	3.21E-01
Rusc2	13.00	9.86	-0.43	8.38E-03	3.21E-01
Gm8213	69.48	94.95	0.42	8.40E-03	3.21E-01
Smc5	3.25	4.77	0.52	8.47E-03	3.22E-01
Ppp1r13l	0.59	0.14	-2.14	8.46E-03	3.22E-01
Thy1	391.08	299.70	-0.42	8.49E-03	3.22E-01
Gpr108	2.42	1.12	-1.15	8.53E-03	3.23E-01
Scrt2	6.10	3.93	-0.67	8.55E-03	3.23E-01
Shisa7	43.60	33.34	-0.42	8.62E-03	3.25E-01
Sema6a	1.58	0.97	-0.75	8.70E-03	3.26E-01
Ptms	69.88	44.40	-0.70	8.70E-03	3.26E-01
Iqsec2	16.74	11.75	-0.55	8.72E-03	3.26E-01
Dagla	16.84	12.81	-0.43	8.77E-03	3.27E-01
Slco2a1	2.02	1.10	-0.92	8.84E-03	3.28E-01

NA	10.08	6.29	-0.72	8.84E-03	3.28E-01
Npcd	7.01	4.44	-0.69	8.89E-03	3.29E-01
Prkcg	413.79	331.70	-0.36	8.91E-03	3.29E-01
Adcy5	7.65	5.88	-0.41	9.13E-03	3.31E-01
Csmd2	1.45	0.93	-0.68	9.14E-03	3.31E-01
C2cd2l	16.77	13.19	-0.38	9.10E-03	3.31E-01
Zfp384	5.02	3.62	-0.51	9.08E-03	3.31E-01
Cc2d2a	1.55	2.51	0.67	9.14E-03	3.31E-01
Rps6-ps1	78.25	106.22	0.41	9.01E-03	3.31E-01
Nt5dc2	4.88	2.36	-1.08	9.11E-03	3.31E-01
Gm10704	22.20	32.11	0.50	9.05E-03	3.31E-01
Cdr1	424.49	299.57	-0.54	9.12E-03	3.31E-01
Rpl13-ps3	111.91	145.80	0.35	9.19E-03	3.32E-01
Tmem63b	13.29	10.31	-0.40	9.30E-03	3.35E-01
Lrrc4b	12.46	8.63	-0.57	9.33E-03	3.35E-01
Tedc2	1.58	3.03	0.90	9.40E-03	3.35E-01
Arhgdia	81.39	65.05	-0.36	9.42E-03	3.35E-01
Rps6	148.43	191.34	0.34	9.41E-03	3.35E-01
Tmem164	0.11	0.46	1.98	9.41E-03	3.35E-01
Zfhx2	6.28	4.29	-0.59	9.45E-03	3.35E-01
Slc35c2	2.07	1.19	-0.84	9.55E-03	3.38E-01
Cybrd1	0.60	0.14	-2.17	9.61E-03	3.38E-01
Creb3l1	2.23	1.07	-1.10	9.59E-03	3.38E-01
Stard7	14.06	18.94	0.40	9.62E-03	3.38E-01
Alg8	5.41	3.43	-0.69	9.68E-03	3.39E-01
Zfp991	2.85	1.60	-0.87	9.67E-03	3.39E-01
Ccdc159	0.51	1.61	1.63	9.79E-03	3.41E-01
Erg28	8.54	6.32	-0.47	9.76E-03	3.41E-01
Crim1	21.62	16.52	-0.43	9.79E-03	3.41E-01
Ltb	1.44	0.03	-5.66	9.89E-03	3.43E-01
Capn5	10.76	8.21	-0.42	9.97E-03	3.44E-01
Hist1h2bk	10.61	5.52	-0.98	9.95E-03	3.44E-01
Ap1s1	14.55	10.57	-0.50	1.01E-02	3.46E-01
Yeats2	3.88	2.52	-0.65	1.01E-02	3.46E-01
Oaz1-ps	122.80	93.19	-0.43	1.01E-02	3.46E-01
Tpt1	178.33	227.27	0.32	1.01E-02	3.46E-01
Per1	7.47	5.20	-0.56	1.02E-02	3.46E-01
Snap23	1.55	0.55	-1.52	1.02E-02	3.46E-01
Nova2	32.34	24.72	-0.43	1.02E-02	3.46E-01
2310002F09Rik	4.69	7.57	0.66	1.01E-02	3.46E-01
Gm26811	6.24	4.63	-0.46	1.02E-02	3.46E-01
Cry1	1.93	3.30	0.74	1.03E-02	3.46E-01
Mrps35	1.98	3.09	0.60	1.03E-02	3.46E-01
Rpl23a-ps3	170.22	220.58	0.34	1.03E-02	3.46E-01
Gm3362	147.39	192.68	0.36	1.03E-02	3.46E-01
Zcwpw1	0.39	1.17	1.55	1.04E-02	3.48E-01
Zfp141	5.08	7.37	0.51	1.04E-02	3.48E-01
Rnasek	362.49	286.90	-0.37	1.04E-02	3.48E-01
Smo	2.24	0.98	-1.22	1.05E-02	3.48E-01
Ccna2	5.34	7.73	0.50	1.05E-02	3.48E-01
B3gat3	8.28	5.10	-0.74	1.05E-02	3.48E-01
Olfr31	0.11	1.00	3.13	1.06E-02	3.48E-01

Gm16581	13.72	9.02	-0.64	1.05E-02	3.48E-01
Ankrd52	8.29	6.36	-0.42	1.06E-02	3.49E-01
Ncdn	121.29	96.31	-0.37	1.08E-02	3.52E-01
Ppp1r9b	39.99	30.79	-0.41	1.08E-02	3.52E-01
Serpina3f	0.31	0.02	-3.78	1.08E-02	3.52E-01
Mir686	25.24	11.22	-1.21	1.08E-02	3.52E-01
Gm16278	4.35	2.47	-0.84	1.08E-02	3.52E-01
Tppp3	3.24	1.79	-0.88	1.09E-02	3.53E-01
Vit	1.10	2.35	1.06	1.09E-02	3.53E-01
AI593442	127.08	104.11	-0.32	1.09E-02	3.53E-01
Gm42418	11789.22	7128.49	-0.77	1.10E-02	3.55E-01
Ptbp1	0.79	0.39	-1.07	1.10E-02	3.55E-01
Pip4k2b	27.23	21.95	-0.35	1.11E-02	3.55E-01
Hpcal4	242.85	207.01	-0.26	1.11E-02	3.55E-01
Sox12	3.13	1.22	-1.42	1.11E-02	3.55E-01
Gas5	23.64	30.85	0.35	1.11E-02	3.55E-01
D630044L22Rik	1.24	0.46	-1.49	1.11E-02	3.55E-01
Sdf2l1	7.06	3.39	-1.10	1.12E-02	3.55E-01
Igsf8	16.39	13.11	-0.36	1.13E-02	3.57E-01
Syngap1	82.04	60.94	-0.47	1.13E-02	3.57E-01
Camk2a	1000.71	825.02	-0.32	1.14E-02	3.58E-01
Coprs	16.29	24.12	0.53	1.14E-02	3.58E-01
1110002L01Rik	13.32	17.43	0.36	1.14E-02	3.58E-01
3110070M22Rik	1.30	0.15	-3.15	1.14E-02	3.58E-01
Pabpc5	2.54	1.15	-1.19	1.14E-02	3.59E-01
Preb	21.13	5.73	-1.96	1.15E-02	3.59E-01
Gm6682	232.57	181.43	-0.39	1.17E-02	3.64E-01
Cbx6	13.71	10.87	-0.37	1.17E-02	3.64E-01
Rps13	243.73	311.99	0.32	1.18E-02	3.66E-01
Hars	30.90	40.19	0.35	1.19E-02	3.67E-01
Rnaseh1	2.26	4.27	0.88	1.19E-02	3.67E-01
Psmg2	10.05	15.92	0.64	1.18E-02	3.67E-01
Etfa	14.26	21.22	0.54	1.19E-02	3.67E-01
2310058D17Rik	0.28	0.91	1.68	1.18E-02	3.67E-01
1500002C15Rik	22.33	29.60	0.37	1.20E-02	3.70E-01
Gm21988	202.76	161.47	-0.36	1.21E-02	3.71E-01
Gm10600	7.12	4.29	-0.77	1.22E-02	3.72E-01
Nova1	8.93	11.88	0.38	1.23E-02	3.73E-01
Mast3	71.17	55.21	-0.40	1.22E-02	3.73E-01
Dctn1	10.08	7.98	-0.37	1.23E-02	3.73E-01
Ccdc106	2.98	1.54	-0.99	1.22E-02	3.73E-01
Ppia	468.11	571.98	0.26	1.23E-02	3.73E-01
H3f3a-ps2	43.34	30.43	-0.54	1.23E-02	3.73E-01
A530032D15Rik	2.24	1.08	-1.09	1.23E-02	3.73E-01
Csnk1g2	10.76	7.88	-0.48	1.25E-02	3.73E-01
Pacs2	7.62	5.76	-0.44	1.25E-02	3.73E-01
Caly	30.87	23.30	-0.44	1.24E-02	3.73E-01
Atp1a1	32.07	26.07	-0.33	1.25E-02	3.73E-01
Blvrb	1.46	0.51	-1.57	1.24E-02	3.73E-01
Zfp592	4.34	2.98	-0.58	1.30E-02	3.73E-01
Atxn10	112.07	142.46	0.32	1.29E-02	3.73E-01
Agap2	45.48	35.38	-0.40	1.30E-02	3.73E-01

Eif4e2	5.27	3.73	-0.54	1.25E-02	3.73E-01
Fam110a	0.97	0.41	-1.27	1.28E-02	3.73E-01
Toe1	1.46	2.61	0.81	1.29E-02	3.73E-01
Cnbp	153.01	199.17	0.35	1.27E-02	3.73E-01
Mzf1	0.70	0.30	-1.24	1.30E-02	3.73E-01
Dcun1d5	42.16	57.15	0.41	1.27E-02	3.73E-01
Tstd2	3.22	4.57	0.47	1.26E-02	3.73E-01
Megf11	5.15	6.98	0.41	1.30E-02	3.73E-01
Sema6c	0.52	0.17	-1.69	1.26E-02	3.73E-01
Ap5z1	1.49	0.79	-0.94	1.29E-02	3.73E-01
Cdk6	0.28	0.05	-2.61	1.26E-02	3.73E-01
Cdc42ep4	1.79	1.01	-0.86	1.28E-02	3.73E-01
Wscd2	3.07	2.34	-0.43	1.25E-02	3.73E-01
Gm24270	36757.47	27302.25	-0.46	1.30E-02	3.73E-01
Ighm	27.14	14.95	-0.90	1.29E-02	3.73E-01
Pisd-ps1	4.38	2.28	-0.98	1.28E-02	3.73E-01
Gm13296	171.28	101.02	-0.80	1.30E-02	3.73E-01
A330023F24Rik	0.09	0.26	1.45	1.29E-02	3.73E-01
Gm26794	12.07	8.91	-0.47	1.27E-02	3.73E-01
Sf3a2	3.84	2.01	-0.98	1.31E-02	3.74E-01
Ciz1	5.85	4.16	-0.53	1.31E-02	3.74E-01
Anks3	2.54	1.44	-0.85	1.32E-02	3.74E-01
Prss23os	0.28	1.56	2.50	1.31E-02	3.74E-01
Cfap58	0.20	0.64	1.65	1.32E-02	3.74E-01
Nol7	32.05	41.89	0.35	1.32E-02	3.74E-01
Psmd12	29.26	38.68	0.37	1.33E-02	3.75E-01
Dgkz	53.76	38.42	-0.53	1.33E-02	3.75E-01
Zmiz2	26.51	19.38	-0.49	1.33E-02	3.75E-01
Smim26	65.09	87.36	0.39	1.33E-02	3.75E-01
Twsg1	8.87	6.68	-0.44	1.35E-02	3.75E-01
Adgrb2	7.77	5.57	-0.52	1.35E-02	3.75E-01
Lrrtm1	28.49	22.14	-0.40	1.34E-02	3.75E-01
Vps25	19.82	14.33	-0.50	1.34E-02	3.75E-01
Plcxd2	4.93	7.39	0.56	1.35E-02	3.75E-01
Mlec	13.45	10.30	-0.42	1.35E-02	3.76E-01
Gm17168	118.80	83.90	-0.54	1.35E-02	3.76E-01
Hadha	2.85	1.90	-0.62	1.36E-02	3.78E-01
Cox17	7.38	9.69	0.36	1.36E-02	3.78E-01
Por	10.27	7.68	-0.45	1.37E-02	3.78E-01
Chkb	1.16	0.64	-0.89	1.38E-02	3.78E-01
Pcna	23.43	30.82	0.36	1.37E-02	3.78E-01
Gm7353	2.32	1.18	-1.03	1.37E-02	3.78E-01
NA	29.55	23.54	-0.36	1.38E-02	3.78E-01
Atp5g3	292.22	362.12	0.28	1.38E-02	3.78E-01
Tmem59l	43.17	32.17	-0.46	1.39E-02	3.80E-01
Rpa3	9.61	18.40	0.89	1.41E-02	3.81E-01
Id2	22.09	30.63	0.44	1.40E-02	3.81E-01
Zbtb1	1.60	2.27	0.48	1.41E-02	3.81E-01
R3hdm4	238.90	185.14	-0.41	1.40E-02	3.81E-01
Rps13-ps2	107.11	140.53	0.36	1.41E-02	3.81E-01
BC024978	16.62	12.69	-0.43	1.40E-02	3.81E-01
Gm14044	81.44	109.09	0.39	1.42E-02	3.83E-01

Acsl1	3.56	2.69	-0.44	1.42E-02	3.84E-01
Tob1	19.97	15.72	-0.38	1.44E-02	3.86E-01
Myo16	3.06	2.06	-0.61	1.45E-02	3.86E-01
Micu3	40.40	51.84	0.33	1.44E-02	3.86E-01
Rapgef1	6.42	4.87	-0.43	1.45E-02	3.86E-01
Zfp438	2.51	1.35	-0.94	1.45E-02	3.86E-01
E030024N20Rik	134.37	174.40	0.35	1.44E-02	3.86E-01
Tyw5	10.77	14.49	0.40	1.45E-02	3.87E-01
Gm13623	39.86	54.43	0.42	1.46E-02	3.88E-01
Rpl9-ps4	60.52	81.29	0.40	1.46E-02	3.88E-01
Nptxr	84.55	70.20	-0.30	1.46E-02	3.88E-01
Slc44a5	3.26	2.26	-0.56	1.47E-02	3.90E-01
Stk32c	3.10	1.36	-1.25	1.49E-02	3.91E-01
3-Sep	167.70	138.61	-0.31	1.49E-02	3.91E-01
Sdhaf2	14.27	18.57	0.35	1.49E-02	3.91E-01
Slc15a3	0.74	0.39	-0.97	1.50E-02	3.91E-01
Pfn2	163.78	203.88	0.28	1.48E-02	3.91E-01
Rcor3	8.69	6.49	-0.45	1.50E-02	3.91E-01
Clip2	3.85	2.47	-0.69	1.50E-02	3.91E-01
1190007I07Rik	7.77	12.47	0.65	1.49E-02	3.91E-01
Arfgef2	5.00	3.69	-0.47	1.49E-02	3.91E-01
Kcnn3	1.91	1.05	-0.89	1.56E-02	3.91E-01
Ramp2	15.68	12.07	-0.41	1.55E-02	3.91E-01
Gmpr2	3.78	6.42	0.74	1.55E-02	3.91E-01
Prkd1	0.43	1.03	1.24	1.58E-02	3.91E-01
Hmox1	1.93	1.00	-0.99	1.51E-02	3.91E-01
Mpped2	18.64	25.75	0.44	1.65E-02	3.91E-01
Mccc2	2.14	4.01	0.87	1.57E-02	3.91E-01
Ifngr2	27.79	22.52	-0.34	1.63E-02	3.91E-01
Ptk7	0.19	0.02	-3.09	1.62E-02	3.91E-01
Syt7	17.10	13.49	-0.38	1.53E-02	3.91E-01
Adpgk	0.79	1.65	1.04	1.65E-02	3.91E-01
Baiap2	32.23	25.60	-0.37	1.54E-02	3.91E-01
Mbd6	1.32	0.71	-0.94	1.62E-02	3.91E-01
Usf1	3.04	1.68	-0.90	1.60E-02	3.91E-01
Tspan18	7.10	4.29	-0.75	1.60E-02	3.91E-01
Psma7	29.36	37.91	0.34	1.64E-02	3.91E-01
Rchy1	16.57	22.03	0.38	1.61E-02	3.91E-01
Ajm1	9.10	6.69	-0.48	1.63E-02	3.91E-01
Auts2	7.16	5.36	-0.45	1.59E-02	3.91E-01
Plxna1	13.56	10.15	-0.46	1.54E-02	3.91E-01
Sipa1l3	12.81	9.66	-0.45	1.57E-02	3.91E-01
Nox1	1.08	1.95	0.82	1.56E-02	3.91E-01
Mak16	12.06	17.77	0.53	1.54E-02	3.91E-01
Scrg1	2.25	5.14	1.16	1.60E-02	3.91E-01
Cfap20	21.51	31.82	0.54	1.53E-02	3.91E-01
Cd59a	3.64	1.63	-1.20	1.51E-02	3.91E-01
Hid1	19.81	15.66	-0.37	1.62E-02	3.91E-01
Cacna1a	8.78	6.86	-0.39	1.62E-02	3.91E-01
Ror1	0.82	0.39	-1.11	1.64E-02	3.91E-01
Cbarp	30.50	24.37	-0.36	1.56E-02	3.91E-01
Tmem255a	19.39	14.87	-0.42	1.64E-02	3.91E-01

Fbxw17	1.07	0.53	-1.05	1.51E-02	3.91E-01
Pak1ip1	8.51	11.44	0.39	1.63E-02	3.91E-01
Bud31	22.27	30.93	0.44	1.56E-02	3.91E-01
Zfp653	3.61	2.07	-0.84	1.52E-02	3.91E-01
Pcdhb12	1.27	0.62	-1.05	1.58E-02	3.91E-01
Rell2	12.18	9.31	-0.42	1.64E-02	3.91E-01
Zc3hav1l	5.81	3.98	-0.58	1.50E-02	3.91E-01
Slc39a9	4.96	3.68	-0.46	1.63E-02	3.91E-01
Gm7984	25.92	16.02	-0.73	1.65E-02	3.91E-01
Ccdc96	0.33	0.80	1.23	1.58E-02	3.91E-01
Pfdn4	14.88	19.77	0.38	1.53E-02	3.91E-01
D10Jhu81e	12.78	19.01	0.55	1.60E-02	3.91E-01
Hist3h2ba	5.96	2.87	-1.08	1.56E-02	3.91E-01
Tubb2a	238.10	294.90	0.28	1.54E-02	3.91E-01
Zfp932	6.43	9.03	0.46	1.58E-02	3.91E-01
Fjx1	7.02	4.75	-0.60	1.62E-02	3.91E-01
Zc3h3	2.83	1.46	-1.00	1.59E-02	3.91E-01
NA	11.07	7.75	-0.55	1.53E-02	3.91E-01
Gm11149	14.72	10.57	-0.51	1.64E-02	3.91E-01
Gm7206	63.62	88.46	0.45	1.52E-02	3.91E-01
Gm13610	14.22	9.46	-0.62	1.61E-02	3.91E-01
Neat1	0.11	0.02	-2.51	1.63E-02	3.91E-01
Gm20659	89.60	116.93	0.35	1.62E-02	3.91E-01
Gm20683	6.67	4.51	-0.60	1.60E-02	3.91E-01
Gm26770	1.80	3.13	0.76	1.58E-02	3.91E-01
Gm26702	9.31	6.84	-0.48	1.64E-02	3.91E-01
Dad1	15.94	12.43	-0.39	1.66E-02	3.91E-01
Mkks	10.95	8.38	-0.42	1.65E-02	3.91E-01
Higd1a	85.95	112.97	0.36	1.66E-02	3.91E-01
Tubb4a	612.30	525.33	-0.26	1.65E-02	3.91E-01
Rps13-ps4	94.84	124.10	0.36	1.66E-02	3.91E-01
Hltf	0.93	1.82	0.93	1.67E-02	3.91E-01
Pex7	10.98	15.16	0.43	1.67E-02	3.91E-01
Alg11	24.39	18.63	-0.42	1.66E-02	3.91E-01
Zgpat	1.36	0.76	-0.89	1.68E-02	3.94E-01
Snapc1	7.69	11.53	0.56	1.69E-02	3.94E-01
Anxa5	1.37	2.68	0.93	1.69E-02	3.94E-01
Mdh1	249.53	307.43	0.27	1.69E-02	3.95E-01
Jmjd8	16.29	12.51	-0.42	1.70E-02	3.95E-01
Tomm6os	11.40	8.10	-0.53	1.70E-02	3.95E-01
Lgmn	5.61	8.29	0.53	1.71E-02	3.95E-01
Wls	1.15	0.57	-1.04	1.71E-02	3.95E-01
Rfc1	3.52	2.29	-0.66	1.73E-02	4.00E-01
Kin	5.00	8.71	0.78	1.73E-02	4.00E-01
Cox14	87.55	117.96	0.40	1.74E-02	4.00E-01
Thyn1	57.26	75.93	0.37	1.74E-02	4.00E-01
Krcc1	4.20	8.55	1.00	1.74E-02	4.00E-01
Glmp	2.51	1.65	-0.64	1.77E-02	4.01E-01
Cpt1c	7.56	5.46	-0.50	1.77E-02	4.01E-01
Ppa1	34.23	43.34	0.31	1.77E-02	4.01E-01
Hcn2	1.13	0.53	-1.14	1.76E-02	4.01E-01
Tbc1d9	6.53	8.66	0.38	1.76E-02	4.01E-01

Afg3l1	2.42	0.52	-2.27	1.75E-02	4.01E-01
Gm49322	4.54	3.22	-0.53	1.78E-02	4.01E-01
Erf	4.35	2.29	-0.97	1.78E-02	4.01E-01
Pnma3	3.31	2.11	-0.69	1.76E-02	4.01E-01
Pcdh19	15.68	12.67	-0.34	1.77E-02	4.01E-01
Trim65	0.93	0.47	-1.04	1.78E-02	4.01E-01
Cbx3-ps7	88.40	117.22	0.38	1.77E-02	4.01E-01
Htatsf1	22.71	29.33	0.34	1.75E-02	4.01E-01
Ndufb6	161.14	208.82	0.34	1.78E-02	4.01E-01
Vps39	6.35	5.09	-0.35	1.78E-02	4.01E-01
Atp5g1	32.99	27.21	-0.31	1.79E-02	4.02E-01
Topbp1	3.91	5.63	0.50	1.80E-02	4.02E-01
Gm3892	9.78	7.07	-0.51	1.80E-02	4.02E-01
Tmem160	8.66	4.92	-0.86	1.81E-02	4.04E-01
Nup98	5.34	3.42	-0.68	1.81E-02	4.04E-01
Prpf40b	2.35	1.54	-0.64	1.82E-02	4.04E-01
Rps24	164.07	208.88	0.32	1.85E-02	4.04E-01
Brdt	4.11	6.11	0.54	1.85E-02	4.04E-01
Gabarapl2	55.69	70.04	0.30	1.85E-02	4.04E-01
Igsf9b	0.82	0.37	-1.18	1.84E-02	4.04E-01
Zic4	0.08	0.01	-3.34	1.85E-02	4.04E-01
Sstr4	26.60	18.48	-0.57	1.83E-02	4.04E-01
Nup88	3.78	5.31	0.46	1.84E-02	4.04E-01
Atf4	56.83	71.72	0.30	1.86E-02	4.04E-01
Bex2	277.41	226.55	-0.33	1.81E-02	4.04E-01
Rtn4rl1	50.35	41.35	-0.32	1.82E-02	4.04E-01
Rpl9	35.98	45.64	0.31	1.84E-02	4.04E-01
Fam174a	21.50	29.06	0.40	1.85E-02	4.04E-01
Cbx3-ps6	146.03	194.16	0.38	1.85E-02	4.04E-01
9230009I02Rik	0.30	1.57	2.40	1.83E-02	4.04E-01
Gm13768	12.98	8.59	-0.63	1.82E-02	4.04E-01
Gm20518	3.14	2.06	-0.65	1.84E-02	4.04E-01
Gm10593	9.60	6.96	-0.50	1.82E-02	4.04E-01
Elavl3	34.82	25.77	-0.47	1.87E-02	4.05E-01
Nifk	20.71	28.78	0.45	1.87E-02	4.05E-01
Gm2163	8.99	6.13	-0.59	1.87E-02	4.05E-01
Actr1b	45.85	37.70	-0.32	1.88E-02	4.07E-01
Fam166a	54.34	71.15	0.36	1.89E-02	4.07E-01
Lrguk	0.95	0.48	-1.00	1.89E-02	4.07E-01
Adam15	4.59	3.15	-0.58	1.89E-02	4.08E-01
Gm20900	111.36	143.59	0.34	1.90E-02	4.09E-01
Chd3	27.28	22.14	-0.34	1.92E-02	4.11E-01
Faim2	54.62	43.84	-0.35	1.91E-02	4.11E-01
Nlgn3	29.17	23.08	-0.38	1.93E-02	4.14E-01
Gm15464	71.94	96.30	0.39	1.93E-02	4.14E-01
Kif1a	85.28	73.19	-0.25	1.94E-02	4.15E-01
Ovol2	0.47	1.38	1.52	1.95E-02	4.15E-01
Dstyk	8.26	6.57	-0.36	1.95E-02	4.15E-01
Agtr1a	0.17	0.66	1.95	1.95E-02	4.15E-01
Ensa	265.98	334.65	0.30	1.96E-02	4.16E-01
Zfp532	4.19	3.24	-0.40	1.96E-02	4.16E-01
Cetn3	123.82	157.73	0.32	1.99E-02	4.18E-01

Mrps30	9.73	14.11	0.51	1.98E-02	4.18E-01
Cbx3	78.21	103.39	0.37	1.99E-02	4.18E-01
Creld1	3.87	2.61	-0.61	1.97E-02	4.18E-01
Sh2b1	5.79	3.81	-0.64	2.01E-02	4.18E-01
Rtn4r	22.38	16.93	-0.44	2.00E-02	4.18E-01
2810002D19Rik	1.24	2.95	1.24	1.99E-02	4.18E-01
Shisa6	101.27	84.15	-0.30	2.00E-02	4.18E-01
Cdh24	0.54	0.17	-1.68	1.98E-02	4.18E-01
Numbl	4.78	3.48	-0.50	2.01E-02	4.18E-01
Gm11196	27.21	40.11	0.53	1.99E-02	4.18E-01
Gm8624	30.08	41.59	0.44	1.98E-02	4.18E-01
Gm15710	105.86	135.14	0.32	1.99E-02	4.18E-01
Tpt1-ps3	280.29	355.33	0.31	2.00E-02	4.18E-01
Kitl	0.95	1.73	0.84	2.01E-02	4.18E-01
Nemf	14.78	20.02	0.41	2.02E-02	4.19E-01
Gm13215	23.48	35.20	0.56	2.02E-02	4.19E-01
Natd1	3.66	2.16	-0.80	2.05E-02	4.21E-01
Bsg	62.33	48.35	-0.40	2.04E-02	4.21E-01
Pcgf5	4.04	6.44	0.64	2.03E-02	4.21E-01
Tjp2	0.57	0.21	-1.46	2.05E-02	4.21E-01
Cops4	16.11	21.05	0.35	2.05E-02	4.21E-01
Naif1	0.83	1.49	0.80	2.05E-02	4.21E-01
Sgms1	3.19	2.43	-0.43	2.04E-02	4.21E-01
Rnf7	98.50	125.53	0.32	2.04E-02	4.21E-01
Cebpb	3.85	6.96	0.82	2.05E-02	4.21E-01
Iglon5	3.00	1.82	-0.76	2.06E-02	4.21E-01
Rpl9-ps6	55.96	73.92	0.37	2.06E-02	4.21E-01
Gm21958	4.87	2.39	-1.06	2.06E-02	4.21E-01
Mrps12	35.51	46.05	0.34	2.07E-02	4.22E-01
B4galnt1	35.79	28.18	-0.38	2.08E-02	4.22E-01
Hdac5	7.08	5.24	-0.47	2.09E-02	4.22E-01
Lta4h	4.24	6.90	0.68	2.09E-02	4.22E-01
Smarcd3	6.14	4.54	-0.47	2.08E-02	4.22E-01
Gm26995	1.89	0.82	-1.24	2.08E-02	4.22E-01
Pp2d1	4.88	7.34	0.56	2.09E-02	4.23E-01
Nap1l5	72.64	93.77	0.34	2.09E-02	4.23E-01
Gm11185	3.36	1.37	-1.32	2.10E-02	4.23E-01
Klc2	26.41	19.55	-0.47	2.12E-02	4.24E-01
Cnih2	100.95	75.34	-0.46	2.13E-02	4.24E-01
Abca2	5.47	4.33	-0.37	2.13E-02	4.24E-01
Smim27	74.77	97.60	0.35	2.12E-02	4.24E-01
Nos1	11.62	8.98	-0.41	2.13E-02	4.24E-01
Zcchc7	1.26	1.92	0.58	2.13E-02	4.24E-01
Scaf1	3.51	2.40	-0.59	2.13E-02	4.24E-01
Mrps27	9.04	6.49	-0.51	2.13E-02	4.24E-01
Swsap1	5.44	3.38	-0.72	2.12E-02	4.24E-01
Camk2b	82.38	70.78	-0.25	2.12E-02	4.24E-01
Hist1h2bl	13.84	8.04	-0.82	2.13E-02	4.24E-01
Plekhg5	11.47	8.72	-0.43	2.15E-02	4.26E-01
Ppp2ca	65.88	80.51	0.26	2.16E-02	4.27E-01
Dcakd	16.48	11.13	-0.61	2.17E-02	4.27E-01
Naga	1.25	0.55	-1.20	2.16E-02	4.27E-01

Scd2	27.99	22.64	-0.34	2.18E-02	4.27E-01
Eef1b2	36.91	47.31	0.33	2.16E-02	4.27E-01
Zw10	1.23	0.49	-1.36	2.18E-02	4.27E-01
Ccdc71	6.66	4.58	-0.57	2.17E-02	4.27E-01
Gm867	15.88	22.09	0.44	2.17E-02	4.27E-01
Cbln4	1.63	3.22	0.94	2.18E-02	4.27E-01
Nat6	1.95	1.15	-0.80	2.17E-02	4.27E-01
Tmem269	0.94	2.17	1.18	2.19E-02	4.28E-01
Ndufa4	1538.47	1891.41	0.27	2.19E-02	4.28E-01
Ap1s3	1.39	2.42	0.77	2.19E-02	4.28E-01
Tpd52	15.47	20.23	0.36	2.20E-02	4.28E-01
Glrx5	27.28	36.85	0.40	2.21E-02	4.29E-01
Sall2	11.02	8.79	-0.36	2.21E-02	4.29E-01
Rps13-ps1	84.78	109.81	0.34	2.21E-02	4.29E-01
Cul2	8.77	11.62	0.38	2.22E-02	4.30E-01
Pnkd	54.56	42.20	-0.41	2.22E-02	4.30E-01
Cspg4	0.53	0.26	-1.07	2.23E-02	4.31E-01
Rpl13	67.17	84.74	0.30	2.24E-02	4.32E-01
Man2b1	3.06	2.02	-0.64	2.24E-02	4.32E-01
Rpl9-ps7	54.50	72.07	0.37	2.24E-02	4.32E-01
Atpif1	207.46	258.13	0.28	2.24E-02	4.32E-01
Pdha1	31.15	39.67	0.32	2.26E-02	4.32E-01
Wnk2	9.47	7.11	-0.45	2.25E-02	4.32E-01
Arf3	467.19	360.87	-0.41	2.26E-02	4.32E-01
mt-Tl1	45.48	23.19	-1.00	2.25E-02	4.32E-01
Ctxn3	1.07	2.53	1.20	2.26E-02	4.32E-01
Gm11759	13.43	21.53	0.65	2.26E-02	4.32E-01
Rttn	0.29	0.11	-1.39	2.27E-02	4.34E-01
Hnrnpul1	31.47	24.64	-0.39	2.28E-02	4.36E-01
Gtpbp2	4.53	3.17	-0.56	2.31E-02	4.39E-01
Disp2	29.86	25.02	-0.29	2.31E-02	4.39E-01
Pgbd5	82.36	69.22	-0.28	2.31E-02	4.39E-01
Dync1h1	8.66	7.08	-0.33	2.32E-02	4.40E-01
Aip	18.74	13.78	-0.48	2.32E-02	4.40E-01
Cyp4v3	0.93	0.31	-1.67	2.33E-02	4.40E-01
Dlgap3	6.25	4.18	-0.62	2.33E-02	4.41E-01
Spin2c	7.29	12.44	0.75	2.34E-02	4.41E-01
Zfp277	15.05	20.04	0.38	2.34E-02	4.41E-01
Ankrd28	8.26	6.41	-0.40	2.38E-02	4.44E-01
Aif1	0.13	0.68	2.42	2.38E-02	4.44E-01
Slc25a15	1.13	2.43	1.08	2.37E-02	4.44E-01
Pygb	5.33	3.79	-0.53	2.37E-02	4.44E-01
Lgi3	6.15	4.42	-0.51	2.37E-02	4.44E-01
Cpne7	10.91	8.82	-0.34	2.37E-02	4.44E-01
Fam219aos	13.95	7.98	-0.85	2.39E-02	4.44E-01
A330070K13Rik	12.07	5.59	-1.15	2.37E-02	4.44E-01
Arntl	4.33	6.44	0.54	2.38E-02	4.44E-01
Znf41-ps	3.16	1.91	-0.76	2.39E-02	4.44E-01
Gm20441	13.88	8.98	-0.66	2.39E-02	4.44E-01
Gm3883	19.61	14.40	-0.48	2.37E-02	4.44E-01
Bcr	20.33	15.14	-0.47	2.39E-02	4.44E-01
Trmt11	0.42	1.08	1.34	2.42E-02	4.45E-01

Ldb3	0.36	0.78	1.10	2.42E-02	4.45E-01
Phf1	2.04	1.13	-0.88	2.42E-02	4.45E-01
Ctnnbl1	4.33	2.85	-0.64	2.41E-02	4.45E-01
Fam69a	39.03	50.88	0.35	2.41E-02	4.45E-01
Tiparp	1.38	2.33	0.72	2.41E-02	4.45E-01
Plxnb2	1.33	0.73	-0.91	2.41E-02	4.45E-01
Zfp574	6.99	4.97	-0.53	2.40E-02	4.45E-01
Tbca	223.69	282.17	0.31	2.43E-02	4.45E-01
Sf3b4	16.40	12.95	-0.37	2.42E-02	4.45E-01
Gm21987	75.99	98.84	0.35	2.43E-02	4.45E-01
Gm26805	2.46	1.41	-0.84	2.43E-02	4.45E-01
Setd4	0.42	0.17	-1.39	2.44E-02	4.46E-01
Scrt1	9.17	6.49	-0.54	2.46E-02	4.49E-01
Bex4	38.69	51.06	0.37	2.46E-02	4.49E-01
Tmem214	2.80	2.02	-0.50	2.48E-02	4.52E-01
Ccdc65	1.71	3.15	0.86	2.48E-02	4.52E-01
Tmem251	4.03	6.10	0.56	2.49E-02	4.53E-01
Ndufa12	108.80	135.54	0.29	2.50E-02	4.54E-01
Slc19a1	0.68	0.31	-1.18	2.53E-02	4.54E-01
Ezh1	5.30	4.22	-0.36	2.54E-02	4.54E-01
Atp6v0c	160.36	137.87	-0.25	2.53E-02	4.54E-01
Adh5	16.03	22.01	0.43	2.52E-02	4.54E-01
Celf5	39.63	30.99	-0.39	2.53E-02	4.54E-01
Prelp	0.40	0.10	-1.99	2.53E-02	4.54E-01
Hrk	5.31	3.73	-0.55	2.52E-02	4.54E-01
Polr1a	3.06	1.96	-0.69	2.54E-02	4.54E-01
Ppp1r1b	4.12	2.61	-0.70	2.52E-02	4.54E-01
Gm16229	0.77	1.81	1.19	2.51E-02	4.54E-01
Lime1	1.85	0.81	-1.24	2.52E-02	4.54E-01
Cwc22	5.52	3.18	-0.84	2.55E-02	4.56E-01
Grb10	3.38	4.66	0.44	2.56E-02	4.58E-01
Katnb1	4.78	3.26	-0.59	2.57E-02	4.58E-01
Gm21093	19.70	14.50	-0.48	2.57E-02	4.58E-01
Fbxo9	30.35	39.19	0.34	2.58E-02	4.59E-01
Arpc1a	52.16	64.88	0.28	2.59E-02	4.59E-01
Ndst1	7.36	5.77	-0.39	2.58E-02	4.59E-01
Vmac	1.35	0.66	-1.07	2.58E-02	4.59E-01
Eif3e	67.39	84.25	0.29	2.63E-02	4.60E-01
Ilkap	9.65	14.52	0.56	2.60E-02	4.60E-01
Gfm1	7.59	9.92	0.36	2.63E-02	4.60E-01
Rsrc1	1.53	2.10	0.43	2.61E-02	4.60E-01
Rammet	147.50	185.50	0.30	2.62E-02	4.60E-01
Ccdc74a	3.26	5.03	0.60	2.62E-02	4.60E-01
Slc35e2	5.00	3.80	-0.43	2.61E-02	4.60E-01
Ccdc42	0.63	0.14	-2.20	2.62E-02	4.60E-01
Blcap	17.09	13.72	-0.35	2.60E-02	4.60E-01
Mpc1-ps	148.30	200.80	0.41	2.61E-02	4.60E-01
Gm20503	52.86	69.71	0.37	2.62E-02	4.60E-01
Gm26733	2.93	1.80	-0.73	2.62E-02	4.60E-01
Prn	0.47	0.17	-1.50	2.63E-02	4.60E-01
Atp6v0a1	25.87	20.66	-0.36	2.64E-02	4.60E-01
Scfd1	8.39	11.78	0.46	2.64E-02	4.60E-01

Golga5	3.59	5.34	0.54	2.64E-02	4.60E-01
Arpc5l	20.06	25.21	0.30	2.65E-02	4.60E-01
Aktip	22.21	27.99	0.30	2.65E-02	4.60E-01
Samd10	3.22	1.94	-0.77	2.65E-02	4.60E-01
Dhcr7	4.28	3.09	-0.51	2.65E-02	4.60E-01
Cetn4	2.93	4.36	0.54	2.66E-02	4.61E-01
Atp2b3	35.47	28.66	-0.34	2.67E-02	4.62E-01
Hmgxb3	3.01	2.15	-0.52	2.68E-02	4.63E-01
Gm49405	0.32	0.10	-1.79	2.69E-02	4.64E-01
Prex1	2.67	1.85	-0.57	2.69E-02	4.64E-01
Seh1l	8.09	12.15	0.56	2.69E-02	4.64E-01
Pdcd2l	8.42	12.77	0.57	2.71E-02	4.65E-01
Zfp839	2.62	1.36	-0.99	2.71E-02	4.65E-01
Ptprn	58.58	48.87	-0.30	2.70E-02	4.65E-01
Cerkl	3.73	2.62	-0.55	2.71E-02	4.65E-01
Gm10335	137.95	174.45	0.31	2.72E-02	4.66E-01
Atg7	10.27	7.69	-0.45	2.72E-02	4.66E-01
Kdm4b	2.22	1.49	-0.61	2.73E-02	4.67E-01
Mrpl21	29.74	38.63	0.34	2.75E-02	4.69E-01
Kdelr2	13.48	9.84	-0.49	2.75E-02	4.69E-01
Klf10	9.74	13.72	0.46	2.76E-02	4.70E-01
Gm10132	132.61	167.55	0.31	2.76E-02	4.70E-01
4632411P08Rik	0.20	0.60	1.57	2.76E-02	4.70E-01
Ndufa2	221.32	284.01	0.33	2.77E-02	4.70E-01
D630045J12Rik	10.28	8.49	-0.31	2.77E-02	4.70E-01
Dele1	11.00	7.90	-0.51	2.78E-02	4.71E-01
Gm16061	3.51	0.98	-1.88	2.78E-02	4.71E-01
Galnt16	24.70	19.00	-0.42	2.79E-02	4.72E-01
Rilpl1	16.74	21.74	0.35	2.79E-02	4.72E-01
Rad51ap2	0.31	0.76	1.26	2.80E-02	4.72E-01
Nrxn2	2.73	2.08	-0.43	2.80E-02	4.72E-01
Dyrk2	7.70	5.25	-0.59	2.81E-02	4.72E-01
Frg2f1	6.61	4.68	-0.53	2.81E-02	4.73E-01
Pex6	3.47	2.33	-0.61	2.82E-02	4.74E-01
Atp5f1	66.99	86.15	0.34	2.84E-02	4.75E-01
Prpf8	80.05	66.10	-0.31	2.85E-02	4.75E-01
Dolpp1	0.78	0.36	-1.14	2.84E-02	4.75E-01
Ehd4	0.90	0.48	-0.93	2.84E-02	4.75E-01
Atpaf1	26.76	34.12	0.32	2.85E-02	4.75E-01
Gpc1	4.87	3.58	-0.48	2.85E-02	4.75E-01
Ctdspl	3.53	2.32	-0.64	2.84E-02	4.75E-01
Tusc1	12.60	7.86	-0.73	2.84E-02	4.75E-01
Adgrl1	42.14	35.91	-0.26	2.86E-02	4.76E-01
Copb2	29.99	38.14	0.32	2.86E-02	4.76E-01
Dcaf15	1.38	0.56	-1.33	2.87E-02	4.76E-01
Ap2a1	4.19	3.09	-0.47	2.87E-02	4.76E-01
Maz	7.01	4.89	-0.55	2.88E-02	4.77E-01
Lix1l	11.06	7.97	-0.51	2.88E-02	4.77E-01
Gm17786	60.05	80.50	0.39	2.88E-02	4.77E-01
Gng10	52.42	68.82	0.37	2.89E-02	4.77E-01
Cacng8	6.95	4.37	-0.71	2.89E-02	4.77E-01
Gm15772	79.76	103.02	0.34	2.89E-02	4.77E-01

Map9	29.21	36.08	0.27	2.91E-02	4.79E-01
Tuba1c	359.11	292.79	-0.33	2.91E-02	4.79E-01
Gm26377	988.63	1265.33	0.33	2.92E-02	4.80E-01
Gm12892	10.78	7.22	-0.62	2.92E-02	4.80E-01
Extl3	9.36	7.45	-0.37	2.93E-02	4.80E-01
Sik3	33.05	26.90	-0.33	2.93E-02	4.80E-01
Gabarap	72.21	58.07	-0.35	2.93E-02	4.80E-01
Plbd2	4.81	3.65	-0.43	2.94E-02	4.80E-01
Slc8a3	1.57	0.93	-0.80	2.95E-02	4.81E-01
Bend5	4.07	6.31	0.60	2.95E-02	4.81E-01
1600029O15Rik	1.82	3.43	0.90	2.96E-02	4.81E-01
Ston2	0.85	0.48	-0.84	2.98E-02	4.82E-01
Col4a3bp	9.28	12.23	0.37	2.97E-02	4.82E-01
Wiz	2.17	1.60	-0.47	2.98E-02	4.82E-01
Rad23b	35.70	44.25	0.28	2.99E-02	4.82E-01
Tagln	0.43	1.14	1.39	2.97E-02	4.82E-01
Tubb4b	71.29	88.75	0.28	2.97E-02	4.82E-01
Rexo1	8.76	6.14	-0.55	2.97E-02	4.82E-01
Gm9905	0.17	0.63	1.83	2.99E-02	4.82E-01
Gspt2	6.34	8.75	0.43	2.99E-02	4.82E-01
Gm12394	6.96	4.93	-0.54	2.98E-02	4.82E-01
Zfp953	0.44	0.20	-1.14	2.97E-02	4.82E-01
Snhg6	5.13	8.40	0.68	3.00E-02	4.82E-01
Rabgef1	4.66	7.02	0.57	3.00E-02	4.82E-01
Stard4	1.93	2.92	0.57	3.01E-02	4.82E-01
Btbd1	48.29	61.07	0.31	3.02E-02	4.84E-01
Nxt1	6.03	9.29	0.59	3.02E-02	4.84E-01
Gm13303	11.03	7.38	-0.62	3.03E-02	4.84E-01
Galnt9	36.64	27.53	-0.45	3.03E-02	4.84E-01
Cmya5	0.38	0.63	0.68	3.04E-02	4.84E-01
Rpl5	60.24	74.27	0.27	3.04E-02	4.84E-01
Gm5453	65.13	86.09	0.37	3.04E-02	4.85E-01
Efr3a	21.75	26.85	0.27	3.08E-02	4.86E-01
Rnf220	19.74	16.07	-0.33	3.07E-02	4.86E-01
Atp2b2	39.21	33.25	-0.27	3.06E-02	4.86E-01
Nucb1	3.34	2.42	-0.51	3.06E-02	4.86E-01
Sec22a	1.61	2.40	0.54	3.08E-02	4.86E-01
Gns	16.32	13.57	-0.30	3.06E-02	4.86E-01
Parp4	0.33	0.14	-1.30	3.07E-02	4.86E-01
Gpx4	10.73	7.27	-0.60	3.08E-02	4.86E-01
Gm17202	17.15	24.03	0.46	3.06E-02	4.86E-01
Utp14b	1.71	0.95	-0.88	3.08E-02	4.86E-01
Apopt1	4.07	5.80	0.48	3.09E-02	4.86E-01
Mrpl15	6.24	8.07	0.34	3.09E-02	4.87E-01
Adcy9	5.76	4.04	-0.55	3.10E-02	4.87E-01
Atp5mpl	714.10	890.11	0.29	3.13E-02	4.87E-01
Celf3	30.74	22.45	-0.49	3.13E-02	4.87E-01
Sppl3	12.42	10.02	-0.34	3.11E-02	4.87E-01
Arpc1b	2.33	1.60	-0.57	3.11E-02	4.87E-01
Sez6l2	21.66	17.22	-0.37	3.12E-02	4.87E-01
Psenen	51.99	40.29	-0.40	3.15E-02	4.87E-01
Paqr7	4.45	3.12	-0.55	3.13E-02	4.87E-01

Klhl23	8.82	11.43	0.34	3.15E-02	4.87E-01
Tubg2	28.81	22.19	-0.41	3.12E-02	4.87E-01
Rhno1	2.07	3.30	0.64	3.15E-02	4.87E-01
Gm10355	8.00	13.26	0.70	3.14E-02	4.87E-01
Lin52	4.24	6.61	0.61	3.15E-02	4.87E-01
A230065N10Rik	1.86	0.77	-1.31	3.11E-02	4.87E-01
Hist1h2bn	9.80	5.39	-0.90	3.11E-02	4.87E-01
Gm26520	0.67	0.09	-2.86	3.15E-02	4.87E-01
NA	0.45	0.21	-1.14	3.13E-02	4.87E-01
Ranbp1	28.04	35.49	0.31	3.15E-02	4.87E-01
Mapk14	21.58	27.47	0.31	3.15E-02	4.87E-01
S100a6	1.45	0.27	-2.41	3.16E-02	4.87E-01
Pdf	16.05	21.89	0.42	3.16E-02	4.87E-01
Peg10	5.72	4.29	-0.44	3.18E-02	4.90E-01
Eif3h	41.09	53.34	0.35	3.19E-02	4.90E-01
Zhx3	9.10	7.48	-0.32	3.19E-02	4.90E-01
Acat3	35.71	44.74	0.29	3.20E-02	4.90E-01
Ttc9b	29.34	21.75	-0.47	3.23E-02	4.93E-01
Vps52	7.74	5.76	-0.46	3.23E-02	4.93E-01
Glipr1	7.79	11.84	0.57	3.22E-02	4.93E-01
Mipep	9.83	13.39	0.41	3.24E-02	4.95E-01
Gm10069	1.41	2.26	0.65	3.24E-02	4.95E-01
Shisal1	11.27	9.34	-0.30	3.24E-02	4.95E-01
Efna2	1.24	0.48	-1.39	3.25E-02	4.95E-01
Slc25a4	335.81	415.04	0.28	3.25E-02	4.95E-01
Trim46	9.32	7.38	-0.37	3.25E-02	4.95E-01
Rps13-ps5	69.74	90.11	0.34	3.26E-02	4.95E-01
Lrrfip2	7.55	10.19	0.40	3.27E-02	4.96E-01
Tuft1	0.20	0.87	2.11	3.28E-02	4.97E-01
Abhd5	4.62	2.77	-0.77	3.28E-02	4.97E-01
Tnr	8.65	6.74	-0.40	3.30E-02	4.99E-01
Mmp24	8.92	7.06	-0.37	3.30E-02	4.99E-01
Zfp628	2.17	1.39	-0.68	3.31E-02	5.00E-01
Ell3	2.43	5.03	1.03	3.32E-02	5.00E-01
Esyt3	0.24	0.07	-1.77	3.32E-02	5.00E-01
Plec	0.51	0.34	-0.61	3.33E-02	5.01E-01
Kctd17	3.87	2.42	-0.72	3.33E-02	5.01E-01
Zfp423	4.90	3.50	-0.52	3.34E-02	5.01E-01
2510002D24Rik	3.21	5.27	0.68	3.34E-02	5.02E-01
Ppp1r16b	8.62	7.05	-0.32	3.35E-02	5.02E-01
Dnmt3a	1.64	1.26	-0.42	3.36E-02	5.03E-01
Hist1h2bc	31.19	46.08	0.54	3.38E-02	5.03E-01
Orc2	7.84	10.73	0.43	3.37E-02	5.03E-01
Mrps33	353.92	435.85	0.27	3.39E-02	5.03E-01
Me1	17.78	23.68	0.39	3.37E-02	5.03E-01
Shc4	2.96	2.08	-0.54	3.37E-02	5.03E-01
Camsap3	5.09	3.69	-0.50	3.39E-02	5.03E-01
Polr2k	46.48	61.60	0.38	3.39E-02	5.03E-01
Tank	3.70	4.93	0.39	3.38E-02	5.03E-01
Fndc10	15.57	12.12	-0.40	3.38E-02	5.03E-01
NA	15.59	12.13	-0.40	3.38E-02	5.03E-01
Gm6061	1294.28	1562.57	0.24	3.39E-02	5.03E-01

Zfp84	5.43	3.97	-0.49	3.40E-02	5.03E-01
Pop5	31.41	39.68	0.31	3.41E-02	5.03E-01
Gm9396	25.46	35.34	0.44	3.40E-02	5.03E-01
Spata7	3.87	5.19	0.39	3.42E-02	5.05E-01
Dtnbp1	58.27	73.72	0.31	3.42E-02	5.05E-01
Nipal3	3.64	2.83	-0.40	3.43E-02	5.05E-01
Mpc1	69.89	92.79	0.38	3.44E-02	5.05E-01
Camkk2	15.76	19.49	0.27	3.44E-02	5.05E-01
Cluh	5.49	4.15	-0.44	3.45E-02	5.06E-01
Fahd1	19.51	27.87	0.49	3.45E-02	5.06E-01
Morn4	53.32	40.29	-0.44	3.45E-02	5.06E-01
Rps15a-ps7	97.18	123.93	0.32	3.45E-02	5.06E-01
Gm2962	23.04	34.04	0.53	3.46E-02	5.06E-01
Lsm12	39.56	31.59	-0.36	3.47E-02	5.06E-01
Psmd14	25.25	33.22	0.36	3.47E-02	5.06E-01
Sh3bgrl3	328.71	267.36	-0.33	3.47E-02	5.06E-01
Rwdd1	55.44	69.87	0.30	3.48E-02	5.07E-01
Rpl22l1	141.64	188.36	0.38	3.48E-02	5.07E-01
Rars	7.86	10.76	0.42	3.49E-02	5.07E-01
Lrp8	4.59	3.69	-0.35	3.50E-02	5.08E-01
Fam222b	33.00	27.15	-0.32	3.51E-02	5.08E-01
Ctnna1	5.40	7.36	0.41	3.50E-02	5.08E-01
Zfp14	3.85	2.22	-0.83	3.50E-02	5.08E-01
Zfp992	1.73	1.04	-0.78	3.51E-02	5.08E-01
Gm15879	2.02	0.87	-1.24	3.50E-02	5.08E-01
Manbal	17.28	12.59	-0.49	3.52E-02	5.08E-01
Ctsl	13.55	18.93	0.45	3.54E-02	5.09E-01
Prelid1	71.26	58.41	-0.32	3.53E-02	5.09E-01
Mgat5	4.88	3.59	-0.48	3.53E-02	5.09E-01
Zcchc2	8.40	10.78	0.33	3.54E-02	5.09E-01
Serpina3e-ps	6.68	3.15	-1.11	3.54E-02	5.09E-01
Rangap1	20.50	15.99	-0.39	3.55E-02	5.10E-01
Thoc1	8.81	11.75	0.39	3.55E-02	5.10E-01
Syvn1	10.56	8.28	-0.38	3.55E-02	5.10E-01
Pdpr	2.59	1.76	-0.59	3.55E-02	5.10E-01
Hrnr	0.24	0.02	-3.96	3.56E-02	5.10E-01
Lancl3	1.18	0.64	-0.91	3.57E-02	5.11E-01
Mpc2	49.57	61.60	0.28	3.58E-02	5.11E-01
Stpg1	3.15	1.92	-0.75	3.57E-02	5.11E-01
Rps15a-ps6	115.76	146.69	0.31	3.58E-02	5.11E-01
Snrpd2	77.94	59.09	-0.44	3.59E-02	5.12E-01
Slc29a3	4.43	2.95	-0.62	3.61E-02	5.13E-01
Foxp4	4.57	3.02	-0.64	3.61E-02	5.13E-01
Ugt8a	0.61	0.26	-1.27	3.61E-02	5.13E-01
Zdhhc14	13.63	10.76	-0.38	3.61E-02	5.13E-01
Gm16740	172.58	213.76	0.28	3.60E-02	5.13E-01
Rasgrp4	1.13	0.66	-0.82	3.62E-02	5.13E-01
NA	607.86	749.13	0.27	3.62E-02	5.13E-01
Tmem201	2.27	1.61	-0.53	3.63E-02	5.13E-01
Ganc	0.83	0.52	-0.71	3.63E-02	5.13E-01
Dek	24.30	30.58	0.30	3.63E-02	5.14E-01
Trappc6b	142.92	179.35	0.30	3.64E-02	5.14E-01

Gm21985	7.82	6.27	-0.36	3.64E-02	5.14E-01
Eif1b	109.16	136.25	0.29	3.67E-02	5.14E-01
Cnot8	11.04	14.42	0.35	3.67E-02	5.14E-01
Mink1	18.33	14.53	-0.37	3.66E-02	5.14E-01
Zc3h7b	5.64	3.99	-0.54	3.66E-02	5.14E-01
Tmem231	2.92	1.73	-0.79	3.66E-02	5.14E-01
Inpp5j	4.14	3.12	-0.44	3.67E-02	5.14E-01
Usp27x	7.86	10.48	0.39	3.68E-02	5.14E-01
Chst8	3.58	2.48	-0.57	3.68E-02	5.14E-01
B2m	104.14	154.70	0.54	3.68E-02	5.14E-01
Kiz	5.79	8.22	0.47	3.68E-02	5.14E-01
Ttbk1	3.37	2.44	-0.50	3.69E-02	5.14E-01
Gstcd	0.23	0.53	1.19	3.68E-02	5.14E-01
Fscn1	14.21	10.61	-0.46	3.69E-02	5.15E-01
Gm15920	176.27	219.73	0.29	3.70E-02	5.15E-01
Emd	51.23	63.44	0.27	3.71E-02	5.16E-01
Sec11c	12.16	16.09	0.37	3.72E-02	5.16E-01
Magee2	16.96	12.57	-0.46	3.72E-02	5.16E-01
Lrrc20	14.38	11.19	-0.40	3.71E-02	5.16E-01
Trerf1	12.12	9.26	-0.43	3.72E-02	5.16E-01
Hdac9	19.57	25.24	0.34	3.73E-02	5.16E-01
Hcn3	0.88	0.50	-0.83	3.73E-02	5.16E-01
Rela	1.62	0.94	-0.81	3.73E-02	5.16E-01
Stard3	2.20	0.95	-1.27	3.75E-02	5.16E-01
Dhx33	6.45	8.27	0.33	3.74E-02	5.16E-01
Lypd6	0.90	0.37	-1.31	3.74E-02	5.16E-01
Nrep	38.85	51.14	0.36	3.75E-02	5.17E-01
Chrna7	6.99	5.04	-0.50	3.76E-02	5.17E-01
Slc16a2	11.01	8.88	-0.34	3.76E-02	5.17E-01
Ncam1	49.56	42.09	-0.27	3.77E-02	5.17E-01
Aplp1	466.38	403.21	-0.24	3.78E-02	5.17E-01
B3galnt2	2.93	4.25	0.51	3.78E-02	5.17E-01
Teddm2	1.38	0.58	-1.29	3.77E-02	5.17E-01
9-Sep	5.48	4.15	-0.44	3.78E-02	5.17E-01
Timp3	0.68	0.34	-1.04	3.79E-02	5.18E-01
Wdtc1	8.57	6.27	-0.49	3.79E-02	5.18E-01
Mrps23	14.44	19.23	0.38	3.81E-02	5.19E-01
Ganab	7.93	6.20	-0.39	3.81E-02	5.19E-01
Coq6	0.72	1.43	0.96	3.82E-02	5.20E-01
Gsk3a	209.80	172.83	-0.31	3.82E-02	5.20E-01
4930419G24Rik	10.08	6.25	-0.73	3.82E-02	5.20E-01
Rgs11	0.37	0.10	-1.91	3.85E-02	5.21E-01
Rftn2	0.11	0.32	1.52	3.84E-02	5.21E-01
Gm6498	0.92	0.39	-1.25	3.84E-02	5.21E-01
Ubr4	3.87	3.03	-0.39	3.84E-02	5.21E-01
Gm13750	29.57	41.83	0.47	3.86E-02	5.23E-01
Timm9	36.78	47.56	0.34	3.87E-02	5.23E-01
Fyco1	2.92	2.07	-0.53	3.87E-02	5.23E-01
Rps15a-ps5	47.96	63.25	0.37	3.87E-02	5.23E-01
Psma2	159.64	195.72	0.26	3.89E-02	5.25E-01
Nab2	9.42	6.33	-0.62	3.89E-02	5.25E-01
Pagr1b	10.09	6.21	-0.74	3.89E-02	5.25E-01

Crtc1	16.61	13.83	-0.30	3.93E-02	5.25E-01
Rab5a	17.13	22.16	0.34	3.94E-02	5.25E-01
Dcbld1	0.61	1.13	0.86	3.93E-02	5.25E-01
Rdh14	31.57	40.16	0.32	3.95E-02	5.25E-01
Serpina3n	54.58	43.55	-0.36	3.93E-02	5.25E-01
Bmpr1a	8.78	11.36	0.34	3.92E-02	5.25E-01
Pi4k2b	0.25	0.07	-1.86	3.93E-02	5.25E-01
Proser3	0.90	0.49	-0.90	3.92E-02	5.25E-01
Man2a2	26.45	22.52	-0.27	3.91E-02	5.25E-01
Daam2	0.33	0.11	-1.60	3.92E-02	5.25E-01
Prrt3	2.52	1.23	-1.07	3.94E-02	5.25E-01
Sgms2	0.26	0.07	-1.89	3.90E-02	5.25E-01
Mrfap1	520.90	647.01	0.28	3.95E-02	5.25E-01
Slit3	12.84	10.46	-0.33	3.91E-02	5.25E-01
Atp5l-ps1	512.91	639.54	0.29	3.95E-02	5.25E-01
Gm12841	3.30	1.14	-1.56	3.95E-02	5.25E-01
Ung	0.57	1.19	1.03	3.96E-02	5.25E-01
Egln2	5.31	3.49	-0.64	3.96E-02	5.25E-01
Gm13767	23.99	18.12	-0.44	3.96E-02	5.25E-01
Mocs2	13.56	16.60	0.26	3.97E-02	5.25E-01
Slc7a4	4.06	2.89	-0.52	3.97E-02	5.25E-01
C030047K22Rik	1.56	0.41	-1.97	3.97E-02	5.25E-01
Tom1l2	21.58	18.40	-0.26	4.13E-02	5.26E-01
Nfix	49.26	40.32	-0.33	4.03E-02	5.26E-01
Car11	62.34	43.77	-0.55	4.00E-02	5.26E-01
Efnb3	18.24	14.97	-0.32	4.02E-02	5.26E-01
Cluap1	7.78	11.42	0.53	4.03E-02	5.26E-01
Adss	30.42	37.39	0.26	4.14E-02	5.26E-01
Dpep1	0.73	0.12	-2.69	4.00E-02	5.26E-01
Katna1	1.42	2.60	0.84	4.11E-02	5.26E-01
Timm13	16.49	11.82	-0.52	4.02E-02	5.26E-01
Cacna1g	1.52	1.06	-0.56	4.07E-02	5.26E-01
Ylpm1	18.98	15.84	-0.30	4.08E-02	5.26E-01
Htr1a	12.24	9.20	-0.45	4.05E-02	5.26E-01
Celsr3	0.86	0.60	-0.54	4.05E-02	5.26E-01
Paqr4	12.10	8.92	-0.48	4.12E-02	5.26E-01
Cnpy3	7.09	5.20	-0.48	4.07E-02	5.26E-01
Fth1	779.52	959.05	0.27	4.15E-02	5.26E-01
Dpf2	6.54	5.19	-0.37	4.02E-02	5.26E-01
Ric8a	7.62	9.80	0.33	4.10E-02	5.26E-01
Rrbp1	0.54	0.30	-0.91	4.09E-02	5.26E-01
Ndufb5	94.88	117.29	0.28	4.15E-02	5.26E-01
Bche	0.91	0.43	-1.13	4.05E-02	5.26E-01
Cmpk1	45.38	56.95	0.30	4.05E-02	5.26E-01
Sfswap	5.93	4.42	-0.45	4.06E-02	5.26E-01
Fam189a1	44.25	35.76	-0.34	4.08E-02	5.26E-01
Adgra2	0.30	0.59	0.92	4.11E-02	5.26E-01
Ist1	31.35	39.89	0.32	4.13E-02	5.26E-01
Psma4	31.21	39.39	0.30	4.11E-02	5.26E-01
Hmg20a	20.96	17.04	-0.33	4.02E-02	5.26E-01
Farp2	3.95	5.28	0.39	4.14E-02	5.26E-01
Fam69b	6.78	4.99	-0.47	4.10E-02	5.26E-01

Igfbp7	2.54	4.57	0.82	4.06E-02	5.26E-01
Stk35	2.94	2.11	-0.51	4.07E-02	5.26E-01
Cntln	2.59	3.55	0.42	4.13E-02	5.26E-01
Bend3	1.64	1.01	-0.73	4.07E-02	5.26E-01
Vars2	0.83	0.49	-0.79	4.15E-02	5.26E-01
Stub1	22.70	18.04	-0.37	4.09E-02	5.26E-01
Lrp1	3.07	2.33	-0.44	4.04E-02	5.26E-01
Etfrf1	13.73	17.59	0.33	4.04E-02	5.26E-01
Tox3	7.73	5.78	-0.45	4.15E-02	5.26E-01
Mageh1	9.69	14.11	0.52	4.15E-02	5.26E-01
Lurap1l	16.08	21.25	0.37	4.14E-02	5.26E-01
Ankrd34a	94.76	73.51	-0.41	4.13E-02	5.26E-01
Tmem248	12.00	9.75	-0.33	4.08E-02	5.26E-01
Serpinb6a	2.52	3.59	0.48	4.13E-02	5.26E-01
mt-Atp6	76.65	51.63	-0.59	4.03E-02	5.26E-01
Wdr35	1.94	2.86	0.53	4.15E-02	5.26E-01
Ptprz1	2.77	1.99	-0.51	4.09E-02	5.26E-01
Eloc	109.56	138.60	0.31	4.10E-02	5.26E-01
H3f3c	133.42	110.60	-0.30	4.11E-02	5.26E-01
Gm26596	2.90	1.88	-0.67	4.01E-02	5.26E-01
Arg2	9.90	14.24	0.50	4.16E-02	5.27E-01
Tesk1	8.99	7.06	-0.38	4.17E-02	5.27E-01
Med17	2.42	3.74	0.60	4.19E-02	5.29E-01
NA	25.52	15.50	-0.76	4.19E-02	5.29E-01
Amigo1	11.02	8.73	-0.37	4.18E-02	5.29E-01
Oprm1	0.21	0.50	1.21	4.32E-02	5.29E-01
Nacc1	34.77	27.81	-0.36	4.32E-02	5.29E-01
Slc4a3	1.51	1.09	-0.50	4.34E-02	5.29E-01
Phax	19.15	24.18	0.30	4.32E-02	5.29E-01
Tenm1	5.72	4.79	-0.29	4.21E-02	5.29E-01
Gid4	6.10	4.83	-0.37	4.31E-02	5.29E-01
Rack1	10.43	13.29	0.32	4.20E-02	5.29E-01
Mettl3	2.52	3.87	0.59	4.31E-02	5.29E-01
Ndufa5	118.19	151.28	0.32	4.29E-02	5.29E-01
Ndufv2	66.57	82.46	0.28	4.26E-02	5.29E-01
Setbp1	9.39	7.82	-0.30	4.25E-02	5.29E-01
Nabp2	40.22	33.15	-0.31	4.29E-02	5.29E-01
Nvl	3.70	5.15	0.44	4.30E-02	5.29E-01
F5	0.44	0.13	-1.83	4.27E-02	5.29E-01
Suv39h2	3.90	2.67	-0.58	4.31E-02	5.29E-01
Gss	0.97	0.60	-0.73	4.26E-02	5.29E-01
Dmac1	48.20	61.46	0.32	4.27E-02	5.29E-01
Nsun7	0.78	1.42	0.83	4.20E-02	5.29E-01
Coro1a	7.60	5.76	-0.44	4.30E-02	5.29E-01
Abcg4	5.77	4.27	-0.47	4.33E-02	5.29E-01
Scaf11	7.34	9.19	0.30	4.31E-02	5.29E-01
Scara3	0.46	0.95	1.02	4.27E-02	5.29E-01
G6pc3	8.70	6.32	-0.50	4.32E-02	5.29E-01
Mospd3	23.75	17.11	-0.51	4.26E-02	5.29E-01
Tbcel	4.46	3.35	-0.45	4.21E-02	5.29E-01
Scarb1	1.12	0.68	-0.76	4.23E-02	5.29E-01
Tmem268	1.20	2.15	0.80	4.25E-02	5.29E-01

Ppp1r15b	3.29	4.48	0.41	4.21E-02	5.29E-01
Lrrc75a	1.55	2.93	0.89	4.32E-02	5.29E-01
Sox1ot	1.36	0.80	-0.81	4.29E-02	5.29E-01
Magi3	7.26	9.54	0.37	4.33E-02	5.29E-01
Rps6-ps2	23.53	31.83	0.41	4.22E-02	5.29E-01
Zfp462	13.84	10.54	-0.44	4.26E-02	5.29E-01
Zfp873	2.98	4.80	0.66	4.25E-02	5.29E-01
Grm4	1.96	1.32	-0.60	4.23E-02	5.29E-01
Hnrnpr	10.27	12.83	0.29	4.23E-02	5.29E-01
Gpr88	0.68	1.29	0.88	4.30E-02	5.29E-01
Fat3	9.61	7.65	-0.37	4.25E-02	5.29E-01
Txnrd2	1.69	0.95	-0.87	4.24E-02	5.29E-01
Gm26384	318.30	417.72	0.36	4.23E-02	5.29E-01
Bambi-ps1	1.41	0.38	-1.93	4.32E-02	5.29E-01
Gm14329	3.47	1.56	-1.19	4.34E-02	5.29E-01
Gm6395	123.55	157.19	0.32	4.30E-02	5.29E-01
Syt6	2.02	3.21	0.64	4.36E-02	5.31E-01
Pcmt1	41.21	50.36	0.26	4.37E-02	5.32E-01
Ptp4a3	1.68	1.10	-0.65	4.37E-02	5.32E-01
Snrnp48	3.46	5.53	0.65	4.39E-02	5.32E-01
Cog8	8.73	11.43	0.36	4.39E-02	5.32E-01
Gm4924	0.38	0.09	-2.09	4.40E-02	5.32E-01
Gm14278	53.84	38.73	-0.50	4.39E-02	5.32E-01
Gm17036	32.06	46.89	0.52	4.39E-02	5.32E-01
Ing2	6.99	9.50	0.41	4.40E-02	5.33E-01
Srsf1	10.01	12.40	0.28	4.41E-02	5.33E-01
Crocc	0.30	0.15	-1.02	4.41E-02	5.33E-01
Rpl36a	48.93	60.52	0.27	4.41E-02	5.33E-01
Gm15832	12.26	17.17	0.46	4.41E-02	5.33E-01
Gm20671	9.38	6.59	-0.55	4.42E-02	5.33E-01
Ncan	65.33	56.73	-0.24	4.46E-02	5.35E-01
Sod2	171.38	141.89	-0.31	4.45E-02	5.35E-01
Acap3	9.05	7.21	-0.36	4.45E-02	5.35E-01
Gemin6	3.65	6.22	0.74	4.45E-02	5.35E-01
NA	22.53	15.77	-0.55	4.46E-02	5.35E-01
Gm15793	12.53	18.84	0.56	4.46E-02	5.35E-01
Wdr6	12.54	15.94	0.32	4.47E-02	5.36E-01
Tnk2	7.68	5.97	-0.40	4.51E-02	5.37E-01
Incenp	0.80	1.44	0.82	4.49E-02	5.37E-01
Dnajb6	48.54	61.23	0.31	4.50E-02	5.37E-01
Sigmar1	3.14	2.18	-0.56	4.50E-02	5.37E-01
Tmc7	1.47	0.91	-0.74	4.51E-02	5.37E-01
Zfp691	1.86	0.96	-1.00	4.51E-02	5.37E-01
Zscan29	0.89	1.39	0.61	4.50E-02	5.37E-01
Grwd1	1.71	2.90	0.73	4.50E-02	5.37E-01
Gm6472	142.09	173.18	0.25	4.49E-02	5.37E-01
Ido1	0.62	0.07	-3.20	4.52E-02	5.38E-01
Ptpn21	1.17	0.58	-1.05	4.52E-02	5.38E-01
Arhgap39	13.71	11.33	-0.31	4.54E-02	5.38E-01
Klhl35	0.49	0.16	-1.67	4.54E-02	5.38E-01
Sez6l	24.02	19.71	-0.32	4.54E-02	5.38E-01
Sco1	1.73	1.17	-0.60	4.54E-02	5.38E-01

Rac3	0.73	0.24	-1.64	4.55E-02	5.38E-01
Clcn6	10.35	8.64	-0.29	4.55E-02	5.39E-01
Gm21981	29.24	23.44	-0.35	4.56E-02	5.39E-01
Polr2a	10.82	8.75	-0.34	4.57E-02	5.40E-01
Tmem38b	2.06	3.01	0.51	4.58E-02	5.40E-01
Rspo1	1.22	0.33	-1.94	4.58E-02	5.40E-01
Fam183b	13.18	20.05	0.58	4.57E-02	5.40E-01
Camta2	150.63	123.85	-0.32	4.58E-02	5.40E-01
Micall1	1.98	1.44	-0.49	4.60E-02	5.41E-01
Gm11605	24.79	17.95	-0.50	4.60E-02	5.41E-01
Gm11425	22.74	31.79	0.45	4.60E-02	5.41E-01
Fut9	5.25	6.69	0.32	4.61E-02	5.42E-01
Ppih	7.76	10.91	0.47	4.62E-02	5.43E-01
Mlh3	7.94	5.86	-0.47	4.64E-02	5.43E-01
Rpl30	63.48	77.10	0.25	4.64E-02	5.43E-01
Tspyl3	10.50	8.09	-0.41	4.64E-02	5.43E-01
Ift43	4.10	6.78	0.69	4.65E-02	5.43E-01
Gm14138	26.16	35.57	0.42	4.65E-02	5.43E-01
Cacnb3	52.86	44.01	-0.30	4.67E-02	5.46E-01
Fbxw5	21.55	17.39	-0.35	4.68E-02	5.46E-01
Cbl	12.89	10.77	-0.29	4.68E-02	5.46E-01
Lamtor3	30.16	38.19	0.31	4.68E-02	5.46E-01
Myo19	2.83	3.96	0.46	4.70E-02	5.46E-01
Ccndbp1	31.21	37.89	0.25	4.72E-02	5.46E-01
Rps6kb2	1.45	0.82	-0.86	4.70E-02	5.46E-01
Sc5d	9.50	7.20	-0.44	4.73E-02	5.46E-01
Fam96a	15.46	21.26	0.43	4.70E-02	5.46E-01
Rbbp8	1.45	2.55	0.79	4.72E-02	5.46E-01
Spidr	1.30	0.72	-0.90	4.72E-02	5.46E-01
Nat8l	15.98	12.64	-0.38	4.71E-02	5.46E-01
Mphosph8	12.88	17.31	0.40	4.69E-02	5.46E-01
Ccl21b	7.90	4.07	-1.00	4.72E-02	5.46E-01
A330074K22Rik	0.44	0.13	-1.74	4.72E-02	5.46E-01
Zic5	0.33	0.08	-2.08	4.74E-02	5.47E-01
Mark4	10.85	7.87	-0.50	4.75E-02	5.48E-01
Arhgap35	71.80	61.86	-0.25	4.76E-02	5.49E-01
Gk	1.07	1.67	0.62	4.77E-02	5.49E-01
Rnf34	26.97	22.00	-0.33	4.77E-02	5.50E-01
Gm5510	2.22	0.81	-1.48	4.78E-02	5.51E-01
Insm1	2.46	3.94	0.65	4.79E-02	5.51E-01
Lrrc10b	12.04	8.40	-0.56	4.79E-02	5.51E-01
Mir9-3hg	2.41	3.73	0.60	4.80E-02	5.51E-01
Gria1	83.03	69.45	-0.30	4.81E-02	5.51E-01
5730522E02Rik	1.04	1.69	0.67	4.81E-02	5.51E-01
Alg1	4.05	2.86	-0.53	4.82E-02	5.52E-01
Cacna1h	3.16	2.54	-0.35	4.85E-02	5.53E-01
Ppp1r11	32.48	26.82	-0.31	4.84E-02	5.53E-01
Dsc3	0.33	0.65	0.93	4.85E-02	5.53E-01
Gm11966	299.19	395.89	0.38	4.85E-02	5.53E-01
Gm12416	5.09	3.27	-0.67	4.85E-02	5.53E-01
Rpl6l	130.57	161.12	0.27	4.85E-02	5.53E-01
Ppig	15.25	19.77	0.34	4.86E-02	5.54E-01

Pianp	59.28	48.05	-0.34	4.87E-02	5.55E-01
Prlr	0.71	0.49	-0.56	4.88E-02	5.55E-01
Rprd2	16.30	13.81	-0.27	4.88E-02	5.55E-01
Rad54l2	3.43	2.67	-0.40	4.89E-02	5.55E-01
Fam98c	2.72	1.71	-0.71	4.89E-02	5.56E-01
Bbs4	13.80	18.13	0.36	4.91E-02	5.57E-01
Tbkbp1	2.21	1.43	-0.66	4.91E-02	5.57E-01
Lrp3	5.99	4.54	-0.44	4.92E-02	5.57E-01
Vcam1	0.93	0.12	-3.02	4.94E-02	5.57E-01
Cap1	43.03	36.76	-0.26	4.93E-02	5.57E-01
Ndufb11	111.48	136.15	0.26	4.93E-02	5.57E-01
Resf1	5.20	4.10	-0.38	4.92E-02	5.57E-01
Tomm6	70.83	57.04	-0.34	4.94E-02	5.57E-01
Cep126	10.45	13.20	0.30	4.92E-02	5.57E-01
Mir3082	61.54	38.11	-0.72	4.94E-02	5.57E-01
Erbin	1.42	0.89	-0.70	4.95E-02	5.57E-01
Cst3	83.16	69.57	-0.29	4.95E-02	5.57E-01
Ddx55	2.81	3.91	0.45	4.95E-02	5.57E-01
Srsf5	36.46	31.14	-0.26	4.99E-02	5.58E-01
Noct	7.23	8.89	0.27	4.98E-02	5.58E-01
Zfp106	44.08	38.28	-0.24	4.97E-02	5.58E-01
Epb41	0.38	0.64	0.72	4.99E-02	5.58E-01
BC017158	1.32	0.85	-0.67	4.97E-02	5.58E-01
Kcnh3	4.19	2.95	-0.55	4.99E-02	5.58E-01
Spen	4.39	3.39	-0.41	4.97E-02	5.58E-01
Yipf6	14.59	12.14	-0.30	4.99E-02	5.58E-01
Ndfip2	6.29	7.88	0.29	4.98E-02	5.58E-01
Gm10250	66.02	83.26	0.30	4.99E-02	5.58E-01
Tipin	5.55	8.59	0.60	5.00E-02	5.58E-01
