# Supplementary material for: Identification of Neurensin-2 as a novel modulator of emotional behavior
Source: Mol Psychiatry. 2021 Mar 19;26(7):2872–85. doi: 10.1038/s41380-021-01058-5 (PMC8505262; doi:10.1038/s41380-021-01058-5)
Supplement: Supplementary file 4 — Table S2 [file 41380_2021_1058_MOESM4_ESM.pdf]

Table S2: Genes that are changed in CCK-cKO and enriched in GAD2 cells

changed+ enriched genes	854	Psma4
		Ppia
		Phax
		Spock2
		Rpl23a-ps3
		Psmg2
		Arhgap35
		Nrep
		Samd10
		Ccdc106
		Nos1
		Sod2
		Dusp26
		Ly6e
		Ccdc71
		Wls
		Fahd1
		Ap1s1
		Ptprs
		Tubb4b
		Lta4h
		Tk2
		Brms1l
		Ppp2ca
		Nts
		Tecr
		Scarb1
		Mrps33
		Elavl3
		AI593442
		Alg1
		Noct
		Lrrc20
		Rps6-ps2
		Chchd2
		Slc24a3
		Pld3
		Prkn
		Dhcr7
		Spata7
		Usp27x
		Eloc
		Alg8
		Vps39

Rpl6l
Dyrk1b
Mpc1
Anks3
Proser3
Mbd1
Atp6v0e2
Smarcd3
Cog8
Rps27a
Lamtor3
Man1c1
Rangap1
Pcna
Prn
Rnf144b
Pdha1
Aktip
Nat6
Tomm7
Dele1
Lrrc4b
Arl8a
Ensa
Caly
Prpf39
Rwdd1
Rexo1
Aplp1
Nt5dc2
Pgbd5
Tlcd1
Naif1
Lrrc75a
Mdh1
Smim26
Cox6c
Bend3
Ap2a1
Tmem59l
Fam183b
Cmpk1
Foxp4
Rnf34
Disp2
Eif1b
Mef2c

Nrsn2
Tnr
Thoc1
Tmem254b
Tiam2
Chchd2-ps
Zic5
Shisal1
Clip2
Cd59a
Lancl3
Copb2
Plxnb2
Zfp438
Polr2a
Psma2
Tesk1
Cox6c2
Ggct
Pop5
Hdac9
Atp6v0a1
Ulk1
Eif4e2
Nkiras1
Col4a2
Atxn10
Katna1
Car11
Celf5
Por
Grwd1
Rbbp8
Apc2
Syvn1
Igsf8
Rabgef1
Nabp2
Utp14b
Kin
Hyou1
Timp3
Lsm2
Shc4
Slc15a3
Sh3bgrl3
Celsr2

Stard3
Rtraf
Tmem214
Mir686
Bambi-ps1
Ctnnbl1
H3f3a-ps2
Rdh14
Tyw5
Jmjd8
Brdt
Cpt1c
Btbd1
Ift43
Fam219aos
Vti1b
Tada3
Rpl36a
Cox17
Erg28
Aip
Paqr7
Fkbp3
Krcc1
Bend5
Prpf8
Creld1
Cbarp
Atn1
Gcn1l1
Ppih
Farp2
Acat3
Mrpl15
Eef1b2
Mon1a
Sema6a
Ppp1r15b
Lin52
Rpl27a
Acsl1
Agap2
Setd1a
Sec11c
Ttbk1
Ccna2
Hnrnpul1

Ly6h
Rps24
Cntnap1
AF529169
Ccdc159
Hnrnpr
E030024N20Rik
Srsf1
Sap30
H3f3c
Ndufv2
Tmem251
Ndufaf5
Slc25a22
Dnajb6
Adgrl1
Shld1
Hist1h2bl
Camsap3
Prelid1
Tox3
Tmem63c
Dpf2
Tipin
Morn4
Bcor
Arpc1a
Rps6
Nova1
Hist1h2bc
Rps13-ps1
Plcxd2
Gal3st3
Dlgap3
Rpl9-ps7
Arpc5l
Chd3
Atp5l-ps1
Zmiz2
Pdf
Clcn7
Emd
Man2b1
Zfp839
Rpl30
Fchsd1
Rps6kb2

Mark2
Rangrf
Porcn
Efnb3
Trappc6b
Ap4s1
Cacnb3
Rela
Lpcat4
Dcun1d5
B2m
Nrxn2
Prrc2b
Pp2d1
Pianp
Actg1
Ptp4a3
Rhno1
Spin2c
Gna11
Ndufb11
Pfdn4
Zcrb1
Mageh1
Mpc1-ps
Hcn3
Rps15a-ps7
Rassf3
Prrt3
Mpped2
Cbx3
Ndufa2
Naca
Tob1
G6pc3
Prr5
Ajm1
Zic4
Id2
Rai1
Ubc
Tubg2
Rsrc1
Micu3
Wdr83os
Ap5z1
Palm

Reep2
Ankrd52
Arhgap23
Rars
Cbln4
Mrps23
Ndufa6
Kcnb1
Adgrb1
Swsap1
Man2a2
Shank3
Mphosph8
Ccndbp1
Gapdh
Rps13-ps2
Tiparp
Atp2b2
Shisa7
Atxn7l2
Fjx1
Rack1
Iglon5
Lrrtm1
Ighm
Elfn2
Sdf2l1
Wiz
Cnnm1
Mipep
Dctn1
Grik5
Cbx3-ps7
Zbed5
Vps25
Rps27l
Eif3f
Ifngr2
Rps13
Camkk2
Bcl9l
Slc8a3
Mgat5
Tsc22d3
Vsnl1
BC017158
Dcaf15

Zfp710
Oprm1
Cbx6
Tppp3
Mlec
Zfp462
Mrpl18
Rpl23a
Hist1h2bn
Ist1
Coro1a
Tomm6os
Caskin1
Gpr108
Mrps12
C2cd2l
Rpp38
Rnf220
Micall1
Pin4
Rchy1
Neurl1a
Ncdn
Psmb1
Pom121
Slc35a4
Ezh1
Sox11
Vars2
Rasgrp4
Coprs
Mark4
Suv39h2
Prrt1
Adam15
Pabpc1l2b-ps
Tpd52l1
Ndufa4
Cacna1a
Rbfox2
Scrt2
Zfp574
Ctsl
Hdac5
Selenow
Yeats2
Ndufb5

Basp1
Dad1
Amigo1
Lypd6
Necab1
Smim27
Hmgn1
Jag2
Polr1a
Kif1a
Nifk
Atp5f1
Egln2
Tusc1
Higd1a
Seh1l
Chtf8
Atpaf1
Hsd3b7
Abcg4
Atf4
Ric8a
Rnasek
Sec22a
Rps13-ps4
Pcdhb12
Tmem268
Syngap1
Ndst1
Bbs4
Mir9-3hg
Ell3
Rps6-ps4
Usf1
Myo19
Adcy5
Fam98c
Scap
Sc5d
Ufc1
Zfp14
Pde4a
Ccdc97
Shank1
Sh2b1
Mak16
Trim65

Pex6
Zc3h3
Galnt16
Tank
Pabpc1l2a-ps
Scrg1
Ddx55
Orc2
Rps15a-ps6
Ranbp1
Naga
Ptms
Dolpp1
Rpl12
Fam166a
Cetn4
Tuba1c
Scaf11
Frg2f1
Cyb5r3
Blvrb
Alg11
Tmem38b
Grm4
Tom1l2
Lgi3
Etfa
Cend1
Pnma3
Tomm6
Rac3
Mpp2
Peg10
Pnkd
Cep126
Eif3h
Tmem254a
Zfp277
Aif1
Syt9
Bex4
Ldb3
Cops4
Uqcrb
Rps6-ps3
Tedc2
Nab2

Serpinb6a
Scaf1
Coq6
Mrps27
Tpt1-ps3
Ttc9b
Msrb2
Tspyl3
Tnk2
Eid2
Mkks
Grin1
Gpx4
Rptor
Timm9
Katnb1
Hmgxb3
Eif6
NA
Dcp1b
Nfs1
D630044L22Rik
Sf3b4
Tbkbp1
Pcnx2
Sall2
Rpl9
Apopt1
Adh5
Tubb4a
Cluh
S100a6
Rps20
Rusc2
Gspt2
Tmem63b
Syt3
Slc35e2
Mrfap1
Bbip1
Pcgf5
Celsr3
Stk32c
Fam216a
Cln3
Tmem255a
Rpa3

Arhgdia
Slc6a11
Toe1
Afap1
Bud31
Gabra3
Grina
Magee2
Hmg20a
Tmem231
Cox16
Kiz
Gtpbp2
Slc29a3
Dmac1
Creb3l1
Atp5g1
Dck
Map10
Oaz1-ps
Fam204a
Mcrip1
Ndfip2
Timm13
Atp1a3
Mettl3
Chkb
Nxt1
Commd7
Kdelr2
Rapgef1
Gnl1
Bcr
Tab1
Rprd2
Ppa1
Chchd7
Uqcrfs1
Fndc10
Gabarapl2
Atp9a
Gpr88
Wdtc1
Rps6-ps1
Kcnc3
Nsmce4a
Nacc1

Rammet
Slc4a3
Snapc1
B4galnt1
Lgmn
Meis3
Zfp141
Ncor2
Cluap1
Dennd1a
Hsbp1l1
Ppp1r9b
Adpgk
Nipal3
Kcnn3
Mef2d
Prss23os
Pisd-ps1
Mrpl21
Prkaca
Sigmar1
Myo16
Pagr1b
Nfasc
Sez6l
Nlgn3
Slc5a2
Htr1a
Trerf1
Fam222b
Celf3
Htatsf1
Pcmt1
Gins2
Kctd17
Nudt16l1
Arfgef2
Snhg6
Hist1h2bk
Ccdc65
Zfp653
Zfp84
Nucb1
Sgms1
Ubb
B3gat3
Gemin6

Ncam1
Golga5
Adss
Zcwpw1
D10Jhu81e
Tmem164
Ehd4
Arpc4
Trp53i11
Per1
Etfrf1
Tbc1d9
Numbl
Sppl3
Snrpa
Polr2k
Ganab
Bahcc1
Fbxo9
Rheb
Ndufa5
Prrc2a
Lrch4
Edf1
Ramp2
Fam45a
Cfap36
Zfp423
Mrps30
Ppp1r11
Txnrd2
Gng10
Pip4k2b
Zrsr1
Gse1
Ilkap
Manbal
Sf3a2
Gria1
Pdcd2l
Cdk16
Tmem269
Ubald1
Sorcs2
Prpf40b
Grin1os
Iqsec2

Casp1
Capn5
Lrp8
Gsk3a
Tmem132a
Cbx3-ps6
Cnot8
Dcakd
Zcchc2
Vps72
Capn15
Grm1
Glmp
Gns
Wnk2
Atp6v0c
Sdhaf2
Pacs2
Arpc1b
Mink1
Fam110a
Rpl13
Rad23b
Zfp691
Phf1
Slc9a1
Sh3bp1
Zfp993
Abca2
Sik3
Mocs2
Tra2a
Ing2
Eif5a
Hcn2
Rogdi
Gabarap
Cacng8
Atpif1
Arg2
Tenm1
Hars
Mbd6
Rgs2
Rnaseh1
Ttll3
Nova2

Cst3
Lrguk
Sarnp
Slc35c2
Psmd14
Mpc2
Nap1l5
Slc39a9
Haus3
Tpd52
Psma7
Syt6
Nemf
Rab5a
Blcap
Mmp24
Plbd2
Pak1ip1
Mrps35
Ctsd
Atp5mpl
Scfd1
Map3k15
Cacna1g
Rrbp1
Sema6c
Nup88
Selenom
Clcn6
Slc25a4
Nat8l
Dek
Zdhhc14
Tpt1
Inpp5j
Tmem248
Abhd14b
Hid1
Zhx3
Rpl13-ps3
Scg5
Zscan29
Maz
Arl4d
Dynll1
Snrpd2
Afg3l1

Crtc1
Gfm1
Sox1ot
Rps13-ps5
Zfp991
Cetn3
Cap1
Stub1
Has3
Cry1
Fam69b
Sec61b
Glrx5
Extl3
Klc2
Rell2
Sema4f
Thy1
Zfp628
Mir3082
Tmem254c
L1cam
Psmd12
Pex7
Tmem126a
Fbxw17
Rpl22l1
Cnpy3
Cox14
Vps52
Rnf7
Phyhip
Fam96a
Ube2e1
Rps27a-ps2
Bex2
Preb
Srsf5
Nol7
Cnbp
Atg7
Fbxw5
Lurap1l
Mospd3
Htra2
BC024978
Rps15a-ps5

Grk2
Lsm12
Tubb2a
Tmem160
Thyn1
Cnih2
Ankrd34a
Prr12
Fscn1
Psenen
Lrrfip2
Stpg1
Lrp3
Magi3
Paqr4
Ciz1
Eno2
Tmem201
Tbca
Sbf1
Lix1l
BC052040
Lynx1
Camta2
Ndufb6
Kdm6b
Spen
Gas5
Znf41-ps
Ptprn
Cfap20
Fancg
Pfn2
Bsg
Grb10
Fam174a
Ddx47
Scrt1
Faim2
Gmpr2
Ndufa12
Anxa5
Ube2s
Atp5g3
Tnrc18
Incenp
Hist3h2ba

		Wdr6
		Mir6236
		Sez6l2
		Csnk1g2
		Trim46
		Syp
		Cdh24
		Actr1b
		Dctd
		Pxmp4
		Vmac
enriched in GAD2	7768	Tnfrsf13b
		Sergef
		Nutf2-ps2
		Dhx9
		Ssu72
		Ruvbl2
		Eif2b2
		Dcun1d2
		Hebp2
		Cep290
		Ifrd2
		Setd6
		Polr3c
		Ube2d-ps
		Pex19
		Eif5al3-ps
		Bnip3l
		Shc2
		Capzb
		Fam205a4
		Trmt5
		Akr1e1
		Snip1
		Npy
		B9d2
		Ap5s1
		Zfp119b
		Atp13a1
		Rplp2
		Hmgn3
		Snhg4
		Gpn3
		Ttll11
		Chrna1os
		11-Sep
		Mettl22

Adrb1
Med4
Hprt
Tigar
Mief1
Gtf2e2
Triqk
Ano2
Ccl19-ps1
Tango2
Kcnh1
Htr5a
Rnf115
Neil1
Hdgf
Chst12
Lzts1
Vash2
Pitpnm3
C1qtnf4
Dnajc19-ps
Sowahc
Nup188
Pqlc2
Rpl35a-ps2
Grk5
Peli3
Alkal2
Pmm1
Pithd1
Pgap2
Dnajc11
Def8
Slc35e3
Ncoa4
Mrps9
Coch
Sharpin
Pbx4
Nup210
Retreg3
Tcea2
Mad1l1
Sgk1
Aig1
Tmsb10
Atp6v1e1

Mir8101
D430042O09Rik
Spata24
Sufu
Hsf4
Asap1
Zscan22
Pcbd2
Emsy
Rpl37
Dda1
Armcx2
Ube4bos1
Otud5
Mzt2
Rps11-ps2
Flywch1
Apex1
Eef1g
Rad51d
Cacfd1
Vamp2
Grem2
Taf5l
Nop9
Rps24-ps3
Rbbp6
Gbf1
Cinp
Ndufs4
Nln
Fam117b
Mrpl38
Ubr3
Dnajc7
Il4i1
Pi4k2a
Aimp1
Rpl24
Phospho1
Mrpl48
Mlf2
Khdrbs3
Zfp703
Fam207a
Larp7
Gbe1

Aida
Rnaset2b
Trmt9b
Hs3st1
Dcaf4
Cmtr2
Zbtb22
Zfp677
Cntd1
Zfp558
Tysnd1
Rusc1
C1qbp
Rasgrp2
Eed
Agpat3
Zfp362
Mpv17l
Icmt
Eef1d
Rgl1
Msl3l2
Cpxm1
Cd33
Llph
Trbj2-3
Ocrl
Stn1
D11Wsu47e
Ppp2r5b
Rbm48
Ckb
Rpl27
Usp13
Tmem196
Hist1h1a
Coa4
Tusc3
Thumpd2
Caprin1
Cops9
Zcchc18
Ndufb3
Plpp7
Rtf1
Dab2ip
Senp3

Pex11a
Cab39l
Tomm40
Dxo
Zdhhc16
Mrpl1
Pim3
C1qtnf1
Cbll1
Ube2m
Trim24
Pla2g12a
Pf4
Bcat1
Rps19-ps2
Cox7b
Tecpr1
Npm1
Mboat7
Bicd2
Ppm1h
Gclm
Ran
Cep63
Rgmb
Mtus2
Tars2
Dbndd1
Grik3
Mir7225
Mcat
Crtac1
Gcdh
Ddah2
Calr
Dmap1
Rufy1
Wasf3
Vsir
Dpy19l1
Cipc
Hist1h2bq
Csnk2b
Nop56
Zhx1
Sfrp1
Tuba8

Suclg1
Prss36
Rpl34-ps1
Xkr4
Isca2
Heca
Paf1
Spats1
Sil1
Fam222a
Tmco1
E2f5
Qtrt1
Fam89b
Fam171a2
Ostc
Avpr1a
Rbm43
Got1
Zfp28
Zfp688
Nelfa
Efs
Srp68
Zbtb17
Akr1c18
Pgm2
Ldah
Gpr3
Gpr137
Zfp760
Tut1
Qrsl1
Tpr
Fam241b
Rybp-ps
Atat1
Stam
Selenop
Gng5-ps
Lsm10
Trafd1
Crip2
Cacng2
Tfb1m
Hist1h3c
Tmem192

Dok7
Zfp64
Srsf3
Prepl
Kcnk6
Hist1h4i
Mir6537
Otud6b
Kbtbd4
Elmo1
B4galnt4
Arl6ip5
Rab2b
Mrps26
Gga3
Prokr2
Dedd
Polr2c
Oser1
Tmem47
Ccdc28b
Hbs1l
Mir6972
Cdca7
Wdr47
Ptpn23
Srprb
Agrn
Txndc15
Rps15a-ps2
Ptpn11
Slc5a5
Ctdnep1
Fam220a
Spry2
Rexo2
Cyp2t4
Zik1
Eif3g
Lig1
Pqbp1
Msh6
Mir7082
Bean1
Nkrf
Camta1
Mir1896

Cux1
Cacna2d3
Hmgn2-ps1
Tomm22
Magee1
Smyd3
Hist1h2ae
Gtpbp1
Sp4
Mrpl48-ps
Snap47
Borcs6
Snhg12
Aldoart1
Zfp386
Nol10
Cox15
AW549877
Coro1b
Pdgfa
Uba1
Pcsk4
Atp6ap1
Mcrip2
Rabl2
Ngf
Fam118b
Ttc39aos1
Tsen34
St8sia3os
Maip1
Fam3c
Itgb2
Hist1h2an
Zfp513
Ostm1
Ndn
Dhdds
Smu1
Man1a2
Samhd1
Pde4d
Nubp1
Tbc1d22a
Slc25a25
Pomc
Nhlrc1

Nkiras2
Copg2
Galk1
Bax
Ube2d2a
Nsdhl
Mthfd1
Ptchd4
Ints1
Psmd5
Pank1
Galnt7
Dpp3
Tspan31
Morc2a
Pak3
Krt1
Bclaf1
Amdhd2
Scyl1
Elmod3
D8Ertd738e
Mfsd3
Iffo2
Dmtf1
Rims3
Tmem86a
Ik
Josd1
Cpne2
Hspa12b
Mir3113
Zdhhc9
Wdr83
Nrm
Nxt2
Cenps
Chmp2a
Dnase1
Zfand1
Htatip2
Rnf208
H2afy
Abtb1
Polr2l
Glra2
Arhgef1

Pgrmc2
Poldip2
Map4k2
Pbdc1
Meis2
Cdkn2aipnl
Slc7a10
Robo2
Fzd1
Epm2a
Lhfpl5
Mir7078
Gtf3c1
Aifm1
Ccnc
Thoc7
Numb
P2ry12
Frmpd4
Necap1
Alg14
Nom1
Tkfc
Mir700
Jade1
Nat8f1
Cdpf1
Tbc1d30
Taf12
Npas3
Fpgt
Lenep
Tubb3
Dtd1
Clptm1
Sox15
Wdr92
Srek1ip1
Nt5c
Arhgdib
Tmem179
Leo1
Rps12-ps3
Ints8
Apbb1ip
Stx6
Wdr36

Alkbh6
Afdn
Morf4l1
Fam104a
Sgcz
Slc38a10
Pigz
Rtp4
Uprt
Tnfsfm13
Taf9b
Epha5
Plekhj1
Prkcz
Txnl1
Panx2
Hira
Arfgap1
Plekhm2
Psmd10
Zfp57
Pgk1-rs7
Pphln1
Polr2m
Eps8
Akip1
Rgs16
Dcst1
Dazap2
Hspe1-ps3
Ccdc189
Rpf1
Prdm16
Mapkbp1
Mettl2
Zfp791
Clcn4
Phf7
Ndrg4
Tyw3
Fancf
Acaa1b
Dip2c
Zfp668
Snd1
Snapc4
Nacc2

Men1
Klhl13
Cand1
Hspa5
Atg5
Zscan2
Rab7
Hnrnpa1l2-ps2
Mta2
Sbk1
Pts
Fmo1
Cdc123
Frg1
Gng3
Rabggtb
Slc12a6
Mmadhc
5-Sep
Exo5
Mybbp1a
Tnfrsf12a
Ints11
Mapt
Cndp2
Atl1
Map7d1
Arhgap6
Cryz
Ammecr1l
Cdk10
Ppa2
Rnf103
Gp1bb
Tmem39b
St6gal2
Pigs
Cacng3
Lias
Ipo13
Bcap31
Nploc4
Mettl26
Eri3
Tollip
Smg5
Rab3b

Zcchc12
H6pd
Alg10b
Cox7a2
Sec61g
Midn
Zscan18
Ss18l2
Pomgnt2
Jmy
Smarca2
Faiml
Retsat
Ppp2r1a
Wdr34
Nudt10
Rps15a-ps3
Large1
Gpr62
Mien1
Pgf
Gpn2
Nol4
Pld5
Mcee
Enkd1
Pdcd10
Efcab10
Upf3a
Tmed7
Kcnq2
Lgi2
Cplx3
Hsd17b10
Trappc4
Kif21a
Pgam1-ps2
Nek7
Cdipt
Sqle
Rps18-ps3
Fkbp8
Acsl3
Isoc2b
Rnf214
Pramef8
Sumf1

Ak1
Rpl18-ps2
Slc32a1
Fam8a1
B630019K06Rik
Mgme1
Sumf2
Mat2b
Igsf11
Pcdh9
Recql
Idh3b
Amz2
Gstp2
Cct8
Phlda3
Rpain
Ring1
Map3k13
Neurl4
Mrs2
Khsrp
Pa2g4
Trp53inp2
Grtp1
Sdhaf3
Pym1
Sh2b3
Pole4
Rmc1
Pnpla2
Cep104
Rabif
Gpi1
Cnot11
Cavin3
Wdyhv1
Khnyn
Abhd14a
Sys1
Abcb8
Znrf1
Pcif1
Selenov
Pcdh17
Minos1
Jsrp1

Gdpd1
Znrf2
Rps2
Tmem151a
Shf
Glrx3
Taok2
Hagh
Tspan4
Nbdy
Pigq
Mapre2
Hax1
Matr3
Dlx6os1
Brd1
Tmem38a
AW822252
Galnt11
Hist2h2ab
Tctn2
Rps7
Sh3glb2
Fbxl16
Ccdc91
Kif2a
Dcaf11
Arrb1
Mthfsd
Gnl2
Aga
Ociad1
Eif3b
Thap11
Ppp2r2c
Acox3
Crnkl1
Cpe
Igfbp5
Ptdss2
Nphp1
Fam50a
Zc3h6
Alg12
Actr1a
Rpl18a-ps1
Rab11a

Rnf167
Gorab
Tmem106c
Dmrtc1a
Gon7
Dap3
Sema4g
Rwdd2a
Cdv3
Gstt1
Prdx3
Limk1
Tstd3
Lrrc73
Lancl1
Zfp414
Eif2s2
Prkar2a
Desi2
Hnrnpa2b1
Rab33b
Zfp524
Nudcd2
Adap1
Pias4
Cops3
Fam32a
Slc45a4
Zfp661
Rcc1
Rpl31-ps18
Rsl1
Adar
Tomm40l
Nosip
Dlg5
Xrcc4
Serhl
Ndufb7
Vamp7-ps
Plvap
Stk25
Pex3
Rps12-ps20
Klhdc9
Tpm2
Spi1

Trpt1
Nsun3
Snrpa1
Hist1h2ag
Adcy3
Tomm34
Fabp3-ps1
Tmem5
Rpl17-ps8
Ppfia4
Pstk
Ccar1
Abracl
Rnf44
Ggact
Ube2nl
Lrp12
Rpl29
Parl
Ndufaf7
Prima1
Gemin7
Cyp4x1os
Clic4
Rad21
Prep
Galntl6
Tgfb1i1
Prmt7
Fam149a
Rab24
Lysmd4
Rtca
Mir7029
Lipe
Cntrob
AW551984
Trpv2
Dnaja2
Cbln1
Mir1895
Fam43b
Lsm6
Kmt2b
Utp11
Hnrnpa0
Dbnl

Anapc5
Dlk1
Bex3
Sybu
Cyb5r4
Gatc
Msn
Rraga
Efna3
Gtf2a2
Dnajb4
Nipsnap2
Picalm
Stx18
Irgq
Itga9
Vdac1
Sipa1l2
Psmc5
Coq7
Ilf2
Sesn3
Zfp318
Pigf
Ankrd13c
Selenoo
Hspd1
Bmper
Ppif
Srrm3
Pheta1
Fam206a
Mcm7
Ufl1
Nup54
Soga3
Kdm1a
Isyna1
Hist1h3b
Oxa1l
Rpl28-ps1
Stim1
Nup107
Glt8d1
Stat5b
Hmgn2
Pcdhb13

Reep6
Atg10
Tex264
Tshb
Hist1h2ap
Trub1
Kars
Nudt2
Mrpl53
Zfp969
Babam1
Rps16
Taf5
Atp5c1
Mtmr2
Cmc4
Ubac1
Terf1
Gusb
Hcfc1r1
Dcun1d4
Zfp874a
Ralbp1
Lin7a
Trmt10c
Mrpl54
Morn2
Reep1
Ppp1r13b
Grk3
Tax1bp1
Clasrp
Got2-ps1
Sec11a
Mrpl12
Tulp4
Ecd
S1pr3
Drp2
Atox1
Mrm2
Phactr3
Slc25a27
AI429214
Pde5a
Ctps2
Fen1

Crat
Uba6
Slc25a47
Fundc1
Crtc2
Ttc30a1
Rtl4
Klhl11
Kyat1
Fsd1
Atxn7l3
Ptpn9
Smim11
Sh2d7
Sar1a
Trim12c
Pxdc1
Cadps2
Rps26
Dctn5
Eaf1
Chadl
Tubg1
Camkmt
Gin1
Asb6
Syndig1
Dolk
Parp11
Gsto1
Nbn
Ptpn18
Sec61a1
Spryd4
Rfxap
D030028A08Rik
Slc35f3
Fam160a2
Prune1
Erp44
Ndufb4c
Ncoa2
Mansc1
Sult2b1
Ubald2
Fhod3
Tigd2

Txn2
Rrp12
Fam98a
Triap1
Mcph1
Iscu
Hjurp
Wdr24
Cpsf6
Rbm34
Ece2
Tfip11
Ccdc47
Rftn1
Slitrk4
Syce2
Cops7a
Ndufaf8
Zfp771
Vstm2a
Slc6a1
Tug1
Kcnt1
Gnb1
Degs2
Ndufs6
Zfp652os
Impact
Ino80e
Amd1
Il34
Rps12-ps13
Nudt18
Gdf10
Rdh5
Psme3
Hdhd3
Cthrc1
Ebpl
Rpl30-ps11
Mhrt
Elac1
Smim19
Ethe1
Sbds
Cit
Daxx

Anxa3
Tspyl2
Klhl33
Ctnnb1
Patl1
Rsl24d1
Rxfp3
Tagap
Carmil3
Acvr1b
Ptov1
Csnk1e
N6amt1
Mis12
Dscaml1
Chrm5
Armcx4
Pcsk6
Trap1
Pcbp1
Epg5
Dnaaf2
Pde11a
Nrg4
Lrrc27
Abcg1
Krt10
Atp23
Hint2
Atrip
Cnnm2
Thoc3
Stk33
Hint3
BC037034
Prkaa1
Btbd11
Urgcp
Fbxo22
Cbx7
Igbp1
Rprd1b
L3mbtl2
Micu1
Tmco3
Strap
Lsm14b

Zc3h14
Crbn
L3mbtl1
Ngef
Chmp6
Brinp3
Sh3kbp1
Selenof
Wdr4
Usp11
Cfap69
Haus7
Lgalsl
Nutf2
Gadd45b
D930015M05Rik
Zfp367
Smim10l2a
Myl12a
Fbxo2
Cntnap5a
Rfx5
Taf7
Inpp4a
Actl6b
Trim26
Cox10
Pfdn2
Sap18b
Dnttip2
Jund
Ina
Cfap126
Zfp354b
Ophn1
Rnh1
Mrps31
Srcin1
Smim24
Abhd1
Slc16a13
Spa17
Taf10
Spg21
Prkab1
Tmem183a
Ap2m1

Sln
Supt16
E2f3
Ncbp3
Ints6
Snx2
Mrnip
Ttc32
Polr1d
Papd5
Cfl1
Tmem200c
Etv3
Ufd1
Pes1
Zfp251
Spef1
Dclk2
Rpl7
Rbm45
Skiv2l
Zfp526
Spindoc
Amn
Ppib
Dnal4
Zfp949
Pitpnm1
Pik3c3
Commd2
Ccdc120
Id4
Selenok
Ctsh
D3Ertd751e
Cdadc1
Ankrd29
Rcn2
Prdx2
Nat10
Tbc1d12
Sap30l
Cwc27
Chic2
Cgrrf1
Ermard
Ncs1

Olfml3
Atp6v0c-ps2
Tenm4
Tfe3
Gle1
Dvl1
Mcl1
Nt5m
Mrpl41
Kcna2
Rsph1
Ptger1
Rnf185
Mir5132
Cercam
Clasp1
Narf
Scp2
Bcl7c
Sox1
Prkrip1
Map3k20
Mrpl42
Mapk3
Vgf
Hsp90b1
Paxip1
Nnt
Cbr4
Uckl1os
Emc9
Phf5a
Srgap3
Zfp811
Rgp1
Fam13c
Arf4
Rplp0
Usp29
Suds3
Ivd
Akap10
Rpl39
Rps19-ps5
Fam205a2
Dnase2a
Zfyve9

Tsfm
Adra1b
Frmd4a
Agap3
Xpo6
Pex26
Fbll1
Rilpl2
Tmem135
Mrps7
Hpgds
Upf3b
Tmem177
Utp4
Bach1
Tamm41
Calca
Lzts3
Flot2
Rps8-ps1
Tgfb3
Mypopos
Zfp316
Fstl5
Bvht
Fgf1
Arhgap27
Dbp
Ebp
Psmb5
Ankra2
Pam16
Lonp2
Spata13
Rpl36-ps2
Nectin4
Gps2
Vav2
Rps6ka2
Grk6
Sfrp2
Neto2
Arhgef7
Nup35
Fam78b
Tmem184b
Aco2

Asic1
C920006O11Rik
Eloa
Nrbp2
Dmac2
Rpl8
Rab3il1
Nog
Pigx
Abhd12
Ap1g2
Tmem14a
Sap130
Samd4
Pam
Epb41l4a
Ano4
H2afy2
Gpr149
Aldh3b2
Pum3
Krit1
Ifi27
Tmem161a
Zfp511
Arfip1
Phyhipl
Leng1
Imp4
Heatr5b
Smarcc2
Mrto4-ps2
Rpl17-ps1
Kcnf1
Ostf1
Zfp59
Ebna1bp2
Rps4l
Coq10b
Inip
Bcam
Gpatch3
Xbp1
Ktn1
Ache
Ntpcr
Rpl19-ps11

Ptbp2
Slc6a8
Nell1
Dpp9
Mthfd2l
Birc2
Smco4
Zdhhc7
Snrnp25
Klf16
D17Wsu92e
Ccni
Shoc2
Stk16
Ndfip1
Zfp623
Lrrc3b
Arhgef11
Zmat3
Adam19
Fxyd6
Bahd1
St8sia6
Ptprn2
Ssb
Alcam
Col19a1
Rnf180
Lamp2
Tdp2
St8sia4
H2-D1
Retreg1
Gtpbp3
Fars2
Meaf6
Ube2v2
Mxd3
Galnt18
Neu1
Arhgef4
Urb2
Ubl7
Csnk2a1
Mettl1
Ube2o
Cplx1

Tmem70
Gfod2
Prdm11
Grid2ip
Epha3
Nono
Stx1b
Stip1
Ski
Elmo2
Aph1a
Mturn
Mir678
Hyi
Gid8
Zfand6
Gabbr2
Ppm1f
Dhx34
Rasgef1c
Prkcsh
Rtl8b
Hexb
Napa
Nkapd1
Agfg2
R3hdm2
Alox8
C530008M17Rik
Gpr75
Mir704
Zscan25
Eya4
Atp2b4
Tmem230
Snrpert
Mir6360
Hmgb1
Rpl30-ps8
Avp
Rnf149
Rufy3
Zfp583
Smarcd1
Zfp598
Gpbp1l1
Pbx2

Avl9
Cltb
Ppp2r5e
Zfp729a
Dnajc24
Tmem163
Fam167a
Gnptab
Serinc4
Sumo2
Prim1
Snapc2
St7l
Cox20
Mir682
Rpl10
Endod1
Socs7
H1f0
Alkbh8
Gpr39
Gaa
H2-M3
Lrrc4
Ddx20
Rpp21
Rpl35a-ps6
Elavl4
Zfp869
Dkc1
Slc39a11
Pla2g15
Tmem42
Vstm5
Mapk10
Lrrc61
Hgsnat
Ccnd3
Hist1h1d
Ankrd13a
Ripor1
Raph1
Unc80
Mir1306
Six3
Mapkapk2
Ywhaq-ps3

U2af1
Mrpl23
Azin1
Slc44a2
Exoc7
Rps12-ps19
P4hb
Yipf3
Fcer1g
Map2k7
Ablim2
Dok5
Snrpb
Gabarapl1
Tmem68
Itsn1
Gpr135
Ube2f
Lair1
Hnrnpa1l2-ps
Ufsp2
Ogfod3
Neurl2
Impdh1
Rbm15b
Psap
Prr13
Frs3
Thra
Cers4
Zfp764
Sfxn4
Alkbh2
Slx4
Psmd3
Gadl1
Trir
Spry4
Fut8
Egr2
Cryba4
Lasp1
Rps12-ps24
Rpl31-ps1
Ptpmt1
Dnajc9
Rpl22

Stx1a
Sntb1
Copz1
Wdr89
Trdmt1
Vdac3-ps1
Spcs3
Tmem9
Kcmf1
Hist1h2bj
Tfpt
Sec62
Ppp1r2-ps4
Pias2
Ibtk
Bckdk
Chchd4
Cdc26
Tmem8b
Rflnb
Mtch2
AW209491
Erc1
Rab1b
Cntfr
Lypd1
Kifc2
Ccs
Syce1l
Tceal9
Tmem241
Zfp553
Mmachc
Pcdhb8
Atp5l
Zfp52
Tax1bp3
Plcxd3
Arnt2
Fndc3a
Rps2-ps5
Nt5c3b
Med21
Hivep1
Fiz1
Ckap5
Rpl7a-ps5

Rab35
Rpl18
Hgs
Cnn2
Inafm2
Slc25a44
Gfra4
Zkscan1
Fkbp14
Apmap
Ptn
Rce1
Lrfn3
Acbd3
Mir1901
B430218F22Rik
Pafah1b2
Crtap
Rai2
Cask
B4galt7
Syn3
Gabrd
Mir8094
Rpl29-ps5
Gnpda1
Acox1
Mgat3
Grik2
Rit2
AI597479
Fam161b
Vkorc1
Specc1
Mycn
Zfp444
Tsc22d1
Tspan6
Zfp784
Rad1
Ppp2r2a
Fam213a
Dhrs13os
Galnt13
Pcdh10
Edem2
Stac2

Plpp4
Ovca2
Uckl1
Rps3
Dlx5
Plcb4
Reps2
Wsb2-ps
Qars
Spred3
Fxr1
Endog
Eif3i
Tmem25
Atp6v1c1
Rbmxl1
Plekha7
Slc37a4
Tspan7
Kdelr1
Brf1
Tmx2
Shd
Prag1
Wdr18
Slc2a5
Rpl31-ps10
Mlx
Celf4
Polb
Lamtor5
Cep120
Zfp212
Bzw1
Wdfy2
Myo1h
Lrp8os3
Tagln3
Eef2kmt
Osgepl1
Rpl36-ps12
Mkx
Nt5c3
Atg9a
Gls2
Serbp1
Thap4

Kptn
G530011O06Rik
Rpl7a-ps3
Bcas2
Tro
Zfp707
Zscan12
Clpp
Tmem147os
Tpm4
Pkd2l2
Trib1
Kcnj3
Tmed1
Ift122
Dynlt3
Gpat4
Arf4os
Cnnm4
Cdk5rap1
Kctd20
Fn3k
Cdc42ep2
Nab1
Guk1
Pex1
Rpgr
Gatd1
Syap1
Adrm1
Comtd1
Lrrc55
Dynlt1b
Taf2
Snapin
Znrd1
Ypel4
Pcca
BC002163
Gtf2ird1
Ddx59
Wdr11
Uimc1
Tmem229b-ps
Myo10
Isg20l2
Serp2

Ube2g2
Susd2
Clba1
Ogdhl
Prkcq
Rab9
Stmn4
Cacnb1
Atp6ap1l
Uck2
Tmed9
Sncb
Lyar
Clptm1l
Ddah1
9-Mar
Slc35b4
Tmsb4x
Arhgap15
Nudt9
Pacsin1
Rps12-ps1
Coro6
Ergic1
Dpp7
Hmga1b
Dgkq
Baalc
Drd2
Vopp1
Ptp4a1
Rps11
Mir3091
Ampd2
Rpl12-ps1
Mvb12a
Eif2b5
Igsf21
AI413582
Hibch
Cox6b2
Gprasp2
Pml
Cdip1
Vbp1
Htr2c
Dcaf13

Acd
Rrp7a
Lrp10
Krt73
Gpr89
AV039307
Spryd7
Ifngr1
Atg3
Vldlr
Rab4b
Hcn1
Zswim4
Ptgfrn
Mroh1
Isg15
Arhgap1
Dctn4
Herpud1
Plgrkt
B230217O12Rik
Snora24
Rec8
Pde1c
Tceal6
Zfp967
Ebi3
Ptcd2
Sars2
Rbx1
Ahsa2
Ppp3cb
Brix1
BC004004
Tm9sf1
Syngr3
Ccdc22
Usp6nl
Atp5j2
Mospd1
Mex3d
Taf9
Syt1
B230398E01Rik
Cyb561d2
Card19
Bmt2

Tsc1
Vstm2l
Kcnc2
Zfp970
Ppp1cc
Impdh2-ps
Dydc2
Zc3hc1
Crabp1
Dvl3
Ubfd1
Snx25
Gemin5
Ddx51
Fads6
Washc1
Aars2
Nrbf2
Akr1a1
Nudt4
Ccdc6
Sp7
Prkacb
Ndufs3
Zc3h12c
Smarca4
B230104I21Rik
Mcm4
Trim66
Ap2b1
Dynlrb2
Ncf1
Usp46os1
Eif4h
Fbxo36
Rfesd
P4htm
Snx19
Asns
Ppfia1
Pced1b
Eif4a2
Oscp1
Pde4dip
Atp5o
Mgll
Rpl31

Pdcd6
Armcx6
Tm4sf5
Zyx
Slc35f1
Rps18-ps1
Nudt21
Rinl
Rgs10
Exosc8
Siva1
Rubcn
Casd1
Klhdc4
Ncoa7
Pdp1
Egfl7
Rab14
Bckdha
Rgs8
Sat2
Selenok-ps4
Sars
Fbxl20
Fam160b2
Pdrg1
Man1b1
Rpl10a-ps2
Kif3b
Unc119
Ino80c
H13
Faim
Me2
Bend6
Ube2e2
Gart
Tbcc
Itm2c
Sdhaf1
Mrpl20
Hsd17b11
Gmds
Morc3
Ier3
Garnl3
Nop53

Cyba
Sstr1
Lrrc45
Slc9a8
Mir6418
Zfp641
Vipas39
Vrk1
Phf8
Zbtb4
Serpine2
Chrm3
Tprgl
Asb8
Trak1
Tnfrsf21
Ptrhd1
Mir6240
Pus3
Fam129b
Exoc3l
Mtfmt
Itgb5
Il6ra
Hist1h2ai
Armc6
Ccm2
Asb1
Rac2
Adcy2
St3gal4
Nucb2
Cpeb3
Ap3s1-ps1
Tmem35a
Atcay
Pabpn1
Klhdc2
Mtrf1
Pip5kl1
Fbxw9
Tfr2
Xrn2
Dtx3
Ptpa
Spint1
Rpa2

Tmem50b
Aldh9a1
Nop58
Zic3
Zc3h10
Gatad2a
Brms1
Helz
Chmp5
Clstn3
Pyurf
Stxbp1
Mir698
Med29
Zc2hc1a
Lsm4
Sardhos
Wbp1
Nsun5
Tcp11l2
Anapc7
Tcof1
Yjefn3
Tcf3
Ifitm10
Supv3l1
Spns1
Cnot7
Edc3
Stox2
Pycrl
Sf3b2
Slc39a6
Lhfp
Gorasp1
Siah2
Repin1
Abca9
Frat2
Zfp804a
Gla
Cntn2
Phtf2
Zfp827
Fam196b
Tpp2
Pkp4

Gstp1
Relt
Klhdc8a
Tmeff2
Pef1
Wars
Cyb5a
Ube2q1
Mmgt1
Lrrc8a
Ifnar2
Ppme1
Topors
Shisa9
Otud1
Rpl32-ps
Ppp1r8
Gng5
Cfap298
Mrgpre
Eps8l1
Lrrtm3
Cdk12
Gnai2
Slc30a6
Wwox
Tbc1d23
Pid1
Ankrd54
Poli
Htr1b
Slc46a3
Pisd-ps2
Tpt1-ps6
Rnf32
Slu7
Cacng5
Zfp422-ps
Ucp2
Ankrd42
Trim7
Rps18
Smg7
Dhcr24
Slc52a2
Anp32a
Ywhaq

Gsx2
Aagab
Runx1t1
Ube2ql1
Mphosph6
Stat3
Mok
Lhpp
Rapgef3os2
Atp5a1
Coq9
Tmem150a
Pik3r2
Nat9
Laptm4a
Eif3d
Camk2n2
Rsph3b
Tob2
Aqr
N4bp1
Bbs1
Psmc1
Maged2
Pih1d1
Fcor
Pdss2
Pde3a
Fdx1l
Ubox5
Fgd3
Cep85
Mrpl51
Zfp110
Arih2
Commd4
Bcar1
Dus4l
Ap1b1
Rnd1
Cdh7
Maea
Hist1h4f
Cul9
Syt13
Gtf3c5
Spata2

Tatdn1
Usp20
Dram2
Ptchd1
Nup50
Snap91
Rap1a
D830030K20Rik
Astn1
Rpl21-ps14
Plppr1
Cyp4x1
Hat1
Ret
Daglb
Lmnb2
Nyap1
Fam117a
Ppp5c
Zfp60
Nsg1
Atg13
Mettl9
Irf5
Mc3r
Tnfaip1
Ctps
Uqcr11
Zfp248
Cox11
Slc38a1
Dus2
Cbr2
Rbm3
Hist1h4m
Camsap1
Tmem53
Gad1
Omg
Hdac3
Yaf2
Pank4
Tmem33
Kat2a
Maf
Rtkn
Bmp3

Arf2
Anapc10
Plxdc2
Clpb
Vps53
Txndc5
Trmt112-ps2
Ttc39c
Mbd5
Psmf1
Cdk5
Sync
Tubb6
Metrnl
Strn4
Mafg
Dnph1
Rpl10-ps2
Ing4
Vps41
Zfyve19
Trmt61a
Chd6
Jkamp
Map1lc3b
Mir7654
Grid1
Fgd5
Metap1
Ccl12
Ankrd9
Slc35a2
Ints5
Polr2i
Pigv
Adam17
Rcc1l
Pcbd1
Ldhb
Pigt
Hnrnpk
Il6st
Slc25a29
Kcnh5
Txndc11
Cystm1
Eftud2

Sv2c
Zfp579
Pdia3
Tspan17
Adamts17
Rrp1b
Vmp1
Syn1
Slc35b1
Mir671
Miga1
Ppp4c
Polr2h
Cadm4
Rpl31-ps7
Drc1
Itpkb
Letm2
Krt222
Fam110b
Sord
Hist1h4d
Rgs17
Pdcl
Ube2e3
Rhobtb2
Rab3gap1
Zdhhc5
Calu
Lmf2
Psmd2
Drosha
Stx4a
C530050E15Rik
Poldip3
Cmtm7
Hspe1-ps2
Iqsec1
Ric3
Hectd4
Hmgb3
Tmtc2
Abhd11os
Klc4
Eif2a
Usp46
Hist1h2aj

Gpx7
Ndufab1-ps
Pi4ka
Mppe1
Zbtb45
Trmt1
Pink1
Med9
Snx7
Yipf5
Ccdc138
Sp8
Mta1
Klf12
Lmo2
Kansl1
Tmem127
Rgs12
B930094E09Rik
Coil
Zfp322a
Aaas
Carns1
Top3b
Pebp1
Uba5
Qpct
Yif1a
Tbc1d17
Arl6ip1
Bet1l
Rpl35a-ps7
Mtfr1
Saysd1
Apeh
Racgap1
Mrpl4
Tubgcp5
Dpm1
Ndel1
Snu13
Scamp1
Mdga2
Prpf6
Foxj2
Rbm19
Asl

Smn1
Rundc3a
Naa20
Aldh18a1
Slc3a2
Ypel5
Ergic3
Hrh2
Ppm1a
Rad51
Abca3
Ntsr1
Cisd2
Ube4a
Chpf2
Wrap73
Cmc2
Fyb
Samd15
Mlycd
Fkbp2
Yipf1
Kat14
Zfp939
Bfar
Hey1
Polr1e
Nup133
H2afz
Zer1
Pcp4
Mrpl37
Camk1
Zfand4
Commd8
Pak6
Ccl22
Idh2
Dhx32
Wdr17
Hist2h2ac
Katnal1
Cyp2u1
Fgf9
H2-T22
Kcnn1
Gtf2h1

Klf5
Rpn1
Kcnd1
Rnf113a1
Msh3
Dner
Dpp10
Abhd11
Dusp22
Fam184b
Mrps17
Pcdhb22
Ctss
Tmem101
Ccl19
Dhodh
Pou6f1
Necab3
Mpv17l2
Itgb1bp1
Sorcs1
Mrps6
Ctsz
Socs5
Mrpl44
Ehbp1l1
Slc7a5
Flrt2
Slc25a19
Tceal7
Cox6a1
Aprt
Sem1
Cyc1
Atp8a2
Cx3cl1
Chrna4
Zfp94
Pou2f2
Trmt12
Vamp8
Capsl
Ppt2
Snta1
Psen1
Cep89
Ikzf1

Sgcb
Znhit1
Tmub1
Gemin4
Mrpl23-ps1
Tmem119
Poc1a
Gosr2
Acad9
Rrp1
Sirt3
Sft2d1
Crmp1
Poc5
Ackr3
Anapc15
Tmem110
Ptpro
Mtap
Fam84a
Nme5
Cuedc1
Aarsd1
Map3k9
Ttbk2
Igfbp3
Edil3
Hexdc
Dnajc15
Kcnmb2
Sgta
Rps6ka4
Cks2
Uggt1
Cir1
Idnk
Atp6v1g2
Smad9
Rab5if
Btg1
Cyth4
Mest
Bpgm
Snx12
Zfp335
Gstm7
Abhd6

Csnk1g3
Nipa2
Atp5g2
Smap1
Tm2d3
Pik3ca
Map6
Phf2
Cc2d1a
Pold3
Dhrs7
Cetn2
Vac14
Rars2
Lsm7
Vamp1
Znrd1as
Cntnap5c
Coro7
Tac2
Akr1b10
Hs3st5
Eral1
Zbtb12
Rapgef3
BC003965
Med19
Fam102b
Tcte2
Rtn3
Rab3ip
Pcdhb19
U2af2
Pgam5
Os9
Dzip1
Fndc9
Gramd1a
Tmem132e
Parva
Dgkk
Fdft1
Tenm2
Fbxo8
Mpg
Irf2bp1
Drap1

Timm8b
Sema6b
Mri1
Adipor1
Spcs2-ps
Gpatch2
Ankrd34b
Cdh10
Ago1
Bola3
Mrpl28
Acsf3
Pak1
Yy1
Hdgfl3
B130034C11Rik
Zc3h4
Ubqln2
Rpl37a
Snrpg
Tmem145
Tsg101
Mrps14
Fyn
Rpl34
Kirrel3
Senp5
Trim28
Cops5
Rpl27-ps1
Cadps
Hfe
Sox13
Sytl5
Gas6
Apoo
Dcaf8
Drg1
Ncstn
Smt3h2-ps
BC029722
Golga2
6-Sep
Fabp5
Zfp354c
Deaf1
Adck5

Slc35g2
Lrfn5
Pmpca
Asah1
Eml3
Inpp5b
Gnb5
Snx4
Smndc1
Pcyt2
Arx
Slc2a13
Ctu1
Rbfox3
Nck1
Plk3
B4galt5
Bap1
Mcm6
Ccdc78
Tmem219
Cutc
Wbp1l
Arpc3
Plekho1
Cntn4
Kctd12
Rpl14-ps1
Ino80dos
Wdr73
Surf1
Slc37a1
Foxred1
Syngr2
Exosc4
Rtcb
Cradd
Dnajb1
Ddb2
Hist2h2aa2
Tuba1a
Psmc3
Med27
Prcp
Pdk3
Cct3
Fbrs

Pdzd8
Sqstm1
Pcx
Slc9a5
C1qb
Chchd5
Tcea1
Slc25a39
Isoc1
Jak1
Brpf3
Kcnh2
Tbc1d22bos
Bcap29
Tbc1d8
Tex9
Rmnd1
Paip2
Rab18
Nip7
Dgcr2
Txnrd1
Cd63-ps
Hnrnpa3
Zbtb2
Arfip2
Spata32
Colgalt1
Havcr2
Srxn1
Rpl15-ps3
Sf3a1
Klhl18
Tspyl4
Gnal
Nenf
Zfp825
Sf1
Rps12-ps16
Vcpip1
Pdcd4
Emc1
Zmiz1
Trim8
Ptpn5
Usp33
Htr7

Hdlbp
Oxnad1
Snx14
Slc25a32
Trpc7
Rbms3
Cidea
Adam22
Prkx
BC001981
Tiprl
Cxcr4
Mir1903
Pno1
Fli1
Cited4
Top2b
Lbh
Alg5
Gpkow
Pcdh7
Nrp2
Prps1
Ccnt1
Fam168a
Tmem50a
Diras1
Akna
Sac3d1
Morf4l2
Acot9
Glp1r
Azi2
Pdap1
Odf3b
Ap3s1-ps2
Cox7a2l
Thap3
C530005A16Rik
Gpr158
Snord42b
Pcgf1
Zfp637
Nanos1
Slc35f6
Tmem199
Rnf121

Fam120a
Asnsd1
Coq8b
Sav1
Abhd17a
Yars2
Tmem176b
Ndufc2
Kpna2
Impad1
Kdm6bos
Zfp358
Dcps
Ttll12
Ormdl2
Anapc2
Nefh
Mfsd10
Cul7
Cdc37
Cebpzos
Fastk
Myo9a
Fbrsl1
Fbxl19
Svop
Ptgs1
Phykpl
Prdm4
Zic2
Ubn1
Tmem28
Mob3a
Exosc2
Mid1
Ech1
Wdr20
Cplx2
Ccdc85b
Atf2
Rps16-ps2
Elp6
Gfra2
Cyld
Hist1h3e
Eif4ebp2
Taco1

Cox5a
Mif-ps3
Vars
Rnmt
Sec24c
Pemt
Armc8
Hmgb1-ps9
Rab42
Mkrn1
Gtpbp6
Exosc9
Syf2
Zfp385a
Cib2
Pcp2
Nrn1l
Sucla2
Gfm2
Cyp46a1
Dnhd1
Arfgap3
Cfdp1
Stac
Hmces
Rpl36-ps3
Mto1
St3gal6
Ifit3b
Zfp7
Rhoq
Commd3
Snrpd3
Prcd
Rpf2
Arfrp1
Ccr5
Eif4g1
Dtx1
Prss23
Dhx8
Zkscan16
Rwdd4a
Ass1
Tacr1
Fez1
Fbxw4

Nudt14
Zfp280b
Afg3l2
Cpsf1
Fntb
Zfp647
Vps8
Ppp1r26
Gapdhs
Sec23b
Sema6d
Avpi1
Gnas
Jakmip3
Rexo4
Jup
Rpl41
Smarcc1
Erlin2
Lysmd2
Nkx6-2
Armcx1
Fkbpl
Tmem256
Nagpa
Smim15
R74862
Eno3
Htr3a
Dlx1
Smyd5
Eef1a2
Mrpl17
Fbf1
Hist1h2al
Uba3
Mfsd6
Slc6a6
Ndufaf3
Ildr2
Cd300c2
Washc3
Tmem189
H2-DMa
Rgs6
Bnip1
Atad3a

Smarca5
Parp16
Chordc1
Msto1
Pla2g6
Pthlh
Hist1h2ac
Ssbp4
Rnf40
Hpcal1
Pou3f4
Csf1r
Srf
Cog4
Hnrnph2
Ero1l
Kat8
Ank1
Hddc3
Ccdc59
Ube3b
Slc5a6
Rnf11
Zfp51
Pip4p2
Ninj1
B3gat2
Golt1b
Tmed3
Slc2a3
Akt1s1
Osbpl3
Tmem41b
Bcl2a1b
Rae1
Cops8
Lrif1
Rnf217
Aspscr1
Fgf18
Tshz1
Magel2
E130102H24Rik
Mocs1
Rpl23
Cd55
Wnt7a

Ift46
Dennd4a
Mfap1b
Gmppa
Mknk1
Atg4c
Caml
Rab11b-ps2
Nxpe3
Cep131
Dym
Scp2-ps2
Chst2
Zfp748
Pwp1
Abr
Gpr153
Slc6a17
Dnajc8
Rasl11a
Thtpa
Erbb4
Fbxo46
Pitrm1
Smg9
Arxes2
Rpap1
Brd4
Gpr61
Tnip1
Slc27a2
Gabrg2
Sntg1
Vax1
Slc2a8
Dpysl4
Nolc1
Tmem14c
Zfp109
Rrs1
Polr3h
Wdr38
Stoml1
Paip2b
Thsd7a
Ctnnd2
Gmps

Cmss1
Sorcs3
Cdc34
Kifc3
Nol8
Cacng7
Mn1
Ddx1
Rrm2b
Pcp4l1
Ddit3
Fgf12
Fibp
Utp18
Stmn2
Tarsl2
Msi2
Rpl21-ps6
Lrrcc1
Nfkbib
Bloc1s5
Tifab
Glod4
Ltv1
Cd2bp2
Nfam1
Naa10
Apoo-ps
AW121686
Fbxw8
Uqcrq
Zfp420
Higd2a
Dync1li1
Phf12
Nap1l1
Sgsm2
Khdrbs1
Gpr84
Tm9sf3
Scand1
Gadd45gip1
Ikbip
Kcnab3
Dohh
Grpel1
Las1l

Tpst2
Tmod3
Gdi2
Trappc10
Fbxl12
Lyn
Noc2l
Sdf2
Senp2
Adck2
Lyrm9
Gfpt1
Ndufs1
Cast
Med8
Galnt14
Rps19-ps6
Lrrc49
Macrod1
Calm3
Nlgn2
Coq3
Rnd3
Mettl18
Slc15a4
Agk
Exoc8
Ssna1
Zmynd19
Gad1-ps
Git1
Fbxl6
Pigyl
Adprhl2
Opa1
Dbn1
Vps33b
Tunar
Gpr137b-ps
Bud13
Krr1
Ypel3
Hsp90aa1
Il13ra1
Pigk
Sh3gl1
S100a4

Rnf4
Ogg1
Bloc1s1
Olfm2
Rpl31-ps9
Zfp454
Btf3
Fam20b
Zfp966
Them4
Polr3gl
Mllt3
Pmvk
Smt3h2-ps4
Plch1
Tmem30a
Brsk1
Pabpc4
Ptrh2
Casc3
Pnpla6
Rims2
Lin9
Elovl6
Mad2l1
Zfp781
Fkbp1a
Hmgb1-ps1
Eif3k
Phpt1
Thop1
Galnt2
Pdzk1
Fam124a
Kalrn
Zfp219
Vkorc1l1
Acta1
Magohb
Icam4
Ajap1
Fam221b
Arid1a
Rora
Aar2
Tsr3
Aamp

Zfp160
Faap24
Ilk
Lyrm7
Med10
Sugt1
Rbp4
Wdr81
Fam19a5
Slc2a6
Zdhhc3
Kcnd3
Zfp330
Spire2
Uhrf1bp1
Dlat
Rps12-ps21
Eps8l3
Eya1
Ldb1
Cd200
Ccdc126
Gba2
Jade2
Tmem206
Rab27a
Mir7666
Cd24a
Cep19
Mtx1
Arpin
Gpatch2l
Cyb5r1
Rfc2
Eif1-ps1
Snrpe
Iqcb1
Rpl31-ps16
Zfp593
Kansl3
Zswim8
Cdkn1a
Rabl6
Sh3bp5l
Mir425
Esf1
Gabrb2

Gnpnat1
Smarce1
Srp72
Ddx24
G0s2
Tmem88
Nme1
Chrac1
Tmem129
Stradb
Ict1os
Slc25a11
Fam131c
Srsf6
Uqcc1
Wdr54
Ppp1r10
Mtfr1l
Tmem65
Mrps2
Tor1aip2
Tmem185a
Tmem151b
Rnf138rt1
Aaed1
Homez
Tcaf1
Rnf145
Derl1
Lrrc71
Ubl3
Tepsin
Degs1
Anks1
Zfand2b
Mrpl35
Casp9
Klhl8
Mrpl11
Ngrn
Zfp846
Slc9a3r2
Rps4l-ps
Fus
Pcdha11
Nupl2
Cnih1

Blvra
Erp29
Rpl35a-ps4
Ecscr
Ube2k
Rpl14
Pcnx3
Hsdl1
Clcn5
Rnpep
Fam192a
Mbd3
Zim1
Epn1
Greb1l
Trappc6a
Saxo2
Rgs19
Rap1gap
Nynrin
Sdhd
Zfp930
Tnnt1
Vma21-ps
Cxxc4
Fads3
Slco3a1
Eif3m
Tor1a
Cnr1
Hist1h2be
Hist3h2a
Lamtor1
Slc39a1
Lrtm2
Pde1b
Zxdb
Tbcb
Ing3
Vegfb
Dnajc5
Ksr2
Jpt1
Hexim1
Hist1h4k
Tceal5
Hmbs

Bcl2
Dlk2
Rab6a
Mrps28
Tbc1d10b
Rasl10b
Lrrc24
Tmem117
Ttc8
Oaz1
Cdc34b
Adnp2
Cars2
Smpd1
Robo1
Csf3r
Pik3r4
Fmc1
Fam3a
Rpl30-ps3
Rrp36
Rpl7l1
Irak1
Champ1
Fbxo6
Cldn3
Dis3l
Plxdc1
Moxd1
Pck2
Tgoln1
Ube2b
Trpc4
Chsy3
Zfp692
Upp2
Rbbp4
Synpr
Rundc3b
Rhot1
AU040320
Polr2f
Nicn1
Gfra1
Atg16l1
Prpf19
Ints3

Twf2
Loxl3
Diaph1
Tnks
Lrp11
Entpd4b
Gchfr
Maoa
Mir6984
Haus4
Map1lc3a
Cenpw
Shisa5
Vps51
Wdr59
Tox
Erlec1
Rab10os
Sv2a
Paqr3
Svbp
Rnps1
Psmd7
Pcyt1b
Sik2
Actb
Supt5
Rps15a-ps4
Eno1
Ascc1
Egr4
Cox7a1
Rarres2
Flcn
Plcxd1
Fam114a1
Zfp865
Fam210b
Gskip
Narfl
Pip5k1b
Rsu1
Gcsh
Slc4a8
Mpi
Coa6
Rpl27-ps3

Dnajc17
Sass6
Adam10
Tpm3-rs7
Slc35a5
Wdr1
Sla2
Hmgb1-ps2
Map3k11
Zdhhc13
Commd1
Spg7
E230016M11Rik
Ndufa13
Dgcr6
Gpr176
Pomt2
Bag4
Bdh1
Noc4l
Gpr101
Wdr45b
Rbl2
Amacr
Pja1
Fam174b
Ifit3
Akirin2
Senp7
Cbx1
Ddr1
Rcbtb2
Ap1g1
Dph1
Memo1
Arid4a
Scnm1
Sh3bp2
Mafk
Enho
Fkbp1b
Snx1
BC035947
Ap1m1
Kcnj12
Cd151
Laptm5

Lmbr1
n-R5s151
Rapgef2
Snrnp200
Rps15a
Mpst
Klc1
Myl1
Npas1
Zdhhc18
Usp19
Spcs2
Dus3l
Qpctl
Hes7
Suox
Mrps10
Tmem147
Eif2b1
Gmcl1
Slc35c1
Pygo1
Pfn1
Ube3a
Rab11fip4
App
Zfp937
Ndufa11
Mapk9
Atp6v1a
Cep250
Rtf2
Minpp1
Rfng
Ccdc124
Cct5
Exoc3
Nprl3
Osbpl5
Bin2
Fah
Tmco6
Hip1r
Cntn6
Gpr137c
Zfp35
Zfp983

Ccdc34
Inpp5d
E2f1
Ranbp10
Commd6
Selenon
Snrpb2
Pdzrn3
Fundc2
Trmt2b
Nup85
Tmem131
Ttc27
Fkbp4
Tmem132c
Slc46a1
Sf3b6
Dynlt1f
Aptx
Zfp53
Rb1cc1
Cog6
Tsnax
Kdm5c
Sms-ps
Mllt1
Mrps18a
Rabac1
Gng7
Cfh
Mras
Cdkl5
Fuz
Tmem107
Rerg
Cox8a
Dmrt2
Snf8
Mrps18c
Wdr13
Rpl35a
Cmklr1
Mrpl47
Slc7a7
Swi5
Kri1
Rasip1

Zfp626
Abcf2
Tcerg1l
Sirt5
M6pr
Tsc2
Ugp2
Ranbp3
Adarb2
Wwp1
Optn
Ccdc167
Irs2
Mb21d2
Zfp119a
Hspa8
Ano10
Clstn1
Ccdc160
Tmem11
Agpat1
Dynlt1-ps1
Tubgcp3
Pcyox1l
Eif2ak3
Kifap3
Rbm8a
Mnat1
Fem1a
Ankrd11
Smarcal1
Psmg4
Ctsc
Gapdh-ps15
C330007P06Rik
Elovl4
Zrsr2
Ngly1
Epb41l2
Mtif3
Epc2
Golga7b
Thada
Pank2
Atp6v0b
Dbi
Hs6st2

Igsf5
Nap1l4
Kcna6
D230017M19Rik
Itpa
Lrrfip1
Nme3
Ano8
Ciapin1
Shtn1
Ddx18
Zfp410
Cd99l2
H2-Ke6
Ldhd
Psma6
Ppp1r3e
D6Wsu163e
Pnoc
Tfam
Psen2
Eef1akmt2
Susd4
Cxadr
Gemin2
Slc29a2
Acp1
Tm2d1
Rps15
Gpr173
Spryd3
Mesd
Lsm5
Fau
Pcdhb14
Zfp709
Ccdc166
Nars
Twistnb
Vamp4
Pgap1
Pcdhb20
Cdk5r1
Bcl2l2
B230217C12Rik
Ppp6r2
Snx10

Eif4enif1
Iars
Pkig
Ltbp3
Vps9d1
Slc25a17
Pomgnt1
Tmem209
Mcts2
Plrg1
Dbt
Mterf2
Armc10
Fxyd2
Frmpd3
Cog1
Mrps36
Fstl1
Parp9
Arhgap44
Hnmt
Pcbp3
Hrasls
Mir7676-2
Ccl25
Fcgr3
Zfp27
Mrpl2
Hnrnpl
Upf1
Ppcdc
Ssbp1
Naxd
Klhl22
Akap2
Gng2
Rtl6
Scmh1
Fdx1
Man2c1
Tefm
Akr1b3
Nop16
Cox7b2
Sympk
Trp53i13
Snrpd1

Ap3s2
Flywch2
D030056L22Rik
Vwa1
Arl4c
Capza1
Snx32
Atp5e
Trbc1
Nectin1
Mier2
Wscd1
Rragd
Mob3c
Dpm2
Asb4
Rnf14
Atp1b2
Hmgcl
Slc18b1
Anxa6
Zkscan14
Scrn1
Psme2
Spred2
Bcar3
Galk2
Tmbim4
Dhdh
Tbpl1
Nudt16
Adcy8
Pdcd2
Fam229b
Cdk11b
Dusp15
Cbx3-ps1
Sparcl1
Arl10
Trmt61b
Scn3a
Znhit6
Rab5b
Ica1l
Nanp
Noc3l
Lrrtm4

Ndufaf1
Mir692-2
Itfg1
Med22
Plekha1
Uchl1os
Cdk2ap1
Kctd8
Lss
Acyp2
Sin3b
Rpl7a-ps8
Entpd4
Btbd2
Ankdd1b
Ftsj3
Xpa
Amd-ps4
Prpsap1
Myo5b
Ccdc127
Zfp382
Rpl30-ps9
Srpk1
Ppip5k2
Timm17a
Cript
Rassf5
Nudt1
Ehbp1
Inpp5k
Mapkapk5
Ebf4
Zfp638
Gopc
Rspry1
Pnck
Gria4
Vwa5a
Efemp2
Bysl
D1Ertd622e
B3gntl1
Scoc
Smim17
Rasd2
Dstn

Ttc13
Gon4l
Zfp422
Dnajb3
Rnf25
Sdr39u1
Lypd6b
Rps25-ps1
Ppp1r21
Ssr4
Dact2
Xrcc5
Mir7010
RF01953
Oasl2
4-Mar
Fbxo25
Mtss1l
Tlk1
Fbxo10
Dip2a
Aard
Gng13
Emc3
Armcx3
Rbks
Grhpr
Extl2
Tars
Mir344g
Gclc
Dmtn
Bag2
Auh
Lgals9
Trio
Cntnap4
Mir343
Anp32e
Cd320
Pnpla8
Ap3d1
Brpf1
Mir21a
Slc43a2
C1ql1
Lypla2

Rdh11
Krtcap2
2-Mar
Mrpl39
Borcs8
E430024P14Rik
Nomo1
Rps11-ps3
Ndufs8
Tbc1d20
Gtf2i
Nhs
Snx16
Dnajb11
Lin7b
Orc5
Prdx5
Smarcb1
Ankrd50
Asxl1
Rnaseh2a
Faap100
Rpl19-ps12
Hpgd
Atp5k
Itm2b
Xylb
Gad2
Pgd
Itga3
Gcc1
Jmjd6
Nat8b-ps
Dennd2a
Ndufa8
Nbl1
Dync1i1
Ier5l
Flad1
Puf60
Rxra
Zdhhc22
Wdr46
Ercc3
Fbxl4
Zfp125
Sf3a3

Dgkd
Emc7
Tmem138
Eif3j2
Faf1
Zfp804b
Fermt3
Hnrnpab
Tmem17
Abi3
Dalrd3
Eme1
Inpp5f
Nae1
Wdr25
G3bp2
Msrb1
Bcl9
AU022252
Castor2
Zfhx2os
Dusp5
Mir3104
Pgk1
Ddb1
Efcab1
Oxr1
Nfkbil1
Hspbp1
Gpx1
Mblac2
Ablim3
Stx5a
Msantd4
Hist1h2bb
Pnma2
Slc36a1
Mtmr4
Etfb
Clu
Pgr
Mettl17
Lhfpl4
Tmem141
Ncoa5
Ttll7
Fastkd1

Fuca1
C230037L18Rik
Gramd4
Ugdh
Tada2b
Atg101
Hhatl
Ints9
Canx
Zbtb5
Syde1
Smc1a
Trim13
Zfp830
Coq5
Lrrc47
Pea15a
Zfp335os
Mir761
Timm44
C2cd3
D430001F17Rik
Emb
Col6a3
Rnf6
Ppie
Abhd16a
Npffr1
Prr36
U2af1l4
Rnf126
Snx21
Cited2
Nol11
Bok
Dlst
Uqcrh
Uqcrc1
Myh6
Scn1b
Wdr91
Pigo
Peg3
Rcan2
Plpp1
Sumo3
Ube2l6

Haghl
Gfap
Pnmal2
Ice1
Unc5c
Rps9
Trim62
Tmem259
Rbsn
Sh3gl3
Rps12-ps15
Taf1c
Igfbpl1
Cpne5
Dlg3
Zfpl1
Cherp
Cd53
Wipi2
Psmb3
Lpin2
Pdlim7
Eml2
Pex13
Camk2g
Trpm4
Ubqln4
Pdia6
Nqo2
Fgf11
Magi2
Pttg1
Mfsd14b
Ppil2
Brd2
Slc25a1
Arl6ip6
Arl3
Nceh1
Rab2a
Srgap1
Tex30
Sst
Habp4
Tmsb15b1
Hist1h2bg
Zar1

Lars
Pars2
Tti2
Vps37c
Slc2a1
Zfp788
Rhbdd3
Sncaip
Ccdc17
Cops7b
Ngdn
Cep170b
Ssh1
Cttnbp2nl
Zmynd12
Akap8l
Eci2
Mid1-ps1
Scyl2
Zc3h8
Rps29
Gpsm3
Dhps
Hist2h3c2
Serinc1
Ccne1
Txndc16
Rnf19b
Zfp959
Nxn
Sephs2
Uqcrh-ps1
Lage3
Mamld1
Rbm17
Zfp667
Mme
Gnl3
Fam96b
Ubtf
Ubl5
Ramp3
Enoph1
Dpysl2
Rpl31-ps14
Clk2
Cdo1

Lipt2
Ccdc58
Ikzf5
Fnbp1l
Igsf3
Rrm1
Endov
Rpl28-ps3
Sh3rf3
Cdc42bpb
Tgfbr2
Fbxw2
Zfp850
Clybl
Zfp746
Tubgcp2
Trim35
Rab8a
Esrrg
Pnkp
Them6
Tgfbrap1
Arhgap5
Tomm5
Bex1
Sugp1
Ppp2r3c
Samd11
Brcc3
Washc5
Ythdf2
Tubb2b
Slirp
Psmd9
Scamp3
Gspt1
Nf1
Dcxr
Syt4
Ssrp1
Usf2
Prmt5
Bloc1s3
Zfp1
AI854703
Snx22
Qdpr

Nppc
Bms1
Zfp952
Tufm
Nckap1l
Hyal3
Unc45a
Rnf31
Nol6
Rida
Tmem97
Cdv3-ps
Uqcc3
Srsf4
Tpbgl
Grip2
Cdc16
Bri3bp
Pafah1b1
Thap1
Ogfod2
Wbp4
Cop1
Dusp4
Sco2
Usp5
Tceanc2
Lmtk3
Yae1d1
Lrrc41
Mtmr6
AI837181
Hist1h2bp
Nek4
Ighj1
Lrrc14
Fuom
Mettl25
Vcpkmt
Ubap2
Mir7019
Cav2
Grsf1
Nt5c1a
Ttc9c
Rpl17-ps5
Scml4

Mpp1
Dr1
Gkap1
Acvr2b
Ube2i
Rab4a
Scn5a
Srrm1
Ube2l3
Cd3eap
Eid2b
Nudt11
Sptlc2
Zfp90
Rybp
Wdr33
Mon1b
Otub1
Zfp853
Sar1b
Mthfs
Ak2
Sarm1
Dctn3
Bscl2
Cept1
Spty2d1
Zfp772
Stk11ip
Fcrls
Hsd17b4
Ripply2
Dennd6b
Psmd11
Glo1
Ntrk2
Dhrs7b
Max
Bicdl1
Glrx2
Slc41a3
Lsg1
Rad54l
Trappc3
Usp46os2
Dctn6
Ggh

Arl6
Odc1
Rnaseh2c
Ddx52
Trim45
Ralgapb
Pcdh8
Snx24
Stk24
Uri1
Irs4
Sox4
Immp2l
Utrn
Stt3a
Frs3os
Necab2
Anp32-ps
Rbm22
Hscb
Cerk
Dzank1
Pole3
Snrk
Adam23
Tmem184c
Fam81a
Pick1
Arl5a
Vangl2
Dock11
Adal
Ppp1r37
Snapc3
Tceal3
Spag9
Prps1l3
Drd1
Cish
Gabra1
Vsp35l
Cstb
Kcnc1
Dlx6os2
Nfkbia
Arl8b
Tfcp2

P2ry13
Gapdh-ps14
Mindy2
Hbegf
Sec13
Fam214b
Vgll4
Glyr1
Fnbp4
Psme2b
Stx8
Xxylt1
Capns1
Fndc5
Kcnu1
Ppp1r14b
Brd9
Nsa2
Moap1
Efl1
Rpp14
Cx3cr1
Ankrd37
Dock3
Ptcd1
Cacng4
Rab3a
Zswim1
Magi1
Mettl27
Dag1
Bcl2l1
Myo5a
Fam49b
Nedd8
Rab11fip3
Rnaseh2b
Rragb
Wdfy1
Smyd2
Med14
Rab11b
Eef2
Ubqln1
Cggbp1
Xk
Szrd1

Atp6ap2
Cckbr
Nudcd3
Cryl1
Cnpy2
Rpl36-ps10
Slc25a33
Lrrc59
Thap7
Gpsm1
Hapln4
Prdx4
Eif2s3x
Hlcs
Sp3os
Fh1
B930082K07Rik
Mrm3
Rabggta
Hs3st2
Ccdc87
Tmtc4
Cct6a
Togaram1
Xrcc3
Sfr1
Mybl1
Ppfia3
Rfc4
Rnf166
Rnf168
Tram1l1
Jakmip1
Mdm1
Plekhb2
Cartpt
Tspan3
Grik1
C2cd2
Cadm1
Mdrl
Trnt1
Slc45a1
Rab34
Creld2
Acbd6
Ttc7b

Xylt2
Ddx3x
Ero1lb
Tvp23b
Sh2b2
Arrb2
Dhrs11
Whamm
Lars2
Zfp521
Flii
Rps8
AI504432
Lrfn4
Trnau1ap
Dhrs13
Sdf4
Slc12a5
Begain
Zfhx3
Pcdh18
Ykt6
Dlgap4
Scn1a
Phf20-ps
Ccdc32
Gne
Tcim
Fancl
Tctn3
Tfb2m
Igfbp4
Cacna1b
Exosc10
Mthfd1l
Unc50
Mtpap
Lemd3
Gars
Ccser1
Phf10
Tab3
Edem1
Fkbp15
Smc6
Tmem262
Rpl31-ps11

Gak
Herpud2
Cmas
Psmc6
Npl
Cenpv
Nus1
I830077J02Rik
Banf1
D330023K18Rik
Ndufs7
Ralyl
Trpc4ap
Atp5h
Hacd3
Fkrp
Ndufa9
Sesn2
Ilf3
Reln
Pag1
Atp5k-ps2
Poll
Alas1
Prkci
Zfp260
Ghr
Rpl22-ps1
Rpl34-ps2
BC051226
Isca1
Rpl26
Rpl31-ps4
Stk19-ps1
Zfp821
Mrpl52
Rer1
C77080
Usp30
Gas2l1
Cyhr1
Csrnp3
Lrpprc
Carmil2
Trappc2l
F2r
Mtmr3

Pantr1
Decr2
Fem1c
Rps11-ps1
Trim3
Zfp961
Stk32a
Wrb
Fastkd2
Nsun2
Rpl23a-ps14
Cope
Gadd45g
Naa38
Anp32b
Tuba1b
Mfsd12
Trmt10b
Mapre3
Rbx1-ps
Emc8
Siah3
Ahcy
Far2
Mir6244
Ndufs2
Arsa
Ghitm
Mtg1
Strip1
Sms
Dhx37
Bmp2k
Uros
Elavl2
Pifo
Taf6
Eif4g3
Qrfpr
Zfp617
Afmid
Akap5
Pet100
Tbccd1
Taf13
Sat1
Imp3

Kcnk1
Phyh
Rps19
Srrm2
Nfatc2
Traf3ip1
Mus81
Itfg2
Kmt2e
Dusp12
Srp19
Mir6903
Ubxn6
Rps15-ps2
Cdh2
Vdac2
Ece1
Pdcl3
Chsy1
Leng9
Chmp4b
Nfyc
Gmppb
Tmem191c
Glis2
Nr1d1
Anapc4
Rnf146
Tmem150b
Zfp467
Hspd1-ps3
Cnot9
Ddt
Cacnb4
Slc39a3
Wdr41
Bphl
Hist1h2ao
Psmb10
Dnmt1
Gpalpp1
Smg8
Mir692-3
Npb
Zfp213
Agtrap
Trmt1l

Armc7
Ggt7
Ss18l1
Traf7
Sos1
Slitrk1
Chrm4
Mitd1
Cfap300
Nudt19
Dnaja3
Fbxl21
Mxd1
Foxp3
Cort
Vezf1
Phkb
Chmp7
Gdf11
Tmem205
Ccar2
Fam185a
Usb1
Cdc42ep3
Epdr1
Ell2
Tmem246
Smim18
Tnfaip8l3
Cct7
Wwc2
Ppp1r2-ps6
Ttc30a2
Sdsl
Slc9a7
Csdc2
Dmwd
Vat1
Tdpx-ps1
Zfp580
Plcd3
Ppard
Rmi1
Slc14a2
Trim23
Zfp426
Mrpl13

Klhdc3
Pik3r1
Grpr
Rpl21-ps13
Scamp5
Taco1os
Luzp1
Nisch
Phospho2
Jtb
Rpl17-ps9
Rpl10a-ps1
Maged1
Fip1l1
Osbpl7
Odf2
Rhof
Bri3
Ano1
B4galt3
Rnf130
Cda
Klhdc1
Fyttd1
Kat7
Nipsnap3b
Zfp346
Mir22
Entpd6
Mtrr
Klf8
Nhp2
Smim8
Oxld1
Fam219a
Exosc5
Tmem234
Emilin2
Med25
Cbfa2t2-ps1
Brf2
Lrrtm2
Slc35f4
Polr3b
Pde6d
Scn2b
Fam98b

Mvk
Mapk8ip1
Clpx
Itgb3bp
Sstr2
Ddx25
Ints14
Rhoa
Rrp15
Trem2
Kctd2
Rps12l1
Efna5
Asf1a
Iah1
P2ry6
Dapp1
Arl6ip4
Zfp114
Col25a1
Tnpo2
Ccdc90b
Dbndd2
Larp1
Rps6ka6
Polr1c
Lgr4
Mlst8
Gps1
Sdc3
Rps10-ps1
Zfp398
Rad51c
Calb1
Amt
Rps17
Pdcd7
Aatf
Ptger4
Zbtb7c
AK157302
Fndc4
Rere
Zscan21
Ocel1
Wdr53
Metap2

E130311K13Rik
Mul1
Agpat4
Rpsa-ps11
B3galt6
Slc3a1
Abhd8
Rps2-ps6
Ube2d1
Suz12
Dnajc4
Mff
Lrrc28
Ftsj1
Tomm20
H2afv
Yrdc
Asic4
Aimp2
Noa1
Zbtb48
Cyb561
Panx1
Nrbp1
Ccdc92b
Armcx5
Emc10
C9orf72
Kcna3
Kcnh6
Pxylp1
Zdhhc1
L2hgdh
Epb41l3
Tfg
Fam205a3
Psma3
Cep57
Psmg1
Vps29
Eif2b3
Lsm3
Mir5133
Golph3
Zfp512
Polr3d
Trp53rka

Ulk3
Rcor2
Cyb5d2
Nfkb1
B9d1
Axin1
Pex16
Nudt22
Sncg
Dvl2
Copg1
Bcl7b
Ptgr2
Snhg3
Mettl23
Pgm3
Atp13a2
Carm1
Mtag2
Zfp651
Gsr
Mecp2
Rpl36
Sult4a1
Nop2
B3gnt4
Ptpn4
Gpr162
Whrn
Zfp882
Xpo7
Car15
Cyp4f16
Zfp759
Git2
Ndufb10
Aldh1b1
Inha
Actg-ps1
Rhbdl3
Ly96
Rapgef3os1
Dnajc10
Ndufb4
Rps6kl1
Pdhx
Mapk8

Mgat1
Arl2bp
Adgra1
Terf2
Mtus1
Nme2
Trim36
Rbm42
Elof1
Lyrm1
Cln8
Fam136a
Ncoa3
Cul4b
Fam173a
Wasf2
Thoc6
Ttll5
Hist1h2br
Gucy1a1
Rhbdd2
Tmem143
Zfp518b
Spata25
Klf6
Synj2bp
Tdrd7
Zfp790
Psmb6
Slc36a4
Rsrc2
Secisbp2
Zbtb21
Prickle4
Rimbp2
Slc19a2
Map4
Lztr1
Blzf1
Mllt6
Mrpl9
Sfxn1
Ip6k1
Cmip
Fbxl2
Bin1
Pkib

Fcgr1
Lamb1
E2f6
Srgap2
Elk3
Hspb8
Mrps18b
B3galnt1
Doc2g
Vip
Ptprt
Mrps34
Hdac6
Pdyn
Napg
Mfng
Ppp2r2d
Rnaset2a
Wdr70
Zfp629
Tbc1d13
Spr
Ankmy2
Paics
Zfp13
Fbxo21
Pcid2
Rtn1
Nutf2-ps1
Bub3
Umps
Rsph9
Plp2
Tnfaip8l2
Zfp319
Zfp768
Hist1h4a
Cxcl14
Rpl10a
Rbbp7
Rgl2
Foxg1
Iqce
Slc30a9
Sp140
Slx1b
Cd180

Rita1
Cdkl1
Spock1
Adprh
Tmem178
Dap
Itgam
Ift140
Coa3
Simc1
Hmgb1-ps4
Myl12b
Bbs2
Lrfn1
Eapp
Abcd1
Ndufaf6
Zfp658
Josd2
Hist1h4j
Prmt8
Tacc2
Pfdn5
Rps27
Pim2
Slc7a6os
Arf5
Tbc1d16
Dnm3
Ptprm
Acat1
Zmat2
Coa7
Lyplal1
Rbfa
Srpr
Tpi1
Atxn2l
Tmem115
Ap2s1
Rhot2
Polr2e
Dscam
Pkm
Pomk
Spcs1
Arhgef12

Hist1h2af
Hpf1
Rgs20
Stk19
Pdia4
Ttc1
E430018J23Rik
Ift172
Pias3
Borcs7
Fbxo3
Zfp275
Slc25a35
Chid1
Egr1
Ephb2
Elmod1
Zfp235
Dynlt1c
Psmd6
Paxx
Rnf141
Nubp2
Ppp1r2-ps1
Zdhhc8
Matn2
Sema3c
Cnbd2
Shc3
Usp51
Mrap2
Serinc3
Mafb
Fbxo41
Mthfd2
Commd10
Erh
Mea1
Zmynd8
Dazap1
Slc25a26
Gnpda2
Ldoc1
Eif2ak1
Lamtor4
Zfp36
Osbpl2

Tceal8
Fam57b
Snrpc
Mrpl24
Snrnp27
Nrip3
Dusp8
Ext2
Mfn2
Fhit
Ptges3
Alg2
Cdk9
Tmx1
Mbtps1
Pgam1
Chmp1a
Pik3cg
Arhgef40
Nprl2
Mir8099-1
Ripor2
Slmap
Usp42
Adarb1
Kbtbd3
Wbp2
Tmem69
Negr1
Rpl17
Sri
Zfp3
Ess2
Map4k3
Zfp523
Gpr4
Uchl5
Prelid3b
Fitm2
Ing1
Setd7
Adssl1
Ablim1
Cd164l2
Mrpl32
Chn2
Chchd1

Srsf7
Ppp2r5d
Dok4
Cdr2
Zfp575
Polr2d
Klhl14
Maneal
Ephx4
Tpgs1
Dcaf12
Pld4
Tnrc6a
Zbtb41
Fbxl15
Myg1
Zfp672
B4gat1
Rbm7
Hist1h1c
Wdr48
Plppr5
Pard6g
Rnf114
Tkt
Uso1
Snx3
Phf6
Arhgap33
Gramd2
Txndc9
Apoa2
Ccdc115
Timm29
B4galt6
Tmem134
Zfp65
Adra2a
Saraf
Acaca
Rbm14
Cul4a
Psmd13
Ccny
Nme4
Clec2l
Nkain3

Cisd1
Mir331
Lcp2
Vapa
Trmt2a
Anapc11
Tmem169
Timm8a1
Cdc42
Stard10
Smim20
Fam169a
E530011L22Rik
Nle1
Slc25a3
Arfgap2
Gnl3l
Ctbp1
Med20
Gap43
Sumo1
Mbd2
Dph7
Clcn3
Rpl35a-ps3
Ube2j1
Tmem208
Ddx23
Ndrg3
Nr4a1
Zfp608
Cux2
Lrpap1
Nalcn
Ambra1
Rps12-ps6
Alkbh5
Pard6a
Pcdhb7
Nsmce1
Ahi1
Mrps11
Atp5b
Parvb
Depdc5
Cryzl1
Gba

Ehmt2
Tssc4
Fam220-ps
Fxyd7
Osbpl9
Pcdh11x
Rarb
Slc16a14
Retreg2
B3glct
Ube2r2
Hps4
Clic1
Lmna
Cdc14b
Chmp2b
Sh3yl1
Sgtb
Ywhab
Capn2
Timm50
Atg9b
Map3k12
St6gal1
Rpl10-ps3
Mtif2
Tmsb15l
Btrc
Tubb4b-ps1
Tmem62
Arl4a
Lyz2
Ppcs
Sec31a
Tmem215
Golga7
Orc6
Azin2
Lgals1
Tdp1
Zfp449
Plekha3
Eif4e
Ftl1-ps2
Madd
Raf1
Pxk

Cacna2d2
Cacna1i
Vasp
Sdhb
Abl1
Zbtb9
Osbp
Zfp706
Akap17b
Siglech
Dis3l2
Cnpy4
Nagk
Bcas3
Rbm10
Pwwp2b
Pacs1
Inpp5a
Tapbpl
Bak1
Iqsec3
Tsta3
Stat2
Ttpal
Zmat1
Rnf152
Mmab
Prkar2b
Rps12
Fuca2
Pde10a
Sdad1
Pex2
Prpf31
Rab3c
Dld
Nit1
Riox1
Sugp2
Casp8
Ift57
Cmtm3
Rpl3-ps1
Ube4b
Slc1a6
Gtf2ird2
Eif1

Tmeff1
Med28
Kif21b
Ssbp3
Ppil3
Cnot10
Tbc1d9b
Igsf10
Nr2f2
Pms2
Tagln2
Tmed2
Ifit1bl1
Ddx21
Calm1
Cdh8
Hspa1l
Rpl36a-ps3
Tapbp
Tent5a
Pafah1b3
Rab36
Atp8b2
Ier3ip1
Rtl5
Trub2
Wdr75
Plekha6
Nlk
Pdhb
Sla
Ndufb8
Acbd4
Ttc17
Rnf8
Jazf1
Pde3b
Xrcc1
Selenos
C430014B12Rik
Acaa1a
Mxra7
Tcerg1
Eepd1
Psmc2
Dlx6
Tbc1d7

Brox
Snw1
Mdh2
Dhx29
Mir6973b
Ntng1
Glce
Ppp2r2b
Dchs1
Abcb9
Zfp868
Alg9
Lbhd2
Hist2h4
Zfp105
B130046B21Rik
Gale
Cops6
Tpra1
Prcc
Senp6
Tcta
Tmem186
Tspan5
Rad9a
Crkl
Ppp1r12c
Vrk3
Tmem240
Lnx1
Rps12-ps9
Zfp622
Rpl37rt
Cyp51
Fam171a1
Cdk20
Ufsp1
Raver1
Nol3
Rfk
Bad
Clip3
Fgf16
Zswim3
Ap2a2
Gpr83
Tmbim6

Cables2
Medag
Fgd1
Gar1
Ptcd3
Cd63
Cspg5
Ogdh
Pgam1-ps1
Desi1
Reps1
Rit1
Pcyox1
Tinf2
Wee1
Zmym3
Sox2
Zfp944
Swap70
Enpp5
Ndufa11b
Kctd21
Cyb561a3
Rpap3
Araf
Polr3e
Mvb12b
Ddx50
Prorsd1
Pdcd6ip
Cnot2
Pced1a
Ncf2
Kcng4
Pecr
Tlnrd1
Hist2h2aa1
Dusp10
Brd7
Setd1b
Skiv2l2
Ankrd26
Tmx3
Dpagt1
Armh3
Agap1
Ints12

Klhl26
Hgh1
Uck1
Rnf26
Tm9sf2
Xpr1
Lrrc75b
Mad2l1bp
Slco2b1
Zfp958
Mgat5b
Otulinl
Ccdc9
Etaa1
Prkar1a
Eif2b4
Hes6
Rnft2
D730003I15Rik
Synj1
Aco1
Zbtb14
Taldo1
Dlc1
Ofd1
Hspa4
Dleu7
Zfp770
Cdk5rap2
Riok1
Sgsm3
Srrd
Top1
Fut11
Lyl1
Gstm5
Sulf2
Slc22a23
Dgat1
St8sia5
Eif3a
Nr1h2
Ppp1r15a
Mir7000
Fam91a1
Disp3
Trappc2

Adat2
Hdac11
Smim7
Ankfn1
Fam133b
Fubp1
Chst10
Aldoa
Ankrd35
Acot8
Mir8104
Spx
Zfp995
Snupn
Ash2l
Tbl1x
Pgls
Tom1
Chrm2
Rab13
Hsd3b3
Amd2
Tmem202
Exog
Pcdhgc4
Akap7
Myo18a
Cntnap2
Srsf2
Vat1l
Ndufaf4
Timm22
Npepps
Edc4
Hist1h2ah
Rhov
Wdr37
Ccdc136
Xpo5
Hist1h2ab
Rpl30-ps10
Dnajc28
Glt1d1
Selplg
Hccs
Arhgef6
Dync2li1

Kat6a
Churc1
Ciao1
Rassf8
Tcea1-ps1
Zcchc10
Ap3m2
Plcg1
Rpl36al
Phlda1
Usp36
B230364G03Rik
Eif1ad
Csnk1d
Bhlhb9
Il2
Rpl31-ps17
Sec22b
Pogz
Igdcc4
Mzt1
Entpd1
Prkcd
Rnase4
Exosc7
Vwc2
Oxtr
Gnaz
Mast2
Ldha
Sdhaf4
Grcc10
Cul1
Kif3a
St3gal3
Rpap2
Eif4a1
Cldn12
Slain1
Isoc2a
Lrch1
Med30
Rpl32
Slc25a28
Pdcd5
Ndst2
Smim12

Cenpt
Ywhag
Rps12-ps12
Unc93b1
Grin3a
Hsd17b12
Egfl8
Hnrnpul2
Nif3l1
Fam71e1
Surf6
Sec61a2
Gnb4
Gdap2
Rpgrip1
Dcbld2
Naglu
Atp5l2-ps
Rcan3
Mcub
Slc25a46
Fam122a
Tbcd
Smad2
Vwa8
Nr1i3
Raly
Tbc1d25
Trappc9
Pdzd2
Arxes1
Hist1h3a
Foxr2
Rpl19-ps1
Trpc1
Mrpl50
Evi5l
Srrm4
Crk
Atp6v0d1
Polr3k
Tpt1-ps5
Dennd4b
Rpl21-ps3
Sgip1
Dpf1
Ccdc153

Hacd1
Insyn1
Hilpda
Hdhd2
Rtl10
Baiap3
Rsph3a
Ndufs5
Tmem175
Ino80b
Dynll2
C330018D20Rik
Akr7a5
Parn
Nudt3
Resp18
Pgrmc1
Asna1
Reep5
Uap1
Pfkm
Ltc4s
Ccna1
Wdr12
Ormdl1
Ptch1
Tmem94
Ccdc12
Ei24
Kpna3
Rab43
Elac2
Fbxo28
Rp9
Arhgef10l
Ckmt1
Galnt1
Eif1a
Osbp2
Lpcat1
Urm1
Pcsk7
Stk11
Gripap1
Fxn
Mtmr14
Fam168b

Tmem126b
Zmynd11
Mrm1
Cyb5b
Ddx41
Fam189b
Gfpt2
Bmyc
Zfp655
Fam196a
Bola1
Ifi27l2a
Gpr27
Inafm1
Wdfy3
Cmpk2
Kcnk3
Gorasp2
Ndufv3
Zfp800
Rsbn1
Mmp15
Nsun4
Ube2q2
Mpeg1
Hsf2
Cybc1
Mir30d
Csk
Phtf1
Rgs7
Zfp955a
Klhdc8b
Amd-ps1
Ankrd55
Hist1h4n
Cyth2
H3f3b
Rps23
Elp2
Ppat
Pgp
Mrps21
Gng4
Malsu1
Pcna-ps2
Bend4

Lrrc8b
Nipsnap1
Cpsf7
Taf8
BC030500
Khk
Cdkn2d
Acbd7
Vps16
Wdr43
Ubl4a
Cdk13
H3f3a
Rnf13
Nanos3
Ifnar1
Rps10
Ppt1
Dctpp1
Mapk6
Nat14
Haus1
Samm50
Sfxn3
Slc35e4
Map2k4
Cox7c
Adgra3
Zpr1
Stx17
Ttc33
Usp8
Alox5ap
Cwc15
Rsl1d1
Gnai1
Sema4a
Arel1
Fosb
Etv6
Rom1
Nmnat2
Zfp560
Uvrag
Ndufb9
Nelfb
Klhl29

Foxk2
Zadh2
Commd5
Osgep
Klhdc10
Rpl6
Acp4
Bpnt1
Map3k4
Pdzd11
Hyal2
Nnat
Exoc1
Ptpra
Fbxl14
Fchsd2
Nufip1
E2f4
Slc23a2
Fam120aos
Foxn3
Mypop
Ly86
Prmt2
Prr7
Dguok
Cd83
Med6
Kcnc4
Bcl10
Papd4
Vps36
Mrpl49
Dgkg
Celf6
Prdx1
Zfp317
Izumo4
Dnm1
Set
Zfp712
Ptprr
Gyg
Zmat4
Rps12-ps11
Spsb4
Supt3

Cbx8
Eva1c
Fzr1
Tm7sf2
Ccdc38
L3mbtl3
Pycr2
Klhl32
Cby1
Ivns1abp
Zdbf2
Epb41l4b
Zc3h15
Terf2ip
Tmem91
Mpnd
Rph3a
Galns
Osgin2
Rps25
Ctnnbip1
Pex11b
St13
Ppan
Flot1
Gadd45a
Cdc37l1
Cse1l
Haus8
Slc25a14
Mief2
Lsm14a
Cks1b
Nub1
Bnip3
Pdlim3
Dnttip1
Cdkl3
Rpl3-ps2
Hmgb1-ps7
Platr17
Npas4
Chl1
Trhde
Ulk2
Slc39a7
Wdr5

Atp6v0a2
Iqcc
Tmem60
Zfp777
Lonp1
Khdrbs2
Mtf1
Srp54c
Ugcg
Znhit2
Necap2
BC031181
Sart1
Masp1
Asic2
Pspc1
Scly
Rps2-ps4
Nmt2
Bag1
Btbd19
Ccdc82
Zfp512b
Anapc16
Trim27
Prune2
Znfx1
Tubgcp6
Gtpbp4
Arhgap25
Cdh20
Btf3l4
Neto1
Wfdc18
Mecr
Dedd2
Rpusd1
Lsm1
Vta1
Sez6
Zfp810
Tspyl1
Zfp341
Hist1h1e
Timp2
Txndc12
Ankrd17

Marcksl1
Atp2c1
Mtrf1l
Clmp
Larp6
Smarca5-ps
Babam2
Pdzd4
Rdh13
Clasp2
Bicral
Arhgap26
Abcb10
Park7
Chtop
Zbtb8os
Gnpat
Dnajc12
Otulin
Mrpl30
Slc33a1
Tprn
Sub1
Btbd6
Yeats4
Rps24-ps2
Mrpl40
Mex3c
Dcaf1
Sowahb
Epb41l1
Zfp689
Ppp2cb
Creb3
Kcnab3os
Slc7a3
Fam177a
Usp10
Tmem263
Rnf123
BC005624
Ubap2l
Pcdhb15
Phb
Cnnm3
Aen
Sirt2

D330041H03Rik
Atraid
Psma1
Rpl36a-ps1
Rplp1
Sobp
Jagn1
Rpl38
Cd164
Rnf181
Vcp-rs
Csrp2
Coasy
Slc18a2
Oat
Zfp931
Apbb1
Brinp2
Rnf219
Mir147
Hmox2
Pex14
Zfp607b
Fam13b
Selenok-ps1
Ikbkap
Sidt2
Cds1
Surf4
Lzts2
Vma21
Rps19-ps4
Fam120c
Fam205a1
Vash1
Akain1
Crhr1
Alkbh7
Wipf2
Dhx35
Pelo
Lrwd1
Aifm2
Smoc1
Ddx28
Prim2
Lrsam1

Msl3
Ift74
Eif4a3
Pigp
Taf11
Rps26-ps1
F8a
Dnajc19
Armh4
Abraxas2
Rmdn3
Chp1
Vps45
Esd
Tpm1
Xpot
Comt
Hexa
Unc119b
Rnf128
Zfp719
Gys1
Dffa
Dapk1
Gabbr1
Clcn1
Rpp40
Cbwd1
Hps6
Nxph1
Ankrd45
Rimkla
Rps4x
Rbmx
Anapc13
Snx5
Rab33a
Sae1
Snap25
Tceal1
Zfp740
Pnpo
Dglucy
Afg1l
Ptdss1
Fhod1
Zfp61

Ptpdc1
Phf23
Chml
Snrnp40
Chac1
Gpr12
Pdik1l
Pnrc1
Oxsm
Sh2d3c
Sap30bp
Csnk2a2
Rims4
Selenoh
Atf6b
Nptn
E130308A19Rik
Slc1a4
Armc1
Pinx1
Zfp143
Hirip3
Cmtr1
Glrx
H2afx
Ndor1
Gtf2f1
Cars
G3bp1
Gpatch4
Rbms1
Entpd3
Praf2
Pelp1
Zdhhc6
Dyrk1a
Ngfr
Apip
Slc6a7
Enox2
Mir705
Arhgef3
Pak4
Plxna3
Slc22a17
Tctex1d2
Chchd3

Sel1l3
Gnao1
Klf15
Zkscan4
Tesk2
Sertad4
Ccz1
H2afj
D330050G23Rik
Sbsn
Trabd
Tonsl
Thumpd1
Satb1
Siae
Stx7
E130317F20Rik
Snhg20
Nckap5los
R3hcc1
Cox18
Pias1
Efnb1
Sorbs1
Ube2v1
Bzw2
Rpl31-ps20
Adsl
Rpl38-ps2
Ap3b2
Phactr2
Ankzf1
Hnrnpa1
Taok3
Psmd4
Actr6
Bcl2l11
Ppargc1a
Actr8
Mir5617
Wdr55
Zfp941
Tbc1d22b
Ppp1r2-ps3
Sh3bgrl2
Coro1c
Borcs5

Sssca1
Ggps1
Rbm3os
Scg2
Fbxo44
Hist2h3b
Sccpdh
Tma7
Rtl8a
M6pr-ps
Gab2
Bag6
Zfp618
Lpin1
Pin1
Pcsk1
Fmnl3
Cd276
Ywhaq-ps2
Cenpa
Ttc3
Kazn
Rab1a
Dynlt1a
Zfp334
Dnajc6
Ruvbl1
Dnajb9
Cep135
Ccl27a
Med16
Rab21
Katnal2
Lcmt1
Dgcr8
Mrpl22
Hist1h4h
Scaf8
Vstm2b
Ccnh
Zfp971
Ift20
Cck
Slc48a1
Prkag1
Tmem43
Alg3

Rpl11
Adgrl3
Spout1
Hspb6
Tymp
Sphk2
Ctc1
Rps12-ps18
Zfp934
Syt12
Rpl21-ps8
Cdk5r2
Lamp1
Steap2
Hunk
Nckipsd
Thrb
Ntm
Pde12
Dnaja1
Ccdc28a
Ggnbp1
Sgpp1
Slc29a1
Ifrd1
D130043K22Rik
Prr14
Fdps
Plppr3
Romo1
Rpl17-ps10
Ficd
Polr2b
Kat2b
Yipf4
Cers5
Mfsd1
Zfp763
Uchl4
Rpl19
Ecel1
Fopnl
Rmnd5b
Pcsk1n
Cntnap3
Fam120b
Copa

Npdc1
Shroom2
Rps19bp1
Gpr165
Gphn
Sra1
Mrps22
Spdya
Tmed10
Tmem19
Zfp968
Rif1
Gpr137b
Flvcr1
Tbc1d24
Nfx1
Nav3
Tmem8
Xntrpc
Mbnl1
Adap2os
Msmp
Olfm3
Faf2
Abcd4
Cabp1
Tspoap1
Kcns3
Atp5j
Ndufa1
C1qtnf12
AW554918
Tmem158
Rnd2
Pigb
Mrto4
Rnf187
Abhd17b
Tsen15
Ndufc1
Setd3
Kbtbd7
Impdh2
Immt
Slc39a13
Tmem104
Mrpl57

Slc38a7
Tmem218
Dis3
Mblac1
Emc4
Egfr
Urod
Lypla1
Stk40
Hist1h2ak
C1qa
Atp5d
Ier2
Cct2
Rpl13a
Idh3g
Dirc2
Cox4i1
Ppp1r2
Glrb
Chchd6
Cds2
Calb2
Prpf38a
Gtf2b
Ythdc1
Mrgbp
Fam58b
Exosc1
E130218I03Rik
Rnf24
Cnmd
Orc3
Rbm8a2
Itpk1
Lrrc8d
Flrt1
Donson
Rab12
Kxd1
Pacsin2
Tma7-ps
Hmbox1
Pdzd9
Clint1
Rab9b-ps1
Eif3c

Paip1
Clec16a
Det1
Mdp1
Dcaf7
Cbln2
Nudt15
Nr2c2ap
Doc2a
Srfbp1
Pcdhb6
Slc30a5
Snx15
Ywhae
Aggf1
Myc
E130307A14Rik
Dpp6
Gatad1
Ctsf
Fez2
Mnt
Pou3f3
Mapk1ip1l
Nnmt
Zmat5
Arc
Scpep1
Llph-ps1
Trip4
Med31
5-Mar
Hddc2
Mrrf
Atmin
Siglece
Nudc
Zfp128
Bptf
N4bp2l1
Ndufaf2
Mettl21a
Mgat2
Tmem132b
Klhl28
Crybb1
B3galt4

Serpini1
Gpbp1
Cstf2t
Tpm3
Eif2s1
Nrg1
Tmem229b
Mpped1
Srp14
Wasl
Cant1
Mustn1
Adprm
Pcgf2
Uba2
Trp53
Ctnna2
Rimklb
Bloc1s6
Hmgb1-ps3
Pisd
Arvcf
Angptl6
B230219D22Rik
Pik3r5
Cables1
Hnrnph1
Olfr1344
Tada1
Tpst1
Cdr2l
Kif3c
Ccdc181
Parp8
Taf1
Rasd1
Srp54a
Zic1
Psmc4
Vapb
Rpl27a-ps1
Gpr25
Mrpl14
Map2k5
Glb1
Cdh6
Sec24b

Fos
Mkrn2
Cdc42ep5
Smim14
Med26
Ift22
Fam92a
Nrxn3
Zfp385b
Atic
Ppid
Megf8
Scx
Fhl3
Tasp1
Tbl3
Gpatch11
Atp1b1
Sh3bgrl
Ybx1
Arhgap24
Nkx2-1
Matk
Vps18
Vps11
St8sia3
Eif4g2
Vcp
Dnajc27
Kdm2b
Anxa7
Cd59b
Ufm1
Kctd7
Dlx2
Irf3
Ahdc1
Sema4d
Oard1
Stam2
Mob3b
Ccdc117
Atxn1
Fasn
Alyref2
Rps12-ps17
Arhgdig

As3mt
Top1mt
Brk1
Dusp1
Smdt1
Scamp2
Trim41
Ogfod1
Mgst3
Bloc1s2
Grm8
Fam166b
Tmed4
Pgs1
Snord49b
Edem3
Ergic2
Ccser2
Uchl3
Rps8-ps2
Zfp108
Stmn3
Nfkbie
Ormdl3
Mir7578
Zfp956
Unk
Eif3j1
Sh2d5
Pex12
Scarb2
Ubiad1
St6galnac4
Mtmr9
Snx20
Lonrf2
Maf1
Filip1
Cd81
Ctsa
Dync1li2
Zfp780b
Mfsd14a
Mir3076
Zfp612
Mettl16
Lrrc8dos

Crem
Nudt5
C130074G19Rik
Crhbp
Pcbp4
Ppp1r14c
Chst15
Xrcc6
Tox2
Rps19-ps7
Slc25a38
Myt1l
Gdi1
Cd8b1
Nap1l2
Tm9sf4
Tsr2
Atp2a2
Crcp
Ak6
Dctn2
Ifitm7
Zcchc17
Mknk2
Rps14
Ppp2r3d
Pomp
Pakap
Sipa1
Pop7
Mfap1a
Elp3
Cnrip1
Cacybp
Nmbr
Sptbn4
Tpp1
Uxt
Rpp25l
Phactr1
Calcoco1
Atg2a
Pyroxd1
Dpysl5
Ppp1r16a
Adra1a
Atg4b

Hrh1
Ankrd63
Gprasp1
Patz1
Ilvbl
C87436
Ppdpf
Spock3
Layn
Vps33a
P2ry1
Coq2
Ppip5k1
Ccl27b
Naa60
Amer2
Aplp2
Tnip2
Coq8a
Abt1
Slc10a4
Ythdf1
Fbxo42
Relch
Zfp711
Tcf25
Nacad
Myadml2
Eml1
Camk2d
Rpl31-ps15
Tmem258
Aff2
Rps12-ps10
Tmppe
Tarbp2
Nectin2
Ube2a
Eid1
Cebpz
Nr2e1
Farsb
Mad2l2
Dus1l
Dnajc18
Atf5
Rpl13-ps1

Rwdd2b
Pdk2
Ddx56
Tln1
D10Wsu102e
Eef1e1
Fam20c
Ccdc85a
AU041133
Syn2
Wbp11
Zfp787
Miga2
Car8
Gfer
Clcc1
Cdc5l
Msantd1
Rab26os
Zbtb7a
Dtymk
Mir1902
Erich1
Ecsit
Yars
Usp21
Oprd1
Ppm1d
Syt5
Pfkp
Oxct2b
Camsap2
Unc5d
Rcan1
Eci1
Amotl1
Gpaa1
Heg1
Mcoln1
Eif3l
Calm2
Elfn1
Scg3
Cenpc1
Tprkb
Polrmt
Lin37

Rab30
Phlpp2
Yif1b
Gngt2
Med7
Col23a1
Prox1os
Tmem131l
Gamt
Tmsb15b2
Ascl1
Mepce
Cdk5rap3
Rpl29-ps2
Atp6v1f
Bace1
Ypel2
Slc25a5
Agbl5
Actn1
Rnf10
Ift27
Naf1
Rnf5
Tspyl5
Ccdc84
Prmt3
Zcchc9
Amot
Cdk4
Golga3
Abat
Dlgap2
Rims1
Tgfb1
Tmem128
Strn3
Tmem165
Slc25a10
Palmd
Sgce
Slc2a12
Rab28
Zfp965
G6pdx
Tspan2os
Ap4m1

Asphd1
Eif4e3
Mia
Hmgcll1
Chga
Rbm11
Atg16l2
Tmem173
Rpl10-ps1
Abce1
Lig4
Spsb3
Elob
Chd4
Trappc1
Ggnbp2
Tub
Psmb7
Slc35b2
Hsdl2
Hsp90ab1
Polr2j
Galt
Acer3
Lipa
Tmem44
Rpl31-ps8
Rps11-ps4
Med18
Ndc1
Slc4a1ap
Atp5l-ps2
Hinfp
Ttc5
Dph3
Kidins220
Ephb6
Mob2
Cyb5d1
Nyap2
Rpl21-ps15
Mydgf
Surf2
Mir6357
Cenpo
Micu2
Naa35

Rab6b
Vps4a
Gp5
Nedd4
Rps5
Fkbp11
Mrpl19
Phf20
Cbr3
Rdx
Zfp87
Syngr1
Ntan1
Zfyve27
Rab27b
Cxxc1
Clns1a
Blmh
Atcayos
Mettl8
Kansl2-ps
Tbc1d19
Zbtb46
Sp9
Zfp39
Yipf2
Sall3
Cers1
Pi4kb
Utp3
Mamdc4
Mark1
Qsox1
Cdan1
Mindy1
Gtf2h2
Dhx30
Ddx39b
Alk
Cacul1
Trbc2
Efnb2
Snrpf
Zfand2a
Cnih4
Rpn2
P2rx4

Rbck1
Ddx27
Sf3b5
Frmd3
Fcf1
Kcnip1
Zfand5
Lats2
Rpl21-ps7
Tent4a
Dapk3
Hs6st1
Pygo2
Samd14
Mir129-1
Crebzf
Ccnk
Rnf113a2
Ttyh3
Uqcr10
Cpt2
Msi1
Mrpl36
Manf
Slain2
Srd5a3
Lemd2
Cmc1
Vps35
Rpl7a
Kcnk13
Bloc1s2-ps
Nupl1
Fam122b
Khdc4
Plek
Tmem178b
Nedd9
Otud7a
Ankrd13d
Cfl2
Podxl2
Zfp85os
Phf13
Tbc1d2b
Hrh3
Foxj1

Lrrc3
Sgsm1
Ntf3
Pip5k1c
Smug1
Derl3
Timm23
Uhmk1
Eif2d
Nop14
Aifm3
Clip4
Pus1
H3f3a-ps1
Dars
Hectd3
Ubtd1
Ppox
Trim17
Mir6340
Coq4
Immp1l
Zfp428
Sfpq
Mid1ip1
Med11
Bckdhb
Serf1
Mycbp
Larp4b
Myh9
Rps2-ps13
Setmar
Gde1
Gmnn
Aacs
Pkmyt1
Mcfd2
Cd72
Pcdhb10
Ppp6c
Frmd4b
Errfi1
Rbm18
Rpl18a
Nr2f1
Aamdc

Mrpl16
Stambpl1
Nol9
Proser1
Cmtm8
D130017N08Rik
Mir207
Acyp1
Ints4
Rnf157
Capn10
Ankrd34c
Mrpl27
Cgref1
Rpp30
Ctr9
Rps6ka1
Stoml2
Cpne8
Mrpl3
Apba1
Mars
B230118H07Rik
Grb2
Dph2
Zc3h18
Epb41l4aos
Chmp3
Nxph2
Smcr8
Tsn
Fnta
Ahsa1
Ubr5
Tmem222
Atp6v1h
Rab39b
Agtpbp1
Gtf3a
Camk1g
Srsf12
Prdm12
Mast1
Gnrh1
Usp22
Amph
Gstm6

Adam9
Cry2
Aldh3a2
Txnl4b
Atxn2
Hspa9
Nfu1
Smarce1-ps1
H1fx
Adgrg1
Smim4
Gpr45
Asb16
Ccdc107
Tm2d2
Gins4
Ipo4
Ctu2
Twnk
Abcf3
Scn9a
Rrp9
Zfp597
Atp6v1g1
Bid
Ppp6r1
Sod1
Zfp955b
Mrps36-ps1
Ppp1ca
Rtl8c
Tmem9b
Zbtb11
Gpr34
Phc2
Ttc28
Nubpl
Tmem242
Zfr2
Eefsec
Ubxn11
Fam184a
Junb
Mt3
H2-K1
Utp23
Tenm3

Furin
Kti12
Nop10
Car10
Cacnb2
Unkl
Lgals8
Plxnc1
Cdh22
C030014I23Rik
Creg1
Mrps16
Bag3
Mir101a
Skp1a
Pitpna
Prrg3
Dcdc2b
Ell
Echdc1
Zfr
Adipor2
Chrna3
Jakmip2
Myl6b
Fbl
Zfp442
Spata5
Gldn
Fto
Mtfp1
Alyref
Ccdc130
Kctd10
Plaa
Znhit3
Mrpl43
Hp1bp3
Kcnb2
Abhd10
Nfil3
Ogfr
Eif2ak4
Taz
Cox6b1
Acat2
Zfp12

Mtss1
Kdm4a
Egln3
Prrc2c
Zfp775
Trim32
Igf2r
Wdr74
Fam53c
Mapk1ip1
Cdkn2aip
Tusc2
Cd47
Eef1akmt1
Gzf1
Bola2
Tac1
Leprot
Ckap4
Uba52-ps
Pogk
Itpa-ps1
Foxred2
Zscan26
Ncbp2
Fbxo38
Ism1
Nckap5l
Phyhd1
Tmem130
Ccl19-ps3
Cygb
Grin2d
Rex1bd
Golph3l
Nuak1
Rad17
Rhbdl1
Smad1
Gabrg1
Ppm1g
Rap1gds1
Zfp324
Snhg9
Arid2
Msh2
Get4

Adnp
Faxc
Pycard
Psmb2
Dgka
Sp2
Maco1
Bod1
Fabp3
Arhgef18
Tmem59
Mro
Xkr8
Pfn4
Dhx38
Fam102a
Bcdin3d
Rtn2
Tmem132d
Dzip1l
Cbx4
Rbmx2-ps
Trbj2-7
Klhl9
Fam213b
Zfp24
Cryab
Myl6
Ptges2
Syt11
Hint1
Cltc
Cldn34c2
Acot13
Tpd52l2
Rps8-ps4
Senp8
Irf2bp2
Phkg2
Sec22c
Nudt7
Srm
Nsfl1c
Ube2n
Dact3
Bud23
Ubxn2a

Acp2
Spats2
Lyrm4
Kcnj11
Sart3
Hdac2
Clgn
Slc9a6
Tmub2
Pfdn1
Hnrnph3
Farp1
Pitpnc1
Gpatch1
Cstf3
Adk
Tsen2
Rsad1
Pnp2
Fv1
Zup1
Dhx57
Penk
Syndig1l
Gpc3
Ncln
Adap2
Aldh5a1
Tcap
Eno4
D230025D16Rik
Bricd5
Acadm
Mrip-ps
Ankrd61
Fn3krp
Zfp639
Mdk
Esrra
Plekhm1
Manea
Cdk2ap2
Bccip
Nelfcd
Rab3gap2
Supt4a
Srpk2

Gatb
Kctd9
Zfp566
Mir3572
Lancl2
Tomm20l
Dnlz
Tor2a
Rnf41
Tyrobp
Wdr45
Trappc5
Arhgef15
Dnpep
Hmgb1-ps5
Amfr
Hypk
Eps15l1
Phrf1
Eipr1
Iffo1
Odr4
Prpsap2
Stau1
Rpl18-ps1
Bop1
Akt1
Mir670hg
Rps28
Mterf3
Ccdc85c
Samd1
Eme2
Rdm1
Tcf4
Cpsf3
Nrg3
Arf1
Nt5c2
Aup1
Mettl5
Atp6v1d
Pcbp2
Dnajb12
Chchd10
Sos2
Atpaf2

Acadvl
Tlr13
Ric8b
Ewsr1
Arl1
Uqcrc2
Jrk
Zfas1
Gipc1
Smurf1
Ntmt1
Dscr3
Insig2
Cnppd1
Ydjc
Exosc3
Llph-ps2
Amn1
Cirbp
Naxe
Rad23a
Rcl1
Mtcl1
Parp6
Ssbp2
Fhl1
Cep170
Tiam1
Hars2
Amd-ps3
Mfsd13a
Sacm1l
Rps6kc1
Oaz2-ps
Pcnx4
Lysmd1
Marcks
Rps2-ps10
Rnasel
Zfp2
Cactin
Pmpcb
Zfp146
Cyp20a1
Cdh4
Rasgrf2
Ip6k2

Atp1b3
Zfp9
Kctd1
Mapk8ip3
Dact1
Psme1
Zfp879
Sash3
Ubac2
Crh
Dnajc30
Hnrnpm
Gpn1
Ndufa7
Opa3
Ubxn8
Xab2
Cog7
Axin2
Mrps24
Triobp
Wrnip1
Actn2
Cox19
Tmem176a
Gpr19
Hist1h2ad
Dnajc14
Safb
Emc6
Timm10b
Pex10
Mir3103
Nol12
Taf1d
Tmem109
Aurkaip1
Rab5c
Abcd2
Rnf139
Ccdc157
Snapc5
Ten1
Rpl39-ps
Tmem216
Parg
Rxrb

Pold2
Mfsd11
Zfp874b
C1d
Pvt1
Tbrg1
Rabep1
Dcaf12l1
Zfp687
Mthfsl
Atp5s
Elp5
Zfp984
Tuba4a
Usp32
Ercc2
Baiap2l2
Rpl10-ps6
Emc2
Klhl12
Nfe2l1
Mid2
Rpusd3
Prkar1b
Kpna6
Riiad1
Fbxo16
Mrpl58
Mta3
Sall1
Daam1
Npc2
Pknox2
Dtnb
Zfp207
Usp39
Wdr77
Gng8
Fbxo11
Idh3a
Dnajb2
Rnf227
Pdzrn4
Psip1
Rgs3
Laptm4b
Fam57a

Map2k2
Proscos
Rpl19-ps4
Scaf4
Ppp1r35
Coq10a
Dpm3
Cct4
Spata33
Cwf19l1
Atxn7l3b
Fance
Smg6
Timmdc1
Tmpo
Ndufv1
Rpl15-ps2
C030017D09Rik
Cs
Chrnb2
Orai1
Pdzd7
E130309D02Rik
Cenpx
Snx17
C1qc
Acot7
Cited1
Zfp93
Mtch1
Zfp395
Adcy6
Fam217b
Rac1
Sun2
Insig1
Tmie
Rbmx2
Zc4h2
Rpp25
Limk2
Kat5
Atxn7l1
Elovl5
Aatk
Zkscan5
Mir8099-2

Pstpip2
Tspan33
S100a10
Lrrc51
Tmem120a
Tmem167b
Arl2
Rasgrp3
Brd3
Pex5
Shb
Trmt112
Hist1h4b
Lrfn2
Aff4
Prr3
Bivm
Csf2ra
Tpgs2
Dhx16
Hibadh
Osbpl10
Casc4
Psmb4
Ntn4
Ddx54
Rwdd3
Trappc13
Mfsd5
Met
Ubxn1
Nsd3
Cenpm
Mttp
Bloc1s4
Tti1
Magoh
Spag7
Cops2
Dzip3
Trpm2
Hyls1
Mgrn1
Psmd1
Fbxo33
Dlg4
Mrpl55

Hist2h3c1
Fam155a
Vps37d
Mir1191
Spats2l
Thg1l
Thrap3
Taf3
Gpr180
Fam43a
Smim10l1
Zwint
Hvcn1
Glmn
Galnt10
Ift52
Ctif
Ccdc25
C1galt1c1
Dnajc2
Hist1h2ba
Hdac1
Dynlrb1
Erich3
Ercc8
Ercc1
Pde4b
Sap18
Cul3
Dhx15
Thoc5
B3gat1
Tmem41a
Ate1
Ndnf
Tmem74b
Inpp5e
F11r
Lsamp
Rpl31-ps13
Glg1
Tgfbr1
Cr1l
Snx6
Gdap1
Crebl2
Rcn1

Ddrgk1
Hikeshi
Arhgap4
Apoh
Pik3r3
Emg1
Cadm3
Hap1
Hif1a
Rps21
Unc5b
Arl16
Metap1d
Plpp2
Dexi
Rps12-ps5
Gjd2
Ndufb2
Txn1
Crlf2
Maml1
Rpl7a-ps11
Arhgap22
Rnu7
Uqcc2
Nans
Becn1
Map7d2
Grip1
Etl4
Zeb2os
Sox6
Yme1l1
Eno1b
Rfc3
Sptssa
Tmem267
Cbx3-ps4
Stmn1
Farsa
Irs1
Tlr7
Mtmr7
Mbtps2
Esd-ps
Zfp664
Ctdp1

Dusp3
Zfp963
Luc7l
Mir7658
Zfyve1
Dlx1as
Dennd5a
Gtf2e1
Krba1
Cox6a2
Fgf13
Ubap1
Ccdc149
Ngb
Ublcp1
Fam162a
Kcnq4
Msl1
Eny2
Fam181b
Nr2f6
Pnmal1
Uchl1
Fgfr1op
Gas8
Cib1
Gpr85
Sdha
Casp3
Ppp1r14a
Ppp3cc
Usp50
Rrn3
Ube2h
Keap1
Lhx6
Fahd2a
Cycs
Pde9a
Ddit4
Hspe1
Use1
Cdk8
Gucy1b1
Dut
Asb13
Capn7

Pibf1
Hmgb1-ps6
Slc7a1
Atg4d
Dpysl3
Sparc
Mogs
Zswim7
Atg14
Mrpl34
Trmu
Dusp28
Nudt17
Nxpe4
Evl
Cdc42bpa
Pdxk
Samd12
Fam149b
Rps19-ps1
Ankrd39
Zfp954
Ppm1m
Dnm1l
Ncaph2
Tyro3
Zfp940
Igf1
Pdcd11
Ccdc50
Trpc5
Med9os
Myh10
Cysrt1
Clk3
Gabrg3
Psd2
Mapkap1
Rnf216
Cdh18
Acly
Rpl30-ps1
Zfp747
Bnip3l-ps
Rufy2
Srd5a1
Meig1

Gtf2h5
Lpgat1
Pcnp
Mllt11
Gpr107
Gal3st4
Tomt
Taf4b
Selenot
Tmem185b
Snx8
Osbpl8
Gnb2
Rras
Slc7a8
Cfap97
Fam131b
Tdrp
Mir670
Prkra
Smim1
Pip4p1
Mrpl10
Oprl1
Cisd3
Map1s
Ciart
Rrnad1
Map3k10
Alad
Mxi1
Rpl35a-ps5
Taf15
Zfp68
Tmem80
Wdr82
Gpank1
Naa50
Sema5b
Dand5
Sdk2
Etf1
Armc5
Zfp973
Sdhc
Rpl17-ps4
Foxj3

Hmga1
Epha10
Mir3098
Ppp2r1b
Trappc11
Cldnd1
Dhrs1
Zdhhc2
Enkur
Pofut2
Hist1h4c
Ttll1
Hras
Ctxn2
Rps27rt
Cxxc5
Nup43
Cmtm6
Plpbp
Clta
Hspb11
Adck1
Mrps36-ps2
Nit2
Eif1ax
Kif17
Cuedc2
Cnot3
Map3k1
Hsbp1
Fcho1
Lst1
Kansl2
Rabepk
Cab39
C330021F23Rik
Pbx3
Man2b2
D430036J16Rik
Ccl17
Tmem243
Cic
Myh7
Dpy30
Ssr2
Hist3h2bb-ps
Bcorl1

Mark3
Vdac3
Ppm1b
Brsk2
Ddx3y
Ube2j2
Tex261
Faap20
Ccpg1os
Cramp1l
Pop4
Rps12-ps14
Polr2g
Jdp2
Slc16a11
Tex10
Trpc3
Zfp287
Irf2bpl
Ankrd13b
Ddost
Zfp938
Atad1
Mtx2
Chd5
Snhg15
Grm5
Wac
Kbtbd2
Sik1
Med15
Adcyap1r1
Hnrnpc
Rpl21
Tatdn2
Tubb5
Fam114a2
Ralb
Gpr139
Cd14
Pdxdc1
Rpl38-ps1
Nsmce2
Rpl15
Slc35d3
Slc25a12
Lamp5

Astn2
Denr
Zfp408
Wtap
Gstz1
Spopl
Suv39h1
Rarres1
Rpl23a-ps1
Frmd5
Ranbp6
Snrnp35
Smap2
Cuta
Slc17a8
Wdr5b
Tle3
Hmgxb4
Pigu
Ppp1r18
Sgf29
Dcun1d1
Fxr2
Efhd2
Lamtor2
Nrip2
Tmem87b
Wwp2
Mkl1
Rbm6
Gigyf1
Hexim2
Itpkc
Rragc
Alkbh4
Gga1
Gdap1l1
Rpl28
Ppil1
Ccdc174
Clvs1
Rap1b
Zfp37
Elmsan1
Setd5
Parm1
Jpt2

Nsg2
Ubash3b
Hist1h2bf
Ube2z
Psmd8
D830025C05Rik
Cnep1r1
Gtf3c6
Rara
Tbrg4
Serf2
Gtf2h4
Fra10ac1
Tagap1
Lrrc23
Nipal2
Cptp
Mob4
Synm
Lmbrd1
Abcf1
Txndc17
Mpdu1
Srp9
Mir1957b
Soga1
Stard3nl
Apold1
Ola1
Oaz2
Atg12
Pacrg
Zmym4
Specc1l
Yju2
Zfp30
Eml4
Stx12
7-Sep
Id3
Hdac4
Trnp1
Ttc9
Rnf111
Exoc2
Trex1
BC055324

Med24
Chpf
Tmem198
Ndufa10
Got2
Rab11fip5
Hipk1
Lyrm2
Ndufab1
Cstad
Pfdn6
Htr2a
B4galt2
Prrt4
Pik3ip1
Mir5122
Timm10
Sirt6
Ankrd46
Hcn4
Cln6
Txnl4a
Polr3a
Foxo3
Plod3
Bbs5
Rundc1
Isy1
Il17d
Dhx40
Lmln
Pold4
Ankrd24
Tnfsf12
Hapln1
Efcab2
Tmem237
Cox5b
Uap1l1
Rpl3
Pvalb
Rbm27
B3gnt9
Nme6
Supt20
Chac2
Ubp1

Tvp23a
N4bp3
Zxda
Tmem29
Tcte1
Dtd2
Fis1
Fbxo18
Lor
Arf6
Mau2
Fxyd1
Drg2
Zfp946
Poglut1
Ptprf
Mif
Rpl27-ps2
Usmg5
Ifit2
Ebag9
8-Mar
Cebpa
Stk39
Vsig10l
Gramd1b
Timm17b
Ccdc137
Fem1b
Arhgap36
Tm6sf1
Kctd13
Uba52
Stambp
Fastkd3
Ccdc86
Ndufa3
Yy2
Bcs1l
Ccdc92
Grn
Ap1s2
Cstf1
Inpp1
Commd9
Tlk2
Anp32b-ps1

Mcf2
Safb2
Stk32b
Iqcg
Atp6v1b2
Nxf1
BC048403
Ost4
Chtf18
Asah2
Kantr
Pygm
D17H6S53E
Zranb2
P3h4
Phb2
Kcnj9
Mgst1
Aes
Mcrs1
Tcaim
Psma5
Ipo9
Nob1
Zfp354a
Iars2
Bsdc1
Cyth3
Lipo3
Ispd
Pcdhb2
Pcyt1a
Gpx3
Slc50a1
Mrps15
Letm1
Ern1
Echs1
Sf3b3
Slc27a4
Pusl1
Ube2d3
Ccdc175
Fgf10
Tox4
Nr1d2
Gnptg

Nelfe
Rps23-ps2
Pmf1
Mcts1
Rnf215
Hk1
Apba3
Cd68
Nap1l3
Vwa5b2
Klhl7
Lrrn3
Mir7676-1
Ppp1r3f
Hsf1
Srrt
D3Ertd254e
Usp14
Rpl35
Cdh13
Uspl1
Tbc1d10a
Slc35e1
Lactb
Dcaf5
Fam219b
Enah
Fbxl17
Carf
Mrpl46
Wnt5a
Sdc2
Slbp
Lpcat2
Aars
Ndufs6b
Dnajc16
Mrpl45
Spire1
Ccdc184
Pik3cd
Arrdc1
Cdyl2
Mib2
Bag5
Barx2
BC002059

		Ap3s1
		Ccdc61
		Rrp8
		Nrf1
		Slc25a40
		Zbtb24
		Cbr1
		Rps6ka5
		Zcchc14
		Hdgfl2
		Dcp2
		Dhx36
		Cotl1
		Ctsb
		Srsf10
		Ptges3-ps
		Cd37
		Mrps5
		Leprotl1
		Sec23ip
		Gosr1
		Rfxank
		Psmg3
		Zfp536
changed in CCK	510	Syt2
		Slc25a15
		Cdk6
		Plp1
		Gm26377
		Ptprz1
		Cdr1
		B3galnt2
		Serpina3n
		Gm15793
		Gm49405
		Gm12715
		Gm1821
		Ovol2
		Rad54l2
		Gm15464
		Lrrc10b
		Gm6180
		Urb1
		Cacna1h
		Chst8
		R3hdm4
		5830462I19Rik

Fyb2
Fgf5
A330023F24Rik
Gm12918
Erbin
Shisa6
Gm12394
Crim1
Gm26702
Hic2
Stk35
Gm5510
Gm6563
Camk2a
Dennd6a
Ccl21a
Capn1
Rnf169
Fth1
Prex1
Gm15564
Gm21958
Dcbld1
Crocc
Prelp
Teddm2
Gm12416
Ston2
Zcchc7
Fam69a
Gm10355
Resf1
Gm26811
A330074K22Rik
Lrp1
mt-Rnr1
Baiap2
Galnt9
Adgra2
Map9
Adcy9
Psd
Sgms2
Gm25911
Sstr4
Gm13694
Gm14138

Ror1
Gm21093
Gm10222
Zfp384
Gm20900
Ylpm1
Med17
Cyp4v3
Gm26384
Cpne9
Gm7353
Muc3a
Gm21988
Ctnna1
Zfp26
Zc3hav1l
Plppr2
Nfic
Gm13623
Gm5859
Gm12350
Gm8624
Serpina3f
Daam2
Zfp106
Nvl
Dgkz
Gm26767
Plxna4
Gm26596
Gm12892
Gm24270
Zgpat
Slc7a4
Prkd1
Gm6395
Efr3a
Gm11759
Ido1
Slitrk5
Tmc7
1600029O15Rik
Gm20498
Nwd1
Klhl35
Ddn
Mast3

Gm7336
Pagr1a
Ywhah
Acp5
Gm2163
A330070K13Rik
Adgrb2
Gm20671
Zfp932
Gss
Gm11605
Ltb
Gm13292
Gm11263
Gm17786
1500002C15Rik
Cpne7
mt-Nd1
5730522E02Rik
Gm6682
Tbl2
Cc2d2a
Gm14329
Tbcel
Gm867
Nfix
Gm7984
Rpl5
Prlr
Ubr4
Gm13303
Gm21985
Vit
Gm16581
Acap3
Cebpb
Serpina3e-ps
Ugt8a
Auts2
2310002F09Rik
Tnfrsf13c
Gm26995
Nsmf
Fat3
Megf11
Mobp
Usp54

Setd4
Zfp992
Rcor3
Gm6472
Dstyk
Mapk14
Gm10132
Slc22a15
Lime1
Gm6498
Gm10359
Gm10069
Gm14044
Cspg4
Adam11
F5
Zfp953
Ccdc74a
Gm13767
Cul2
Epb41
Mzf1
Hrnr
Gm21987
Gm20417
Gm960
Dph6
Sco1
Wdr35
Gm24187
Pdpr
Gm12070
Gm12841
Klf10
Gm3892
Csmd2
Tjp2
Gm26602
Erf
Rspo1
Hpcal4
Gm10335
2810455O05Rik
Snrnp48
Rad51ap2
Col4a3bp
Nptxr

Gm4995
Esyt3
Ap1s3
Spidr
Gm2606
Ddx46
9-Sep
Gm13696
Gm13691
Gm10250
Ttn
Gm13301
Gm13215
Cep41
Gm23935
Ctxn3
Gm20659
Gm26794
Gm6222
mt-Cytb
Natd1
Gm10600
Stard7
Gm5577
Cdc42ep4
Smc5
4930419G24Rik
Fam234b
Vcam1
Wscd2
mt-Tl1
A230065N10Rik
Pnpla1
Gm16229
Gm17167
Ccdc42
Ppp1r16b
Gm26805
Agtr1a
D630045J12Rik
Gm9396
Gm26917
Sfswap
2510002D24Rik
Pcdh1
3-Sep
Gm15879

Ctdspl
Gm8172
Ppig
Setbp1
Gm10593
Fgd6
Plxna1
Gm16409
Znrf3
Sipa1l3
Gprin3
Syt17
2810002D19Rik
Gm11149
Gm4924
Efna2
Pygb
Gm26770
C030047K22Rik
Aldh1l2
Tmem121b
Tuft1
Gm20499
mt-Rnr2
Parp4
Prrt2
Scara3
Gm13698
Pi4k2b
Cybrd1
Gm7206
Gm16278
mt-Nd4l
Rtn4rl1
Nox1
mt-Atp6
Gm42418
Chrna7
Umad1
Gm9905
Angel1
Mlh3
Prkcg
Gm13750
Arntl
Gm8213
Gm11966

Rad21l
4731419I09Rik
Olfr31
Ccl21b
Cavin2
Cerkl
Gm21981
Stard4
Gm13296
Gm13304
Arid4b
Ccl28
Gm3362
Gm20441
Camk2b
Islr2
Med13l
Gm21541
Npcd
Krt20
Gm20518
Gm13340
Gm6061
Gm26520
Gk
Tpmt
Rttn
Rsrp1
Rpl9-ps4
Slit3
Hltf
Ung
Syt7
Me1
Plec
Zbtb1
Gm3883
Gm14494
Gm15772
Insm1
Gm17036
Zc3h7b
Gm7536
Rfc1
Cntln
Ankrd28
Igfbp7

Igsf9b
Slc44a5
Btaf1
Gstcd
Gm13610
Arf3
Nup98
Neat1
Slc17a7
Slit1
Hadha
Dyrk2
Il17rd
Gpr171
Gm8430
Gm21984
Hebp1
Gm2962
Plekhg5
Ppp1r1b
Ptk7
Zfp532
Gm13695
3110070M22Rik
Gm38393
Unc5a
1190007I07Rik
Ptbp1
Tspan18
mt-Nd4
Bmpr1a
Zfp873
Nsun7
Gm16740
Scd2
Atp1a1
Topbp1
Ganc
Gm14760
Itga6
Hmgcs1
Cmya5
Atp2b3
Zfp592
Gm17202
Lrrn2
Gm6851

Gm26560
2310058D17Rik
Hrk
Gm12671
Gm49322
Cfap58
Glipr1
Gm13768
Cwc22
Kdm4b
Ncan
Ccdc96
Gid4
Fut9
Tet3
Snap23
Gm20605
Gm20503
Tmcc2
Bche
Gm14703
mt-Co1
Dagla
Gm21986
Ldlrad3
Ptpn21
Slco2a1
Dhx33
Gm5453
Gm17494
Yipf6
Gm11425
Btbd17
Gm10123
Rpl9-ps6
A530032D15Rik
4632411P08Rik
Kitl
Dtnbp1
Jarid2
A330102I10Rik
Mccc2
Amy1
Hmox1
Arhgap39
Gm13697
Dync1h1

Trmt11
Rilpl1
Gm13693
Gm15710
Slc19a1
Gm26733
Slc16a2
Smo
Klhl23
Gm26821
Rpl4
Gm12537
Rftn2
Xlr3b
Dsc3
Csf1
Fam189a1
Gm17168
9130019P16Rik
Atf7
Tstd2
Galnt17
Pabpc5
Gm11196
Ppp1r13l
Eif3e
Aim2
Gm11185
mt-Nd5
Tagln
Gm10704
Cobll1
Gm10284
Gm20683
Gm16061
Zw10
Rgs11
Cpne6
A230005M16Rik
Urah
Gm15920
Mdga1
Sox12
Gm10275
Dpep1
Kcnh3
Rtn4r

Gm15832
Pcdh19
Zfhx2
Gm14278
Gm14164
9230009I02Rik
Gpc1
Lxn
Gm13302
Gm6863
Twsg1
Cbl
1110002L01Rik
Abhd5
Dnmt3a
Fyco1
Nlrx1
