# Supplementary material for: Identification of Neurensin-2 as a novel modulator of emotional behavior
Source: Mol Psychiatry. 2021 Mar 19;26(7):2872–85. doi: 10.1038/s41380-021-01058-5 (PMC8505262; doi:10.1038/s41380-021-01058-5)
Supplement: Supplementary file 5 — Table S3 [file 41380_2021_1058_MOESM5_ESM.pdf]

Table S3

Genes related to the post synaptic membrane

Gene ID	pval	RPKM in CCK	cluster
GRIA1	0.048	80	AMPA
CNIH2	0.021	75	
NEURL1A	0.001	13	
MINK1	0.037	7	
CACNG8	0.029	5	
SHANK1	0.003	80	PSD
SHANK3	0.002	7	
NLGN3	0.019	23	
DLGAP3	0.023	2	
GRIN1	0.001	15	
GABRA3	0.005	5	GABA
Grm1	0.006	5.5	Glutamate Metabotropic
Grik5	0.001	10	Kainate
faim2	0.019	54	Non specific
LRRTM1	0.013	35	
Shc4	0.034	3	
BCR	0.024	15	
SEMA4F	0.001	13	
KCNB1	0.007	7.5	
