# Supplementary material for: Identification of Neurensin-2 as a novel modulator of emotional behavior
Source: Mol Psychiatry. 2021 Mar 19;26(7):2872–85. doi: 10.1038/s41380-021-01058-5 (PMC8505262; doi:10.1038/s41380-021-01058-5)
Supplement: Supplementary file 6 — Table S4 [file 41380_2021_1058_MOESM6_ESM.pdf]

Table S4

Protein-transport related genes

Gene ID
nxt1
bbs4
naca
vps52
eif5a
timm13
arfgef2
pex7
copb2
ap1s1
pom121
tomm7
tomm6
seh1l
atg7
timm9
sec22a
rabgef1
vps39
ap5z1
kdelr2
gabarapl2
ramp2
nup88
rrbp1
dennd1a
vti1b
ap4s1
gabarap
micall1
preb
phax
scfd1
sec61b
aktip
ap2a1
cog8
tom1l2
ist1
rab5a
rangrf

labeling of the protein transport pathway
Endosome
Golgi/TGN
ER/ER-Golgi
autophagy
peroxisome
nucleus-cytoplasm
mitochondria- cytoplasm
secretory
other

slc15a3
vps25
mon1a
