# Supplementary material for: Identification of Neurensin-2 as a novel modulator of emotional behavior
Source: Mol Psychiatry. 2021 Mar 19;26(7):2872–85. doi: 10.1038/s41380-021-01058-5 (PMC8505262; doi:10.1038/s41380-021-01058-5)
Supplement: Supplementary file 7 — Table S5 [file 41380_2021_1058_MOESM7_ESM.pdf]

## Table S5

**CRISPR validation:  Results of OFF-Target verification by Sanger sequencing**

| Gene ID | location | chromosomal location | sequance | PAM | Result-Editing | F primer | R primer | length |
|---|---|---|---|---|---|---|---|---|
| **Nrsn2** | **Exon 2** | **chr2:152,369,584-152,374,334** | **TGGAGGAAAGTACATGGTAT GGG** | **GGG** | **Yes** | TGCCAGATGGTCCTCAGAGA | CTCTGCCAGAAGGCATCCAA | 591 |
| Zdhhc5 | Exon 2 | chr2:84,710,597-84,710,619 | TGAAGGAAAGCACATGGGAG | GGG | No | GGGCATGTTGGAGCTCTGAT | GCTGCTCCTAGTGTGGTGTT | 493 |
| Abca8a | Exon 39 | chr11:110,025,789-110,025,811 | TGAAGGAAAATACATGGAAT | TGG | No | GCTGATGAAAATTTAGAGAAAAAGCAATGATAAT | AAAAGCCACTGAGCTAGCTCTCTCTACAATT | 689 |
| Rhbg | Exon 5 | chr3:88,245,808-88,245,830 | TGGACGACTGGACATGGTAT | GGG | No | CGTTCTGGACGTGGACCTTA | GGCTGCTTAGTAGTGACGGA | 517 |
| Fat4 | Exon 7 | chr3:38,970,701-38,970,723 | TGAAGTTAAGTACATGTTAT | TGG | No | AGGCAGTTGAGCTCTTAGGTC | CTTAGAAGAAGCAGACACGCT | 541 |
| Wdfy3 | Exon 35 | chr5:101,889,296-101,889,318 | TGGAGGAAGCTACCAGGTAT | TGG | No | CAAAGTTTGGGTACACCTGAGAAT | TCACAGGCTGGCTTTAAGTGTA | 539 |
| Ccdc80 | Exon 3 | chr3:45,095,317-45,095,339 | TGCAGGAAAGAACAGGGTAT | GGG | No | GCAGTAGGTCAGCTCGGTATC | ACAGCACCATGCTAAACTTGC | 579 |
| Theg1 | Exon 6 | chr5:77,056,055-77,056,077 | TGGAGGAAGGTAGCTGGGAT | TGG | No | CATGCGTGTGGGAAAAGAGC | TGAACTCGCTCAGAGAACGG | 534 |
| Ntm | intron 1 | chr9:29,941,757-29,941,779 | GGGAGGAAAGAATGTGGTAT | TGG | No | ACAGCAGATTCGGTGCATGA | GGGTGGCGGTTAGGATAGTG | 526 |
| Zpf644 | intron 1 | chr5:106,667,595-106,667,617 | TGGTGTAAAGTACATGGAAG | AGG | No | TATGAGCCATCAGTTCTGCACC | TAGCTGCTATCACAATTCTCACTTT | 437 |
| Spidr | intron 1 | chr3:88,245,808-88,245,830 | ATGATAAAAGTACATGGTAT | TGG | No | CCAGGATAGCCAAACAATCC | AACTCAAGTGTCCCCAGGTG | 194 |
| Gm5139-Gm18294 | intergenic | chr3:64190048-64190070 | TTTAAAAAAGTACATGGTAT | TGG | No | GGAATCGTGAATGGTGTATTGGTAAGC | ATAGGTAGGGAGATAGTGGGTAGCCTTGTC | 680 |
| Gm12010-Gm12011 | intergenic | chr11:16210581-16210603 | TGTTAAAAAGTACATGGTAT | TGG | No | AGTTCCTTGATCCACTTAGACTTGAGATTTGTAC | AGAAAGAAATTTAAGAAGATCACAGAAGACAGAAA | 533 |
| Kcnq5-Gm26580 | intergenic | chr1:21959279-21959301 | TTGGGTAAAGTACATGGTAT | GGG | No | CTCCCTGCCTACAGACCTCAA | TTAAGCTCATCTCCTTGGGTTGAT | 560 |
| Olfr1353-Olfr1352 | intergenic | chr10:78977749-78977771 | TGATAAAAAGTACATGGTAT | TGG | No | ACCAAGCAATATATGAAATATCTGTATGGCAA | TGTTCTGTTCTGGAAATTTTCCCCTG | 668 |
| Slc10a2-n-R5s94 | intergenic | chr8:5212502-5212524 | CCATGGAAAGTACATGGTAT | GGA | No | TACTCAAGTAAAAGGGGTAAAAGGA | AGGCCAATGACAGGTGTGTA | 431 |
| Gm7803-Gm14611 | intergenic | chrX:50332539-50332561 | AGGTAGAGAGTACATGGTAT | AAG | No | ACATCAAAACCGACTCCATCCT | TCTGACTTTGAAGAAAATGACCAAA | 486 |
