# Supplementary material for: Identification of Neurensin-2 as a novel modulator of emotional behavior
Source: Mol Psychiatry. 2021 Mar 19;26(7):2872–85. doi: 10.1038/s41380-021-01058-5 (PMC8505262; doi:10.1038/s41380-021-01058-5)
Supplement: Supplementary file 8 — Table S6 [file 41380_2021_1058_MOESM8_ESM.pdf]

ID	Protein name						IP_1	IP_2	IP_3	Con_1	Con_2
Accession	Description	ΣCoverage	Σ# Proteins	Σ# Unique Peptides	Σ# Peptides	Σ# PSMs	A5: Area	B5: Area	C5: Area	G5: Area	H5: Area
Q9Z2Y3	Homer protein homolog 1 OS=Mus musculus GN=Homer1 PE=1 SV=2 - [HOME1_MOUSE]	69.95	9	24	25	77	1.725E8	1.739E8	1.539E8	0.000E0	0.000E0
Q99JP6	Homer protein homolog 3 OS=Mus musculus GN=Homer3 PE=1 SV=2 - [HOME3_MOUSE]	70.51	5	22	23	72	2.966E8	3.506E8	3.577E8	0.000E0	0.000E0
P48678	Prelamin-A/C OS=Mus musculus GN=Lmna PE=1 SV=2 - [LMNA_MOUSE]	35.49	2	20	20	44	5.284E7	4.831E7	5.457E7	0.000E0	0.000E0
Q9QUR6	Prolyl endopeptidase OS=Mus musculus GN=Prep PE=2 SV=1 - [PPCE_MOUSE]	21.13	1	11	11	32	1.939E7	2.836E7	2.476E7	0.000E0	0.000E0
P62192	26S protease regulatory subunit 4 OS=Mus musculus GN=Psmc1 PE=1 SV=1 - [PRS4_MOUSE]	36.36	1	10	12	31	3.167E7	4.873E8	4.179E7	0.000E0	0.000E0
B1ASP2	Tyrosine-protein kinase OS=Mus musculus GN=Jak1 PE=3 SV=1 - [B1ASP2_MOUSE]	20.12	2	18	18	30	1.528E7	1.535E7	1.512E7	0.000E0	0.000E0
Q8C2Q3	RNA-binding protein 14 OS=Mus musculus GN=Rbm14 PE=1 SV=1 - [RBM14_MOUSE]	25.71	7	14	14	29	2.883E7	4.491E7	4.882E7	0.000E0	0.000E0
Q91YU6	Leucine zipper putative tumor suppressor 2 OS=Mus musculus GN=Lzts2 PE=2 SV=3 - [LZTS2_MOUSE]	32.79	2	14	14	29	2.749E7	3.248E7	1.760E7	0.000E0	0.000E0
Q8BGJ5	MCG13402, isoform CRA_a OS=Mus musculus GN=Ptbp1 PE=2 SV=1 - [Q8BGJ5_MOUSE]	34.40	16	12	12	27	2.364E7	2.883E7	3.853E7	0.000E0	0.000E0
Q9WV32	Actin-related protein 2/3 complex subunit 1B OS=Mus musculus GN=Arpc1b PE=1 SV=4 - [ARC1B_MOUSE]	37.37	5	10	11	27	2.463E7	4.276E7	5.359E7	0.000E0	0.000E0
Q8BK64	Activator of 90 kDa heat shock protein ATPase homolog 1 OS=Mus musculus GN=Ahsa1 PE=2 SV=2 - [AHSA1_MOUSE]	38.76	1	11	11	26	4.107E7	3.947E7	4.208E7	0.000E0	0.000E0
Q8BY87	Ubiquitin carboxyl-terminal hydrolase 47 OS=Mus musculus GN=Usp47 PE=1 SV=2 - [UBP47_MOUSE]	11.85	1	12	12	25	8.489E6	1.068E7	9.749E6	0.000E0	0.000E0
B1ATZ0	HGF-regulated tyrosine kinase substrate OS=Mus musculus GN=Hgs PE=4 SV=1 - [B1ATZ0_MOUSE]	19.71	6	12	12	25	4.049E7	3.575E7	3.110E7	0.000E0	0.000E0
Q8K298	Actin-binding protein anillin OS=Mus musculus GN=Anln PE=1 SV=2 - [ANLN_MOUSE]	13.92	1	13	13	24	1.614E7	1.642E7	1.174E7	0.000E0	0.000E0
D3YX27	Serine protease HTRA2, mitochondrial OS=Mus musculus GN=Htra2 PE=4 SV=1 - [D3YX27_MOUSE]	29.11	7	7	7	21	4.623E7	3.790E7	6.221E7	0.000E0	0.000E0
P21956	Lactadherin OS=Mus musculus GN=Mfge8 PE=1 SV=3 - [MFGM_MOUSE]	24.62	1	10	10	21	3.436E7	2.457E7	3.584E7	0.000E0	0.000E0
Q99M87	DnaJ homolog subfamily A member 3, mitochondrial OS=Mus musculus GN=Dnaja3 PE=1 SV=1 - [DNJA3_MOUSE]	22.71	1	8	8	21	2.049E7	1.513E7	4.282E7	0.000E0	0.000E0
Q9EP71	Ankycorbin OS=Mus musculus GN=Rai14 PE=1 SV=1 - [RAI14_MOUSE]	11.95	1	11	11	21	4.997E6	1.406E7	9.741E6	0.000E0	0.000E0
Q9WVM1	Rac GTPase-activating protein 1 OS=Mus musculus GN=Racgap1 PE=2 SV=1 - [RGAP1_MOUSE]	21.66	4	11	11	20	1.333E7	1.629E7	1.710E7	0.000E0	0.000E0
P61222	ATP-binding cassette sub-family E member 1 OS=Mus musculus GN=Abce1 PE=2 SV=1 - [ABCE1_MOUSE]	20.03	1	10	10	19	9.106E6	1.184E7	1.485E7	0.000E0	0.000E0
P52651	Homeobox protein Rhox5 OS=Mus musculus GN=Rhox5 PE=2 SV=1 - [RHOX5_MOUSE]	50.00	3	9	9	19	2.254E7	2.096E7	1.806E7	0.000E0	0.000E0
Q61210	Rho guanine nucleotide exchange factor 1 OS=Mus musculus GN=Arhgef1 PE=1 SV=2 - [ARHG1_MOUSE]	18.80	5	12	12	19	5.850E6	1.224E7	3.350E7	0.000E0	0.000E0
O88398	Advillin OS=Mus musculus GN=Avil PE=1 SV=2 - [AVIL_MOUSE]	15.26	1	10	10	18	9.902E6	2.071E7	3.913E7	0.000E0	0.000E0
Q8R1Q8	Cytoplasmic dynein 1 light intermediate chain 1 OS=Mus musculus GN=Dync1li1 PE=1 SV=1 - [DC1L1_MOUSE]	19.12	1	9	9	18	2.069E7	1.545E7	3.439E7	0.000E0	0.000E0
Q91WQ3	Tyrosine--tRNA ligase, cytoplasmic OS=Mus musculus GN=Yars PE=2 SV=3 - [SYYC_MOUSE]	20.27	3	11	11	18	1.074E7	1.557E7	8.327E6	0.000E0	0.000E0
Q925I1	ATPase family AAA domain-containing protein 3 OS=Mus musculus GN=Atad3 PE=1 SV=1 - [ATAD3_MOUSE]	19.12	5	10	10	18	9.306E6	1.579E7	4.638E7	0.000E0	0.000E0
Q9CVB6	Actin-related protein 2/3 complex subunit 2 OS=Mus musculus GN=Arpc2 PE=1 SV=3 - [ARPC2_MOUSE]	38.67	3	8	8	18	2.094E7	3.818E7	5.789E7	0.000E0	0.000E0
Q9JKR6	Hypoxia up-regulated protein 1 OS=Mus musculus GN=Hyou1 PE=1 SV=1 - [HYOU1_MOUSE]	13.11	3	9	9	18	1.225E6	1.945E7	1.634E7	0.000E0	0.000E0
Q9R0Q6	Actin-related protein 2/3 complex subunit 1A OS=Mus musculus GN=Arpc1a PE=1 SV=1 - [ARC1A_MOUSE]	25.95	3	7	8	18	1.504E7	2.789E7	5.449E7	0.000E0	0.000E0
P46460	Vesicle-fusing ATPase OS=Mus musculus GN=Nsf PE=1 SV=2 - [NSF_MOUSE]	9.14	2	7	7	17	8.280E6	1.709E7	2.129E7	0.000E0	0.000E0
Q3THK7	GMP synthase [glutamine-hydrolyzing] OS=Mus musculus GN=Gmps PE=1 SV=2 - [GUAA_MOUSE]	15.58	1	8	8	17	8.700E6	1.185E7	1.006E7	0.000E0	0.000E0
Q3UGN9	Signal transducing adapter molecule 1 OS=Mus musculus GN=Stam PE=2 SV=1 - [Q3UGN9_MOUSE]	25.32	5	9	11	17	2.474E7	1.405E7	2.868E7	0.000E0	0.000E0
Q3UIL6	Pleckstrin homology domain-containing family A member 7 OS=Mus musculus GN=Plekha7 PE=1 SV=2 - [PKHA7_MOUSE]	15.30	4	11	11	17	1.184E7	8.725E6	1.948E7	0.000E0	0.000E0
Q99LC5	Electron transfer flavoprotein subunit alpha, mitochondrial OS=Mus musculus GN=Etfa PE=1 SV=2 - [ETFA_MOUSE]	39.94	1	9	9	17	2.298E7	2.839E7	3.700E7	0.000E0	0.000E0
P70206	Plexin-A1 OS=Mus musculus GN=Plxna1 PE=1 SV=1 - [PLXA1_MOUSE]	7.23	4	10	10	16	9.711E6	1.243E7	1.209E7	0.000E0	0.000E0
Q3T9X3	Dynamin-2 OS=Mus musculus GN=Dnm2 PE=2 SV=1 - [Q3T9X3_MOUSE]	13.14	11	9	9	16	1.158E7	4.591E6	1.084E7	0.000E0	0.000E0
Q3V3R1	Monofunctional C1-tetrahydrofolate synthase, mitochondrial OS=Mus musculus GN=Mthfd1l PE=1 SV=2 - [C1TM_MOUSE]	12.49	1	9	9	16	4.646E6	9.869E6	7.450E6	0.000E0	0.000E0
Q8R5H1	Ubiquitin carboxyl-terminal hydrolase 15 OS=Mus musculus GN=Usp15 PE=2 SV=1 - [UBP15_MOUSE]	11.42	2	10	11	16	1.090E7	1.338E7	9.390E6	0.000E0	0.000E0
Q91VD9	NADH-ubiquinone oxidoreductase 75 kDa subunit, mitochondrial OS=Mus musculus GN=Ndufs1 PE=1 SV=2 - [NDUS1_MOUSE]	12.52	1	7	7	16	5.600E6	1.151E7	1.333E7	0.000E0	0.000E0
Q9DCJ5	NADH dehydrogenase [ubiquinone] 1 alpha subcomplex subunit 8 OS=Mus musculus GN=Ndufa8 PE=1 SV=3 - [NDUA8_MOUSE]	48.84	1	7	7	16	1.318E7	1.219E7	1.167E7	0.000E0	0.000E0
Q9JLB2	MAGUK p55 subfamily member 5 OS=Mus musculus GN=Mpp5 PE=1 SV=1 - [MPP5_MOUSE]	17.19	1	10	10	16	1.944E7	2.181E7	1.770E7	0.000E0	0.000E0
E9PUE7	Active breakpoint cluster region-related protein OS=Mus musculus GN=Abr PE=4 SV=1 - [E9PUE7_MOUSE]	10.58	6	7	8	15	2.033E7	1.588E7	1.755E7	0.000E0	0.000E0
O54916	RalBP1-associated Eps domain-containing protein 1 OS=Mus musculus GN=Reps1 PE=1 SV=2 - [REPS1_MOUSE]	13.08	9	7	7	15	2.062E7	1.797E7	1.882E7	0.000E0	0.000E0
Q3UDE2	Tubulin--tyrosine ligase-like protein 12 OS=Mus musculus GN=Ttll12 PE=1 SV=1 - [TTL12_MOUSE]	19.25	2	8	8	15	2.995E6	1.833E7	1.424E7	0.000E0	0.000E0
Q3UIR3	E3 ubiquitin-protein ligase DTX3L OS=Mus musculus GN=Dtx3l PE=2 SV=1 - [DTX3L_MOUSE]	16.44	1	10	10	15	1.723E7	3.825E6	8.271E6	0.000E0	0.000E0
P51150	Ras-related protein Rab-7a OS=Mus musculus GN=Rab7a PE=1 SV=2 - [RAB7A_MOUSE]	48.31	3	8	8	14	4.079E7	2.778E7	3.700E7	0.000E0	0.000E0
O54984	ATPase Asna1 OS=Mus musculus GN=Asna1 PE=1 SV=2 - [ASNA_MOUSE]	30.46	1	8	8	14	1.093E7	2.774E7	8.435E6	0.000E0	0.000E0
P26638	Serine--tRNA ligase, cytoplasmic OS=Mus musculus GN=Sars PE=2 SV=3 - [SYSC_MOUSE]	16.41	4	7	7	14	1.446E7	1.393E7	1.468E7	0.000E0	0.000E0
P32883	GTPase KRas OS=Mus musculus GN=Kras PE=1 SV=1 - [RASK_MOUSE]	42.33	8	3	6	14	4.791E7	9.053E7	6.934E7	0.000E0	0.000E0
Q5HZK2	Neurensin-2 OS=Mus musculus GN=Nrsn2 PE=2 SV=1 - [NRSN2_MOUSE]	40.10	1	4	4	14	6.925E7	2.087E8	2.539E8	0.000E0	0.000E0
Q8BMJ2	Leucine--tRNA ligase, cytoplasmic OS=Mus musculus GN=Lars PE=2 SV=2 - [SYLC_MOUSE]	10.78	1	11	11	14	5.720E6	8.635E6	6.793E6	0.000E0	0.000E0
Q8CG48	Structural maintenance of chromosomes protein 2 OS=Mus musculus GN=Smc2 PE=1 SV=2 - [SMC2_MOUSE]	10.50	2	10	10	13	6.785E6	1.598E7	3.385E6	0.000E0	0.000E0
O09172	Glutamate--cysteine ligase regulatory subunit OS=Mus musculus GN=Gclm PE=2 SV=1 - [GSH0_MOUSE]	28.83	4	6	6	13	2.708E7	2.025E7	3.055E7	0.000E0	0.000E0
P28658	Ataxin-10 OS=Mus musculus GN=Atxn10 PE=1 SV=2 - [ATX10_MOUSE]	19.79	1	8	8	13	8.579E6	3.269E6	1.586E7	0.000E0	0.000E0
Q6PGL7	WASH complex subunit FAM21 OS=Mus musculus GN=Fam21 PE=1 SV=1 - [FAM21_MOUSE]	7.95	3	7	7	13	6.883E6	9.555E6	1.154E7	0.000E0	0.000E0
Q99K41	EMILIN-1 OS=Mus musculus GN=Emilin1 PE=1 SV=1 - [EMIL1_MOUSE]	11.80	1	8	8	13	1.170E7	2.410E7	1.394E7	0.000E0	0.000E0
Q9DCW4	Electron transfer flavoprotein subunit beta OS=Mus musculus GN=Etfb PE=1 SV=3 - [ETFB_MOUSE]	26.27	7	6	6	13	7.189E6	1.260E7	1.936E7	0.000E0	0.000E0
Q9Z2I8	Succinyl-CoA ligase [GDP-forming] subunit beta, mitochondrial OS=Mus musculus GN=Suclg2 PE=2 SV=3 - [SUCB2_MOUSE]	32.33	2	9	9	13	1.497E7	6.250E6	1.536E7	0.000E0	0.000E0
Q922B6	E3 ubiquitin-protein ligase TRAF7 OS=Mus musculus GN=Traf7 PE=1 SV=1 - [TRAF7_MOUSE]	10.61	7	6	6	12	1.121E7	1.497E7	1.666E7	0.000E0	0.000E0
F7AVU1	Malcavernin OS=Mus musculus GN=Ccm2 PE=4 SV=2 - [F7AVU1_MOUSE]	22.11	4	6	6	12	1.220E7	8.002E6	1.436E7	0.000E0	0.000E0
Q5SX75	Procollagen-proline, 2-oxoglutarate 4-dioxygenase (Proline 4-hydroxylase), alpha II polypeptide OS=Mus musculus GN=P4ha2 PE=2 SV=1 - [Q5SX75_	11.55	3	5	5	12	1.809E7	1.599E7	1.637E7	0.000E0	0.000E0
Q8BFZ9	Erlin-2 OS=Mus musculus GN=Erlin2 PE=1 SV=1 - [ERLN2_MOUSE]	20.29	1	6	6	12	1.148E7	2.458E7	2.887E7	0.000E0	0.000E0
Q8BW10	RNA-binding protein NOB1 OS=Mus musculus GN=Nob1 PE=1 SV=1 - [NOB1_MOUSE]	15.14	3	6	6	12	1.325E7	1.340E7	3.216E7	0.000E0	0.000E0
Q99PG2	Opioid growth factor receptor OS=Mus musculus GN=Ogfr PE=2 SV=1 - [OGFR_MOUSE]	14.53	1	4	4	12	1.265E7	2.082E7	2.640E7	0.000E0	0.000E0
Q9CR50	RING finger and CHY zinc finger domain-containing protein 1 OS=Mus musculus GN=Rchy1 PE=1 SV=1 - [ZN363_MOUSE]	18.39	2	5	5	12	8.094E6	7.844E6	9.433E6	0.000E0	0.000E0

Q923T9	Calcium/calmodulin-dependent protein kinase type II subunit gamma OS=Mus musculus GN=Camk2g PE=1 SV=1 - [KCC2G_MOUSE]	10.59	11	2	4	11	9.006E6	2.723E7	1.697E7	0.000E0	0.000E0
A2A6T1	Cerebellar degeneration-related protein 2-like OS=Mus musculus GN=Cdr2l PE=2 SV=1 - [CDR2L_MOUSE]	11.40	1	5	5	11	8.660E6	1.410E7	1.262E7	0.000E0	0.000E0
G3UY38	Heterogeneous nuclear ribonucleoprotein L OS=Mus musculus GN=Hnrnpl PE=4 SV=1 - [G3UY38_MOUSE]	21.27	4	8	8	11	1.035E7	8.137E6	2.980E7	0.000E0	0.000E0
G5E8Q4	Cytohesin-3 OS=Mus musculus GN=Cyth3 PE=4 SV=1 - [G5E8Q4_MOUSE]	12.54	2	3	4	11	1.148E7	1.551E7	8.665E6	0.000E0	0.000E0
Q61474	RNA-binding protein Musashi homolog 1 OS=Mus musculus GN=Msi1 PE=1 SV=1 - [MSI1H_MOUSE]	25.14	4	7	7	11	5.998E6	9.207E6	3.583E7	0.000E0	0.000E0
Q64282	Interferon-induced protein with tetratricopeptide repeats 1 OS=Mus musculus GN=Ifit1 PE=1 SV=2 - [IFIT1_MOUSE]	15.12	4	5	5	11	2.152E7	1.986E7	6.742E6	0.000E0	0.000E0
Q64339	Ubiquitin-like protein ISG15 OS=Mus musculus GN=Isg15 PE=1 SV=4 - [ISG15_MOUSE]	27.33	1	4	4	11	1.214E7	1.054E7	1.666E7	0.000E0	0.000E0
Q8C156	Condensin complex subunit 2 OS=Mus musculus GN=Ncaph PE=2 SV=1 - [CND2_MOUSE]	10.81	1	6	6	11	1.498E7	1.119E7	1.311E7	0.000E0	0.000E0
Q9DB77	Cytochrome b-c1 complex subunit 2, mitochondrial OS=Mus musculus GN=Uqcrc2 PE=1 SV=1 - [QCR2_MOUSE]	14.13	1	4	4	11	1.705E7	1.773E7	1.807E7	0.000E0	0.000E0
G3UXH4	Serine/threonine-protein kinase N2 OS=Mus musculus GN=Pkn2 PE=4 SV=1 - [G3UXH4_MOUSE]	5.35	3	5	5	10	1.120E7	1.119E7	1.336E7	0.000E0	0.000E0
B1AQR8	Galectin-9 OS=Mus musculus GN=Lgals9 PE=4 SV=1 - [B1AQR8_MOUSE]	12.50	3	4	4	10	2.348E7	4.707E7	4.029E7	0.000E0	0.000E0
G3UZ26	Serine hydroxymethyltransferase (Fragment) OS=Mus musculus GN=Shmt1 PE=3 SV=1 - [G3UZ26_MOUSE]	17.08	3	6	6	10	8.948E6	9.997E6	1.003E7	0.000E0	0.000E0
P18155	Bifunctional methylenetetrahydrofolate dehydrogenase/cyclohydrolase, mitochondrial OS=Mus musculus GN=Mthfd2 PE=1 SV=1 - [MTDC_MOUSE]	14.86	1	6	6	10	6.825E6	1.730E6	6.369E6	0.000E0	0.000E0
P25206	DNA replication licensing factor MCM3 OS=Mus musculus GN=Mcm3 PE=1 SV=2 - [MCM3_MOUSE]	8.00	1	6	6	10	7.711E6	1.201E7	9.447E6	0.000E0	0.000E0
P46061	Ran GTPase-activating protein 1 OS=Mus musculus GN=Rangap1 PE=1 SV=2 - [RAGP1_MOUSE]	13.24	1	6	6	10	1.855E7	4.206E7	1.026E7	0.000E0	0.000E0
Q8C181	Muscleblind-like protein 2 OS=Mus musculus GN=Mbnl2 PE=2 SV=2 - [MBNL2_MOUSE]	11.53	2	1	5	10	1.821E7	1.692E7	1.345E7	0.000E0	0.000E0
Q9CWE0	Protein FAM54B OS=Mus musculus GN=Fam54b PE=1 SV=1 - [FA54B_MOUSE]	16.96	4	4	4	10	1.675E7	1.143E7	2.646E7	0.000E0	0.000E0
Q9CZY3	Ubiquitin-conjugating enzyme E2 variant 1 OS=Mus musculus GN=Ube2v1 PE=1 SV=1 - [UB2V1_MOUSE]	30.61	8	4	4	10	3.967E7	3.426E7	4.543E7	0.000E0	0.000E0
Q9D2R0	Acetoacetyl-CoA synthetase OS=Mus musculus GN=Aacs PE=1 SV=1 - [AACS_MOUSE]	11.46	1	8	8	10	1.482E7	1.295E7	8.862E6	0.000E0	0.000E0
Q9DCC4	Pyrroline-5-carboxylate reductase 3 OS=Mus musculus GN=Pycrl PE=2 SV=2 - [P5CR3_MOUSE]	27.74	1	6	6	10	1.999E7	1.481E7	1.454E7	0.000E0	0.000E0
G3X957	Liprin-beta-2 OS=Mus musculus GN=Ppfibp2 PE=4 SV=1 - [G3X957_MOUSE]	8.50	2	4	7	9	9.693E6	2.084E7	4.712E6	0.000E0	0.000E0
A2AQD6	Sperm specific antigen 2 OS=Mus musculus GN=Ssfa2 PE=4 SV=1 - [A2AQD6_MOUSE]	4.51	3	5	5	9	9.725E6	1.738E7	1.255E7	0.000E0	0.000E0
E9PWV3	Ribosomal protein S6 kinase OS=Mus musculus GN=Rps6ka1 PE=3 SV=1 - [E9PWV3_MOUSE]	9.32	11	2	6	9	1.342E7	1.607E7	2.574E7	0.000E0	0.000E0
O08915	AH receptor-interacting protein OS=Mus musculus GN=Aip PE=1 SV=1 - [AIP_MOUSE]	18.79	2	5	5	9	7.924E6	2.102E7	6.322E6	0.000E0	0.000E0
O70435	Proteasome subunit alpha type-3 OS=Mus musculus GN=Psma3 PE=1 SV=3 - [PSA3_MOUSE]	16.86	1	4	4	9	9.111E6	9.646E6	1.297E7	0.000E0	0.000E0
P0CG14	Chromosome transmission fidelity protein 8 homolog isoform 2 OS=Mus musculus GN=Chtf8 PE=2 SV=1 - [CTF8A_MOUSE]	14.26	1	5	5	9	1.370E7	4.426E7	5.402E7	0.000E0	0.000E0
P63005	Platelet-activating factor acetylhydrolase IB subunit alpha OS=Mus musculus GN=Pafah1b1 PE=1 SV=2 - [LIS1_MOUSE]	16.34	2	5	5	9	1.204E7	1.345E7	9.225E6	0.000E0	0.000E0
Q5SXA5	TOM1-like protein 2 OS=Mus musculus GN=Tom1l2 PE=2 SV=1 - [Q5SXA5_MOUSE]	20.35	5	5	5	9	1.516E7	1.171E7	1.168E7	0.000E0	0.000E0
Q80Y83	Dixin OS=Mus musculus GN=Dixdc1 PE=1 SV=1 - [DIXC1_MOUSE]	9.56	1	6	6	9	1.054E7	1.016E7	1.014E7	0.000E0	0.000E0
Q8R2Q8	Bone marrow stromal antigen 2 OS=Mus musculus GN=Bst2 PE=1 SV=1 - [BST2_MOUSE]	16.28	1	3	3	9	3.714E7	1.087E7	5.337E7	0.000E0	0.000E0
Q91W59	RNA-binding motif, single-stranded-interacting protein 1 OS=Mus musculus GN=Rbms1 PE=2 SV=1 - [RBMS1_MOUSE]	11.41	6	4	4	9	1.365E7	2.178E7	5.953E6	0.000E0	0.000E0
Q9CQ65	S-methyl-5'-thioadenosine phosphorylase OS=Mus musculus GN=Mtap PE=2 SV=1 - [MTAP_MOUSE]	22.61	1	5	5	9	1.805E7	3.186E7	2.057E7	0.000E0	0.000E0
Q9D6R2	Isocitrate dehydrogenase [NAD] subunit alpha, mitochondrial OS=Mus musculus GN=Idh3a PE=1 SV=1 - [IDH3A_MOUSE]	12.57	1	5	5	9	1.531E7	1.831E7	1.713E7	0.000E0	0.000E0
Q9D8W5	26S proteasome non-ATPase regulatory subunit 12 OS=Mus musculus GN=Psmd12 PE=1 SV=4 - [PSD12_MOUSE]	8.11	3	4	4	9	7.042E6	1.098E7	1.066E7	0.000E0	0.000E0
Q9DBY8	Nuclear valosin-containing protein-like OS=Mus musculus GN=Nvl PE=1 SV=1 - [NVL_MOUSE]	7.95	1	5	5	9	9.427E6	3.592E7	1.512E7	0.000E0	0.000E0
Q9Z1W9	STE20/SPS1-related proline-alanine-rich protein kinase OS=Mus musculus GN=Stk39 PE=1 SV=1 - [STK39_MOUSE]	9.35	1	5	5	9	1.035E7	2.489E7	1.704E7	0.000E0	0.000E0
Q921R2	40S ribosomal protein S13 OS=Mus musculus GN=Rps13 PE=2 SV=1 - [Q921R2_MOUSE]	22.14	2	3	3	9	7.445E6	1.120E7	2.579E7	0.000E0	0.000E0
A0A0G2JE25	GTPase NRas (Fragment) OS=Mus musculus GN=Nras PE=1 SV=1 - [A0A0G2JE25_MOUSE]	42.67	9	1	4	9	3.865E7	1.060E8	9.217E7	0.000E0	0.000E0
O55029	Coatomer subunit beta' OS=Mus musculus GN=Copb2 PE=2 SV=2 - [COPB2_MOUSE]	8.07	1	6	6	8	6.056E6	2.004E7	2.079E7	0.000E0	0.000E0
B1AT92	Growth factor receptor bound protein 2 OS=Mus musculus GN=Grb2 PE=4 SV=1 - [B1AT92_MOUSE]	20.69	3	4	4	8	3.060E5	2.127E5	9.147E6	0.000E0	0.000E0
E9Q0U1	26S proteasome non-ATPase regulatory subunit 13 OS=Mus musculus GN=Psmd13 PE=4 SV=1 - [E9Q0U1_MOUSE]	25.73	4	5	5	8	7.776E6	1.908E7	2.619E7	0.000E0	0.000E0
G3UXL2	Ribose-phosphate pyrophosphokinase OS=Mus musculus GN=Prps1l3 PE=3 SV=1 - [G3UXL2_MOUSE]	22.33	4	4	4	8	1.801E7	1.997E7	2.442E7	0.000E0	0.000E0
P01900	H-2 class I histocompatibility antigen, D-D alpha chain OS=Mus musculus GN=H2-D1 PE=1 SV=1 - [HA12_MOUSE]	18.36	19	3	5	8	1.761E7	2.944E7	2.217E7	0.000E0	0.000E0
P03991	H-2 class I histocompatibility antigen, K-W28 alpha chain OS=Mus musculus GN=H2-K1 PE=1 SV=2 - [HA1W_MOUSE]	13.32	18	2	4	8	1.223E7	2.411E7	3.423E7	0.000E0	0.000E0
P35288	Ras-related protein Rab-23 OS=Mus musculus GN=Rab23 PE=1 SV=2 - [RAB23_MOUSE]	23.21	3	5	5	8	2.321E7	4.116E7	2.222E7	0.000E0	0.000E0
P35951	Low-density lipoprotein receptor OS=Mus musculus GN=Ldlr PE=1 SV=2 - [LDLR_MOUSE]	2.78	1	2	2	8	2.021E7	2.054E7	2.236E7	0.000E0	0.000E0
Q5NBU8	XIAP-associated factor 1 OS=Mus musculus GN=Xaf1 PE=2 SV=3 - [XAF1_MOUSE]	18.68	3	4	4	8	8.686E6	1.832E7	1.247E7	0.000E0	0.000E0
Q61081	Hsp90 co-chaperone Cdc37 OS=Mus musculus GN=Cdc37 PE=2 SV=1 - [CDC37_MOUSE]	8.44	1	2	2	8	9.772E6	1.832E7	7.080E6	0.000E0	0.000E0
Q7M739	Nuclear pore complex-associated intranuclear coiled-coil protein TPR OS=Mus musculus GN=Tpr PE=2 SV=1 - [Q7M739_MOUSE]	2.46	2	5	5	8	7.248E6	6.270E6	7.061E6	0.000E0	0.000E0
Q99MN1	Lysine--tRNA ligase OS=Mus musculus GN=Kars PE=1 SV=1 - [SYK_MOUSE]	7.90	2	4	4	8	1.280E7	1.912E7	2.032E7	0.000E0	0.000E0
Q9D1L9	Ragulator complex protein LAMTOR5 OS=Mus musculus GN=Lamtor5 PE=2 SV=1 - [LTOR5_MOUSE]	59.34	2	3	3	8	3.199E7	3.129E7	1.390E7	0.000E0	0.000E0
Q9Z0W1	Tumor necrosis factor receptor superfamily member 16 OS=Mus musculus GN=Ngfr PE=1 SV=1 - [TNR16_MOUSE]	13.19	2	3	3	8	8.068E7	1.158E7	2.868E7	0.000E0	0.000E0
A0A0U1RNT6	S-adenosylmethionine synthase isoform type-2 OS=Mus musculus GN=Mat2a PE=4 SV=1 - [A0A0U1RNT6_MOUSE]	16.30	6	5	5	8	6.861E6	3.315E6	8.656E6	0.000E0	0.000E0
F7B5B5	Protein Hnrnpr OS=Mus musculus GN=Hnrnpr PE=1 SV=1 - [F7B5B5_MOUSE]	10.36	3	1	5	8	1.591E7	2.110E7	1.438E7	0.000E0	0.000E0
P53026	60S ribosomal protein L10a OS=Mus musculus GN=Rpl10a PE=1 SV=3 - [RL10A_MOUSE]	16.59	3	4	4	7	2.064E7	1.845E7	3.347E7	0.000E0	0.000E0
E9Q9B3	Protein Spryd3 OS=Mus musculus GN=Spryd3 PE=4 SV=1 - [E9Q9B3_MOUSE]	10.63	1	4	4	7	1.109E7	3.904E6	8.292E6	0.000E0	0.000E0
P59326	YTH domain family protein 1 OS=Mus musculus GN=Ythdf1 PE=2 SV=1 - [YTHD1_MOUSE]	5.90	2	1	3	7	1.218E7	8.269E6	3.099E7	0.000E0	0.000E0
Q05DV1	NADPH--cytochrome P450 reductase OS=Mus musculus GN=Por PE=2 SV=1 - [Q05DV1_MOUSE]	4.47	4	3	3	7	1.103E7	1.095E7	1.189E7	0.000E0	0.000E0
Q71FD5	E3 ubiquitin-protein ligase ZNRF2 OS=Mus musculus GN=Znrf2 PE=1 SV=1 - [ZNRF2_MOUSE]	15.13	2	3	3	7	1.253E7	1.096E7	1.566E7	0.000E0	0.000E0
Q8C6B2	Rhotekin OS=Mus musculus GN=Rtkn PE=1 SV=3 - [RTKN_MOUSE]	10.82	4	5	5	7	4.250E6	3.599E6	2.491E6	0.000E0	0.000E0
Q8R0J7	Vacuolar protein sorting-associated protein 37B OS=Mus musculus GN=Vps37b PE=2 SV=1 - [VP37B_MOUSE]	12.63	1	3	3	7	1.063E7	1.943E7	1.779E7	0.000E0	0.000E0
Q921J2	GTP-binding protein Rheb OS=Mus musculus GN=Rheb PE=1 SV=1 - [RHEB_MOUSE]	17.93	4	4	4	7	7.811E6	1.286E7	1.670E7	0.000E0	0.000E0
Q9DBR0	A-kinase anchor protein 8 OS=Mus musculus GN=Akap8 PE=1 SV=1 - [AKAP8_MOUSE]	4.80	1	4	4	7	1.202E7	1.559E7	1.721E7	0.000E0	0.000E0
Q9QZM0	Ubiquilin-2 OS=Mus musculus GN=Ubqln2 PE=1 SV=2 - [UBQL2_MOUSE]	4.70	1	2	2	7	7.884E6	1.156E7	1.711E7	0.000E0	0.000E0
Q9R1T4	Septin-6 OS=Mus musculus GN=Sept6 PE=1 SV=4 - [SEPT6_MOUSE]	10.37	2	1	4	7	1.468E7	1.154E7	9.528E6	0.000E0	0.000E0
A0A0G2JGN4	Small nuclear ribonucleoprotein-associated protein B OS=Mus musculus GN=Snrpb PE=4 SV=1 - [A0A0G2JGN4_MOUSE]	40.00	3	3	3	7	1.590E7	1.980E7	2.460E7	0.000E0	0.000E0
E9Q9H2	DnaJ homolog subfamily C member 2 OS=Mus musculus GN=Dnajc2 PE=4 SV=1 - [E9Q9H2_MOUSE]	10.60	3	4	4	6	3.482E6	2.566E6	6.147E6	0.000E0	0.000E0

P35278	Ras-related protein Rab-5C OS=Mus musculus GN=Rab5c PE=1 SV=2 - [RAB5C_MOUSE]	18.06	5	2	3	6	2.393E7	3.768E7	1.613E7	0.000E0	0.000E0
P39429	TNF receptor-associated factor 2 OS=Mus musculus GN=Traf2 PE=1 SV=1 - [TRAF2_MOUSE]	8.58	1	3	3	6	1.076E7	6.206E6	8.443E6	0.000E0	0.000E0
P67984	60S ribosomal protein L22 OS=Mus musculus GN=Rpl22 PE=2 SV=2 - [RL22_MOUSE]	18.75	1	2	2	6	3.072E7	2.967E7	7.421E7	0.000E0	0.000E0
Q3V2R3	Beta-chimaerin OS=Mus musculus GN=Chn2 PE=2 SV=1 - [Q3V2R3_MOUSE]	14.74	7	5	5	6	8.236E6	1.592E7	3.620E6	0.000E0	0.000E0
Q5RL57	A kinase (PRKA) anchor protein 8-like OS=Mus musculus GN=Akap8l PE=2 SV=1 - [Q5RL57_MOUSE]	5.15	2	3	3	6	1.392E7	6.894E6	1.507E7	0.000E0	0.000E0
Q9CPW4	Actin-related protein 2/3 complex subunit 5 OS=Mus musculus GN=Arpc5 PE=2 SV=3 - [ARPC5_MOUSE]	28.48	3	4	4	6	3.192E7	9.431E6	3.437E7	0.000E0	0.000E0
Q9CR68	Cytochrome b-c1 complex subunit Rieske, mitochondrial OS=Mus musculus GN=Uqcrfs1 PE=1 SV=1 - [UCRI_MOUSE]	6.20	1	3	3	6	1.540E7	2.493E7	2.278E7	0.000E0	0.000E0
Q9JKX6	ADP-sugar pyrophosphatase OS=Mus musculus GN=Nudt5 PE=1 SV=1 - [NUDT5_MOUSE]	18.35	3	3	3	6	3.157E7	6.928E6	1.793E7	0.000E0	0.000E0
Q9QYH6	Melanoma-associated antigen D1 OS=Mus musculus GN=Maged1 PE=1 SV=1 - [MAGD1_MOUSE]	3.61	1	2	2	6	6.300E6	9.148E6	1.343E7	0.000E0	0.000E0
A0A0A6YW28	Ubiquitin carboxyl-terminal hydrolase 4 OS=Mus musculus GN=Usp4 PE=1 SV=1 - [A0A0A6YW28_MOUSE]	5.25	5	3	4	6	1.049E7	9.788E6	6.495E6	0.000E0	0.000E0
Q3TX55	Actin-related protein 2/3 complex subunit 4 OS=Mus musculus GN=Arpc4 PE=1 SV=1 - [Q3TX55_MOUSE]	20.51	3	2	2	5	3.412E6	8.687E6	1.753E7	0.000E0	0.000E0
Q9WVA3	Mitotic checkpoint protein BUB3 OS=Mus musculus GN=Bub3 PE=2 SV=2 - [BUB3_MOUSE]	11.35	1	3	3	5	1.480E7	1.999E7	2.387E7	0.000E0	0.000E0
P31750	RAC-alpha serine/threonine-protein kinase OS=Mus musculus GN=Akt1 PE=1 SV=2 - [AKT1_MOUSE]	9.58	3	1	3	5	1.540E7	9.435E6	6.602E6	0.000E0	0.000E0
Q6IR34	G-protein-signaling modulator 1 OS=Mus musculus GN=Gpsm1 PE=1 SV=3 - [GPSM1_MOUSE]	9.36	3	4	4	5	1.250E7	1.996E7	4.128E6	0.000E0	0.000E0
Q8BH57	WD repeat-containing protein 48 OS=Mus musculus GN=Wdr48 PE=1 SV=1 - [WDR48_MOUSE]	4.14	1	2	2	5	9.707E6	8.787E6	1.096E7	0.000E0	0.000E0
Q8BIA4	F-box/WD repeat-containing protein 8 OS=Mus musculus GN=Fbxw8 PE=1 SV=2 - [FBXW8_MOUSE]	3.85	1	2	2	5	1.848E7	1.923E7	2.148E7	0.000E0	0.000E0
Q8VCP8	Adenylate kinase isoenzyme 6 OS=Mus musculus GN=Taf9 PE=2 SV=1 - [KAD6_MOUSE]	9.88	2	2	2	5	1.128E7	1.386E7	7.157E6	0.000E0	0.000E0
Q91VK1	Basic leucine zipper and W2 domain-containing protein 2 OS=Mus musculus GN=Bzw2 PE=1 SV=1 - [BZW2_MOUSE]	4.06	1	1	2	5	8.976E6	1.476E7	9.994E6	0.000E0	0.000E0
Q9CY50	Translocon-associated protein subunit alpha OS=Mus musculus GN=Ssr1 PE=1 SV=1 - [SSRA_MOUSE]	6.64	1	2	2	5	2.298E7	1.711E7	2.758E7	0.000E0	0.000E0
Q9CZG9	PDZ domain-containing protein 11 OS=Mus musculus GN=Pdzd11 PE=1 SV=1 - [PDZ11_MOUSE]	27.14	1	3	3	5	1.489E7	2.752E7	1.850E7	0.000E0	0.000E0
Q9D1M0	Protein SEC13 homolog OS=Mus musculus GN=Sec13 PE=2 SV=3 - [SEC13_MOUSE]	11.49	1	3	3	5	1.080E7	1.144E7	2.123E7	0.000E0	0.000E0
Q9JII6	sorting	10.15	3	3	3	5	2.145E7	1.215E7	3.585E7	0.000E0	0.000E0
P61963	DDB1- and CUL4-associated factor 7 OS=Mus musculus GN=Dcaf7 PE=2 SV=1 - [DCAF7_MOUSE]	9.36	1	3	3	4	9.122E6	1.191E7	1.377E7	0.000E0	0.000E0
Q80X95	Ras-related GTP-binding protein A OS=Mus musculus GN=Rraga PE=2 SV=1 - [RRAGA_MOUSE]	6.71	3	2	2	4	8.054E6	1.400E7	8.461E6	0.000E0	0.000E0
P97376	Protein FRG1 OS=Mus musculus GN=Frg1 PE=1 SV=2 - [FRG1_MOUSE]	7.75	1	2	2	4	6.897E6	5.684E6	6.422E6	0.000E0	0.000E0
D6RE33	Enhancer of mRNA-decapping protein 4 OS=Mus musculus GN=Edc4 PE=4 SV=1 - [D6RE33_MOUSE]	1.21	4	1	1	4	3.821E6	6.483E6	1.253E7	0.000E0	0.000E0
F6ZL69	Nischarin (Fragment) OS=Mus musculus GN=Nisch PE=4 SV=1 - [F6ZL69_MOUSE]	21.48	3	2	2	4	3.352E6	4.489E6	6.124E6	0.000E0	0.000E0
Q8C373	Cancer-related nucleoside-triphosphatase homolog OS=Mus musculus GN=Ntpcr PE=2 SV=1 - [Q8C373_MOUSE]	10.74	3	1	1	4	5.429E6	1.678E7	1.373E7	0.000E0	0.000E0
Q9CY97	RNA polymerase II subunit A C-terminal domain phosphatase SSU72 OS=Mus musculus GN=Ssu72 PE=2 SV=1 - [SSU72_MOUSE]	11.86	1	2	2	4	9.148E6	7.269E6	8.399E6	0.000E0	0.000E0
Q9QX11	Cytohesin-1 OS=Mus musculus GN=Cyth1 PE=2 SV=2 - [CYH1_MOUSE]	5.78	3	1	2	4	1.536E7	2.090E7	1.406E7	0.000E0	0.000E0
E9Q5H2	Acidic leucine-rich nuclear phosphoprotein 32 family member E (Fragment) OS=Mus musculus GN=Anp32e PE=4 SV=1 - [E9Q5H2_MOUSE]	11.21	3	1	1	3	2.306E7	2.062E7	2.724E7	0.000E0	0.000E0
P56382	ATP synthase subunit epsilon, mitochondrial OS=Mus musculus GN=Atp5e PE=2 SV=2 - [ATP5E_MOUSE]	13.46	1	1	1	3	2.333E7	2.178E7	2.684E7	0.000E0	0.000E0
Q8C7U1	NEDD4-binding protein 3 OS=Mus musculus GN=N4bp3 PE=2 SV=1 - [N4BP3_MOUSE]	2.23	1	1	1	3	7.021E6	7.536E6	1.029E7	0.000E0	0.000E0
Q99MX0	Transketolase-like protein 1 OS=Mus musculus GN=Tktl1 PE=2 SV=2 - [TKTL1_MOUSE]	1.68	1	1	1	3	1.443E8	1.451E8	2.334E8	0.000E0	0.000E0

[redacted]
